# Hybrid Molecules of Azithromycin with Chloramphenicol and Metronidazole: Synthesis and Study of Antibacterial Properties

**DOI:** 10.3390/ph17020187

**Published:** 2024-01-31

**Authors:** Inna A. Volynkina, Elena N. Bychkova, Anastasiia O. Karakchieva, Alexander S. Tikhomirov, George V. Zatonsky, Svetlana E. Solovieva, Maksim M. Martynov, Natalia E. Grammatikova, Andrey G. Tereshchenkov, Alena Paleskava, Andrey L. Konevega, Petr V. Sergiev, Olga A. Dontsova, Ilya A. Osterman, Andrey E. Shchekotikhin, Anna N. Tevyashova

**Affiliations:** 1 Department of Chemistry, Lomonosov Moscow State University, Leninskie Gory 1, 119234 Moscow, Russia; karakchievaa21@gmail.com (A.O.K.); petya@genebee.msu.ru (P.V.S.); dontsova@belozersky.msu.ru (O.A.D.); osterman@yandex.ru (I.A.O.); 2 Gause Institute of New Antibiotics, B. Pirogovskaya 11, 119021 Moscow, Russia; e-bychkova@mail.ru (E.N.B.); tikhomirov.chem@gmail.com (A.S.T.); gzatonsk@gmail.com (G.V.Z.); sv_solov@mail.ru (S.E.S.); maximmartynow@mail.ru (M.M.M.); ngrammatikova@yandex.ru (N.E.G.); shchekotikhin@mail.ru (A.E.S.); 3 Belozersky Institute of Physico-Chemical Biology, Lomonosov Moscow State University, Leninskie Gory 1, 119234 Moscow, Russia; 4 Department of Molecular and Radiation Biophysics, Petersburg Nuclear Physics Institute Named by B.P. Konstantiniv of NRC “Kurchatov Institute”, Mkr. Orlova Roshcha 1, 188300 Gatchina, Russia; polesskova_ev@pnpi.nrcki.ru (A.P.); konevega_al@pnpi.nrcki.ru (A.L.K.); 5 Institute of Biomedical Systems and Biotechnologies, Peter the Great St. Petersburg Polytechnic University, Khlopina 11, 195251 Saint Petersburg, Russia; 6 NBICS Center, NRC “Kurchatov Institute”, Kurchatov Square 1, 123182 Moscow, Russia; 7 Institute of Functional Genomics, Lomonosov Moscow State University, Leninskie Gory 1, 119234 Moscow, Russia; 8 Department of Functioning of Living Systems, Shemyakin-Ovchinnikov Institute of Bioorganic Chemistry, Miklukho-Maklaya 16/10, 117997 Moscow, Russia; 9 School of Science, Constructor University, Campus Ring 1, 28759 Bremen, Germany

**Keywords:** hybrid antibiotics, azithromycin, chloramphenicol, metronidazole, antibacterial activity, antibiotic mode of action, 70S ribosome, dual reporter assay

## Abstract

The sustained rise of antimicrobial resistance (AMR) causes a strong need to develop new antibacterial agents. One of the methods for addressing the problem of antibiotic resistance is through the design of hybrid antibiotics. In this work, we proposed a synthetic route for the conjugation of an azithromycin derivative with chloramphenicol and metronidazole hemisuccinates and synthesized two series of new hybrid molecules **4a**–**g** and **5a**–**g**. While a conjugation did not result in tangible synergy for wild-type bacterial strains, new compounds were able to overcome AMR associated with the inducible expression of the *ermC* gene on a model *E. coli* strain resistant to macrolide antibiotics. The newly developed hybrids demonstrated a tendency to induce premature ribosome stalling, which might be crucial since they will not induce a macrolide-resistant phenotype in a number of pathogenic bacterial strains. In summary, the designed structures are considered as a promising direction for the further development of hybrid molecules that can effectively circumvent AMR mechanisms to macrolide antibiotics.

## 1. Introduction

The emergence of bacterial resistance to existing antimicrobial agents is a problem affecting countries worldwide. The sustained rise of antimicrobial resistance (AMR) attenuates the effectiveness of drug therapy, resulting in higher treatment costs, an increased burden on healthcare systems, and, finally, an elevated risk of death in patients [1,2,3]. While the reasons for the rapid spreading of AMR are more complex than just the use of older antibacterials, many scientists are focused on developing new approaches to the creation of antibacterial chemotherapeutic agents. One of the methods being developed to address the problem of antibiotic resistance is through the design of hybrid antibacterial substances based on two different antibiotics covalently linked to each other [4]. The chemical conjugation of several drugs with different mechanisms of action has the potential to overcome antibiotic resistance compared to the original compounds and can lead to new effective antibacterial substances. To date, several heterodimeric agents developed using this approach have reached various phases of clinical trials. The most promising molecule candidates belong to combinations of such classes as oxazolidinone and quinolone (cadazolid [5] and MCB3681 [6]), glycopeptide and cephalosporin (cefilavancin [7]), ansamycin and quinolone (TNP-2092 [8]), etc.

Macrolides represent a class of highly effective and low-toxicity antibiotics. Structurally, they contain large ester cycles (macrolactones) to which one or more deoxy sugars, typically cladinose and desosamine, are attached. One of the best-studied representatives of macrolides is the semi-synthetic antibiotic azithromycin (AZT), which has given rise to an independent subclass known as azalides. Azithromycin has been widely used in medical practice since the late 1980s to treat a broad range of bacterial infections. However, every year sees an increase in the number of pathogens developing resistance to this antibiotic [9,10,11,12]. Several promising macrolide hybrid molecules, incorporating antibiotics such as glycopeptides [13], quinolones [14,15,16], benzoxaboroles [17], tridecaptin [18], etc., have been synthesized and evaluated. The majority of the developed azithromycin conjugates have been synthesized via the transformation of its 4′′-position [13,14,15,16,17]. Structure–activity analysis of azithromycin derivatives indicates that modifications at the 4″-hydroxy group of the cladinose residue are among the most promising directions for synthesizing improved analogs capable of enhancing activity against pathogenic microorganisms including macrolide-resistant variants [14,15,19,20]. At the same time, there are no reported examples of macrolide-based hybrids with well-known antibacterial agents, such as chloramphenicol and metronidazole, in the literature.

Chloramphenicol (CLM) stands as one of the oldest antibiotics with potency against Gram-positive, Gram-negative, and anaerobic bacteria [21]. However, its high general toxicity, potential to cause bone marrow depression and aplastic anemia, along with the emergence of many CLM-resistant strains, currently limit CLM medical use [22]. Nevertheless, ongoing research and optimization of the chloramphenicol structure continue [23,24]. The mode of action of both azithromycin and chloramphenicol is implemented via the interaction with the 50S ribosomal subunit, resulting in the inhibition of protein synthesis. A combined therapy with AZT and CLM is antagonistic and may accelerate the development of resistance [25]. However, their binding sites differ at the molecular level—the macrolide blocks the nascent polypeptide exit tunnel (NPET) in the ribosome—whereas chloramphenicol blocks tRNA binding to the A-site crevice of the 50S ribosomal subunit [26,27]. Therefore, the chemical conjugation of these two antibiotics has the potential for the improvement of antibacterial properties and the benefit in circumventing AMR mechanisms associated with one of the components.

Metronidazole (MNZ) is another well-known antiprotozoal and antibacterial agent used in medical practice [21]. The biochemical reduction of the nitro group by cellular reductases determines the action of metronidazole on the DNA of protozoa and bacteria, leading to the inhibition of nucleic acid synthesis. Metronidazole is often employed in combined antimicrobial therapy, such as with clindamycin, spiramycin, trovafloxacin, and levofloxacin [28]. Importantly, some studies have demonstrated the synergistic effect of using a combination of macrolides and metronidazole [29,30]. Therefore, the chemical binding of the azithromycin molecule and metronidazole may expand the spectrum of antimicrobial activity and enhance the effectiveness of the conjugates against resistant strains.

**Figure 1:**
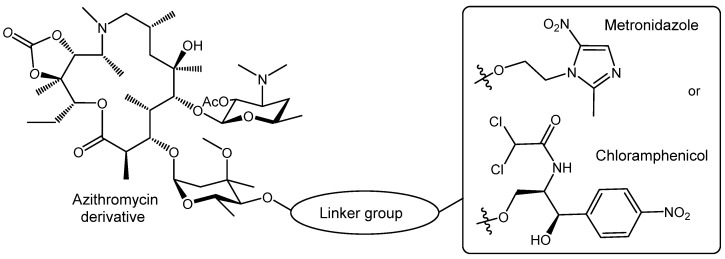
General structure of new hybrids based on azithromycin linked with chloramphenicol or metronidazole.

In this work, two sets of hybrid molecules have been synthesized and characterized. Azithromycin, at the 4″-position, was bound to chloramphenicol or metronidazole using linker fragments of different lengths and structures (Figure 1 and Figure S1, Supplementary Materials).

## 2. Results

### 2.1. Synthesis of Hybrid Molecules

**Scheme 1:**
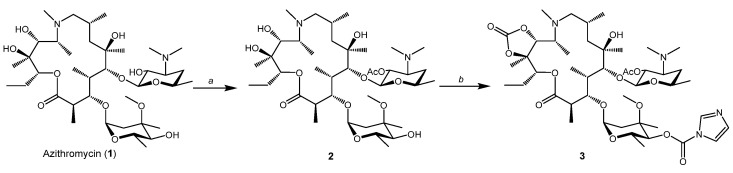
Synthesis of 2′-*O*-acetyl-4″-*O*-(imidazolylcarbonyl)azithromycin 11,12-cyclic carbonate (**3**). Reagents and conditions: (**a**) Ac_2_O, NEt_3_, DCM, rt, yield 92%; (**b**) CDI, NEt_3_, PhMe, 55 °C, yield 88%.

One of the most versatile and accessible intermediates for modifying the 4′′-position of azithromycin is 2′-acetyl-4′′-*O*-imidazolylcarbonyl-11,12-cyclic azithromycin carbonate (**3**). This compound was obtained from commercially available azithromycin (**1**) in two steps according to the previously described procedure [17]. Firstly, acylation of the 2′-hydroxy group of the desosamine residue of azithromycin (**1**) with acetic anhydride in the presence of NEt_3_ gives 2′-acetylazithromycin (**2**). Subsequently, the interaction of compound **2** with *N,N*-carbonyldiimidazole (CDI) under mild heating leads to the carbomoylation at the 4″-position of the desosamine and the formation of 11,12-cyclic carbonate, resulting in derivative **3** (Scheme 1).

**Scheme 2:**
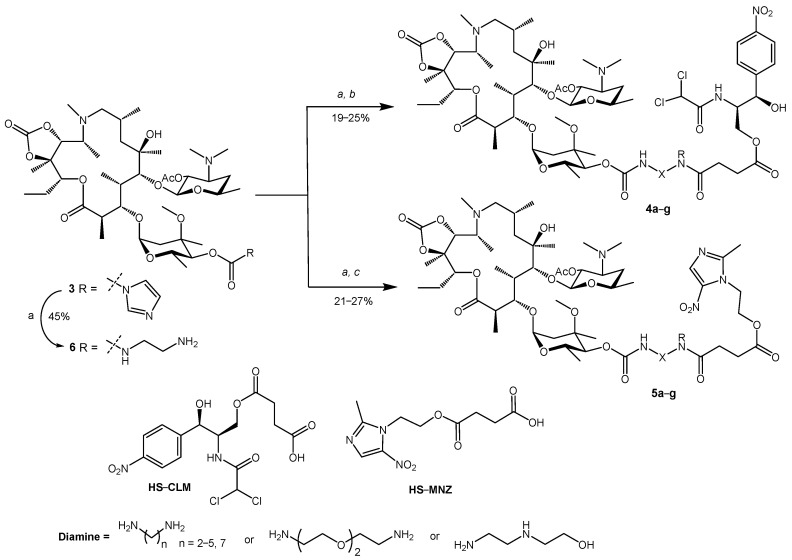
Synthesis of new hybrid antibacterials **4a**−**g** and **5a**−**g** based on azithromycin conjugated with chloramphenicol or metronidazole, respectively, and azithromycin derivative **6**. Reagents and conditions: (**a**) diamine, DBU, DMF, rt; (**b**) HS-CLM, PyBOP, DIPEA, DMF, rt, 1 h; (**c**) HS-MNZ, PyBOP, DIPEA, DMF, rt, 1 h.

For the chemical conjugation of chloramphenicol or metronidazole to azithromycin derivative **3**, we chose their hemisuccinate esters (HS-CLM [31]) and (HS-MNZ [32]) as building blocks, respectively (Scheme 2). The treatment of 2′-acetyl-4′′-*O*-imidazolylcarbonyl-11,12-cyclic azithromycin carbonate (**3**) with linear diamines of different lengths and linker fragment structures (e.g., 1,ω-diaminoalkanes, 2-(2-(2-aminoethoxy)ethoxy)ethanamine or *N*-(2-hydroxyethyl)diaminoethane) in the presence of 1,8-diazabicyclo[5.4.0]undec-7-ene (DBU) led to imidazole substitution and the formation of the corresponding carbamates. The synthesis and isolation of these intermediates as individual compounds for spectral characterization proved to be a laborious task due to a significant decrease in the yield during purification. Thus, for the transformation of 2′-acetyl-4″-*O*-imidazolylcarbonyl-11,12-cyclic azithromycin carbonate (**3**) into the target heterodimeric compounds, intermediate carbamates were used without isolation and purification. A similar approach has been applied previously in independent works [13,17,33]. Accordingly, the crude carbamates were directly reacted with HS–CLM or HS–MNZ using the peptide coupling reagent benzotriazol-1-yl-oxytripyrrolidinophosphonium-hexafluorophosphate (PyBOP), resulting in two series of hybrids, namely AZT–CLM (**4a**–**g**) and AZT–MNZ (**5a**–**g**) derivatives (Scheme 2, Table 1).

The 11,12-cyclic carbonate moiety was not removed from compounds **4a**–**g** and **5a**–**g**, as it has been shown to be important for the overcoming of efflux resistance [34]. The presence of the 2′-acetyl group also did not affect antibacterial properties, as demonstrated in a series of benzoxaborole–azithromycin conjugates [17] as well as in the Supplementary Materials (Table S3). Despite the fact that the condensation reaction leading to compounds **4a**–**g** and **5a**–**g** proceeded with good regioselectivity at the terminal amino group of the linker fragment of carbamate intermediates, purification of the target compounds **4a**–**g** and **5a**–**g** also posed sufficient difficulties resulting in low (19–27%) yields of the target compounds.

Finally, we synthesized one carbamate derivative of azithromycin (**6**) by treating **3** with 1,2-diaminoethane to evaluate the effect of conjugation on antimicrobial properties. Despite compound **6** having been previously prepared, no biological properties or NMR data are available to our knowledge. The structures of the new derivatives **4a**–**g**, **5a**–**g**, and **6** were confirmed using NMR spectroscopy and high-resolution mass spectrometry (HRMS) methods. All signals in NMR spectra for compounds **4a**–**g** and **5a**–**g** were assigned and are presented in the Supplementary Materials (Tables S1 and S2).

### 2.2. In Vitro Antimicrobial Activity

The new hybrid compounds were in vitro evaluated on a panel of bacteria, including Gram-positive strains *Streptococcus pneumoniae* ATCC 49619, *Streptococcus agalactiae* 1Cp, *Staphylococcus aureus* ATCC 29213, Gram-negative *Escherichia coli* ATCC 25922, and anaerobic pathogens *Clostridium sporogenes* ATCC 19404 and *Propionibacterium acnes* 55. Azithromycin, chloramphenicol, and metronidazole were used as positive controls. Minimum inhibitory concentration (MIC) values are provided in Table 2 and Table 3.

The series of AZT–CLM hybrids **4a**–**g** demonstrated submicromolar antibacterial activity against Gram-positive *S. pneumoniae* ATCC 49619 (Table 2), which is typically used for the initial characterization of azithromycin derivatives. The conjugation of azithromycin with chloramphenicol slightly attenuated potency compared to azithromycin; however, the MIC values of all new compounds **4a**–**g** were superior to that of chloramphenicol (Table 2). Varying the length of the alkyl linker from C_2_ to C_7_ between AZT and HS-CLM did not reveal a substantial difference in bacterial growth inhibition, while the introduction of the conformationally more flexible diethylene glycol fragment (derivative **4f**) provided hybrids with the highest potency. The absence of the chloramphenicol part in the azithromycin derivative **6** resulted in a drop in antibacterial activity, indicating the important role of the CLM-unit. In contrast to *S. pneumoniae* bacteria, compounds **4a**–**g** were not active against *S. aureus* or *E. coli* strains.

The conjugation of azithromycin with metronidazole (compounds **5a**–**g**) was also accompanied by a decrease in antibacterial activity compared to parent antibiotics. Again, *S. pneumoniae* was the most sensitive to AZT–MNZ hybrids **5a**–**g**, with activity comparable to azithromycin and significantly higher than that of metronidazole (Table 3). In contrast, the MIC values for **5a**–**g** were less pronounced toward *S. agalactiae* 1Cp and *S. aureus* ATCC 29213. For the derivatives **5a**–**g**, we generally observed the same correlation between the linker structure and antibacterial activity, namely 2-(2-(2-aminoethoxy)ethoxy)ethane as a linker contributes to the compounds **4f** and **5f** with the greatest activity. It is noteworthy that AZT–MNZ hybrid **5f** turned out to be four times more active than AZT–CLM derivative **4f** toward *S. aureus*. Importantly, the binding of azithromycin to the hemisuccinate ester of metronidazole via the ethylenediamine moiety (derivative **5a**) demonstrated a synergetic effect against the anaerobic bacteria *C. sporogenes* ATCC 19404.

The new hybrid molecules were also tested against two model *E. coli* strains known to be fully or partially resistant to macrolide antibiotics (Table 4). The common mechanism of bacterial resistance to macrolides is the dimethylation of the adenine residue A2058 of 23S rRNA (*Escherichia coli* numbering), catalyzed by methyltransferases from the Erm family [35]. As recently shown, A2058-dimethylated 70S ribosomes are completely protected from macrolide antibiotics due to the inability to coordinate the water molecule essential for the proper macrolide accommodation in the binding site [36]. The resistant phenotype can be caused by a permanent or inducible expression of the *erm* gene [37]. The permanent expression of genes such as *ermA*, *ermB*, *ermC*, etc., leads to the development of the so-called constitutive resistance to macrolide antibiotics and the manifestation of the cMLS_B_ phenotype [38], as in the case of laboratory *E. coli* Δ*tolC* pErmC strain (Table 4). However, most *erm* genes found among clinical isolates are expressed in an inducible way since methylation of the ribosome negatively affects bacterial translation [39]. Typically, their expression is regulated by the mRNA upstream sequence encoding a short leader peptide called ErmL [37,40]. Low concentrations of macrolide antibiotics (e.g., erythromycin) block the translation of the leader peptide, resulting in a rearrangement of the mRNA secondary structure and, as a consequence, the induction of Erm synthesis. This regulatory mechanism confers inductive resistance to macrolide antibiotics and manifestation of the iMLS_B_ phenotype [38], as in the case of model *E. coli* Δ*tolC* pErmCL-ErmC strain (Table 4). Two other strains—*E. coli* Δ*tolC* and *E. coli* Δ*tolC* pERMZα—were used as macrolide-sensitive controls. The *E. coli* Δ*tolC* pERMZα harbors the pERMZα plasmid, which was used as a backbone to create the pErmCL-ErmC plasmid in this work.

Indeed, we observed that all azithromycin derivatives did not inhibit the growth of *E. coli* Δ*tolC* pErmC bacteria, which were constitutively resistant to macrolides (Table 4). Therefore, we assume that new hybrids occupy the same binding site in the 50S ribosomal subunit as azithromycin. However, they were active against the *E. coli* Δ*tolC* pErmCL-ErmC strain inducibly resistant to macrolide antibiotics. As expected, we observed a 16- and 4-fold increase in the MIC values for erythromycin (ERY) and azithromycin (AZT), respectively, as well as no changes in the MIC values for chloramphenicol, compared to the macrolide-sensitive *E. coli* Δ*tolC* strain. Meanwhile, the MIC values remained the same or almost the same for most hybrid molecules. This indicates that azithromycin derivatives somehow inhibit the induction of ErmC synthesis, which might be useful against a number of macrolide-resistant pathogenic bacteria.

Among all azithromycin derivatives, we observed that AZT–MNZ hybrids **5a**–**g** were similarly or slightly more potent than AZT–CLM hybrids **4a**–**g** (e.g., derivatives **5b**, **5g**, etc., Table 4); some representatives as **4e**, **4g** or metronidazole were completely inactive against *E. coli* model strains. In general, all AZT derivatives seem to be less active than their precursor—azithromycin, which might be a consequence of poorer penetration into the cells. Of interest, the non-hybrid azithromycin derivative **6** was only 2–4 times weaker than the parental antibiotic toward *E. coli* strains (Table 4) while demonstrating totally dropped MIC values on *S. pneumoniae* (Table 2). This fact confirms the suggestion that the structure–activity relationship in antibacterials can be different and hardly predicted for Gram-positive and Gram-negative species.

### 2.3. Elucidation of the Mode of Action of New Hybrid Antibiotics

**Figure 2:**
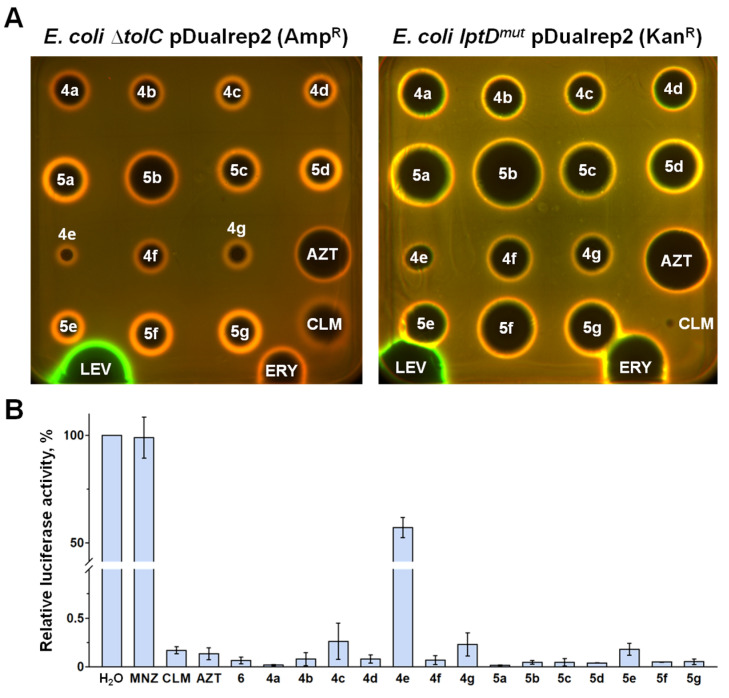
AZT–CLM and AZT–MNZ hybrid molecules **4a**–**g**, **5a**–**g** inhibit protein synthesis in bacteria cells. (**A**) Azithromycin derivatives **4a**–**g**, **5a**–**g** demonstrate strong induction of the pDualrep2 reporter similar to azithromycin (AZT) and erythromycin (ERY). Agar plates were coated with either *E. coli* Δ*tolC* pDualrep2 (Amp^R^) or *E. coli lptD^mut^* pDualrep2 (Kan^R^) reporter strain and spotted with synthesized compounds and control antibiotics—erythromycin (ERY) and levofloxacin (LEV). The plates were scanned in Cy3 (for TurboRFP) and Cy5 (for Katushka2S) channels, shown as green and red pseudocolor, respectively. (**B**) Azithromycin derivatives **4a**–**g**, **5a**–**g**, **6** inhibit protein synthesis in vitro in a cell-free bacterial translation system based on the *E. coli* S30 lysate. Relative maximal rates of the firefly luciferase (Fluc) accumulation are shown. Error-bars represent standard deviations of the mean of three independent measurements. All compounds were tested at a final concentration of 50 μM.

To further elaborate on the data regarding antibacterial activity, all CLM and MNZ derivatives of azithromycin were tested on the dual reporter system, pDualrep2 [41] (Figure 2A). This system consists of two fluorescent protein genes, *katushka2S*, and *turboRFP*. The expression of the far-red fluorescent protein gene *katushka2S* in the zone of antibiotic sublethal concentrations occurs in response to ribosome stalling during translation. The expression of the red fluorescent protein gene *turboRFP* indicates the induction of the SOS response triggered by the accumulation of DNA damage. Two different antibiotic-sensitive *E. coli* reporter strains were used to examine the contribution of two mechanisms of intrinsic antibiotic resistance. The *E. coli* Δ*tolC* pDualrep2 (Amp^R^) strain has a deletion of the *tolC* gene coding for an outer membrane efflux channel, which is involved in the active export of chemical molecules (e.g., toxins or antibiotics) and provides *E. coli* with intrinsic multidrug resistance (MDR) [42]. Meanwhile, the *E. coli lptD^mut^* pDualrep2 (Kan^R^) strain has a partial deletion (codons 330 to 352) of the *lptD* gene coding for a protein involved in lipopolysaccharide (LPS) assembly at the outer membrane surface [43]. This mutation is known to increase the membrane permeability to various antibiotics [44,45]. In addition, the *E. coli lptD^mut^* pDualrep2 (Kan^R^) strain encodes the chloramphenicol resistance gene *cat* as a selective marker.

Indeed, all conjugates demonstrated strong reporter induction similar to azithromycin and erythromycin on both reporter strains, indicating that they might negatively affect protein biosynthesis in bacteria cells (Figure 2A). The size of bacterial growth inhibition zones, as usual, reasonably displays the antibacterial activity of compounds on the tested strains. The observed activity of hybrid compounds against both reporter strains implies that both mechanisms of intrinsic antibiotic resistance—active efflux and low outer membrane permeability—negatively affect the performance of AZT derivatives against the wild-type *E. coli* ATCC 25922 strain (Table 2). It is noteworthy that AZT–CLM hybrids **4a**–**g** were active against the *E. coli lptD^mut^* pDualrep2 (Kan^R^) strain resistant to chloramphenicol due to the encoded chloramphenicol acetyltransferase (CAT), which catalyzes the transfer of an acetyl moiety to chloramphenicol, thus making it inactive (Figure 2A) [46].

**Figure 3:**
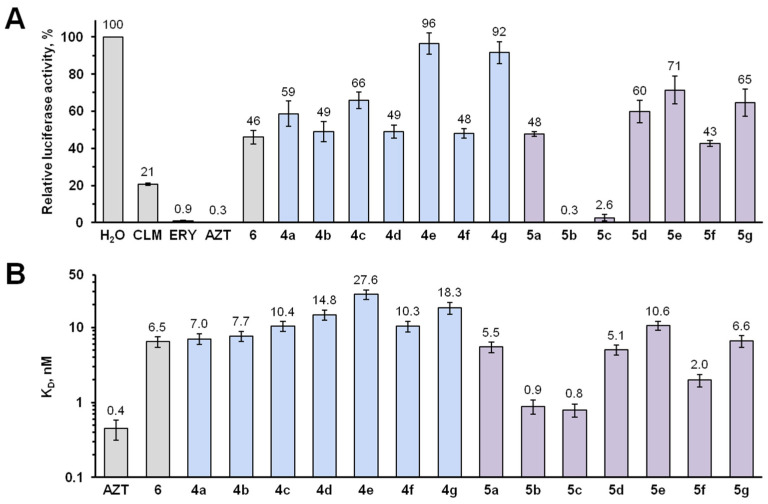
Efficiency of protein synthesis inhibition mediated by AZT–CLM (**4a**–**g**) and AZT–MNZ (**5a**–**g**) hybrid molecules in comparison with their affinity for the 70S *E. coli* ribosomes. (**A**) Azithromycin derivatives partially inhibit protein synthesis in vitro in the cell-free bacterial translation system at a final concentration of 5 μM. Relative maximal rates of the firefly luciferase (Fluc) accumulation are shown. Error-bars represent standard deviations of the mean of at least 2 independent measurements. All compounds were tested at a final concentration of 5 μM. (**B**) Apparent dissociation constants (K_D_) represent the affinity of compounds to the 70S *E. coli* ribosomes determined by the competition-binding assay using fluorescently labeled erythromycin (BODIPY-ERY). For each compound, at least 2 replicates were performed. Values are given as means, and error bars represent 95% confidence intervals.

Subsequently, azithromycin derivatives were tested in a cell-free bacterial translation system based on the *E. coli* S30 lysate (Figure 2B). All compounds, except **4e**, were proven to effectively inhibit protein synthesis in vitro at a concentration of 50 μM. As expected, no inhibition was observed in the presence of even high concentrations of metronidazole. Lower concentrations (5 μM) of the hybrids revealed that AZT–MNZ (**5a**–**g**) compounds are more efficient than AZT–CLM hybrids (**4a**–**g**) with corresponding linkers (Figure 3A). Moreover, the inhibitory activity of compounds **5b** and **5c** was comparable to that of azithromycin and significantly higher than that of its unconjugated derivative **6**. In addition, derivatives with 1,7-diaminoheptane (compounds **4e** and **5e**) and *N*-(2-hydroxyethyl)diaminoethane (compounds **4g** and **5g**) linkers were shown to have a decreased efficiency of protein synthesis inhibition in the *E. coli*-based translation system. At all steps, the purity of the compounds was confirmed using the LC-MS analysis.

To assess whether the inhibitory activity of hybrid compounds could be explained by differences in affinity for the *E. coli* ribosomes, we employed a competition-binding assay utilizing BODIPY-labeled erythromycin (BODIPY-ERY) [47,48]. All tested compounds demonstrated a decrease in fluorescence anisotropy upon an increase in their concentration (Figure S2, Supplementary Materials), indicating the ability of the conjugates to displace BODIPY-ERY from its binding site located in the NPET. Once again, we observed that AZT–MNZ conjugates **5a**–**g** possess greater potency to compete for the ribosome binding than AZT–CLM hybrids **4a**–**g** with corresponding linkers (Figure 3B and Figure S2, Supplementary Materials). In the case of AZT–CLM conjugates **5a**–**g**, a decrease in their ability to bind to the ribosome is observed along with an increase in the linker length, while for the HS-MNZ derivatives of azithromycin (**5a**–**g**), the optimal length of the alkyl linker is three (**5b**, K_D_ = 0.9 ± 0.2 nM) or four (**5c**, K_D_ = 0.8 ± 0.2 nM) carbon atoms. The affinity of these compounds for the ribosome is close to that of the parent antibiotic azithromycin (K_D_ = 0.4 ± 0.1 nM) and substantially greater than that of the 2′-acetylated carbamate derivative of azithromycin (**6**) (K_D_ = 6 ± 1 nM), which may indicate additional interactions of the MNZ moiety with the NPET elements. As in the case of in vitro translation, the incorporation of a 2-hydroxyethyl group into the structure of diaminoethane linker (compounds **4g** and **5g**) resulted in a decrease in their ability to bind to the ribosome, while the use of diethylene glycol fragment as a spacer (**4f** and **5f**) increased the affinity of the compounds (Figure 3B). Taken together, there is a correlation between the ability of azithromycin derivatives to inhibit bacterial translation in vitro and their affinity for the 70S *E. coli* ribosome (Figure S3, Supplementary Materials).

Azithromycin (AZT), like most macrolide antibiotics, inhibits protein synthesis in a sequence-specific way. It triggers translation arrest primarily at mRNA motifs encoding (R/K)x(R/K) (38%), xPx (35%), x(R/K)x (15%), xDx (10%), where x represents any amino acid residue [49]. These sequences, highly sensitive to the presence of macrolide antibiotics, are frequently used to control the expression of macrolide resistance genes. One of the best-characterized examples is the regulation of ErmC methyltransferase production due to the mRNA leader sequence encoding a short leader peptide (ErmCL) [37,40]. Low concentrations of azithromycin and some other macrolide antibiotics cause ribosome stalling during translation on the *ermCL* coding sequence. This leads to a rearrangement of the mRNA secondary structure and, as a consequence, the induction of ErmC synthesis. This particular regulatory mechanism is implemented in the *E. coli* Δ*tolC* pErmCL-ErmC strain inducibly resistant to macrolide antibiotics.

**Figure 4:**
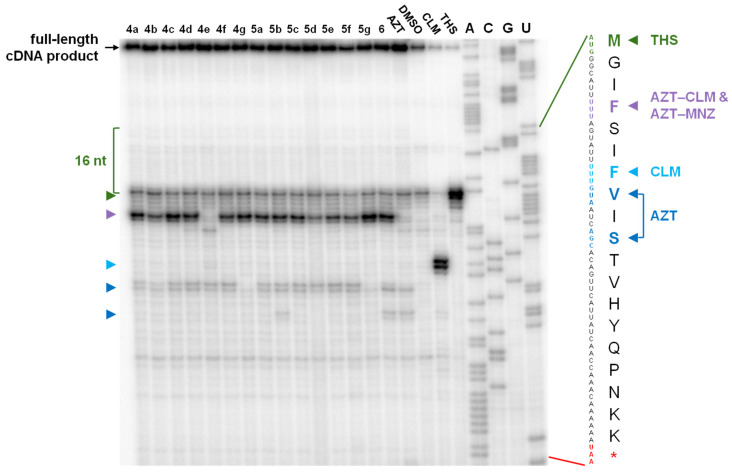
Toe-printing (or primer extension inhibition) analysis of azithromycin derivatives on *ermCL* mRNA. Ribosome stalling is detected via reverse transcription in a cell-free bacterial transcription–translation coupled system. The toe-printing bands are marked with colored arrowheads, and the A-site codons (except for the start codon, P-site codon is indicated) occupied by the stalled ribosomes are indicated in the mRNA sequence with the corresponding colors. The stop codon is marked with an asterisk. All compounds but chloramphenicol (CLM) were tested at 50 μM. Chloramphenicol (CLM) was tested at 30 μM. DMSO was used as a negative control at 0.25% final concentration. Thiostrepton (THS) was used to indicate the translation start site.

In order to validate the MIC data and compare the sequence-specificity of the AZT–CLM and AZT–MNZ derivatives, we applied a toe-printing analysis using short *ermCL* mRNA as a template (Figure 4). Azithromycin, as anticipated, induces ribosome stalling when GUA (Val) or AGC (Ser) codons enter the A-site of the ribosome (Figure 4), similar to the observations with erythromycin [40]. Chloramphenicol exhibits strong ribosome stalling when the Ser codon (AGU) occupies the E-site of the ribosome, which is consistent with previous findings [50]. None of the azithromycin derivatives displayed ribosome stalling patterns similar to those for chloramphenicol. Notably, even the introduction of a small diamine linker at the 4″-position (compound **6**) led to the appearance of premature ribosome stalling at the first Phe codon (UUU), resulting in the formation of only three amino acids long peptide—fMet-Gly-Ile. However, the stalling seems to be not completely tight since a number of AZT derivatives allowed the downstream translation as well (Figure 4, bands at the GUA (Val) codon).

Compounds with larger chemical substituents, like metronidazole or chloramphenicol (derivatives **4a**–**g**, **5a**–**g**), exhibited a complete absence of ribosome arrest at the third site, AGC (Ser) codon, in contrast to **6** (Figure 4). Translation arrest at this specific codon is considered to result in the induction of ErmC production and the formation of a macrolide-resistant phenotype. Furthermore, compounds **4g** and **5g** demonstrated a complete absence of both bands characteristic of azithromycin, making them the most promising in terms of alterations in the AZT mechanism of action.

Apparently, premature ribosome stalling appears to be the primary reason we did not observe a dramatic increase in the MIC values for AZT derivatives on the *E. coli* Δ*tolC* pErmCL-ErmC strain (Table 4). We assume that substituents at the 4″-position are directed toward the ribosomal peptidyl transferase center (PTC) and thus nonselectively interfere with the synthesis of longer polypeptides by pausing the ribosome during the elongation step at the very beginning of the mRNA coding sequence. However, a fraction of translating ribosomes goes through the first Phe codon (UUU), probably due to the displacement of the substituent by the nascent peptide. But still, it barely reaches the crucial AGC (Ser) codon essential for the ErmC synthesis regulation.

## 3. Discussion

In summary, a synthetic route for the conjugation of 2′-*O*-acetyl-11,12-cyclic azithromycin carbonate with chloramphenicol and metronidazole hemisuccinates has been developed, resulting in the preparation of two series of new hybrid molecules **4a**–**g** and **5a**–**g**. It has been found earlier that the introduction of different aromatic substituents linked to the 4′′-position of azithromycin promotes the competitive binding of derivatives at the chloramphenicol binding site in the 50S ribosomal subunit, increasing antibacterial activity against resistant strains [19,20]. In contrast, the conjugation of azithromycin with chloramphenicol using different linkers has no tangible synergy for wild-type bacterial strains. The observed results in the study could be attributed to several factors. Hybrid molecules might have a reduced ability to enter bacterial cells, impacting their overall effectiveness. The chosen conjugation position and the nature of the used linker might affect the orientation and accessibility of chloramphenicol at its binding site in the 50S ribosomal subunit. If the conjugation interferes with the optimal positioning of chloramphenicol, it could impact its ability to exert the antibacterial effect. This has been confirmed by the lower affinity of AZT–CLM hybrids **4a**–**g** for the ribosome and their lower activity to inhibit bacterial translation compared to parent antibiotics. As was demonstrated for the other type of heterodimeric molecules—protein targeting chimeras (PROTACs)—classical alkyl- or ethylene glycol-based linkers do not always provide the correct positioning of each part at the binding sites [51]. Finally, the majority of chloramphenicol’s modifications were accompanied with a bigger or lesser decrease in potency, making the natural antibiotic hard to improve [24]. This might be explained by the fact that to arrest translation, chloramphenicol must interact with specific amino acids in the nascent peptide, and even slight modifications of the antibiotic, not to mention the addition of a bulk azithromycin moiety, can interfere with such interaction. Nevertheless, this encourages further attempts at the synthesis and evaluation of new hybrid antibacterial agents, taking into account the latest structural data. Optimizing the design of hybrid compounds, including the linker and conjugation sites, is crucial for enhancing antibacterial activity and addressing challenges related to membrane permeability and conformational changes.

The analysis of the antibacterial activity of two series, **4a**–**g** and **5a**–**g**, reveals a slight preference for metronidazole over chloramphenicol for conjugation with azithromycin. The introduction of a pharmacophore with a distinct mechanism of action to azithromycin may broaden its antibacterial activity spectrum, particularly, against anaerobic strains, as demonstrated by compound **5a**. Moreover, the conjugation of azithromycin with chloramphenicol or metronidazole hemisuccinates resulted in up to 64 times better MIC values toward *S. pneumoniae* ATCC 49619 compared to benzoxaborole–azithromycin hybrids with the same 1,2-diaminoethane linker [17]. To our knowledge, the highest in vitro antibacterial activity was reached by combining azithromycin with quinolone derivatives [14].

Among both series **4a**–**g** and **5a**–**g**, the diethylene glycol linker (compounds **4f** and **5f**) corresponds to the greatest antibacterial activity against Gram-positive strains. However, the activity against the model laboratory *E. coli ΔtolC* strain turned out to be the best in the case of compounds **5b** and **5c**. Accordingly, they are superior to other AZT conjugates in experiments utilizing ribosomes derived from *E. coli*. This situation indicates how minor differences in the ribosome structure of diverse bacterial strains may affect the compound potency. A similar issue was previously mentioned in the article on tetracenomycin X [52]. Presumably, for the same reason, compound **6** exhibits relatively good antibacterial activity toward the *E. coli* Δ*tolC* strain, whereas it is totally not active against *S. pneumoniae* ATCC 49619.

It is worth noting that all AZT derivatives are not active against the *E. coli* ATCC 25922 strain, whereas the deletion of the *tolC* gene (*E. coli* Δ*tolC* strain) results in a significant decrease in their MIC values. Hence, the outer membrane protein TolC plays a role in the excretion of antibacterial substances through the efflux system.

In general, we observed that shorter alkyl linkers with two-four carbon atoms were more efficient than longer ones in *E. coli*-based experiments. The 1,7-diaminoheptane linker was found to be the least suitable. However, the introduction of two oxygen atoms into the linear linker structure (compounds **4f** and **5f**) resulted in a substantial improvement in antibacterial properties. We assume that the conformationally more flexible diethylene glycol fragment may form additional hydrogen bonds with the ribosome, thus contributing to greater potency. Interestingly, the *N*-(2-hydroxyethyl)diaminoethane linker (compounds **4g** and **5g**) might form additional hydrogen bonds within the ribosome as well, which is supported by a specific ribosome stalling pattern in the toe-printing analysis. However, the antibacterial activity of hybrids **4g** and **5g** is not superior to the activity of their analogs (compounds **4a** and **5a**, respectively), which lack the 2-hydroxyethyl fragment.

We should also mention that derivative **6**, containing two protective groups—11,12-cyclic carbonate and 2′-*O*-acetyl—and the 4′′-*O*-(2-aminoethyl)carbamoyl linker, has significantly lower antibacterial activity, affinity for the ribosome, and activity to inhibit bacterial translation in comparison with azithromycin. It is supposed that the 2′-*O*-acetyl moiety may not affect the antibacterial properties, considering the previous results [17], but still negatively influences in vitro experiments. The 2′-hydroxy group of azithromycin is known to interact with the base of A2058 in the ribosome to stabilize the complex [26]. Thus, acetylation at this position should lead to a decrease in affinity for the ribosome—exactly what we have observed. Apparently, bacterial strains are able to hydrolyze the acetyl group, which is why the presence of the protective group does not affect the MIC values. To address this issue, we have estimated the stability of the selected hybrid compounds **4a**, **5a**, and **5c** in phosphate buffer (PBS) upon incubation at 37 °C (see the Supplementary Methods section in the Supplementary Materials for more details on the experimental procedure). A partial hydrolysis of the 2′-*O*-acetyl moiety was observed over time, producing 2′-*O*-deacetyl metabolites. The structures and quantities of these metabolites were analyzed using HPLC and HRMS methods (Figures S69–S73, Supplementary Materials). Experimental MIC values for hybrids **5a**, **5c**, and their 2′-*O*-deacetyl counterparts showed no meaningful differences (Table S3, Supplementary Materials). Nevertheless, we should always bear in mind that the in vivo stability of hybrid compounds remains an open question.

Finally, we have demonstrated that azithromycin’s mode of action substantially changes upon conjugation at the 4′′-position. The resulting compounds cause premature ribosome stalling during translation, which might be crucial as it may prevent the induction of a macrolide-resistant phenotype in certain pathogenic bacterial strains. The changes in sequence-specificity due to chemical modifications of azithromycin offer a promising outlook for the future application of new hybrid molecules for circumventing AMR. Further investigations are essential to assess the activity of these new hybrids against different clinically isolated pathogenic bacterial strains, including those with M, MS_B_, cMLS_B_, and iMLS_B_ phenotypes. Moreover, current research contributes to paving the way for the development of new candidates for clinical practice.

## 4. Materials and Methods

### 4.1. Chemistry

All reagents and solvents used were obtained commercially unless otherwise specified. All steps of the chemical transformations, extraction, and purification were monitored by TLC using 60F_254_ silica gel plates (Merck, Darmstadt, Germany). Azithromycin and its derivatives were visualized on chromatograms in an iodine vapor or using a solution of ninhydrin in ethanol or (NH_4_)HSO_4_ solution followed by heating. UV-absorbing derivatives were also detected under UV_254_ light. Preparative purification of the compounds was conducted on silica gel columns (Kieselgel G60, 0.040–0.063 mm, Merck, Darmstadt, Germany). All solutions were evaporated in a vacuum at a temperature below 40 °C. High-resolution mass spectra (ESI) were recorded on a micrOTOF-Q II spectrometer (Bruker Daltonics, Billerica, MA, USA). NMR spectra were recorded in DMSO-*d*_6_ at 25 °C on Bruker Avance III 500 MHz NMR spectrometer (Bruker BioSpin, Billerica, MA, USA) with 500.2 and 125.8 MHz resonance frequencies for ^1^H and ^13^C, respectively, or on Varian Mercury 400 Plus NMR spectrometer (Agilent Technologies, Santa Clara, CA, USA) with 400 (^1^H) and 100 MHz (^13^C) in the case of compound **6**. Spectra were referenced to residual DMSO solvent signals: 2.50 ppm for DMSO-d_5_ for ^1^H spectra and 49.5 ppm for DMSO-d_6_ for ^13^C spectra. Analytical HPLC was performed on an LC-20AD chromatograph (Shimadzu, Kyoto, Japan) using a diode array UV detector and a Kromasil-100-C18, 4.6–250 mm column with 5 μm particle size (AkzoNobel, Amsterdam, Netherlands) with injection volume of 20 μL at a flow rate of 1 mL/min in the following systems: system (A), A—HCO_2_NH_4_ (0.2%) pH = 4.5, B—MeCN, concentration of B varied from 40% to 90% for 30 min; and system (B), A—HCO_2_NH_4_ (0.2%) pH = 4.5, B—MeCN, concentration of B varied from 30% to 70% for 30 min.

#### 4.1.1. 2′-*O*-Acetylazithromycin (**2**) [17,33]

A solution of azithromycin (**1**) (2.0 g, 2.67 mmol), acetic anhydride (0.5 mL, 5.34 mmol), and Et_3_N (1.48 mL, 10.68 mmol) in dichloromethane (20 mL) was stirred for 24 h at room temperature. The reaction mixture was diluted with 5% aqueous NaHCO_3_ (20 mL), and the product was extracted with dichloromethane (3 × 10 mL). The combined organic layers were washed with brine (15 mL), dried over Na_2_SO_4_, and the solvent was evaporated. The crude residue was dried in vacuum. The yield of **2** is 1.93 g (92%), white solid, mp 164–167 °C (mp 167–170 °C [53]); R_f_ = 0.50 (DCM/methanol, 10:1). HRMS (ESI) calculated for C_40_H_75_N_2_O_13_ [M + H]^+^: 791.5261; found: 791.5333.

#### 4.1.2. 2′-*O*-Acetyl-4″-*O*-acylimidazolylazithromycin-11,12-cyclic carbonate (**3**) [17,33]

A solution of 2′-*O*-acetylazithromycin (**2**) (1.5 g, 1.90 mmol), Et_3_N (0.68 mL, 4.33 mmol), and CDI (1.23 g, 7.60 mmol) in toluene (20 mL) was stirred at 55 °C for 24 h. The reaction mixture was then diluted with 5% aqueous NaHCO_3_ (20 mL), and the product was extracted with toluene (3 × 10 mL). The combined organic layers were washed with brine (15 mL), dried over Na_2_SO_4_, and the solvent was evaporated. The crude residue was dried in vacuum. The yield of **3** is 1.52 g (88%), white solid, mp 114–117 °C (mp 117–120 °C [53]); R_f_ = 0.61 (DCM/methanol, 10:1); HRMS (ESI) calculated for C_45_H_75_N_4_O_15_ [M + H]^+^: 911.5223; found: 911.5287.

#### 4.1.3. 2′-*O*-Acetyl-4′′-*O*-((2-(4-((2*R*,3*R*)-2-(2,2-dichloroacetamido)-3-hydroxy-3-(4-nitrophenyl)propoxy)-4-oxobutanamido)ethyl)carbamoyl)-11,12-cyclic carbonate of azithromycin (**4a**)

A solution of the compound **3** (0.91 g, 1.00 mmol), 1,8-diazabicyclo[5.4.0]undec-7-ene (DBU, 0.30 mL, 2.00 mmol) and 1,2-diaminoethane (0.20 g, 3.00 mmol) in DMF (10 mL) was stirred for 2 h at room temperature. The reaction mixture was diluted with 5% aqueous NaHCO_3_ (20 mL), and the product was extracted with ethyl acetate (3 × 15 mL). The combined organic layers were washed with brine (15 mL), and dried over Na_2_SO_4_. The solvent was evaporated, yielding 2′-*O*-acetyl-4′′-*O*-((2-aminoethyl)carbamoyl)-11,12-cyclic carbonate of azithromycin as a white solid, which was used for the next step without purification.

The crude powder of 2′-*O*-acetyl-4′′-*O*-((2-aminoethyl)carbamoyl)-11,12-cyclic carbonate of azithromycin was dissolved in DMF (5 mL) and added to the solution of chloramphenicol hemisuccinate [31] (0.47 g, 1.1 mmol), DIPEA (0.52 mL, 3 mmol), and PyBOP (0.78 g, 1.5 mmol) in DMF (5 mL). The reaction mixture was stirred for 1 h at room temperature, and diluted with 5% aqueous NaHCO_3_ solution (10 mL). The product was extracted with ethyl acetate (3 × 15 mL), the combined organic layers were washed with brine (10 mL), dried over Na_2_SO_4_, and the solvent was evaporated. The crude residue was purified via column chromatography (CHCl_3_:MeOH, 15:1) to give 0.26 g (20%) of compound **4a** as a white powder; R_f_ = 0.31 (DCM/MeOH, 7:1). HPLC: *t*_R_ = 13.6 min (system A). ^1^H and ^13^C NMR spectra are given in Table S1 and Figures S19 and S20, Supplementary Materials. HRMS (ESI) calculated for C_59_H_93_Cl_2_N_6_O_22_ [M + H]^+^: 1307.5715; found: 1307.5711 (Figure S4, Supplementary Materials).

#### 4.1.4. 2′-*O*-Acetyl-4′′-*O*-((3-(4-((2*R*,3*R*)-2-(2,2-dichloroacetamido)-3-hydroxy-3-(4-nitrophenyl)propoxy)-4-oxobutanamido)propyl)carbamoyl)-11,12-cyclic carbonate of azithromycin (**4b**)

This compound was prepared as described for **4a** using 1,3-diaminopropane. White powder, yield 21%; R_f_ = 0.54 (DCM/MeOH, 7:1). HPLC: *t*_R_ = 14.0 min (system A). ^1^H and ^13^C NMR spectra are given in Table S1 and Figures S21 and S22, Supplementary Materials. HRMS (ESI) calculated for C_60_H_97_Cl_2_N_6_O_22_ [M + H]^+^: 1321.5871; found: 1321.5891 (Figure S5, Supplementary Materials).

#### 4.1.5. 2′-*O*-Acetyl-4′′-*O*-((4-(4-((2*R*,3*R*)-2-(2,2-dichloroacetamido)-3-hydroxy-3-(4-nitrophenyl)propoxy)-4-oxobutanamido)butyl)carbamoyl)-11,12-cyclic carbonate of azithromycin (**4c**)

This compound was prepared as described for **4a** using 1,4-diaminobutane. White powder, yield 24%; R_f_ = 0.48 (DCM/MeOH, 7:1). HPLC: *t*_R_ = 14.3 min (system A). ^1^H and ^13^C NMR spectra are given in Table S1 and Figures S23–S26, Supplementary Materials. HRMS (ESI) calculated for C_61_H_97_Cl_2_N_6_O_22_ [M + H]^+^: 1335.6027; found: 1335.6051 (Figure S6, Supplementary Materials).

#### 4.1.6. 2′-*O*-Acetyl-4′′-*O*-((5-(4-((2*R*,3*R*)-2-(2,2-dichloroacetamido)-3-hydroxy-3-(4-nitrophenyl)propoxy)-4-oxobutanamido)pentyl)carbamoyl)-11,12-cyclic carbonate of azithromycin (**4d**)

This compound was prepared as described for **4a** using 1,5-diaminopentane. White powder, yield 25%; R_f_ = 0.50 (DCM/MeOH, 7:1). HPLC: *t*_R_ = 15.1 min (system A). ^1^H and ^13^C NMR spectra are given in Table S1 and Figures S27 and S28, Supplementary Materials. HRMS (ESI) calculated for C_62_H_99_Cl_2_N_6_O_22_ [M + H]^+^: 1349.6184; found: 1349.6141 (Figure S7, Supplementary Materials).

#### 4.1.7. 2′-*O*-Acetyl-4′′-*O*-((7-(4-((2*R*,3*R*)-2-(2,2-dichloroacetamido)-3-hydroxy-3-(4-nitrophenyl)propoxy)-4-oxobutanamido)heptyl)carbamoyl)-11,12-cyclic carbonate of azithromycin (**4e**)

This compound was prepared as described for **4a** using 1,7-diaminoheptane. White powder, yield 25%; R_f_ = 0.55 (DCM/MeOH, 7:1). HPLC: *t*_R_ = 17.1 min (system A). ^1^H and ^13^C NMR spectra are given in Table S1 and Figures S29–S32, Supplementary Materials. HRMS (ESI) calculated for C_64_H_103_Cl_2_N_6_O_22_ [M + H]^+^: 1377.6497; found: 1377.6511 (Figure S8, Supplementary Materials).

#### 4.1.8. 2′-*O*-Acetyl-4′′-*O*-((8-(4-((2*R*,3*R*)-2-(2,2-dichloroacetamido)-3-hydroxy-3-(4-nitrophenyl)propoxy)-4-oxobutanamido)-2-(2-(2-aminoethoxy)ethoxy)ethyl)carbamoyl)-11,12-cyclic carbonate of azithromycin (**4f**)

This compound was prepared as described for **4a** using 2-(2-(2-aminoethoxy)ethoxy)ethane. White powder, yield 22%; R_f_ = 0.53 (DCM/MeOH, 7:1). HPLC: *t*_R_ = 13.9 min (system A). ^1^H and ^13^C NMR spectra are given in Table S1 and Figures S33–S36, Supplementary Materials. HRMS (ESI) calculated for C_63_H_101_Cl_2_N_6_O_24_ [M + H]^+^: 1395.6239; found: 1395.6237 (Figure S9, Supplementary Materials).

#### 4.1.9. 2′-*O*-Acetyl-4′′-*O*-((2-(*N*-(2-hydroxyethyl))-2-(4-((2*R*,3*R*)-2-(2,2-dichloroacetamido)-3-hydroxy-3-(4-nitrophenyl)propoxy)-4-oxobutanamido)ethyl)carbamoyl)-11,12-cyclic carbonate of azithromycin (**4g**)

This compound was prepared as described for **4a** using *N*-(2-hydroxyethyl)diaminoethane. White powder, yield 19%; R_f_ = 0.39 (DCM/MeOH, 7:1). HPLC: *t*_R_ = 12.9 min (system A). ^1^H and ^13^C NMR spectra are given in Table S1 and Figures S37–S40, Supplementary Materials. HRMS (ESI) calculated for C_61_H_96_Cl_2_N_6_O_23_ [M + H]^+^: 1351.5977; found: 1351.5975 (Figure S10, Supplementary Materials).

#### 4.1.10. 2′-*O*-Acetyl-4′′-*O*-((2-(4-(2-(2-methyl-5-nitro-1*H*-imidazol-1-yl)ethoxy)-4-oxobutanamido)ethyl)carbamoyl)-11,12-cyclic carbonate of azithromycin (**5a**)

This compound was prepared as described for **4a** using metronidazole hemisuccinate [32]. White powder, yield 24%; R_f_ = 0.36 (DCM/MeOH, 7:1). HPLC: *t*_R_ = 9.1 min (system A). ^1^H and ^13^C NMR spectra are given in Table S2 and Figures S41 and S42, Supplementary Materials. HRMS (ESI) calculated for C_54_H_90_N_7_O_20_ [M + H]^+^: 1156.6235; found: 1156.6227 (Figure S11, Supplementary Materials).

#### 4.1.11. 2′-*O*-Acetyl-4′′-*O*-((3-(4-(2-(2-methyl-5-nitro-1*H*-imidazol-1-yl)ethoxy)-4-oxobutanamido)propyl)carbamoyl)-11,12-cyclic carbonate of azithromycin (**5b**)

This compound was prepared as described for **4a** using 1,3-diaminopropane and metronidazole hemisuccinate [32]. White powder, yield 26%; R_f_ = 0.23 (DCM/MeOH, 7:1). HPLC: *t*_R_ = 15.8 min (system B). ^1^H and ^13^C NMR spectra are given in Table S2 and Figures S43–S46, Supplementary Materials. HRMS (ESI) calculated for C_55_H_92_N_7_O_20_ [M + H]^+^: 1170.6392; found: 1170.6282 (Figure S12, Supplementary Materials).

#### 4.1.12. 2′-*O*-Acetyl-4′′-*O*-((4-(4-(2-(2-methyl-5-nitro-1*H*-imidazol-1-yl)ethoxy)-4-oxobutanamido)butyl)carbamoyl)-11,12-cyclic carbonate of azithromycin (**5c**)

This compound was prepared as described for **4a** using 1,4-diaminobutane and metronidazole hemisuccinate [32]. White powder, yield 25%; R_f_ = 0.46 (DCM/MeOH, 7:1). HPLC: *t*_R_ = 15.3 min (system B). ^1^H and ^13^C NMR spectra are given in Table S2 and Figures S47–S50, Supplementary Materials. HRMS (ESI) calculated for C_56_H_94_N_7_O_20_ [M + H]^+^: 1184.6548; found: 1184.6530 (Figure S13, Supplementary Materials).

#### 4.1.13. 2′-*O*-Acetyl-4′′-*O*-((5-(4-(2-(2-methyl-5-nitro-1*H*-imidazol-1-yl)ethoxy)-4-oxobutanamido)pentyl)carbamoyl)-11,12-cyclic carbonate of azithromycin (**5d**)

This compound was prepared as described for **4a** using 1,5-diaminopentane and metronidazole hemisuccinate [32]. White powder, yield 27%; R_f_ = 0.43 (DCM/MeOH, 7:1). HPLC: *t*_R_ = 10.5 min (system A). ^1^H and ^13^C NMR spectra are given in Table S2 and Figures S51–S54, Supplementary Materials. HRMS (ESI) calculated for C_57_H_96_N_7_O_20_ [M + H]^+^: 1198.6705; found: 1198.6682 (Figure S14, Supplementary Materials).

#### 4.1.14. 2′-*O*-Acetyl-4′′-*O*-((7-(4-(2-(2-methyl-5-nitro-1*H*-imidazol-1-yl)ethoxy)-4-oxobutanamido)heptyl)carbamoyl)-11,12-cyclic carbonate of azithromycin (**5e**)

This compound was prepared as described for **4a** using 1,7-diaminoheptane and metronidazole hemisuccinate [32]. White powder, yield 26%; R_f_ = 0.65 (DCM/MeOH, 7:1). HPLC: *t*_R_ = 12.4 min (system A). ^1^H and ^13^C NMR spectra are given in Table S2 and Figures S55–S58, Supplementary Materials. HRMS (ESI) calculated for C_59_H_100_N_7_O_20_ [M + H]^+^: 1226.7018; found: 1226.7078 (Figure S15, Supplementary Materials).

#### 4.1.15. 2′-*O*-Acetyl-4′′-*O*-((8-(4-(2-(2-methyl-5-nitro-1*H*-imidazol-1-yl)ethoxy)-4-oxobutanamido)-2-(2-(2-aminoethoxy)ethoxy)ethyl)carbamoyl)-11,12-cyclic carbonate of azithromycin (**5f**)

This compound was prepared as described for **4a** using 2-(2-(2-aminoethoxy)ethoxy)ethane and metronidazole hemisuccinate [32]. White powder, yield 23%; R_f_ = 0.41 (DCM/MeOH, 7:1). HPLC: *t*_R_ = 7.7 min (system A). ^1^H and ^13^C NMR spectra are given in Table S2 and Figures S59–S62, Supplementary Materials. HRMS (ESI) calculated for C_59_H_100_N_7_O_20_ [M + H]^+^: 1244.6759; found: 1244.6773 (Figure S16, Supplementary Materials).

#### 4.1.16. 2′-*O*-Acetyl-4′′-*O*-((2-(*N*-(2-hydroxyethyl))-2-(4-(2-(2-methyl-5-nitro-1*H*-imidazol-1-yl)ethoxy)-4-oxobutanamido)ethyl)carbamoyl)-11,12-cyclic carbonate of azithromycin (**5g**)

This compound was prepared as described for **4a** using *N*-(2-hydroxyethyl)diaminoethane and metronidazole hemisuccinate [32]. White powder, yield 21%; R_f_ = 0.32 (DCM/MeOH, 7:1). HPLC: *t*_R_ = 13.4 min (system B). ^1^H and ^13^C NMR spectra are given in Table S2 and Figures S63–S66, Supplementary Materials. HRMS (ESI) calculated for C_56_H_94_N_7_O_21_ [M + H]^+^: 1200.6497; found: 1200.6533 (Figure S17, Supplementary Materials).

#### 4.1.17. 2′-*O*-Acetyl-4′′-*O*-((2-aminoethyl)carbamoyl)-11,12-cyclic carbonate of azithromycin (**6**)

A solution of the compound **3** (0.18 g, 0.20 mmol), DBU (60 μL, 0.40 mmol), and 1,2-diaminoethane (40 mg, 0.60 mmol) in DMF (2 mL) was stirred for 2 h at room temperature. The reaction mixture was diluted with 5% aqueous NaHCO_3_ (20 mL), and the product was extracted with ethyl acetate (3 × 10 mL). The combined organic layers were washed with brine (15 mL), dried over Na_2_SO_4_, and the solvent was evaporated, yielding **6** as a white solid. The crude residue was purified via column chromatography (CHCl_3_:MeOH:NH_3 (25% aq.)_, 5:1:0.02) to give 81 mg (45%) of compound **6** as a white powder. R_f_ = 0.52 (DCM:MeOH:NH_3 (25% aq.)_, 5:1:0.05). ^1^H NMR (DMSO-*d*_6_, δ ppm): 6.98–6.90 (m, 1H); 6.72 (s, 1H); 4.91–4.83 (m, 1H); 4.82–4.73 (m, 1H); 4.58–4.50 (m, 1H); 4.42–4.35 (m, 1H); 4.26–4.13 (m, 3H); 3.67–3.57 (m, 1H); 3.45–3.31 (m, 3H); 3.20 (s, 3H); 3.12–3.06 (m, 1H); 3.05–2.94 (m, 2H); 2.80–2.74 (m, 1H); 2.58–2.51 (m, 1H); 2.34–2.25 (m, 2H); 2.21–2.10 (m, 7H); 2.06 (s, 3H); 1.96–1.88 (m, 4H); 1.88–1.78 (m, 2H); 1.73–1.63 (m, 2H); 1.42 (s, 3H); 1.36–1.29 (m, 2H); 1.16–1.07 (m, 10H); 1.07–1.00 (m, 6H); 0.94 (d, 3H, *J* = 5.5 Hz); 0.86–0.76 (m, 9H). ^13^C NMR (DMSO-*d*_6_, δ ppm): 177.7; 169.5; 156.9; 153.2; 100.2; 94.9; 86.4; 85.5; 83.0; 78.2; 77.5; 75.9; 73.3; 71.8; 67.8; 66.8; 63.2; 60.2; 49.3; 44.6; 44.2; 42.0; 41.2; 40.9; 35.0; 34.6; 30.4; 27.4; 25.8; 22.6; 21.9; 21.6; 21.5; 20.9; 18.3; 15.2; 13.8; 10.7; 9.5; 5.6. ^1^H and ^13^C NMR spectra are given in Figures S67 and S68, Supplementary Materials. HRMS (ESI) calculated for C_44_H_79_N_4_O_15_ [M + H]^+^: 903.5536; found: 903.5569 (Figure S18, Supplementary Materials).

### 4.2. Plasmids and Cloning

Plasmids pERMCTP [54] (referred to here as pErmC) and pERMZα [55] were kindly provided by Prof. Alexander S. Mankin, University of Illinois, Chicago, IL, USA. The pDualrep2 (Amp^R^) plasmid was prepared as described previously [41].

In order to create the construct pErmCL-ErmC, the vector backbone was amplified using pZa-fwd (5′-TAAGAATTCTCTAGCCCG-3′) and pZa-rev (5′-CTTAAGGTTCATTATAACCCTC-3′) primers, and the pERMZα plasmid as a template. The *ermC* gene was amplified from the pERMCTP plasmid with ErmC-fwd (5′-GGGTTATAATGAACCTTAAGAACGAGAAAAATATAAAACACAGTC-3′) and ErmC-rev (5′-TAGGCGGGCTAGAGAATTC-3′) primers. The joining of two DNA fragments was performed using the NEBuilder*^®^* HiFi DNA Assembly Master Mix (New England Biolabs, Ipswich, MA, USA). Sequences of intermediate products and the final construct were confirmed by Sanger sequencing with appropriate primers.

### 4.3. Bacterial Strains and Media

To evaluate the antimicrobial activity of hybrid molecules, clinical and reference strains obtained from the Laboratory of Medical Microbiology of the State Scientific Center of Antibiotics (Moscow, Russia) were used. The activity of azithromycin derivatives conjugated with chloramphenicol was tested on the following reference strains: *Streptococcus pneumoniae* ATCC 49619, *Staphylococcus aureus* ATCC 29213, and *Escherichia coli* ATCC 25922. Additionally, azithromycin derivatives conjugated with metronidazole were tested on the *Streptococcus agalactiae* 1Cp strain, *Streptococcus pneumoniae* ATCC 6305 strain (see Table S3, Supplementary Materials), on the anaerobic Gram-positive *Clostridium sporogenes* ATCC 19404 strain, and *Propionibacterium acnes* 55 clinical isolate.

The *E. coli* JW5503 strain with a deletion of the *tolC* gene (referred to here as *E. coli* Δ*tolC*) was kindly provided by Prof. Hironori Niki, National Institute of Genetics, Japan [56]. The *E. coli* BW25113 strain with a partial deletion of the *lptD* gene, codons 330 to 352 (referred to here as *E. coli lptD^mut^*) was kindly provided by Prof. Alexander S. Mankin, University of Illinois, Chicago, IL, USA [44]. The *E. coli* Δ*tolC* strain was transformed with the pDualrep2 (Amp^R^) plasmid [41], whereas the *E. coli lptD^mut^* strain was transformed with the pDualrep2 (Kan^R^) plasmid (not yet published). Both *E. coli* strains were grown at 37 °C in Luria–Bertani (LB) medium supplemented with either 100 μg/mL ampicillin or 50 μg/mL kanamycin.

The *E. coli* Δ*tolC* strain was also transformed with the pErmC, pErmCL-ErmC, and pERMZα plasmids. The resulting strains were grown at 37 °C in LB medium supplied with 100 μg/mL ampicillin.

### 4.4. In Vitro Antimicrobial Activity

Minimum inhibitory concentration (MIC) values for facultative anaerobic bacteria were determined using the broth microdilution method outlined in the CLSI guidelines [57]. Initially, all compounds were dissolved in dimethyl sulfoxide (DMSO) to achieve a concentration of 10 mg/mL. Subsequently, they were diluted with the Mueller–Hinton nutrient broth (MHB) supplemented with 5% defibrinated horse blood (for *Streptococcus* spp.) to a concentration of 64 μg/mL. Following 2-fold serial dilutions of the substances, bacterial cultures in the log growth phase were added to 96-well plates at a concentration of 2.5–5 × 10^5^ CFU/mL, and the plates were incubated at 37 °C for 16–20 h.

The antimicrobial activity against anaerobic bacteria was evaluated using the agar dilution method in accordance with the CLSI document M11-A9 [58]. Twofold serial dilutions of compounds, at 10 times the desired concentration, were prepared in water and diluted (1:10) with the Brucella Agar medium containing 5% defibrinated horse blood, 5 mg/L hemin, and 1 mg/L vitamin K. The resulting plates were inoculated with approximately 10^5^ CFU per spot using a replicator and incubated anaerobically at 36 °C for 48 h.

MIC values for a panel of *E. coli* Δ*tolC* strains were determined via the broth microdilution assay as described here [59] with minor modifications. Briefly, the overnight *E. coli* culture was diluted to an OD_600_ of 0.01 in the fresh LB medium supplied with 100 μg/mL ampicillin, if required. Then, the cells were additionally incubated for 1–1.5 h at 37 °C with shaking at 200 rpm (Shaker-Incubator ES-20/80, BioSan, Riga, Latvia). Meanwhile, 100 μL of the liquid growth medium was placed in each well of a 96-well sterile plate (200 μL was placed in column 1). Solutions of tested compounds were added in the first column of the plate at 2 times the final concentration of 200 μg/mL. Then, 2-fold serial dilutions were performed. Immediately before use, bacterial inoculum was diluted (1:100) in the growth medium, and 100 μL of it was transferred in each well except for the sterility control column. The plate was incubated overnight (16–20 h) at 37 °C with constant shaking at 200 rpm (Shaker-Incubator ES-20/80, BioSan, Riga Latvia). Cell growth was assessed by scanning the OD_600_ with a VICTOR X5 Multilabel Plate Reader (PerkinElmer, Waltham, MA, USA).

Minimum inhibitory concentration (MIC) was defined as the lowest concentration of a chemical compound that inhibits the visible growth of the bacterial strain. At least three replicates of MIC measurements were performed.

### 4.5. Dual Reporter Assay on Agar Plates

The overnight cultures of reporter strains diluted 5 times with fresh LB medium were plated on LB agar medium supplied with either 100 µg/mL ampicillin or 50 μg/mL kanamycin. The tested compounds **4a**–**g** (20 mg/mL, 1 μL), **5a**–**g** (20 mg/mL, 1 μL), azithromycin (AZT, 20 mM, 1 μL), chloramphenicol (CLM, 1 mg/mL, 1 μL), levofloxacin (LEV, 25 μg/mL, 1 μL), erythromycin (ERY, 5 mg/mL, 1 μL), were applied on the surface of dried agar plates covered with a reporter strain. Following overnight incubation at 37 °C, the plates were scanned using a ChemiDoc™ Imaging System (Bio-Rad Laboratories, Hercules, CA, USA) with two channels: Cy3 (emission filter 605 ± 50 nm, green pseudocolor) for TurboRFP fluorescence, and Cy5 (emission filter 695 ± 50 nm, red pseudocolor) for Katushka2S fluorescence. The results were visualized using the Image Lab™ software (version 6.0.1).

### 4.6. In Vitro Translation in a Cell-Free Bacterial System

The inhibition of firefly luciferase (Fluc) synthesis by new azithromycin derivatives was assessed with the *E. coli* S30 Extract System for Linear Templates (Promega, Madison, WI, USA). Each reaction (5 μL total volume) was supplied with 0.1 mM mixture of all canonical amino acids, 4 U of RiboLock RNase Inhibitor (Thermo Fisher Scientific, Waltham, MA, USA), 0.1 mM of D-luciferin (Sigma-Aldrich, Burlington, MA, USA), 50 ng of firefly luciferase (Fluc) mRNA, and a chemical compound at a final concentration, as indicated in the main text, or nuclease-free water instead. Before the addition of mRNA, reaction tubes were pre-incubated at RT for 5 min and then placed back on ice. After the addition of mRNA, reaction mixtures were immediately subjected to continuous chemiluminescence measurement using the VICTOR X5 Multilabel Plate Reader (PerkinElmer, Waltham, MA, USA) at 37 °C for 30 min. Maximal rates of the firefly luciferase (Fluc) accumulation were calculated using the OriginPro 7.5 software. The values were normalized to a positive control (nuclease-free water, assigned a value of 100%). The results were visualized using the QtiPlot software (version 0.9.8.9).

### 4.7. In Vitro Competition-Binding Assay with E. coli Ribosomes

Affinity of compounds to the 70S *E. coli* ribosomes (isolated from the *E. coli* MRE600 strain according to a published procedure [60]) was evaluated with a competition-binding assay using fluorescently labeled erythromycin (BODIPY-ERY) as described earlier [47,48]. Briefly, BODIPY-ERY (16 nM) was mixed with ribosomes (34 nM) in 384-well plates in the buffer containing 20 mM HEPES-KOH (pH 7.5), 50 mM NH_4_Cl, 10 mM Mg(CH_3_CO_2_)_2_, 4 mM β-mercaptoethanol, and 0.05% (*v*/*v*) Tween-20. Solutions of the tested compounds were added to the obtained complexes to final concentrations from 1 to 1000 nM and incubated for 2 h at 30 °C. The values of fluorescence anisotropy were obtained using the VICTOR X5 Multilabel Plate Reader (PerkinElmer, Waltham, MA, USA). Filters of 485 and 535 nm were used for excitation and emission, respectively. To calculate the apparent dissociation constants, the assumption that the competitive equilibrium binding of two ligands occurs at a single binding site was used [61]. For each compound, at least 2 replicates were performed. Values of apparent dissociation constants are given as means with 95% confidence intervals. The results were visualized using the GraphPad Prism software (version 8.0.1).

### 4.8. Toe-Printing Analysis

The toe-printing (primer extension inhibition) analysis of azithromycin derivatives was carried out essentially as previously described [62] with some minor modifications, as indicated below. The DNA template ErmCL was generated by PCR using two overlapping primers, ErmCL-fwd (5′-ACTAATACGACTCACTATAGGGAGTTTTATAAGGAGGAAAAAATATGGGCATTTTTAGTATTTTTGTAATCAGCACAGTTCATTATCAA-3′) and ErmCL-rev (5′-GGTTATAATGAATTTTGCTTATTAACGATAGAATTCTATCACTTTTTTTATTATTATTATTTTTTGTTTGGTTGATAATGAACTGTGCT-3′). Before the addition of the DNA template to reaction mixtures at the in vitro translation step, tubes were pre-incubated at RT for 5 min. Moreover, phenol-chloroform extraction of reverse transcription products was replaced with DNA purification using the QIAquick^®^ PCR Purification Kit (Qiagen, Hilden, Germany) according to the manufacturer’s protocol.

## 5. Conclusions

Two series of azithromycin derivatives conjugated with either chloramphenicol (**4a**–**g**) or metronidazole (**5a**–**g**) hemisuccinates were synthesized in this study as potential antimicrobial compounds. Hybrids containing chloramphenicol turned out to be less active than their metronidazole counterparts on a set of bacterial strains. Azithromycin, upon conjugation with metronidazole hemisuccinate via the ethylenediamine linker (**5a**), was characterized by a synergetic effect against anaerobic bacteria *Clostridium sporogenes* ATCC 19404. In addition, the hybrids turned out to be active against the *Escherichia coli* strain inducibly resistant to macrolide antibiotics due to the *ermCL*-dependent regulation of ErmC methyltransferase synthesis. Translation and competition-binding assays revealed that almost all new conjugates efficiently inhibit protein synthesis, and their activity in vitro correlates well with their affinity for the *E. coli* ribosome. Shorter linear linkers containing 3 or 4 carbon atoms between azithromycin and metronidazole hemisuccinate were preferable for better activity in experiments utilizing ribosomes derived from *E. coli*. On the other hand, the introduction of two oxygen atoms into the longer linear linker resulted in a substantial improvement in antibiotic potency, especially toward Gram-positive strains. Unexpectedly, the vast majority of hybrids demonstrated a slightly different mechanism of action in contrast to parental antibiotics. None of them showed ribosome stalling patterns similar to those for chloramphenicol. Instead, they caused premature ribosome stalling at the 3rd codon of the short *ermCL* mRNA and demonstrated a complete absence of ribosome arrest at positions characteristic of azithromycin (and crucial for the regulation or ErmC synthesis). These data imply that substituents at the 4″-position are directed toward the ribosomal peptidyl transferase center (PTC) and thus nonselectively interfere with the synthesis of longer polypeptides by pausing the ribosome during translation. Overall, the synthesized hybrids have a number of valuable features that can be considered in the future for the development of new therapeutic antibacterial agents based on azithromycin. Nevertheless, these compounds might already be active against certain pathogenic bacterial strains inducibly resistant to the macrolide antibiotics commonly used in clinics.

## Figures and Tables

**Table 1:** Structures and yields of hybrid antibacterials **4a**−**g** and **5a**−**g**.

AZT–CLM derivatives							
Cmpd	**4a**	**4b**	**4c**	**4d**	**4e**	**4f**	**4g**
R	H	H	H	H	H	H	(CH_2_)_2_OH
X	(CH_2_)_2_	(CH_2_)_3_	(CH_2_)_4_	(CH_2_)_5_	(CH_2_)_7_	((CH_2_)_2_O)_2_(CH_2_)_2_	(CH_2_)_2_
Yield, %	20	21	24	25	25	22	19
AZT–MZN derivatives							
Cmpd	**5a**	**5b**	**5c**	**5d**	**5e**	**5f**	**5g**
R	H	H	H	H	H	H	(CH_2_)_2_OH
X	(CH_2_)_2_	(CH_2_)_3_	(CH_2_)_4_	(CH_2_)_5_	(CH_2_)_7_	((CH_2_)_2_O)_2_(CH_2_)_2_	(CH_2_)_2_
Yield, %	24	26	25	27	26	23	21

**Table 2:** Antibacterial activity (MIC, μg/mL) of azithromycin derivatives **4a**–**g** conjugated with chloramphenicol and control compound **6**.

Strain	CLM	AZT	4a	4b	4c	4d	4e	4f	4g	6
* S. pneumoniae * ATCC 49619	4	0.06	0.5	0.25	0.25	0.25	0.5	0.125	0.5	>32
* S. aureus * ATCC 29213	8	0.5	32	32	32	16	32	16	>32	32
* E. coli * ATCC 25922	8	2	32	>32	>32	>32	>32	>32	>32	>32

**Table 3:** Antibacterial activity (MIC, μg/mL) of azithromycin derivatives **5a**–**g** conjugated with metronidazole.

Strain	MNZ	AZT	5a	5b	5c	5d	5e	5f	5g
* S. pneumoniae * ATCC 49619	>32	0.06	0.25	0.125	0.125	0.125	0.125	0.06	0.25
* S. agalactiae * 1Cp	>32	0.015	1–2	1	1–2	1	0.5	0.5	1
* S. aureus * ATCC 29213	>32	0.5	8–16	8	16	4	8	4	32
* C. sporogenes * ATCC 19404	8	4	0.25	4	8	8	4	4	16
* P. acnes * 55	>32	<0.06	4	4	2	2	0.25	8	4

**Table 4:** Antibacterial activity (MIC, μg/mL) of azithromycin derivatives **4a**–**g**, **5a**–**g**, and **6** against macrolide-resistant *E. coli* strains.

Strain	MNZ	CLM	AZT	ERY	4a	4b	4c	4d	4e	4f	4g	5a	5b	5c	5d	5e	5f	5g	6
*E. coli* Δ*tolC* pErmC	>200	1.6	>200	>200	>200	>200	>200	>200	>200	>200	>200	>200	>200	>200	>200	>200	>200	>200	>200
* E. coli * Δ*tolC* pErmCL-ErmC	>200	1.6	3.1	50	12.5	25	12.5	25	>200	25	100	12.5	6.3	12.5	12.5	12.5	12.5	25	6.3
* E. coli * Δ*tolC*	>200	1.6	0.8	3.1	12.5	12.5	12.5	12.5	>200	12.5	50	12.5	6.3	6.3	6.3	6.3	12.5	25	3.1
* E. coli * Δ*tolC* pERMZα	>200	1.6	0.8	1.6	12.5	12.5	12.5	12.5	>200	12.5	50	12.5	6.3	6.3	6.3	12.5	12.5	25	3.1
Ratio *	-	1:1	1:4	1:16	1:1	1:2	1:1	1:2	-	1:2	1:2	1:1	1:1	1:2	1:2	1:2	1:1	1:1	1:2

* The ratio between MIC values for *E. coli* Δ*tolC* and *E. coli* Δ*tolC* pErmCL-ErmC strains.

## Data Availability

Data are contained within the article and in the Supplementary Materials.

## Supplementary Materials

The following supporting information can be downloaded at https://www.mdpi.com/article/10.3390/ph17020187/s1. Figure S1: Numbering of atoms of AZT hybrids **4a**–**g** and **5a**–**g**; Figure S2: Competition-binding assay; Figure S3: Correlation analysis of affinity for the 70S *E. coli* ribosome and translation inhibitory activity of azithromycin derivatives; Figures S4–S18: HRMS-ESI spectra for compounds **4a**–**g**, **5a**–**g**, and **6**; Figures S19–S66: NMR spectra for hybrids **4a**–**g** and **5a**–**g**; **Figures S67–S68**: NMR spectra for compound **6**; Figure S69: HPLC analysis of compounds **4c**, **5a**, **5c** stability in PBS buffer during incubation at 37 °C; Figure S70: Kinetic stability of compounds **4c**, **5a**, and **5c** in PBS buffer represented as kinetic curves; Figures S71–S73: HRMS-ESI spectra of **4c**, **5a**, and **5c** metabolites corresponding to 2′-*O*-deacetyl derivatives; Table S1: Assignment of signals of ^1^H and ^13^C NMR spectra for AZT–CLM hybrids **4a**–**g**; Table S2: Assignment of signals of ^1^H and ^13^C NMR spectra for AZT–MNZ hybrids **5a**–**g**; Table S3: Antibacterial activity of AZT–MNZ conjugates **5a**, **5c**, and their 2′-*O*-deacetyl metabolites; Supplementary Methods: Analysis of Kinetic Stability.
