# Supplementary material for: Hybrid Molecules of Azithromycin with Chloramphenicol and Metronidazole: Synthesis and Study of Antibacterial Properties
Source: Pharmaceuticals (Basel). 2024 Jan 31;17(2):187. doi: 10.3390/ph17020187 (PMC10892836; doi:10.3390/ph17020187)
Supplement: Supplementary file 1 [file pharmaceuticals-17-00187-s001.zip › pharmaceuticals-2807946-supplementary.pdf]

*SUPPORTING INFORMATION*

# Hybrid Molecules of Azithromycin with Chloramphenicol and Metronidazole: Synthesis and Study of Antibacterial Properties

**Inna A. Volynkina [1,*,†], Elena N. Bychkova [2,†], Anastasiia O. Karakchieva [1], Alexander S. Tikhomirov [2], George V. Zatonsky [2], Svetlana E. Solovieva [2], Maksim M. Martynov [2], Natalia E. Grammatikova [2], Andrey G. Tereshchenkov [1,3], Alena Paleskava [4,5], Andrey L. Konevega [4,5,6], Petr V. Sergiev [1,3,7], Olga A. Dontsova [1,3,8], Ilya A. Osterman [1], Andrey E. Shchekotikhin [2] and Anna N. Tevyashova [2,9,*]**

1 Department of Chemistry, Lomonosov Moscow State University, Leninskie Gory 1, 119234 Moscow, Russia
2 Gause Institute of New Antibiotics, B. Pirogovskaya 11, 119021 Moscow, Russia
3 Belozersky Institute of Physico-Chemical Biology, Lomonosov Moscow State University, Leninskie Gory 1, 119234 Moscow, Russia
4 Department of Molecular and Radiation Biophysics, Petersburg Nuclear Physics Institute named by B.P. Konstantiniv of, NRC "Kurchatov Institute", mkr. Orlova Roshcha 1, 188300 Gatchina, Russia
5 Institute of Biomedical Systems and Biotechnologies, Peter the Great St. Petersburg Polytechnic University, Khlopina 11, 195251 Saint Petersburg, Russia
6 NBICS Center, NRC "Kurchatov Institute", Kurchatov Square 1, 123182 Moscow, Russia
7 Institute of Functional Genomics, Lomonosov Moscow State University, Leninskie Gory 1, 119234 Moscow, Russia
8 Department of Functioning of Living Systems, Shemyakin-Ovchinnikov Institute of Bioorganic Chemistry, Miklukho-Maklaya 16/10, 117997 Moscow, Russia
9 School of Science, Constructor University, Campus Ring 1, 28759 Bremen, Germany
* Correspondence: inna-volynkina@yandex.ru (I.A.V.); chulis@mail.ru (A.N.T.)
† These authors contributed equally to this work.

## Table of Content

**Figure S1.** Numbering of atoms of AZT hybrids **4a**–**g** and **5a–g**..........5

**Table S1.** Assignment of signals of $^{1}$H and $^{13}$C NMR spectra for AZT–CLM hybrids **4a–g**..........6

**Table S2.** Assignment of signals of $^{1}$H and $^{13}$C NMR spectra for AZT–MNZ hybrids **5a–g**. .... **Error! Bookmark not defined.**

**Figure S2.** Competition-binding assay. ..........10

**Figure S3.** Correlation analysis of affinity for the 70S *E. coli* ribosome and translation inhibitory activity of azithromycin derivatives. ..........11

**Figure S4.** HRMS-ESI of **4a**..........12

**Figure S5.** HRMS-ESI of **4b**. ..........13

**Figure S6.** HRMS-ESI of **4c**..........14

**Figure S7.** HRMS-ESI of **4d**. ..........15

**Figure S8.** HRMS-ESI of **4e**..........16

**Figure S9.** HRMS-ESI of **4f**. ..........17

**Figure S10.** HRMS-ESI of **4g**. ..........18

**Figure S11.** HRMS-ESI of **5a**..........19

**Figure S12.** HRMS-ESI of **5b**. ..........20

**Figure S13.** HRMS-ESI of **5c**..........21

**Figure S14.** HRMS-ESI of **5d**. ..........22

**Figure S15.** HRMS-ESI of **5e**..........23

**Figure S16.** HRMS-ESI of **5f**. ..........24

**Figure S17.** HRMS-ESI of **5g**. ..........25

**Figure S18.** HRMS-ESI of **6**..........26

**Figure S19.** $^{1}$H NMR spectra of **4a** (500 MHz, DMSO-$d_6$). ..........27

**Figure S20.** $^{13}$C NMR spectra of **4a** (125 MHz, DMSO-$d_6$)..........28

**Figure S21.** $^{1}$H NMR spectra of **4b** (500 MHz, DMSO-$d_6$). ..........29

**Figure S22.** $^{13}$C NMR spectra of **4b** (125 MHz, DMSO-$d_6$). ..........30

**Figure S23.** $^{1}$H NMR spectra of **4c** (500 MHz, DMSO-$d_6$)..........31

**Figure S24.** $^{13}$C NMR spectra of **4c** (125 MHz, DMSO-$d_6$). ..........32

**Figure S25.** $^{1}$H-$^{1}$H COSY NMR spectrum of **4c** (DMSO-$d_6$). ..........33

**Figure S26.** $^{1}$H-$^{13}$C HSQC NMR spectrum of **4c** (DMSO-$d_6$). ..........34

**Figure S27.** $^{1}H$ NMR spectra of **4d** (500 MHz, DMSO-$d_6$). ....35
**Figure S28.** $^{13}C$ NMR spectra of **4d** (125 MHz, DMSO-$d_6$). ....36
**Figure S29.** $^{1}H$ NMR spectra of **4e** (500 MHz, DMSO-$d_6$). ....37
**Figure S30.** $^{13}C$ NMR spectra of **4e** (125 MHz, DMSO-$d_6$). ....38
**Figure S31.** $^{1}H$-$^{1}H$ COSY NMR spectrum of **4e** (DMSO-$d_6$). ....39
**Figure S32.** $^{1}H$-$^{13}C$ HSQC NMR spectrum of **4e** (DMSO-$d_6$). ....40
**Figure S33.** $^{1}H$ NMR spectra of **4f** (500 MHz, DMSO-$d_6$). ....41
**Figure S34.** $^{13}C$ NMR spectra of **4f** (125 MHz, DMSO-$d_6$). ....42
**Figure S35.** $^{1}H$-$^{1}H$ COSY NMR spectrum of **4f** (DMSO-$d_6$). ....43
**Figure S36.** $^{1}H$-$^{13}C$ HSQC NMR spectrum of **4f** (DMSO-$d_6$). ....44
**Figure S37.** $^{1}H$ NMR spectra of **4g** (500 MHz, DMSO-$d_6$). ....45
**Figure S38.** $^{13}C$ NMR spectra of **4g** (125 MHz, DMSO-$d_6$). ....46
**Figure S39.** $^{1}H$-$^{1}H$ COSY NMR spectrum of **4g** (DMSO-$d_6$). ....47
**Figure S40.** $^{1}H$-$^{13}C$ HSQC NMR spectrum of **4g** (DMSO-$d_6$). ....48
**Figure S41.** $^{1}H$ NMR spectra of **5a** (500 MHz, DMSO-$d_6$). ....49
**Figure S42.** $^{13}C$ NMR spectra of **5a** (125 MHz, DMSO-$d_6$). ....50
**Figure S43.** $^{1}H$ NMR spectra of **5b** (500 MHz, DMSO-$d_6$). ....51
**Figure S44.** $^{13}C$ NMR spectra of **5b** (125 MHz, DMSO-$d_6$). ....52
**Figure S45.** $^{1}H$-$^{1}H$ COSY NMR spectrum of **5b** (DMSO-$d_6$). ....53
**Figure S46.** $^{1}H$-$^{13}C$ HSQC NMR spectrum of **5b** (DMSO-$d_6$). ....54
**Figure S47.** $^{1}H$ NMR spectra of **5c** (500 MHz, DMSO-$d_6$). ....55
**Figure S48.** $^{13}C$ NMR spectra of **5c** (125 MHz, DMSO-$d_6$). ....56
**Figure S49.** $^{1}H$-$^{1}H$ COSY NMR spectrum of **5c** (DMSO-$d_6$). ....57
**Figure S50.** $^{1}H$-$^{13}C$ HSQC NMR spectrum of **5c** (DMSO-$d_6$). ....58
**Figure S51.** $^{1}H$ NMR spectra of **5d** (500 MHz, DMSO-$d_6$). ....59
**Figure S52.** $^{13}C$ NMR spectra of **5d** (125 MHz, DMSO-$d_6$). ....60
**Figure S53.** $^{1}H$-$^{1}H$ COSY NMR spectrum of **5d** (DMSO-$d_6$). ....61
**Figure S54.** $^{1}H$-$^{13}C$ HSQC NMR spectrum of **5d** (DMSO-$d_6$). ....62
**Figure S55.** $^{1}H$ NMR spectra of **5e** (500 MHz, DMSO-$d_6$). ....63
**Figure S56.** $^{13}C$ NMR spectra of **5e** (125 MHz, DMSO-$d_6$). ....64
**Figure S57.** $^{1}H$-$^{1}H$ COSY NMR spectrum of **5e** (DMSO-$d_6$). ....65

**Figure S58.** $^1$H-$^{13}$C HSQC NMR spectrum of **5e** (DMSO-$d_6$)....66

**Figure S59.** $^1$H NMR spectra of **5f** (500 MHz, DMSO-$d_6$)....67

**Figure S60.** $^{13}$C NMR spectra of **5f** (125 MHz, DMSO-$d_6$)....68

**Figure S61.** $^1$H-$^1$H COSY NMR spectrum of **5f** (DMSO-$d_6$)....69

**Figure S62.** $^1$H-$^{13}$C HSQC NMR spectrum of **5f** (DMSO-$d_6$)....70

**Figure S63.** $^1$H NMR spectra of **5g** (500 MHz, DMSO-$d_6$)....71

**Figure S64.** $^{13}$C NMR spectra of **5g** (125 MHz, DMSO-$d_6$)....72

**Figure S65.** $^1$H-$^1$H COSY NMR spectrum of **5g** (DMSO-$d_6$)....73

**Figure S66.** $^1$H-$^{13}$C HSQC NMR spectrum of **5g** (DMSO-$d_6$)....74

**Figure S67.** $^1$H NMR spectra of **6** (400 MHz, DMSO-$d_6$)....75

**Figure S68.** $^{13}$C NMR spectra of **6** (100 MHz, DMSO-$d_6$)....76

**Figure S69.** HPLC analysis of compounds **4c**, **5a**, **5c** stability in PBS buffer during incubation at 37 °C....77

**Figure S70.** Kinetic stability of compounds **4c**, **5a** and **5c** in PBS buffer represented as kinetic curves....78

**Figure S71.** HRMS-ESI of the **4c** metabolite corresponding to 2′-*O*-deacetyl-**4c**....79

**Figure S72.** HRMS-ESI of the **5a** metabolite corresponding to 2′-*O*-deacetyl-**5a**....80

**Figure S73.** HRMS-ESI of the **5c** metabolite corresponding to 2′-*O*-deacetyl-**5c**....81

**Table S3.** Antibacterial activity of AZT–MNZ conjugates **5a**, **5c** and their 2′-*O*-deacetyl metabolites....82

**Supplementary Methods**....83

**Supplementary References**....84

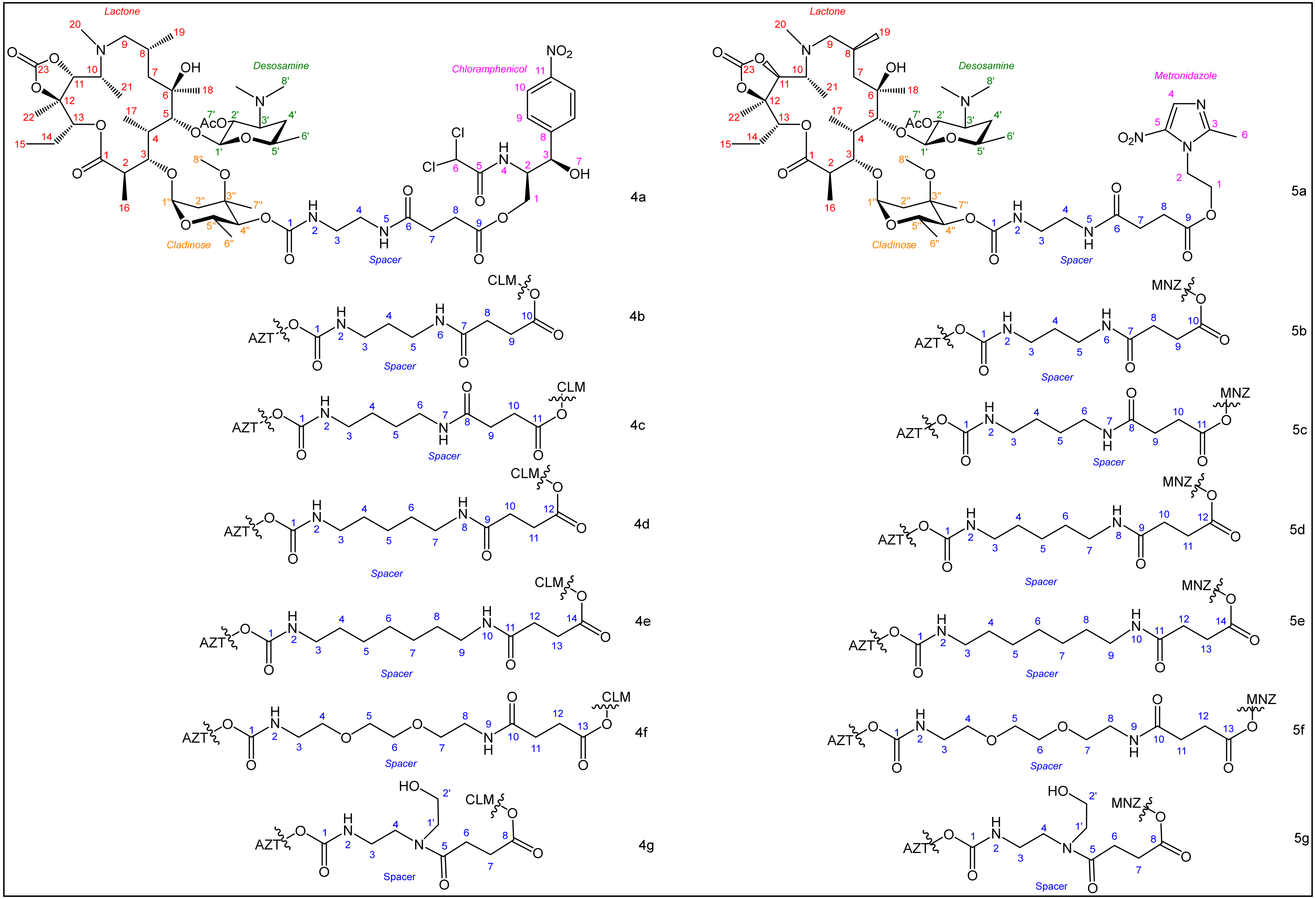


**Figure S1.** Numbering of atoms of AZT hybrids **4a–g** and **5a–g**.

**Table S1.** Assignment of signals of $^{1}H$ and $^{13}C$ NMR spectra for AZT-CLM hybrids **4a–g**.

| Compound 4a | | | Compound 4b | | | Compound 4c | | | Compound 4d | | | Compound 4e | | | Compound 4f | | | Compound 4g | | |
|---|---|---|---|---|---|---|---|---|---|---|---|---|---|---|---|---|---|---|---|---|
| position | δC | δH (δH') | position | δC | δH (δH') | position | δC | δH (δH') | position | δC | δH (δH') | position | δC | δH (δH') | position | δC | δH (δH') | position | δC | δH (δH') |
| Lactone | | | Lactone | | | Lactone | | | Lactone | | | Lactone | | | Lactone | | | Lactone | | |
| 1 C=O | 177,2 | - | 1 C=O | 177,2 | - | 1 C=O | 177,1 | - | 1 C=O | 177,2 | - | 1 C=O | 177,2 | - | 1 C=O | 177,1 | - | 1 C=O | 177,2 | - |
| 2 CH | 44,1 | 2,78 | 2 CH | 44,1 | 2,79 | 2 CH | 44,1 | 2,79 | 2 CH | 44,1 | 2,79 | 2 CH | 44,1 | 2,78 | 2 CH | 44,1 | 2,79 | 2 CH | 44,1 | 2,79 |
| 3 CH | 77,0 | 4,18 | 3 CH | 77,0 | 4,18 | 3 CH | 77,0 | 4,19 | 3 CH | 77,1 | 4,18 | 3 CH | 77,1 | 4,18 | 3 CH | 77,0 | 4,18 | 3 CH | 77,0 | 4,19 |
| 4 CH | 40,6 | 1,90 | 4 CH | 40,7 | 1,90 | 4 CH | 40,7 | 1,90 | 4 CH | 40,7 | 1,89 | 4 CH | 40,7 | 1,90 | 4 CH | 40,7 | 1,90 | 4 CH | 40,7 | 1,91 |
| 5 CH | 82,6 | 3,42 | 5 CH | 82,6 | 3,43 | 5 CH | 82,6 | 3,43 | 5 CH | 82,6 | 3,42 | 5 CH | 82,6 | 3,43 | 5 CH | 82,6 | 3,43 | 5 CH | 82,6 | 3,44 |
| 6 C-OH | 72,7 | 6,76 | 6 C-OH | 72,8 | 6,73 | 6 C-OH | 72,8 | 6,72 | 6 C-OH | 72,8 | 6,73 | 6 C-OH | 72,8 | 6,74 | 6 C-OH | 72,6 | 6,74 | 6 C-OH | 72,8 | 6,75 |
| 7 $CH_2$ | 40,8 | 1,35 | 7 $CH_2$ | 40,9 | 1,35 | 7 $CH_2$ | 40,9 | 1,36 | 7 $CH_2$ | 40,8 | 1,35 | 7 $CH_2$ | 40,8 | 1,35 | 7 $CH_2$ | 40,8 | 1,35 | 7 $CH_2$ | 40,8 | 1,36 |
| 8 CH | 25,3 | 1,84 | 8 CH | 25,3 | 1,84 | 8 CH | 25,3 | 1,84 | 8 CH | 25,3 | 1,84 | 8 CH | 25,3 | 1,84 | 8 CH | 25,3 | 1,84 | 8 CH | 25,3 | 1,85 |
| 9 $CH_2$ | 66,3 | 2,32 (2,21) | 9 $CH_2$ | 66,2 | 2,32 (2,21) | 9 $CH_2$ | 66,3 | 2,32 (2,21) | 9 $CH_2$ | 66,2 | 2,32(2,22) | 9 $CH_2$ | 66,3 | 2,32 (2,21) | 9 $CH_2$ | 66,2 | 2,32(2,22) | 9 $CH_2$ | 66,3 | 2,33(2,22) |
| 10 CH | 59,8 | 3,09 | 10 CH | 59,7 | 3,10 | 10 CH | 59,7 | 3,10 | 10 CH | 59,7 | 3,10 | 10 CH | 59,7 | 3,10 | 10 CH | 59,7 | 3,10 | 10 CH | 59,7 | 3,11 |
| 11 CH | 85,7 | 4,23 | 11 CH | 85,9 | 4,22 | 11 CH | 85,9 | 4,23 | 11 CH | 85,9 | 4,23 | 11 CH | 85,9 | 4,23 | 11 CH | 85,9 | 4,23 | 11 CH | 85,9 | 4,23 |
| 12 C | 85,9 | - | 12 C | 85,0 | - | 12 C | 85,8 | - | 12 C | 85,0 | - | 12 C | 85,0 | - | 12 C | 85,0 | - | 12 C | 85,0 | - |
| 13 CH | 75,5 | 4,78 | 13 CH | 75,5 | 4,78 | 13 CH | 75,5 | 4,79 | 13 CH | 75,5 | 4,78 | 13 CH | 75,5 | 4,78 | 13 CH | 75,5 | 4,79 | 13 CH | 75,5 | 4,79 |
| 14 $CH_2$ | 21,4 | 1,73 (1,58) | 14 $CH_2$ | 21,4 | 1,72 (1,58) | 14 $CH_2$ | 21,4 | 1,72 (1,58) | 14 $CH_2$ | 21,4 | 1,71 (1,58) | 14 $CH_2$ | 21,4 | 1,72 (1,58) | 14 $CH_2$ | 21,4 | 1,72 (1,58) | 14 $CH_2$ | 21,4 | 1,73(1,59) |
| 15 $CH_3$ | 10,2 | 0,84 | 15 $CH_3$ | 10,1 | 0,84 | 15 $CH_3$ | 10,1 | 0,84 | 15 $CH_3$ | 10,2 | 0,84 | 15 $CH_3$ | 10,2 | 0,84 | 15 $CH_3$ | 10,1 | 0,84 | 15 $CH_3$ | 10,2 | 0,84 |
| 16 $CH_3$ | 14,6 | 1,13 | 16 $CH_3$ | 14,6 | 1,13 | 16 $CH_3$ | 14,6 | 1,13 | 16 $CH_3$ | 14,7 | 1,13 | 16 $CH_3$ | 14,7 | 1,13 | 16 $CH_3$ | 14,6 | 1,13 | 16 $CH_3$ | 14,6 | 1,14 |
| 17 $CH_3$ | 9,1 | 0,82 | 17 $CH_3$ | 9,0 | 0,83 | 17 $CH_3$ | 9,0 | 0,83 | 17 $CH_3$ | 9,0 | 0,83 | 17 $CH_3$ | 9,0 | 0,83 | 17 $CH_3$ | 9,0 | 0,83 | 17 $CH_3$ | 9,0 | 0,83 |
| 18 $CH_3$ | 26,9 | 1,14 | 18 $CH_3$ | 26,9 | 1,14 | 18 $CH_3$ | 26,9 | 1,14 | 18 $CH_3$ | 26,9 | 1,14 | 18 $CH_3$ | 26,9 | 1,15 | 18 $CH_3$ | 26,9 | 1,15 | 18 $CH_3$ | 26,9 | 1,14 |
| 19 $CH_3$ | 22,1 | 0,84 | 19 $CH_3$ | 22,0 | 0,85 | 19 $CH_3$ | 22,0 | 0,85 | 19 $CH_3$ | 22,1 | 0,84 | 19 $CH_3$ | 22,1 | 0,85 | 19 $CH_3$ | 22,0 | 0,85 | 19 $CH_3$ | 22,0 | 0,85 |
| 20 N-$CH_3$ | 34,1 | 2,08 | 20 N-$CH_3$ | 34,1 | 2,08 | 20 N-$CH_3$ | 34,1 | 2,08 | 20 N-$CH_3$ | 34,1 | 2,08 | 20 N-$CH_3$ | 34,1 | 2,08 | 20 N-$CH_3$ | 34,1 | 2,08 | 20 N-$CH_3$ | 34,1 | 2,08 |
| 21 $CH_3$ | 5,1 | 0,95 | 21 $CH_3$ | 5,1 | 0,95 | 21 $CH_3$ | 5,1 | 0,95 | 21 $CH_3$ | 5,1 | 0,95 | 21 $CH_3$ | 5,1 | 0,95 | 21 $CH_3$ | 5,1 | 0,95 | 21 $CH_3$ | 5,1 | 0,95 |
| 22 $CH_3$ | 13,3 | 1,44 | 22 $CH_3$ | 13,3 | 1,44 | 22 $CH_3$ | 13,3 | 1,44 | 22 $CH_3$ | 13,3 | 1,44 | 22 $CH_3$ | 13,3 | 1,44 | 22 $CH_3$ | 13,3 | 1,44 | 22 $CH_3$ | 13,3 | 1,44 |
| 23 C | 152,7 | - | 23 C | 152,7 | - | 23 C | 152,6 | - | 23 C | 152,7 | - | 23 C | 152,7 | - | 23 C | 152,7 | - | 23 C | 152,7 | - |
| Desosamine | | | Desosamine | | | Desosamine | | | Desosamine | | | Desosamine | | | Desosamine | | | Desosamine | | |
| 1' CH | 99,6 | 4,54 | 1' CH | 99,7 | 4,55 | 1' CH | 99,6 | 4,54 | 1' CH | 99,7 | 4,54 | 1' CH | 99,7 | 4,54 | 1' CH | 99,7 | 4,55 | 1' CH | 99,5 | 4,54 |
| 2' CH | 71,2 | 4,56 | 2' CH | 71,2 | 4,56 | 2' CH | 71,3 | 4,54 | 2' CH | 71,3 | 4,55 | 2' CH | 71,3 | 4,54 | 2' CH | 71,4 | 4,55 | 2' CH | 71,0 | 4,59 |
| 3' CH | 62,6 | 2,61 | 3' CH | 62,7 | 2,66 | 3' CH | 62,7 | 2,64 | 3' CH | 62,7 | 2,63 | 3' CH | 62,7 | 2,65 | 3' CH | 62,7 | 2,63 | 3' CH | 62,7 | 2,66 |
| 4' $CH_2$ | 29,9 | 1,86(1,20) | 4' $CH_2$ | 30,0 | 1,85(1,20) | 4' $CH_2$ | 30,0 | 1,84(1,19) | 4' $CH_2$ | 30,0 | 1,83(1,18) | 4' $CH_2$ | 30,0 | 1,84(1,18) | 4' $CH_2$ | 30,1 | 1,83(1,18) | 4' $CH_2$ | 29,9 | 1,87(1,26) |
| 5' CH | 67,2 | 3,64 | 5' CH | 67,3 | 3,65 | 5' CH | 67,3 | 3,64 | 5' CH | 67,3 | 3,64 | 5' CH | 67,2 | 3,64 | 5' CH | 67,3 | 3,64 | 5' CH | 67,2 | 3,64 |
| 6' $CH_3$ | 21,0 | 1,12 | 6' $CH_3$ | 21,0 | 1,12 | 6' $CH_3$ | 20,9 | 1,11 | 6' $CH_3$ | 21,0 | 1,11 | 6' $CH_3$ | 20,9 | 1,11 | 6' $CH_3$ | 21,0 | 1,11 | 6' $CH_3$ | 20,9 | 1,13 |
| 7' **C**=O | 170,9 | - | 7' **C**=O | 170,6 | - | 7' **C**=O | 170,4 | - | 7' **C**=O | 170,3 | - | 7' **C**=O | 170,4 | - | 7' **C**=O | 170,7 | - | 7' **C**=O | 170,7 | - |
| 7' $CH_3$ | 21,2 | 1,96 | 7' $CH_3$ | 21,1 | 1,95 | 7' $CH_3$ | 21,1 | 1,95 | 7' $CH_3$ | 21,1 | 1,94 | 7' $CH_3$ | 21,1 | 1,95 | 7' $CH_3$ | 21,1 | 1,95 | 7' $CH_3$ | 21,2 | 1,97 |
| 8' $N(CH_3)_2$ | 40,4 | 2,19 | 8' $N(CH_3)_2$ | 40,3 | 2,20 | 8' $N(CH_3)_2$ | 40,4 | 2,19 | 8' $N(CH_3)_2$ | 40,4 | 2,18 | 8' $N(CH_3)_2$ | 40,4 | 2,19 | 8' $N(CH_3)_2$ | 40,4 | 2,18 | 8' $N(CH_3)_2$ | 40,2 | 2,26 |

**Table S1** (continuation). Assignment of signals of $^1$H and $^{13}$C NMR spectra for AZT-CLM hybrids **4a–g**.

| Compound 4a | | | Compound 4b | | | Compound 4c | | | Compound 4d | | | Compound 4e | | | Compound 4f | | | Compound 4g | | |
|---|---|---|---|---|---|---|---|---|---|---|---|---|---|---|---|---|---|---|---|---|
| position | δC | δH (δH') | position | δC | δH (δH') | position | δC | δH (δH') | position | δC | δH (δH') | position | δC | δH (δH') | position | δC | δH (δH') | position | δC | δH (δH') |
| Cladinose | | | Cladinose | | | Cladinose | | | Cladinose | | | Cladinose | | | Cladinose | | | Cladinose | | |
| 1" CH | 94,4 | 4,88 | 1" CH | 94,4 | 4,89 | 1" CH | 94,4 | 4,89 | 1" CH | 94,4 | 4,88 | 1" CH | 94,5 | 4,88 | 1" CH | 94,4 | 4,89 | 1" CH | 94,4 | 4,90 |
| 2" $CH_2$ | 34,5 | 2,32 (1,68) | 2" $CH_2$ | 34,5 | 2,32 (1,68) | 2" $CH_2$ | 34,6 | 2,32 (1,68) | 2" $CH_2$ | 34,5 | 2,32 (1,68) | 2" $CH_2$ | 34,5 | 2,32 (1,68) | 2" $CH_2$ | 34,5 | 2,32 (1,68) | 2" $CH_2$ | 34,5 | 2,32 (1,69) |
| 3" C | 72,8 | - | 3" C | 72,7 | - | 3" C | 72,7 | - | 3" C | 72,7 | - | 3" C | 72,7 | - | 3" C | 72,7 | - | 3" C | 72,7 | - |
| 4" CH | 77,9 | 4,40 | 4" CH | 77,8 | 4,40 | 4" CH | 77,7 | 4,40 | 4" CH | 77,7 | 4,40 | 4" CH | 77,6 | 4,40 | 4" CH | 77,8 | 4,41 | 4" CH | 77,9 | 4,41 |
| 5" CH | 62,8 | 4,23 | 5" CH | 62,8 | 4,25 | 5" CH | 62,8 | 4,25 | 5" CH | 62,8 | 4,23 | 5" CH | 62,9 | 4,23 | 5" CH | 62,7 | 4,25 | 5" CH | 62,7 | 4,25 |
| 6" $CH_3$ | 17,9 | 1,04 | 6" $CH_3$ | 17,8 | 1,04 | 6" $CH_3$ | 17,8 | 1,04 | 6" $CH_3$ | 17,8 | 1,04 | 6" $CH_3$ | 17,8 | 1,04 | 6" $CH_3$ | 17,8 | 1,05 | 6" $CH_3$ | 17,8 | 1,05 |
| 7" $CH_3$ | 20,4 | 1,06 | 7" $CH_3$ | 20,4 | 1,05 | 7" $CH_3$ | 20,4 | 1,05 | 7" $CH_3$ | 20,4 | 1,05 | 7" $CH_3$ | 20,4 | 1,05 | 7" $CH_3$ | 20,4 | 1,05 | 7" $CH_3$ | 20,4 | 1,05 |
| 8" $OCH_3$ | 48,8 | 3,25 | 8" $OCH_3$ | 48,8 | 3,25 | 8" $OCH_3$ | 48,8 | 3,25 | 8" $OCH_3$ | 48,8 | 3,24 | 8" $OCH_3$ | 48,8 | 3,24 | 8" $OCH_3$ | 48,8 | 3,25 | 8" $OCH_3$ | 48,8 | 3,25 |
| Spacer | | | Spacer | | | Spacer | | | Spacer | | | Spacer | | | Spacer | | | Spacer | | |
| 1 C=O | 156,3 | - | 1 C=O | 156,3 | - | 1 C=O | 156,2 | - | 1 C=O | 156,2 | - | 1 C=O | 156,2 | - | 1 C=O | 156,3 | - | 1 C=O | 156,3 | - |
| 2 C=ONH | - | 7,91 | 2 C=ONH | - | 7,86 | 2 C=ONH | - | 6,95 | 2 C=ONH | - | 6,96 | 2 C=ONH | - | 7,84 | 2 C=ONH | - | 6,97 | 2 C=ONH | - | 6,96(7,08) |
| 3 $CH_2$ | 38,7 | 3,11 | 3 $CH_2$ | 38,0 | 3,05(2,99) | 3 $CH_2$ | 39,9 | 3,03(2,96) | 3 $CH_2$ | 40,1 | 3,04(2,94) | 3 $CH_2$ | 40,1 | 3,04(2,93) | 3 $CH_2$ | 40,1 | 3,16(2,99) | 3 $CH_2$ * | 38,4/39,0 | 3,14/3,21 |
| 4 $CH_2$ | 38,7 | 3,10 | 4 $CH_2$ | 29,6 | 1,52 | 4 $CH_2$ | 26,9 | 1,38 | 4 $CH_2$ | 29,1 | 1,39 | 4 $CH_2$ | 29,4 | 1,38 | 4 $CH_2$ | 69,0 | 3,40 | 4 $CH_2$ * | 45,3/47,3 | 3,40/3,41 |
| | | | 5 $CH_2$ | 36,1 | 3,05 | 5 $CH_2$ | 26,3 | 1,37 | 5 $CH_2$ | 28,9 | 1,23 | 5 $CH_2$ | 26,4 | 1,22 | 5 $CH_2$ | 69,4 | 3,48 | 1' $CH_2$ * | 49,8/48,3 | 3,36/3,33 |
| | | | | | | 6 $CH_2$ | 38,2 | 3,02 | 6 $CH_2$ | 28,7 | 1,37 | 6 $CH_2$ | 28,4 | 1,22 | 6 $CH_2$ | 69,4 | 3,48 | 2' $CH_2$ * | 58,8/58,6 | 3,54/3,46 |
| | | | | | | | | | 7 $CH_2$ | 38,5 | 2,99 | 7 $CH_2$ | 26,0 | 1,22 | 7 $CH_2$ | 69,0 | 3,38 | 5 C=O | 170,7/ 171,2 | - |
| | | | | | | | | | | | | 8 $CH_2$ | 29,0 | 1,35 | 8 $CH_2$ | 38,5 | 3,18 | | | |
| | | | | | | | | | | | | 9 $CH_2$ | 38,5 | 3,01 | | | | 6 $CH_2$ | 29,0 | 2,48 |
| 5 C=ONH | - | 6,93 | 6 C=ONH | - | 6,94 | 7 C=ONH | - | 7,85 | 8 C=ONH | - | 7,84 | 10 C=ONH | - | 6,94 | 9 C=ONH | - | 7,96 | 7 $CH_2$ * | 27,6/27,4 | 2,63/2,61 |
| 6 C=O | 170,9 | - | 7 C=O | 170,9 | - | 8 C=O | 170,4 | - | 9 C=O | 170,3 | - | 11 C=O | 170,9 | - | 10 C=O | 170,7 | - | 8 C=O * | 172,2/ 172,3 | - |
| 7 $CH_2$ | 28,9 | 2,47 | 8 $CH_2$ | 29,0 | 2,48 | 9 $CH_2$ | 29,0 | 2,49 | 10 $CH_2$ | 29,0 | 2,47 | 12 $CH_2$ | 29,0 | 2,48 | 11 $CH_2$ | 29,7 | 2,36 | | | |
| 8 $CH_2$ | 29,7 | 2,34 | 9 $CH_2$ | 29,7 | 2,35 | 10 $CH_2$ | 29,7 | 2,34 | 11 $CH_2$ | 29,7 | 2,34 | 13 $CH_2$ | 29,7 | 2,35 | 12 $CH_2$ | 28,9 | 2,48 | * - denotes second signal due to dynamics | | |
| 9 C=O | 172,1 | - | 10 C=O | 172,1 | - | 11 C=O | 172,1 | - | 12 C=O | 172,2 | - | 14 C=O | 172,1 | - | 13 C=O | 172,1 | - | | | |
| Chloramphenicol | | | Chloramphenicol | | | Chloramphenicol | | | Chloramphenicol | | | Chloramphenicol | | | Chloramphenicol | | | Chloramphenicol | | |
| 1 $CH_2$ | 63,0 | 4,21(4,09) | 1 $CH_2$ | 63,0 | 4,22(4,09) | 1 $CH_2$ | 62,9 | 4,21(4,09) | 1 $CH_2$ | 62,9 | 4,21(4,09) | 1 $CH_2$ | 62,9 | 4,20(4,08) | 1 $CH_2$ | 62,9 | 4,21(4,09) | 1 $CH_2$ | 62,9 | 4,21(4,10) |
| 2 CH | 53,5 | 4,22 | 2 CH | 53,5 | 4,23 | 2 CH | 53,5 | 4,23 | 2 CH | 53,5 | 4,22 | 2 CH | 53,5 | 4,22 | 2 CH | 53,5 | 4,22 | 2 CH | 53,5 | 4,23 |
| 3 CH | 69,5 | 5,02 | 3 CH | 69,5 | 5,02 | 3 CH | 69,5 | 5,02 | 3 CH | 69,5 | 5,02 | 3 CH | 69,5 | 5,02 | 3 CH | 69,4 | 5,02 | 3 CH | 69,4 | 5,04 |
| 4 C=ONH | - | 8,51 | 4 C=ONH | - | 8,51 | 4 C=ONH | - | 8,51 | 4 C=ONH | - | 8,52 | 4 C=ONH | - | 8,52 | 4 C=ONH | - | 8,54 | 4 C=ONH | - | 8,51 |
| 5 C=O | 163,6 | - | 5 C=O | 163,6 | - | 5 C=O | 163,6 | - | 5 C=O | 163,6 | - | 5 C=O | 163,6 | - | 5 C=O | 163,6 | - | 5 C=O | 163,6 | - |
| 6 $CHCl_2$ | 66,3 | 6,43 | 6 $CHCl_2$ | 66,3 | 6,43 | 6 $CHCl_2$ | 66,2 | 6,44 | 6 $CHCl_2$ | 66,3 | 6,43 | 6 $CHCl_2$ | 66,3 | 6,43 | 6 $CHCl_2$ | 66,2 | 6,44 | 6 $CHCl_2$ | 66,3 | 6,44 |
| 7 OH | - | 6,22 | 7 OH | - | 6,21 | 7 OH | - | 6,21 | 7 OH | - | 6,22 | 7 OH | - | 6,74 | 7 OH | - | 6,23 | 7 OH | - | 6,21 |
| 8 C | 146,6 | - | 8 C | 146,7 | - | 8 C | 146,6 | - | 8 C | 146,6 | - | 8 C | 146,6 | - | 8 C | 146,6 | - | 8 C | 146,6 | - |
| 9 CH | 127,4 | 7,64 | 9 CH | 127,4 | 7,63 | 9 CH | 127,4 | 7,64 | 9 CH | 127,4 | 7,64 | 9 CH | 127,5 | 7,64 | 9 CH | 127,4 | 7,64 | 9 CH | 127,5 | 7,64 |
| 10 CH | 122,9 | 8,17 | 10 CH | 122,9 | 8,17 | 10 CH | 122,8 | 8,16 | 10 CH | 122,8 | 8,16 | 10 CH | 122,8 | 8,16 | 10 CH | 122,8 | 8,17 | 10 CH | 122,9 | 8,17 |
| 11 C | 150,2 | - | 11 C | 150,3 | - | 11 C | 150,2 | - | 11 C | 150,2 | - | 11 C | 150,2 | - | 11 C | 150,2 | - | 11 C | 150,2 | - |

**Table S2.** Assignment of signals of $^{1}$H and $^{13}$C NMR spectra for AZT–MNZ hybrids **5a–g.**

| Compound 5a | | | Compound 5b | | | Compound 5c | | | Compound 5d | | | Compound 5e | | | Compound 5f | | | Compound 5g | | |
|---|---|---|---|---|---|---|---|---|---|---|---|---|---|---|---|---|---|---|---|---|
| position | δC | δH (δH') | position | δC | δH (δH') | position | δC | δH (δH') | position | δC | δH (δH') | position | δC | δH (δH') | position | δC | δH (δH') | position | δC | δH (δH') |
| Lactone | | | Lactone | | | Lactone | | | Lactone | | | Lactone | | | Lactone | | | Lactone | | |
| 1 C=O | 177,1 | - | 1 C=O | 177,2 | - | 1 C=O | 177,2 | - | 1 C=O | 177,1 | - | 1 C=O | 177,1 | - | 1 C=O | 177,2 | - | 1 C=O | 177,2 | - |
| 2 CH | 44,1 | 2,79 | 2 CH | 44,3 | 2,79 | 2 CH | 44,1 | 2,78 | 2 CH | 44,1 | 2,79 | 2 CH | 44,1 | 2,78 | 2 CH | 44,1 | 2,79 | 2 CH | 44,1 | 2,78 |
| 3 CH | 77,0 | 4,18 | 3 CH | 77,1 | 4,23 | 3 CH | 77,0 | 4,18 | 3 CH | 76,9 | 4,20 | 3 CH | 76,9 | 4,21 | 3 CH | 77,0 | 4,19 | 3 CH | 77,0 | 4,20 |
| 4 CH | 40,6 | 1,91 | 4 CH | 41 | 1,92 | 4 CH | 40,7 | 1,90 | 4 CH | 40,6 | 1,92 | 4 CH | 40,6 | 1,93 | 4 CH | 40,7 | 1,91 | 4 CH | 40,6 | 1,92 |
| 5 CH | 82,5 | 3,43 | 5 CH | 83 | 3,46 | 5 CH | 82,5 | 3,43 | 5 CH | 82,5 | 3,45 | 5 CH | 82,4 | 3,46 | 5 CH | 82,5 | 3,44 | 5 CH | 82,4 | 3,45 |
| 6 C-OH | 72,8 | 6,75 | 6 C-OH | 73 | 6,59 | 6 C-OH | 72,8 | 6,77 | 6 C-OH | 72,7 | 6,79 | 6 C-OH | 72,7 | 6,87 | 6 C-OH | 72,8 | 6,79 | 6 C-OH | 72,7 | 6,86 |
| 7 $CH_2$ | 40,8 | 1,35 | 7 $CH_2$ | 41 | 1,45 | 7 $CH_2$ | 40,8 | 1,35 | 7 $CH_2$ | 40,7 | 1,36 | 7 $CH_2$ | 40,7 | 1,35 | 7 $CH_2$ | 40,7 | 1,35 | 7 $CH_2$ | 40,7 | 1,34 |
| 8 CH | 25,3 | 1,84 | 8 CH | 25,3 | 1,87 | 8 CH | 25,3 | 1,84 | 8 CH | 25,3 | 1,85 | 8 CH | 25,3 | 1,84 | 8 CH | 25,3 | 1,84 | 8 CH | 25,3 | 1,84 |
| 9 $CH_2$ | 66,3 | 2,32(2,21) | 9 $CH_2$ | 66,3 | 2,35(2,21) | 9 $CH_2$ | 66,3 | 2,32(2,21) | 9 $CH_2$ | 66,3 | 2,32(2,22) | 9 $CH_2$ | 66,3 | 2,32(2,22) | 9 $CH_2$ | 66,3 | 2,33(2,22) | 9 $CH_2$ | 66,3 | 2,33(2,21) |
| 10 CH | 59,7 | 3,10 | 10 CH | 59,7 | 3,10 | 10 CH | 59,7 | 3,10 | 10 CH | 59,7 | 3,11 | 10 CH | 59,7 | 3,11 | 10 CH | 59,7 | 3,11 | 10 CH | 59,8 | 3,10 |
| 11 CH | 85,9 | 4,23 | 11 CH | 85,5 | 4,26 | 11 CH | 85,9 | 4,22 | 11 CH | 85,9 | 4,22 | 11 CH | 85,9 | 4,22 | 11 CH | 85,9 | 4,23 | 11 CH | 85,9 | 4,21 |
| 12 C | 85 | - | 12 C | 85,2 | - | 12 C | 85 | - | 12 C | 85,0 | - | 12 C | 85,0 | - | 12 C | 85,0 | - | 12 C | 85,0 | - |
| 13 CH | 75,5 | 4,78 | 13 CH | 75,5 | 4,8 | 13 CH | 75,5 | 4,78 | 13 CH | 75,5 | 4,79 | 13 CH | 75,6 | 4,79 | 13 CH | 75,5 | 4,79 | 13 CH | 75,6 | 4,79 |
| 14 $CH_2$ | 21,4 | 1,72(1,58) | 14 $CH_2$ | 21,4 | 1,75(1,58) | 14 $CH_2$ | 21,4 | 1,72(1,58) | 14 $CH_2$ | 21,4 | 1,73(1,59) | 14 $CH_2$ | 21,4 | 1,73(1,60) | 14 $CH_2$ | 21,4 | 1,72(1,59) | 14 $CH_2$ | 21,4 | 1,72(1,58) |
| 15 $CH_3$ | 10,1 | 0,84 | 15 $CH_3$ | 10,2 | 0,84 | 15 $CH_3$ | 10,1 | 0,83 | 15 $CH_3$ | 10,1 | 0,84 | 15 $CH_3$ | 10,2 | 0,85 | 15 $CH_3$ | 10,2 | 0,84 | 15 $CH_3$ | 10,2 | 0,84 |
| 16 $CH_3$ | 14,6 | 1,13 | 16 $CH_3$ | 14,7 | 1,13 | 16 $CH_3$ | 14,7 | 1,13 | 16 $CH_3$ | 14,6 | 1,14 | 16 $CH_3$ | 14,6 | 1,14 | 16 $CH_3$ | 14,6 | 1,14 | 16 $CH_3$ | 14,6 | 1,14 |
| 17 $CH_3$ | 9,0 | 0,83 | 17 $CH_3$ | 9,1 | 0,97 | 17 $CH_3$ | 9,0 | 0,83 | 17 $CH_3$ | 9,0 | 0,83 | 17 $CH_3$ | 8,9 | 0,83 | 17 $CH_3$ | 9,0 | 0,83 | 17 $CH_3$ | 8,9 | 0,82 |
| 18 $CH_3$ | 26,9 | 1,14 | 18 $CH_3$ | 27,1 | 1,17 | 18 $CH_3$ | 26,9 | 1,14 | 18 $CH_3$ | 26,9 | 1,15 | 18 $CH_3$ | 26,3 | 1,2 | 18 $CH_3$ | 26,9 | 1,14 | 18 $CH_3$ | 26,9 | 1,14 |
| 19 $CH_3$ | 22,0 | 0,84 | 19 $CH_3$ | 22,1 | 0,86 | 19 $CH_3$ | 22,0 | 0,84 | 19 $CH_3$ | 22,0 | 0,85 | 19 $CH_3$ | 22,0 | 0,84 | 19 $CH_3$ | 22,1 | 0,84 | 19 $CH_3$ | 22,0 | 0,84 |
| 20 N-$CH_3$ | 34,1 | 2,08 | 20 N-$CH_3$ | 34,1 | 2,09 | 20 N-$CH_3$ | 34,2 | 2,08 | 20 N-$CH_3$ | 34,1 | 2,08 | 20 N-$CH_3$ | 34,1 | 2,09 | 20 N-$CH_3$ | 34,1 | 2,11 | 20 N-$CH_3$ | 34,1 | 2,08 |
| 21 $CH_3$ | 5,1 | 0,95 | 21 $CH_3$ | 5,4 | 0,97 | 21 $CH_3$ | 5,1 | 0,95 | 21 $CH_3$ | 5,1 | 0,95 | 21 $CH_3$ | 5,1 | 0,96 | 21 $CH_3$ | 5,1 | 0,96 | 21 $CH_3$ | 5,1 | 0,95 |
| 22 $CH_3$ | 13,3 | 1,44 | 22 $CH_3$ | 13,6 | 1,46 | 22 $CH_3$ | 13,3 | 1,44 | 22 $CH_3$ | 13,2 | 1,45 | 22 $CH_3$ | 13,2 | 1,46 | 22 $CH_3$ | 13,3 | 1,45 | 22 $CH_3$ | 13,3 | 1,45 |
| 23 C | 152,6 | - | 23 C | 152,6 | - | 23 C | 152,7 | - | 23 C | 152,6 | - | 23 C | 152,7 | - | 23 C | 152,7 | - | 23 C | 152,7 | - |
| Desosamine | | | Desosamine | | | Desosamine | | | Desosamine | | | Desosamine | | | Desosamine | | | Desosamine | | |
| 1' CH | 99,5 | 4,54 | 1' CH | 101,8 | 4,38 | 1' CH | 99,5 | 4,54 | 1' CH | 99,2 | 4,55 | 1' CH | 98,9 | 4,55 | 1' CH | 99,5 | 4,55 | 1' CH | 98,8 | 4,54 |
| 2' CH | 71,2 | 4,56 | 2' CH | 69,2 | 3,23 | 2' CH | 70,8 | 4,6 | 2' CH | n.o. | n.o. | 2' CH | n.o. | n.o. | 2' CH | n.o. | n.o. | 2' CH | n.o. | n.o. |
| 3' CH | 63 | 2,66 | 3' CH | not four | not found | 3' CH | 62,8 | 2,82 | 3' CH | n.o. | n.o. | 3' CH | n.o. | n.o. | 3' CH | n.o. | 2,67 | 3' CH | n.o. | n.o. |
| 4' $CH_2$ | 30,0 | 1,85(1,20) | 4' $CH_2$ | 29,7 | 1,91(1,34) | 4' $CH_2$ | 29,9 | 1,88(1,28) | 4' $CH_2$ | n.o. | n.o. | 4' $CH_2$ | n.o. | n.o. | 4' $CH_2$ | n.o. | 1,87(1,25) | 4' $CH_2$ | n.o. | n.o. |
| 5' CH | 67,1 | 3,65 | 5' CH | 66,8 | 3,63 | 5' CH | 67,2 | 3,65 | 5' CH | 67 | 3,68 | 5' CH | 66,8 | 3,71 | 5' CH | 67,1 | 3,67 | 5' CH | 66,9 | 3,67 |
| 6' $CH_3$ | 20,9 | 1,13 | 6' $CH_3$ | 21,0 | 1,14 | 6' $CH_3$ | 20,9 | 1,13 | 6' $CH_3$ | 20,8 | 1,16 | 6' $CH_3$ | 20,7 | 1,19 | 6' $CH_3$ | 20,9 | 1,14 | 6' $CH_3$ | 20,8 | 1,16 |
| 7' **C**=O | 170,6 | - | 7' **C**=O | 170,5 | - | 7' **C**=O | 170,1 | - | 7' **C**=O | 170,1 | - | 7' **C**=O | 170,1 | - | 7' **C**=O | 170,5 | - | 7' **C**=O | 170,5 | - |
| 7' $CH_3$ | 21,1 | 1,97 | 7' $CH_3$ | not four | not found | 7' $CH_3$ | 21,2 | 1,98 | 7' $CH_3$ | 21,2 | 2 | 7' $CH_3$ | 21,3 | 2,05 | 7' $CH_3$ | 21,2 | 1,99 | 7' $CH_3$ | 21,4 | 2,08 |
| 8' $N(CH_3)_2$ | 40,2 | 2,22 | 8' $N(CH_3)_2$ | 40,4 | 2,54 | 8' $N(CH_3)_2$ | 40,4 | 2,54 | 8' $N(CH_3)_2$ | 40,4 | 2,54 | 8' $N(CH_3)_2$ | 40,4 | 2,54 | 8' $N(CH_3)_2$ | 40,4 | 2,54 | 8' $N(CH_3)_2$ | 40,4 | 2,54 |

**Table S2** (continuation). Assignment of signals of $^{1}$H and $^{13}$C NMR spectra for AZT–MNZ hybrids **5a–g**.

| Compound 5a | | | Compound 5b | | | Compound 5c | | | Compound 5d | | | Compound 5e | | | Compound 5f | | | Compound 5g | | |
|---|---|---|---|---|---|---|---|---|---|---|---|---|---|---|---|---|---|---|---|---|
| position | δC | δH (δH') | position | δC | δH (δH') | position | δC | δH (δH') | position | δC | δH (δH') | position | δC | δH (δH') | position | δC | δH (δH') | position | δC | δH (δH') |
| **Cladinose** | | | **Cladinose** | | | **Cladinose** | | | **Cladinose** | | | **Cladinose** | | | **Cladinose** | | | **Cladinose** | | |
| 1" CH | 94,4 | 4,89 | 1" CH | 94,5 | 4,91 | 1" CH | 94,4 | 4,89 | 1" CH | 94,4 | 4,9 | 1" CH | 94,4 | 4,91 | 1" CH | 94,4 | 4,89 | 1" CH | 94,4 | 4,90 |
| 2" $CH_2$ | 34,5 | 2,32(1,69) | 2" $CH_2$ | 34,5 | 2,31(1,67) | 2" $CH_2$ | 34,5 | 2,32(1,68) | 2" $CH_2$ | 34,5 | 2,33(1,69) | 2" $CH_2$ | 34,5 | 2,32(1,70) | 2" $CH_2$ | 34,5 | 2,32(1,68) | 2" $CH_2$ | 34,5 | 2,32(1,70) |
| 3" C | 72,7 | - | 3" C | 72,7 | - | 3" C | 72,7 | - | 3" C | 72,7 | - | 3" C | 72,7 | - | 3" C | 72,7 | - | 3" C | 72,7 | - |
| 4" CH | 77,9 | 4,40 | 4" CH | 77,8 | 4,39 | 4" CH | 77,7 | 4,40 | 4" CH | 77,6 | 4,41 | 4" CH | 77,6 | 4,41 | 4" CH | 77,7 | 4,40 | 4" CH | 77,9 | 4,41 |
| 5" CH | 62,7 | 4,22 | 5" CH | 62,7 | 4,24 | 5" CH | 62,8 | 4,22 | 5" CH | 62,8 | 4,22 | 5" CH | 62,8 | 4,2 | 5" CH | 62,8 | 4,22 | 5" CH | 62,8 | 4,21 |
| 6" $CH_3$ | 17,8 | 1,04 | 6" $CH_3$ | 17,8 | 1,04 | 6" $CH_3$ | 17,8 | 1,04 | 6" $CH_3$ | 17,8 | 1,05 | 6" $CH_3$ | 17,9 | 1,06 | 6" $CH_3$ | 17,8 | 1,04 | 6" $CH_3$ | 17,8 | 1,04 |
| 7" $CH_3$ | 20,4 | 1,06 | 7" $CH_3$ | 20,4 | 1,04 | 7" $CH_3$ | 20,4 | 1,05 | 7" $CH_3$ | 20,4 | 1,07 | 7" $CH_3$ | 20,3 | 1,07 | 7" $CH_3$ | 20,4 | 1,05 | 7" $CH_3$ | 20,4 | 1,05 |
| 8" O**$CH_3$** | 48,8 | 3,26 | 8" O**$CH_3$** | 48,9 | 3,22 | 8" O**$CH_3$** | 48,8 | 3,25 | 8" O**$CH_3$** | 48,9 | 3,26 | 8" O**$CH_3$** | 49 | 3,27 | 8" O**$CH_3$** | 48,8 | 3,25 | 8" O**$CH_3$** | 49 | 3,26 |
| **Spacer** | | | **Spacer** | | | **Spacer** | | | **Spacer** | | | **Spacer** | | | **Spacer** | | | **Spacer** | | |
| 1 C=O | 156,3 | - | 1 C=O | 156,1 | - | 1 C=O | 156,2 | - | 1 C=O | 156,2 | - | 1 C=O | 156,2 | - | 1 C=O | 156,3 | - | 1 C=O | 156,2 | - |
| 2 C=ON**H** | - | 6,9 | 2 C=ON**H** | - | 6,84 | 2 C=ON**H** | - | 6,93 | 2 C=ON**H** | - | 6,87 | 2 C=ON**H** | - | 6,83 | 2 C=ON**H** | - | 6,89 | 2 C=ON**H** | - | 6,86 |
| 3 CH2 | 38,6 | 3,11(3,06) | 3 CH2 | 37,9 | 3,03(2,97) | 3 CH2 | 40,0 | 3,03(2,96) | 3 CH2 | 40,1 | 3,05(2,96) | 3 CH2 | 40,1 | 3,06(2,96) | 3 CH2 | 40,2 | 3,17 | 3 CH2 | 45,4 | 3,35(3,27) |
| 4 CH2 | 40,1 | 3,05 | 4 CH2 | 29,6 | 1,49 | 4 CH2 | 26,9 | 1,38 | 4 CH2 | 29,1 | 1,4 | 4 CH2 | 29,4 | 1,4 | 4 CH2 | 69,1 | 3,41 | 4 CH2 | 38,7 | 3,19(3,12) |
| | | | 5 CH2 | 35,9 | 3,02 | 5 CH2 | 26,3 | 1,35 | 5 CH2 | 23,4 | 1,22 | 5 CH2 | 26,3 | 1,21 | 5 CH2 | 69,1 | 3,37 | 1' CH2 | 47,2 | 3,37(3,28) |
| | | | | | | 6 CH2 | 38,2 | 2,99 | 6 CH2 | 28,7 | 1,36 | 6 CH2 | 28,3 | 1,23 | 6 CH2 | 69,5 | 3,49 | 2' CH2 | 58,7 | 3,51(3,43) |
| | | | | | | | | | 7 CH2 | 38,4 | 2,97 | 7 CH2 | 26,0 | 1,23 | 7 CH2 | 69,5 | 3,49 | | | |
| | | | | | | | | | | | | 8 CH2 | 29,0 | 1,34 | 8 CH2 | 38,5 | 3,16 | | | |
| | | | | | | | | | | | | 9 CH2 | 38,4 | 2,98 | | | | | | |
| 5 C=ON**H** | - | 7,87 | 6 C=ON**H** | - | 7,86 | 7 C=ON**H** | - | 7,82 | 8 C=ON**H** | - | 7,79 | 10 C=ON**H** | - | 7,8 | 9 C=ON**H** | - | 7,92 | | | |
| 6 C=O | 170,6 | - | 7 C=O | 170,5 | - | 8 C=O | 170,1 | - | 9 C=O | 170,1 | - | 11 C=O | 170,1 | - | 10 C=O | 170,5 | - | 5 C=O | 171 | - |
| 7 CH2 | 28,7 | 2,43 | 8 CH2 | 28,7 | 2,43 | 9 CH2 | | 2,42 | 10 CH2 | 28,7 | 2,42 | 12 CH2 | 28,8 | 2,42 | 11 CH2 | 28,7 | 2,41 | 6 CH2 | 27,2 | 2,52 |
| 8 CH2 | 29,5 | 2,27 | 9 CH2 | 29,6 | 2,28 | 10 CH2 | 29,5 | 2,26 | 11 CH2 | 29,5 | 2,27 | 13 CH2 | 29,5 | 2,27 | 12 CH2 | 29,5 | 2,29 | 7 CH2 | 27,4 | 2,56 |
| 9 C=O | 171,9 | - | 10 C=O | 172 | - | 11 C=O | 172 | - | 12 C=O | 172 | - | 14 C=O | 172 | - | 13 C=O | 172 | - | 8 C=O | 172,1 | - |
| **Metronidazole** | | | **Metronidazole** | | | **Metronidazole** | | | **Metronidazole** | | | **Metronidazole** | | | **Metronidazole** | | | **Metronidazole** | | |
| 1 CH2 | 62,2 | 4,35 | 1 CH2 | 62,2 | 4,35 | 1 CH2 | 62,2 | 4,35 | 1 CH2 | 62,1 | 4,35 | 1 CH2 | 62,2 | 4,34 | 1 CH2 | 62,4 | 4,35 | 1 CH2 | 62,1 | 4,35 |
| 2 CH2 | 44,7 | 4,56 | 2 CH2 | 44,7 | 4,55 | 2 CH2 | 44,7 | 4,55 | 2 CH2 | 44,7 | 4,56 | 2 CH2 | 44,7 | 4,56 | 2 CH2 | 44,7 | 4,56 | 2 CH2 | 44,7 | 4,56 |
| 3 C | 151,6 | - | 3 C | 151,5 | - | 3 C | 151,5 | - | 3 C | 151,5 | - | 3 C | 151,5 | - | 3 C | 151,6 | - | 3 C | 151,5 | - |
| 4 CH | 133 | 8,03 | 4 CH | 133 | 8,04 | 4 CH | 133 | 8,03 | 4 CH | 133 | 8,03 | 4 CH | 133 | 8,03 | 4 CH | 133 | 8,03 | 4 CH | 133 | 8,04 |
| 5 C | 138,4 | - | 5 C | 138,4 | - | 5 C | 138,4 | - | 5 C | 138,4 | - | 5 C | 138,5 | - | 5 C | 138,5 | - | 5 C | 138,5 | - |
| 6 CH3 | 13,8 | 2,45 | 6 CH3 | 13,8 | 2,45 | 6 CH3 | 13,9 | 2,45 | 6 CH3 | 13,8 | 2,46 | 6 CH3 | 13,9 | 2,45 | 6 CH3 | 13,9 | 2,45 | 6 CH3 | 13,9 | 2,46 |

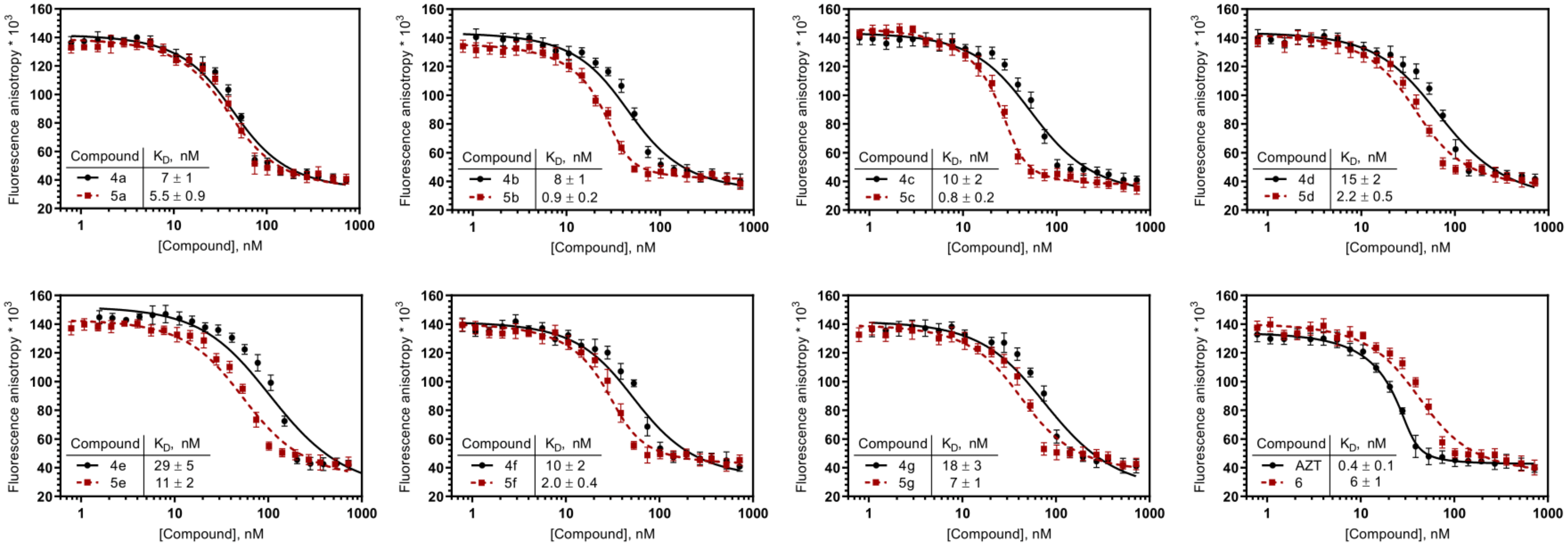


**Figure S2.** Competition-binding assay. The competition-binding assay was applied to test the displacement of fluorescently labeled BODIPY-ERY from the *E. coli* 70S ribosomes in the presence of increasing concentrations of AZT–CLM (**4a–g**) and AZT–MNZ (**5a–g**) derivatives measured by fluorescence anisotropy. AZT and compound **6** were used as controls. The resulting values for apparent dissociation constants ($K_D$) and 95% confidence intervals are shown on each plot. All reactions were repeated at least two times. Error bars represent standard deviation.

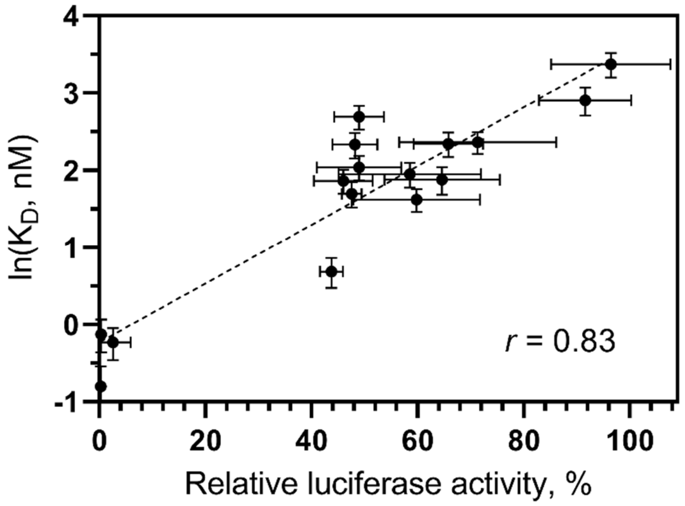


**Figure S3.** Correlation analysis of affinity for the 70S *E. coli* ribosome (shown as the natural logarithm of the apparent dissociation constant) and translation inhibitory activity (shown as the relative residual level of firefly luciferase activity) of azithromycin derivatives. The trend line is shown as a dash. Error bars represent 95% confidence intervals. Spearman correlation coefficient ($r$) is presented on the plot, $p$-value = 0.0001.

# Compound Spectrum List Report

**Analysis Info**

| | | | |
|---|---|---|---|
| Analysis Name | D:\Data\Tevjashova\LCTA-3003_MeOH_pos_tune_wide.d | Acquisition Date | 22.05.2019 10:25:52 |
| Method | tune_wide.m | | |
| Sample Name | | Operator | Korolev |
| Comment | | Instrument / Ser# | micrOTOF-Q II 10225 |

**Acquisition Parameter**

| | | | | | |
|---|---|---|---|---|---|
| Source Type | ESI | Ion Polarity | Positive | Set Nebulizer | 0.4 Bar |
| Focus | Active | Set Capillary | 4500 V | Set Dry Heater | 180 °C |
| Scan Begin | 50 m/z | Set End Plate Offset | -500 V | Set Dry Gas | 4.0 l/min |
| Scan End | 3000 m/z | Set Collision Cell RF | 550.0 Vpp | Set Divert Valve | Source |

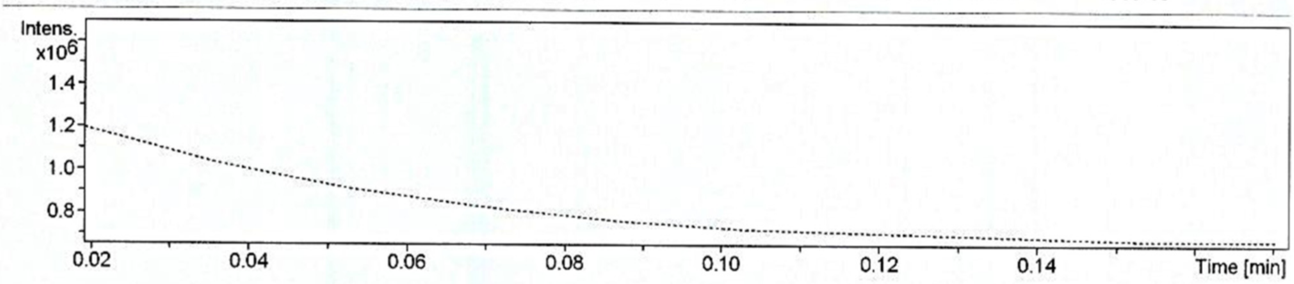


| # | RT [min] | Area | Int. Type | Intens. | S/N | Chromatogram | Max. m/z |
|---|---|---|---|---|---|---|---|
| n.a. | 0.1 | n.a. | Average spectrum | n.a. | n.a. | n.a. | 1307.5711 |

**+MS, 0.0-0.1min #(2-6)**

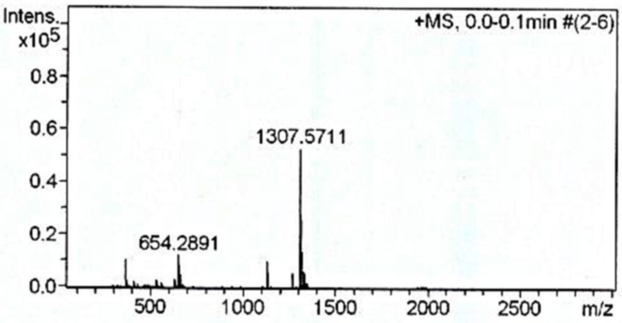


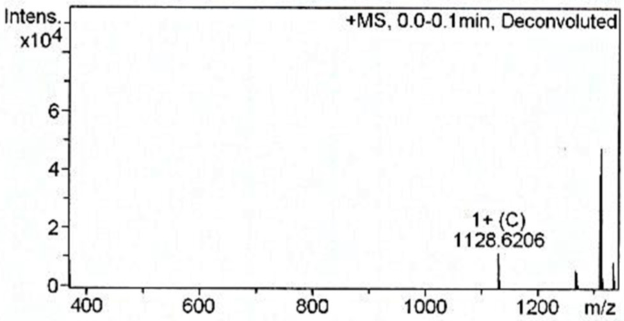


| # | m/z | Res. | S/N | I | I % |
|---|---|---|---|---|---|
| 1 | 369.3853 | 13732 | 579.4 | 10685 | 20.2 |
| 2 | 654.2891 | 20505 | 260.3 | 12231 | 23.2 |
| 3 | 654.7906 | 21878 | 167.0 | 7854 | 14.9 |
| 4 | 655.2889 | 17758 | 189.2 | 8903 | 16.9 |
| 5 | 1128.6183 | 22048 | 204.7 | 10243 | 19.4 |
| 6 | 1307.5711 | 24517 | 813.4 | 52781 | 100.0 |
| 7 | 1308.5680 | 23250 | 498.0 | 32359 | 61.3 |
| 8 | 1309.5684 | 21614 | 606.3 | 39448 | 74.7 |
| 9 | 1310.5680 | 26069 | 400.9 | 26118 | 49.5 |
| 10 | 1311.5656 | 17923 | 110.0 | 7189 | 13.6 |

| # | m/z | Res. | S/N | I | I % |
|---|---|---|---|---|---|
| 1 | 369.3850 | | | 10684 | 26.6 |
| 2 | 1128.6206 | | | 12280 | 30.5 |
| 3 | 1265.5513 | | | 5703 | 14.2 |
| 4 | 1308.5645 | | | 40213 | 100.0 |
| 5 | 1331.5494 | | | 9109 | 22.7 |

Bruker Compass DataAnalysis 4.0 printed: 22.05.2019 10:51:51 Page 1 of 1

**Figure S4.** HRMS-ESI of **4a**.

# Compound Spectrum List Report

**Analysis Info**

| | | | |
|---|---|---|---|
| Analysis Name | D:\Data\Tevjashova\LCTA-3002_CHCl3+MeOH__pos_tune_wide.d | Acquisition Date | 18.06.2018 10:04:25 |
| Method | tune_wide.m | Operator | Korolev |
| Sample Name | | Instrument / Ser# | micrOTOF-Q II 10225 |
| Comment | | | |

**Acquisition Parameter**

| | | | | | |
|---|---|---|---|---|---|
| Source Type | ESI | Ion Polarity | Positive | Set Nebulizer | 0.4 Bar |
| Focus | Active | Set Capillary | 4500 V | Set Dry Heater | 180 °C |
| Scan Begin | 50 m/z | Set End Plate Offset | -500 V | Set Dry Gas | 4.0 l/min |
| Scan End | 3000 m/z | Set Collision Cell RF | 550.0 Vpp | Set Divert Valve | Source |

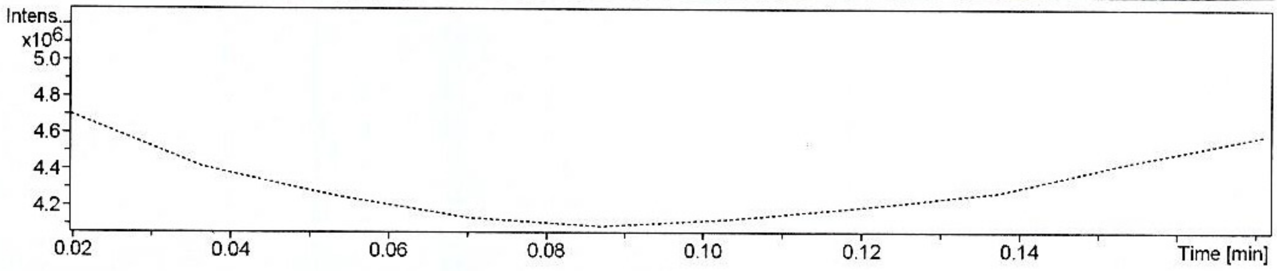


| # | RT [min] | Area | Int. Type | Intens. | S/N | Chromatogram | Max. m/z |
|---|---|---|---|---|---|---|---|
| n.a. | 0.1 | n.a. | Average spectrum | n.a. | n.a. | n.a. | 1321.5916 |

**+MS, 0.0-0.1min #(2-8)**

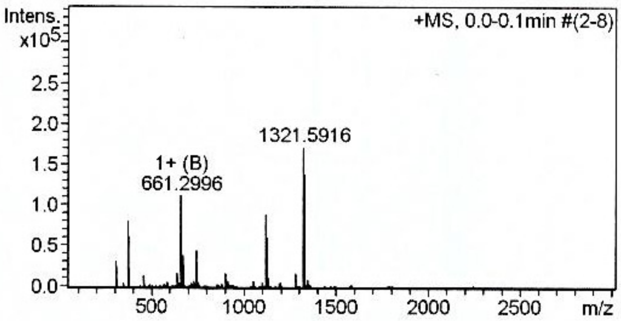


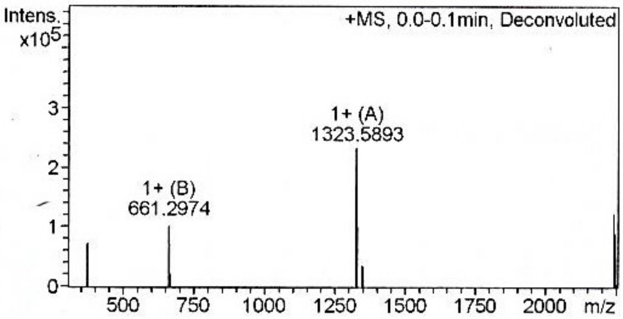


| # | m/z | Res. | S/N | I | I % |
|---|---|---|---|---|---|
| 1 | 375.2039 | 19257 | 1346.9 | 79403 | 46.3 |
| 2 | 661.2996 | 23884 | 588.9 | 111860 | 65.2 |
| 3 | 661.8006 | 18306 | 334.4 | 63671 | 37.1 |
| 4 | 662.3000 | 21528 | 511.5 | 97501 | 56.8 |
| 5 | 1120.0850 | 21570 | 327.5 | 64650 | 37.7 |
| 6 | 1120.5860 | 23936 | 448.3 | 88481 | 51.5 |
| 7 | 1321.5916 | 28582 | 1108.5 | 171644 | 100.0 |
| 8 | 1322.5936 | 25474 | 693.3 | 107232 | 62.5 |
| 9 | 1323.5910 | 25794 | 903.3 | 139474 | 81.3 |
| 10 | 1324.5920 | 22392 | 409.9 | 63261 | 36.9 |

| # | m/z | Res. | S/N | I | I % |
|---|---|---|---|---|---|
| 1 | 308.1972 | | | 31065 | 13.1 |
| 2 | 375.2039 | | | 79402 | 33.5 |
| 3 | 661.2974 | | | 111859 | 47.2 |
| 4 | 1323.5893 | | | 236973 | 100.0 |
| 5 | 1345.5717 | | | 38273 | 16.2 |
| 6 | 2238.1566 | | | 132787 | 56.0 |

Bruker Compass DataAnalysis 4.0 printed: 18.06.2018 10:14:34 Page 1 of 1

**Figure S5.** HRMS-ESI of **4b**.

# Compound Spectrum List Report

**Analysis Info**

| | | | |
|---|---|---|---|
| Analysis Name | D:\Data\Tevjashova\BEN-7-05-2019_MeOH_pos_tune_wide.d | Acquisition Date | 15.05.2019 10:00:40 |
| Method | tune_wide.m | Operator | Korolev |
| Sample Name | | Instrument / Ser# | micrOTOF-Q II 10225 |
| Comment | | | |

**Acquisition Parameter**

| | | | | | |
|---|---|---|---|---|---|
| Source Type | ESI | Ion Polarity | Positive | Set Nebulizer | 0.4 Bar |
| Focus | Active | Set Capillary | 4500 V | Set Dry Heater | 180 °C |
| Scan Begin | 50 m/z | Set End Plate Offset | -500 V | Set Dry Gas | 4.0 l/min |
| Scan End | 3000 m/z | Set Collision Cell RF | 550.0 Vpp | Set Divert Valve | Source |

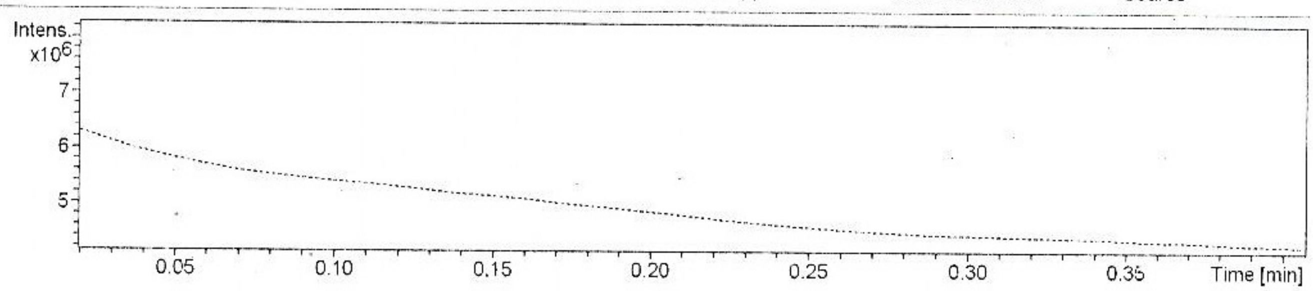


| # | RT [min] | Area | Int. Type | Intens. | S/N | Chromatogram | Max. m/z |
|---|---|---|---|---|---|---|---|
| n.a. | 0.2 | n.a. | Average spectrum | n.a. | n.a. | n.a. | 679.3016 |

**+MS, 0.0-0.3min #(2-18)**

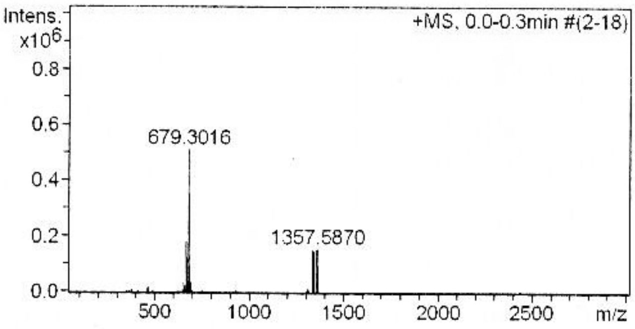


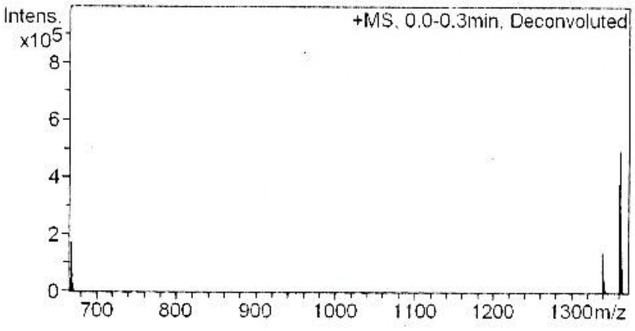


| # | m/z | Res. | S/N | I | I % |
|---|---|---|---|---|---|
| 1 | 668.3094 | 25849 | 1021.7 | 180191 | 35.3 |
| 2 | 679.3016 | 28942 | 2742.2 | 510797 | 100.0 |
| 3 | 679.8030 | 27038 | 1800.8 | 336296 | 65.8 |
| 4 | 680.3020 | 24609 | 2190.3 | 409997 | 80.3 |
| 5 | 680.8006 | 22528 | 1183.1 | 222046 | 43.5 |
| 6 | 681.2989 | 21965 | 663.5 | 124868 | 24.4 |
| 7 | 1335.6051 | 28252 | 1592.1 | 151242 | 29.6 |
| 8 | 1337.6051 | 30744 | 1545.6 | 146186 | 28.6 |
| 9 | 1357.5870 | 31196 | 1720.7 | 155606 | 30.5 |
| 10 | 1359.5899 | 27069 | 1386.2 | 124793 | 24.4 |

| # | m/z | Res. | S/N | I | I % |
|---|---|---|---|---|---|
| 1 | 666.3061 | | | 57556 | 13.8 |
| 2 | 668.3063 | | | 180191 | 43.1 |
| 3 | 1337.6033 | | | 146185 | 35.0 |
| 4 | 1338.6048 | | | 75841 | 18.2 |
| 5 | 1358.5902 | | | 417650 | 100.0 |

Bruker Compass DataAnalysis 4.0 printed: 15.05.2019 10:11:34 Page 1 of 1

**Figure S6.** HRMS-ESI of **4c**.

# Compound Spectrum List Report

**Analysis Info**

| | | | |
|---|---|---|---|
| Analysis Name | D:\Data\Tevjashova\11-10-2018_AcCN+MeOH_pos_tune_wide.d | Acquisition Date | 11.10.2018 13:44:36 |
| Method | tune_wide.m | Operator | Korolev |
| Sample Name | polyene-BSG(K6) | Instrument / Ser# | micrOTOF-Q II 10225 |
| Comment | | | |

**Acquisition Parameter**

| | | | | | |
|---|---|---|---|---|---|
| Source Type | ESI | Ion Polarity | Positive | Set Nebulizer | 1.0 Bar |
| Focus | Active | Set Capillary | 4500 V | Set Dry Heater | 180 °C |
| Scan Begin | 50 m/z | Set End Plate Offset | -500 V | Set Dry Gas | 4.0 l/min |
| Scan End | 3000 m/z | Set Collision Cell RF | 550.0 Vpp | Set Divert Valve | Source |

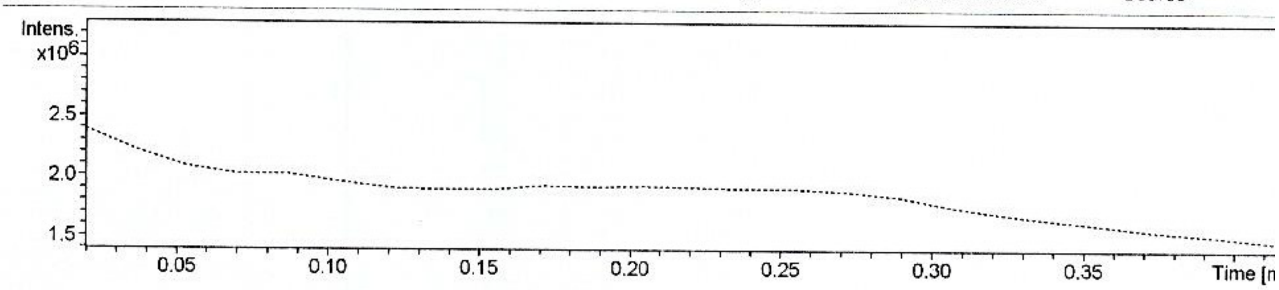


| # | RT [min] | Area | Int. Type | Intens. | S/N | Chromatogram | Max. m/z |
|---|---|---|---|---|---|---|---|
| n.a. | 0.2 | n.a. | Average spectrum | n.a. | n.a. | n.a. | 1349.6339 |

**+MS, 0.1-0.3min #(4-19)**

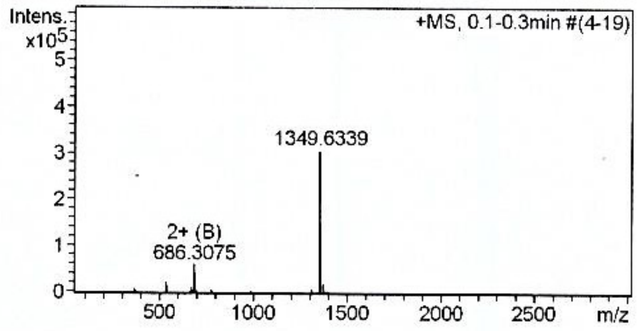


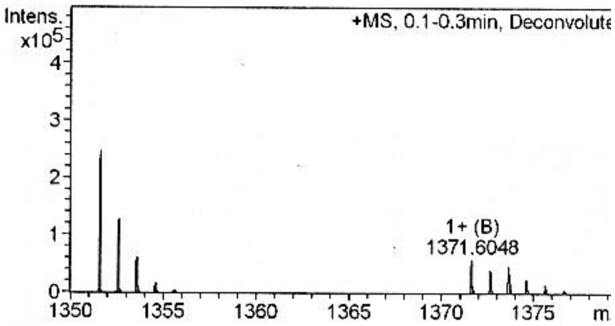


| # | m/z | Res. | S/N | I | I % |
|---|---|---|---|---|---|
| 1 | 537.3189 | 16511 | 842.3 | 21545 | 7.0 |
| 2 | 686.3075 | 23564 | 1428.7 | 60654 | 19.8 |
| 3 | 686.8094 | 20597 | 1030.2 | 43799 | 14.3 |
| 4 | 687.3095 | 19447 | 1212.2 | 51603 | 16.9 |
| 5 | 687.8098 | 17975 | 577.6 | 24631 | 8.0 |
| 6 | 1349.6339 | 33986 | 4099.8 | 306153 | 100.0 |
| 7 | 1350.6325 | 29105 | 2522.5 | 187674 | 61.3 |
| 8 | 1351.6335 | 31235 | 3455.1 | 256079 | 83.6 |
| 9 | 1352.6323 | 25553 | 1708.8 | 126189 | 41.2 |
| 10 | 1353.6260 | 22809 | 818.5 | 60228 | 19.7 |

| # | m/z | Res. | S/N | I | I % |
|---|---|---|---|---|---|
| 1 | 1351.6296 | | | 266675 | 100.0 |
| 2 | 1371.6048 | | | 60654 | 22.7 |

Bruker Compass DataAnalysis 4.0 printed: 11.10.2018 13:51:48 Page 1 of 1

**Figure S7.** HRMS-ESI of **4d**.

# Compound Spectrum List Report

**Analysis Info**

| | | | |
|---|---|---|---|
| Analysis Name | D:\Data\Tevjashova\BEN-13-05-2019_MeOH_pos_tune_wide.d | Acquisition Date | 15.05.2019 10:04:30 |
| Method | tune_wide.m | Operator | Korolev |
| Sample Name | | Instrument / Ser# | micrOTOF-Q II 10225 |
| Comment | | | |

**Acquisition Parameter**

| | | | | | |
|---|---|---|---|---|---|
| Source Type | ESI | Ion Polarity | Positive | Set Nebulizer | 0.4 Bar |
| Focus | Active | Set Capillary | 4500 V | Set Dry Heater | 180 °C |
| Scan Begin | 50 m/z | Set End Plate Offset | -500 V | Set Dry Gas | 4.0 l/min |
| Scan End | 3000 m/z | Set Collision Cell RF | 550.0 Vpp | Set Divert Valve | Source |

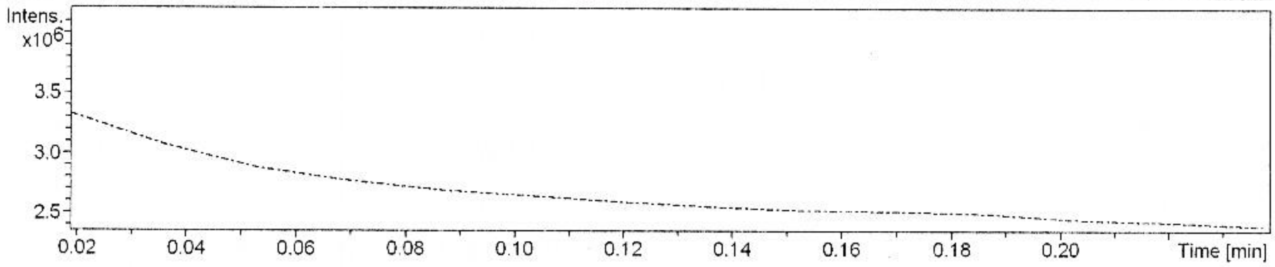


| # | RT [min] | Area | Int. Type | Intens. | S/N | Chromatogram | Max. m/z |
|---|---|---|---|---|---|---|---|
| n.a. | 0.2 | n.a. | Average spectrum | n.a. | n.a. | n.a. | 701.3227 |

**+MS, 0.1-0.2min #(4-14)**

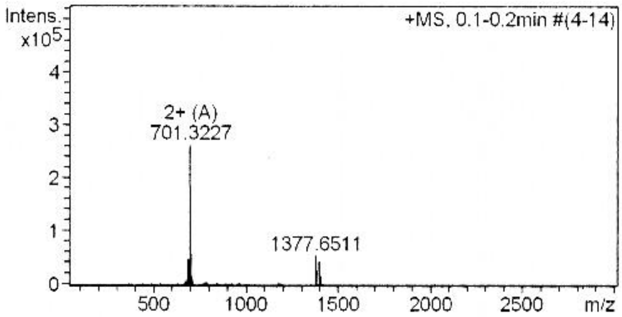


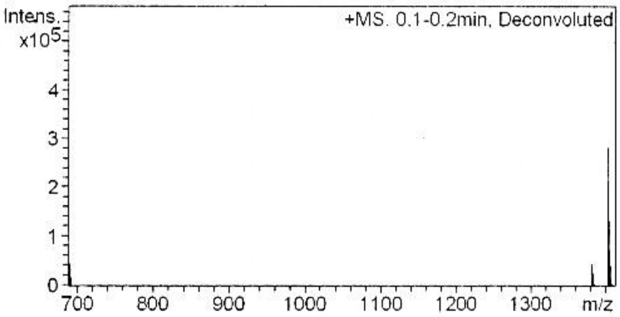


| # | m/z | Res. | S/N | I | I % |
|---|---|---|---|---|---|
| 1 | 689.3303 | 19182 | 332.0 | 47894 | 18.3 |
| 2 | 690.3296 | 18985 | 313.9 | 45486 | 17.4 |
| 3 | 700.3219 | 19858 | 1421.4 | 214944 | 82.0 |
| 4 | 700.8227 | 22597 | 1221.2 | 185066 | 70.6 |
| 5 | 701.3227 | 28128 | 1725.3 | 261991 | 100.0 |
| 6 | 701.8222 | 19967 | 742.4 | 113009 | 43.1 |
| 7 | 702.3196 | 21789 | 441.8 | 67422 | 25.7 |
| 8 | 1377.6511 | 23434 | 913.4 | 54648 | 20.9 |
| 9 | 1379.6456 | 24199 | 772.8 | 46057 | 17.6 |
| 10 | 1399.6289 | 24335 | 734.5 | 42011 | 16.0 |

| # | m/z | Res. | S/N | I | I % |
|---|---|---|---|---|---|
| 1 | 689.3273 | | | 47893 | 15.8 |
| 2 | 1379.6452 | | | 46057 | 15.2 |
| 3 | 1400.6320 | | | 302574 | 100.0 |

Bruker Compass DataAnalysis 4.0 printed: 15.05.2019 10:14:39 Page 1 of 1

**Figure S8.** HRMS-ESI of **4e**.

# Compound Spectrum List Report

**Analysis Info**

| | | | |
|---|---|---|---|
| Analysis Name | D:\Data\Tevjashova\25-09-2018_fr_2_AcCN+MeOH_pos_tune_wide.d | Acquisition Date | 09.10.2018 11:07:00 |
| Method | tune_wide.m | Operator | Korolev |
| Sample Name | | Instrument / Ser# | micrOTOF-Q II 10225 |
| Comment | | | |

**Acquisition Parameter**

| | | | | | |
|---|---|---|---|---|---|
| Source Type | ESI | Ion Polarity | Positive | Set Nebulizer | 0.4 Bar |
| Focus | Active | Set Capillary | 4500 V | Set Dry Heater | 180 °C |
| Scan Begin | 50 m/z | Set End Plate Offset | -500 V | Set Dry Gas | 4.0 l/min |
| Scan End | 3000 m/z | Set Collision Cell RF | 550.0 Vpp | Set Divert Valve | Source |

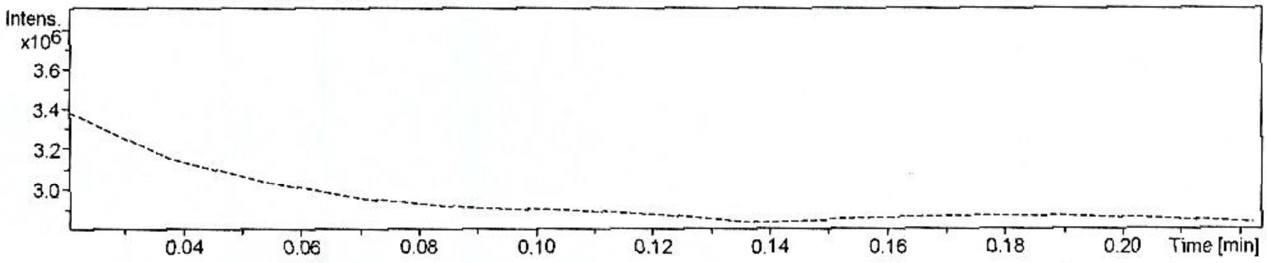


| # | RT [min] | Area | Int. Type | Intens. | S/N | Chromatogram | Max. m/z |
|---|---|---|---|---|---|---|---|
| n.a. | 0.1 | n.a. | Average spectrum | n.a. | n.a. | n.a. | 1395.6237 |

**+MS, 0.0-0.1min #(2-6)**

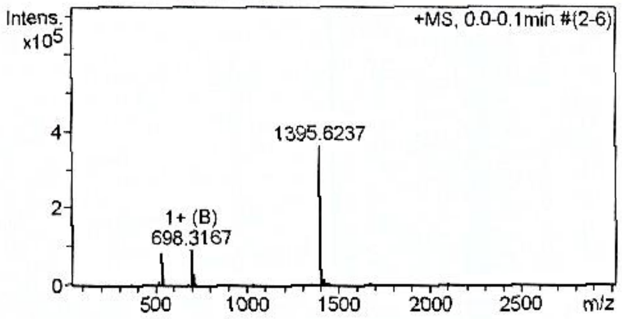


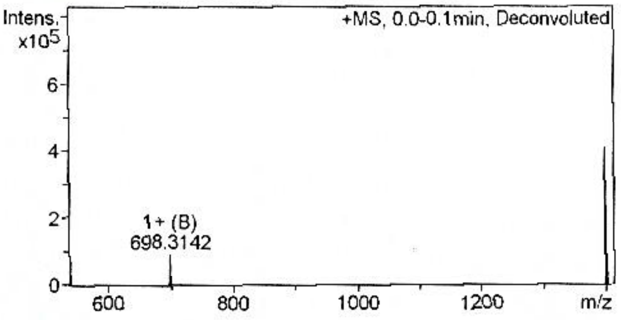


| # | m/z | Res. | S/N | I | I % |
|---|---|---|---|---|---|
| 1 | 537.3205 | 16721 | 1830.6 | 85570 | 23.6 |
| 2 | 698.3167 | 20738 | 1037.4 | 93482 | 25.8 |
| 3 | 698.8190 | 21155 | 767.4 | 69269 | 19.1 |
| 4 | 699.3164 | 21283 | 938.0 | 84782 | 23.4 |
| 5 | 699.8160 | 18576 | 464.6 | 42072 | 11.6 |
| 6 | 1395.6237 | 30703 | 3076.1 | 361931 | 100.0 |
| 7 | 1396.6241 | 31055 | 2255.4 | 264714 | 73.1 |
| 8 | 1397.6232 | 35139 | 3079.3 | 360494 | 99.6 |
| 9 | 1398.6215 | 31392 | 1764.2 | 206033 | 56.9 |
| 10 | 1399.6183 | 27477 | 866.3 | 100927 | 27.9 |

| # | m/z | Res. | S/N | I | I % |
|---|---|---|---|---|---|
| 1 | 537.3206 | | | 85570 | 25.6 |
| 2 | 698.3142 | | | 93482 | 28.0 |
| 3 | 1396.6192 | | | 333981 | 100.0 |

Bruker Compass DataAnalysis 4.0 printed: 09.10.2018 11:11:20 Page 1 of 1

**Figure S9.** HRMS-ESI of **4f**.

# Compound Spectrum List Report

**Analysis Info**

| | | | |
|---|---|---|---|
| Analysis Name | D:\Data\Tevjashova\BEN-22-04-2019-II_MeOH_pos_tune_wide.d | Acquisition Date | 22.05.2019 10:38:27 |
| Method | tune_wide.m | Operator | Korolev |
| Sample Name | | Instrument / Ser# | micrOTOF-Q II 10225 |
| Comment | | | |

**Acquisition Parameter**

| | | | | | |
|---|---|---|---|---|---|
| Source Type | ESI | Ion Polarity | Positive | Set Nebulizer | 0.4 Bar |
| Focus | Not active | Set Capillary | 4500 V | Set Dry Heater | 180 °C |
| Scan Begin | 50 m/z | Set End Plate Offset | -500 V | Set Dry Gas | 4.0 l/min |
| Scan End | 3000 m/z | Set Collision Cell RF | 550.0 Vpp | Set Divert Valve | Source |

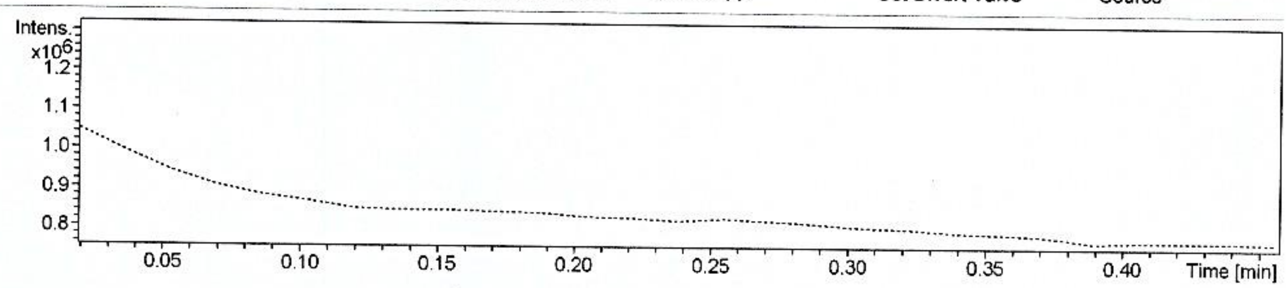


| # | RT [min] | Area | Int. Type | Intens. | S/N | Chromatogram | Max. m/z |
|---|---|---|---|---|---|---|---|
| n.a. | 0.1 | n.a. | Average spectrum | n.a. | n.a. | n.a. | 375.2071 |

**+MS, 0.0-0.1min #(2-8)**

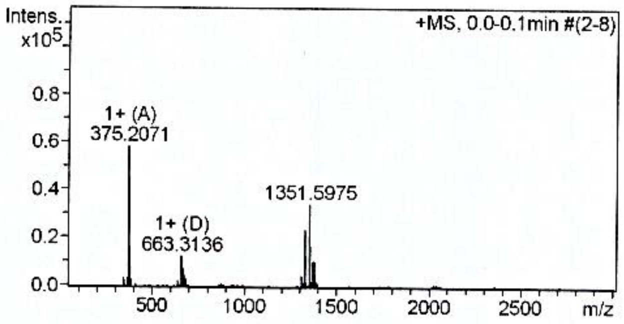


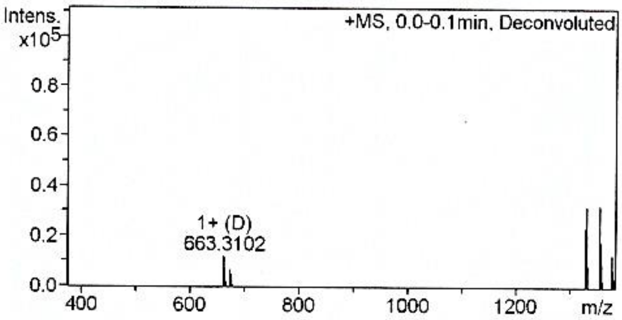


| # | m/z | Res. | S/N | I | I % |
|---|---|---|---|---|---|
| 1 | 375.2071 | 9767 | 5537.5 | 57997 | 100.0 |
| 2 | 663.3136 | 13171 | 591.5 | 12371 | 21.3 |
| 3 | 664.3133 | 13127 | 516.4 | 10834 | 18.7 |
| 4 | 1325.6184 | 15171 | 472.3 | 23409 | 40.4 |
| 5 | 1326.6226 | 16240 | 320.0 | 15924 | 27.5 |
| 6 | 1327.6145 | 15926 | 445.6 | 22115 | 38.1 |
| 7 | 1351.5975 | 14978 | 739.3 | 34252 | 59.1 |
| 8 | 1352.5997 | 14995 | 431.6 | 19952 | 34.4 |
| 9 | 1353.5999 | 14824 | 649.0 | 29895 | 51.5 |
| 10 | 1354.5969 | 15845 | 334.3 | 15368 | 26.5 |

| # | m/z | Res. | S/N | I | I % |
|---|---|---|---|---|---|
| 1 | 375.2069 | | | 57997 | 100.0 |
| 2 | 663.3102 | | | 12371 | 21.3 |
| 3 | 674.2993 | | | 7160 | 12.3 |
| 4 | 1326.6142 | | | 24060 | 41.5 |
| 5 | 1352.5949 | | | 22739 | 39.2 |
| 6 | 1374.5745 | | | 9698 | 16.7 |

Bruker Compass DataAnalysis 4.0 printed: 22.05.2019 10:55:02 Page 1 of 1

**Figure S10.** HRMS-ESI of **4g**.

# Compound Spectrum List Report

**Analysis Info**

| | | | |
|---|---|---|---|
| Analysis Name | D:\Data\Tevjashova\BEN-23-11-2018_MeOH_pos_tune_wide.d | Acquisition Date | 23.11.2018 12:24:50 |
| Method | tune_wide.m | Operator | Korolev |
| Sample Name | | Instrument / Ser# | micrOTOF-Q II 10225 |
| Comment | | | |

**Acquisition Parameter**

| | | | | | |
|---|---|---|---|---|---|
| Source Type | ESI | Ion Polarity | Positive | Set Nebulizer | 0.4 Bar |
| Focus | Not active | Set Capillary | 4500 V | Set Dry Heater | 180 °C |
| Scan Begin | 50 m/z | Set End Plate Offset | -500 V | Set Dry Gas | 4.0 l/min |
| Scan End | 3000 m/z | Set Collision Cell RF | 550.0 Vpp | Set Divert Valve | Source |

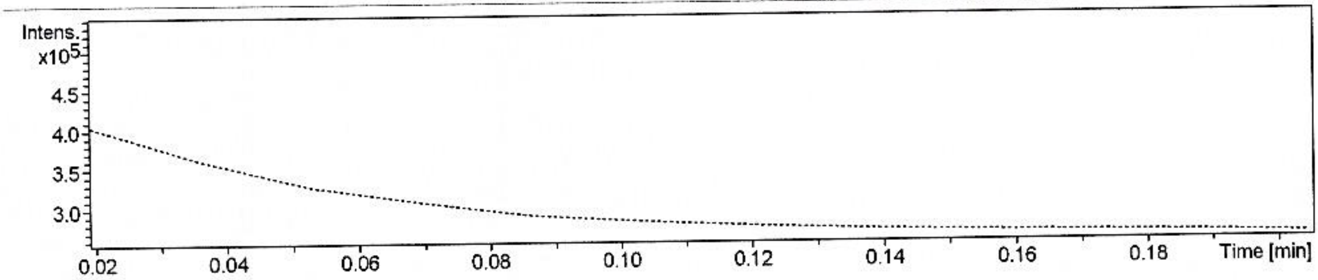


| # | RT [min] | Area | Int. Type | Intens. | S/N | Chromatogram | Max. m/z |
|---|---|---|---|---|---|---|---|
| n.a. | 0.1 | n.a. | Average spectrum | n.a. | n.a. | n.a. | 578.8179 |

**+MS, 0.1-0.2min #(3-9)**

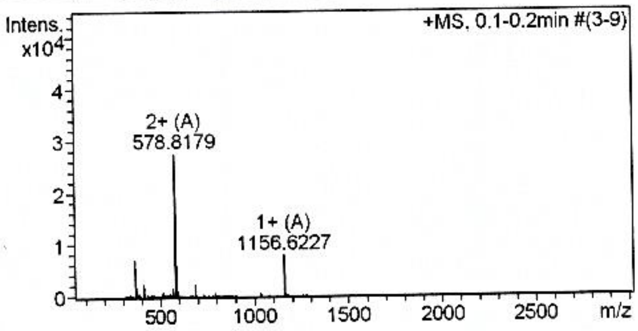


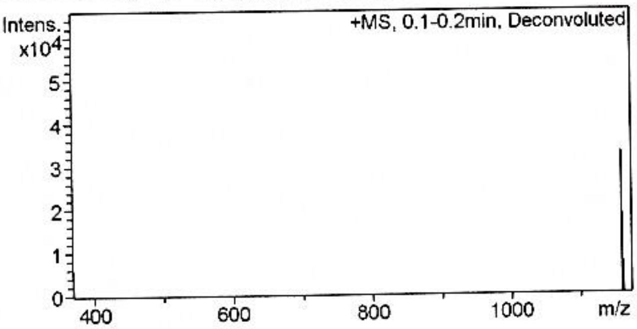


| # | m/z | Res. | S/N | I | I % |
|---|---|---|---|---|---|
| 1 | 369.3851 | 10704 | 855.4 | 7184 | 26.2 |
| 2 | 413.2662 | 12077 | 198.3 | 2284 | 8.3 |
| 3 | 578.8179 | 11976 | 1406.3 | 27451 | 100.0 |
| 4 | 579.3199 | 12294 | 892.2 | 17414 | 63.4 |
| 5 | 579.8218 | 12753 | 317.2 | 6198 | 22.6 |
| 6 | 580.3228 | 12138 | 91.9 | 1805 | 6.6 |
| 7 | 687.0665 | 12401 | 127.2 | 2240 | 8.2 |
| 8 | 687.4007 | 11919 | 128.8 | 2267 | 8.3 |
| 9 | 1156.6227 | 14798 | 673.6 | 8032 | 29.3 |
| 10 | 1157.6276 | 17246 | 527.3 | 6284 | 22.9 |

| # | m/z | Res. | S/N | I | I % |
|---|---|---|---|---|---|
| 1 | 369.3846 | | | 7184 | 20.2 |
| 2 | 1156.6277 | | | 35481 | 100.0 |

Bruker Compass DataAnalysis 4.0 printed: 23.11.2018 12:29:16 Page 1 of 1

**Figure S11.** HRMS-ESI of **5a**.

# Compound Spectrum List Report

**Analysis Info**

| | | | |
|---|---|---|---|
| Analysis Name | D:\Data\Tevjashova\BEN-28-11-2018_(III)_MeOH_pos_tune_low.d | Acquisition Date | 29.11.2018 11:41:52 |
| Method | tune_wide.m | Operator | Korolev |
| Sample Name | | Instrument / Ser# | micrOTOF-Q II 10225 |
| Comment | | | |

**Acquisition Parameter**

| | | | | | | | |
|---|---|---|---|---|---|---|---|
| Source Type | ESI | Ion Polarity | Positive | Set Nebulizer | 0.4 Bar |
| Focus | Active | Set Capillary | 4500 V | Set Dry Heater | 180 °C |
| Scan Begin | 50 m/z | Set End Plate Offset | -500 V | Set Dry Gas | 4.0 l/min |
| Scan End | 3000 m/z | Set Collision Cell RF | 550.0 Vpp | Set Divert Valve | Source |

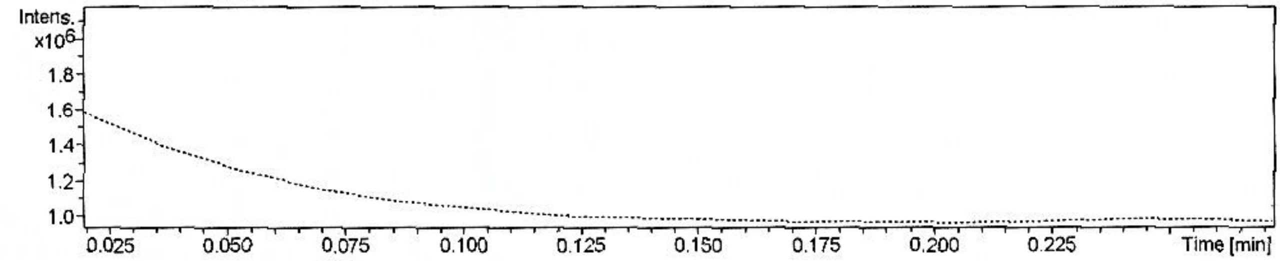


| # | RT [min] | Area | Int. Type | Intens. | S/N | Chromatogram | Max. m/z |
|---|---|---|---|---|---|---|---|
| n.a. | 0.1 | n.a. | Average spectrum | n.a. | n.a. | n.a. | 585.8225 |

**+MS, 0.0-0.1min #(2-5)**

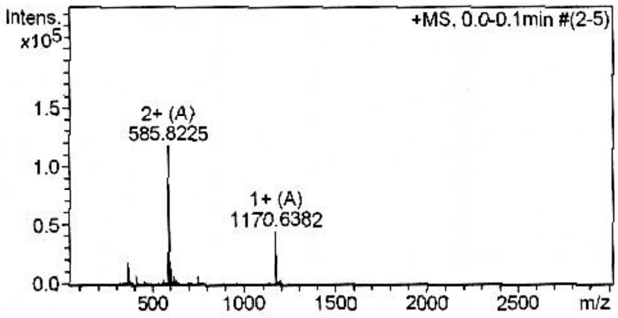


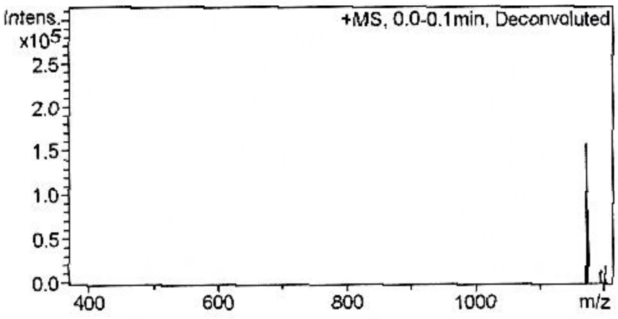


| # | m/z | Res. | S/N | I | I % |
|---|---|---|---|---|---|
| 1 | 369.3799 | 13752 | 391.9 | 18066 | 15.4 |
| 2 | 585.8225 | 23289 | 1166.8 | 117690 | 100.0 |
| 3 | 586.3242 | 18548 | 625.9 | 63133 | 53.6 |
| 4 | 586.8263 | 20716 | 243.5 | 24570 | 20.9 |
| 5 | 596.8131 | 21141 | 141.8 | 14261 | 12.1 |
| 6 | 597.3143 | 21618 | 90.7 | 9124 | 7.8 |
| 7 | 599.8384 | 21210 | 197.8 | 19859 | 16.9 |
| 8 | 600.3407 | 17311 | 105.9 | 10643 | 9.0 |
| 9 | 1170.6382 | 25511 | 574.5 | 45076 | 38.3 |
| 10 | 1171.6423 | 24652 | 298.0 | 23368 | 19.9 |

| # | m/z | Res. | S/N | I | I % |
|---|---|---|---|---|---|
| 1 | 369.3799 | | | 18065 | 11.1 |
| 2 | 1170.6382 | | | 162764 | 100.0 |
| 3 | 1192.6187 | | | 14260 | 8.8 |
| 4 | 1198.6696 | | | 19858 | 12.2 |

Bruker Compass DataAnalysis 4.0 printed: 29.11.2018 11:45:00 Page 1 of 1

**Figure S12.** HRMS-ESI of **5b**.

# Compound Spectrum List Report

**Analysis Info**

| | | | |
|---|---|---|---|
| Analysis Name | D:\Data\Tevjashova\BEN-30-11-2018_(II)_MeOH_pos_tune_wide.d | Acquisition Date | 03.12.2018 11:07:39 |
| Method | tune_wide.m | Operator | Korolev |
| Sample Name | | Instrument / Ser# | micrOTOF-Q II 10225 |
| Comment | | | |

**Acquisition Parameter**

| | | | | | |
|---|---|---|---|---|---|
| Source Type | ESI | Ion Polarity | Positive | Set Nebulizer | 0.4 Bar |
| Focus | Not active | Set Capillary | 4500 V | Set Dry Heater | 180 °C |
| Scan Begin | 50 m/z | Set End Plate Offset | -500 V | Set Dry Gas | 4.0 l/min |
| Scan End | 3000 m/z | Set Collision Cell RF | 550.0 Vpp | Set Divert Valve | Source |

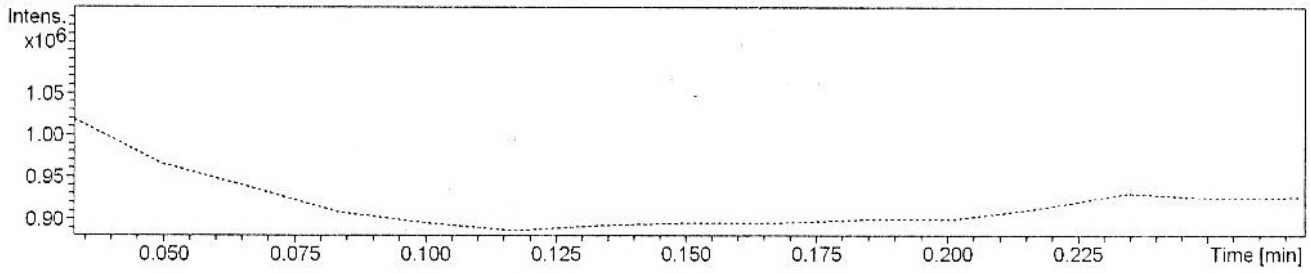


| # | RT [min] | Area | Int. Type | Intens. | S/N | Chromatogram | Max. m/z |
|---|---|---|---|---|---|---|---|
| n.a. | 0.2 | n.a. | Average spectrum | n.a. | n.a. | n.a. | 592.8309 |

**+MS, 0.1-0.3min #(3-14)**

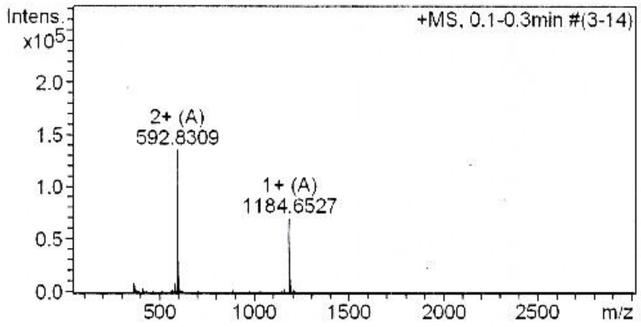


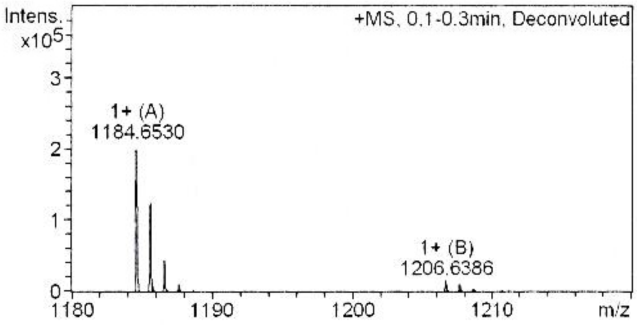


| # | m/z | Res. | S/N | I | I % |
|---|---|---|---|---|---|
| 1 | 578.8343 | 12284 | 221.3 | 8579 | 6.3 |
| 2 | 592.8309 | 10509 | 3671.7 | 135922 | 100.0 |
| 3 | 593.3319 | 10620 | 2295.8 | 84871 | 62.4 |
| 4 | 593.8339 | 12183 | 882.6 | 32598 | 24.0 |
| 5 | 594.3341 | 12166 | 222.7 | 8236 | 6.1 |
| 6 | 603.8219 | 12365 | 455.9 | 16331 | 12.0 |
| 7 | 604.3248 | 12395 | 286.9 | 10272 | 7.6 |
| 8 | 1184.6527 | 13514 | 3507.3 | 70661 | 52.0 |
| 9 | 1185.6528 | 14484 | 2095.3 | 42121 | 31.0 |
| 10 | 1186.6560 | 15338 | 637.4 | 12805 | 9.4 |

| # | m/z | Res. | S/N | I | I % |
|---|---|---|---|---|---|
| 1 | 1184.6530 | | | 206582 | 100.0 |
| 2 | 1206.6386 | | | 16331 | 7.9 |

Bruker Compass DataAnalysis 4.0 printed: 03.12.2018 11:12:09 Page 1 of 1

**Figure S13.** HRMS-ESI of **5c**.

# Compound Spectrum List Report

**Analysis Info**

| | | | |
|---|---|---|---|
| Analysis Name | D:\Data\Tevjashova\LCTA-3063_MeOH_pos_tune_wide.d | Acquisition Date | 22.05.2019 10:29:41 |
| Method | tune_wide.m | Operator | Korolev |
| Sample Name | . | Instrument / Ser# | micrOTOF-Q II 10225 |
| Comment | | | |

**Acquisition Parameter**

| | | | | | |
|---|---|---|---|---|---|
| Source Type | ESI | Ion Polarity | Positive | Set Nebulizer | 0.4 Bar |
| Focus | Active | Set Capillary | 4500 V | Set Dry Heater | 180 °C |
| Scan Begin | 50 m/z | Set End Plate Offset | -500 V | Set Dry Gas | 4.0 l/min |
| Scan End | 3000 m/z | Set Collision Cell RF | 550.0 Vpp | Set Divert Valve | Source |

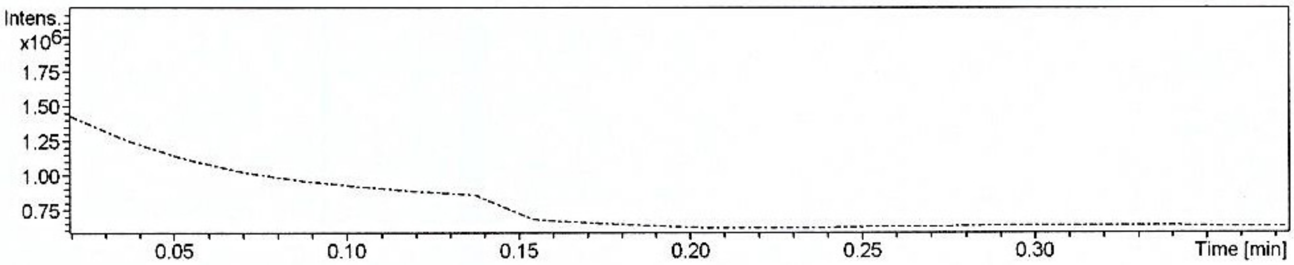


| # | RT [min] | Area | Int. Type | Intens. | S/N | Chromatogram | Max. m/z |
|---|---|---|---|---|---|---|---|
| n.a. | 0.1 | n.a. | Average spectrum | n.a. | n.a. | n.a. | 1198.6682 |

**+MS, 0.0-0.1min #(2-6)**

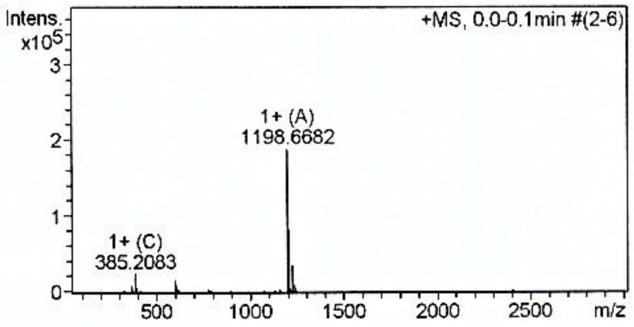


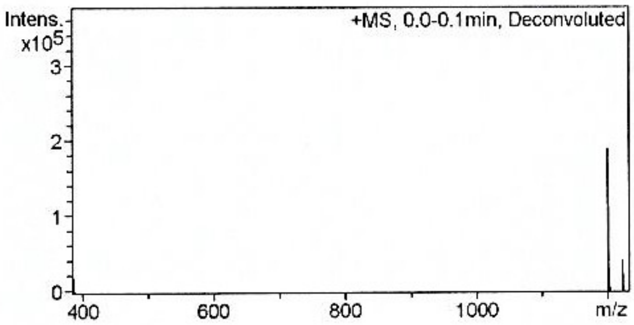


| # | m/z | Res. | S/N | I | I % |
|---|---|---|---|---|---|
| 1 | 369.3852 | 13664 | 382.4 | 7498 | 4.0 |
| 2 | 385.2083 | 14349 | 1104.6 | 24175 | 12.9 |
| 3 | 599.8362 | 17943 | 305.8 | 14050 | 7.5 |
| 4 | 600.3389 | 15061 | 165.2 | 7596 | 4.0 |
| 5 | 1198.6682 | 30635 | 2789.6 | 187642 | 100.0 |
| 6 | 1199.6719 | 22058 | 1226.5 | 82388 | 43.9 |
| 7 | 1200.6682 | 21767 | 381.1 | 25576 | 13.6 |
| 8 | 1220.6439 | 23764 | 540.9 | 35223 | 18.8 |
| 9 | 1221.6517 | 22530 | 271.4 | 17655 | 9.4 |
| 10 | 1236.6273 | 18815 | 166.8 | 10609 | 5.7 |

| # | m/z | Res. | S/N | I | I % |
|---|---|---|---|---|---|
| 1 | 385.2084 | | | 24174 | 12.0 |
| 2 | 1198.6675 | | | 201692 | 100.0 |
| 3 | 1220.6453 | | | 40468 | 20.1 |

Bruker Compass DataAnalysis 4.0 printed: 22.05.2019 10:52:32 Page 1 of 1

**Figure S14.** HRMS-ESI of **5d**.

# Compound Spectrum List Report

**Analysis Info**

| | | | |
|---|---|---|---|
| Analysis Name | D:\Data\Tevjashova\BEN-12-12-2018_MeOH_pos_tune_wide.d | Acquisition Date | 12.12.2018 10:45:53 |
| Method | tune_wide.m | Operator | Korolev |
| Sample Name | | Instrument / Ser# | micrOTOF-Q II 10225 |
| Comment | | | |

**Acquisition Parameter**

| | | | | | |
|---|---|---|---|---|---|
| Source Type | ESI | Ion Polarity | Positive | Set Nebulizer | 0.4 Bar |
| Focus | Active | Set Capillary | 4500 V | Set Dry Heater | 180 °C |
| Scan Begin | 50 m/z | Set End Plate Offset | -500 V | Set Dry Gas | 4.0 l/min |
| Scan End | 3000 m/z | Set Collision Cell RF | 550.0 Vpp | Set Divert Valve | Source |

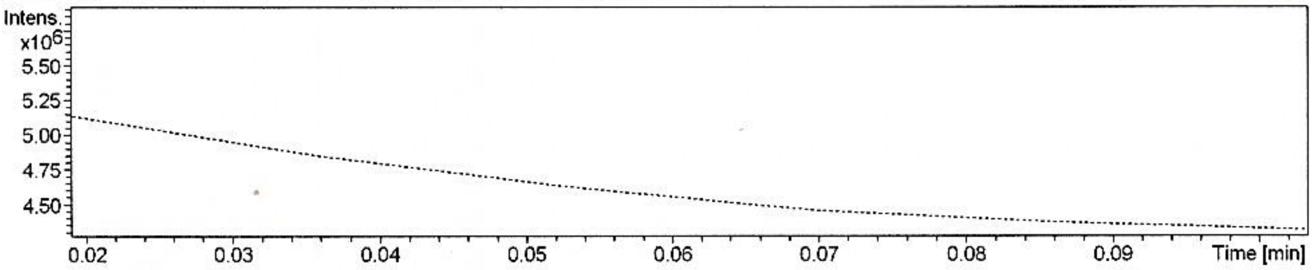


| # | RT [min] | Area | Int. Type | Intens. | S/N | Chromatogram | Max. m/z |
|---|---|---|---|---|---|---|---|
| n.a. | 0.1 | n.a. | Average spectrum | n.a. | n.a. | n.a. | 1226.7080 |

**+MS, 0.0-0.1min #(2-5)**

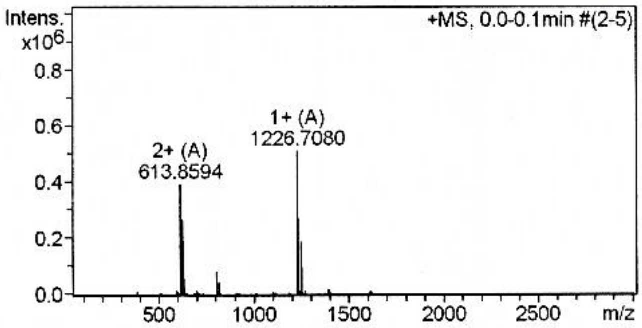


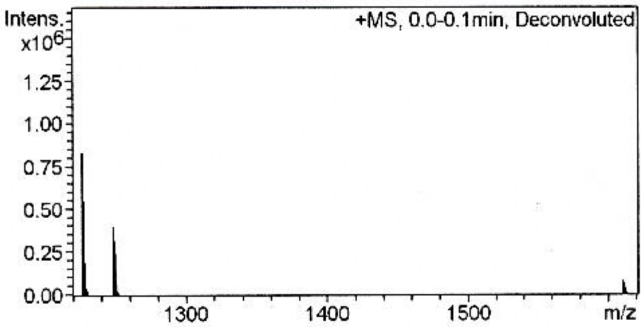


| # | m/z | Res. | S/N | I | I % |
|---|---|---|---|---|---|
| 1 | 613.8594 | 25236 | 2432.4 | 393482 | 76.9 |
| 2 | 614.3601 | 23876 | 1501.6 | 243155 | 47.5 |
| 3 | 624.8492 | 19776 | 1594.3 | 263101 | 51.4 |
| 4 | 625.3500 | 24625 | 1220.9 | 201663 | 39.4 |
| 5 | 805.4681 | 24592 | 449.9 | 80425 | 15.7 |
| 6 | 1226.7080 | 31532 | 3694.1 | 511615 | 100.0 |
| 7 | 1227.7078 | 26938 | 2328.3 | 322330 | 63.0 |
| 8 | 1228.7095 | 23095 | 694.3 | 96110 | 18.8 |
| 9 | 1248.6850 | 29678 | 1355.7 | 185951 | 36.3 |
| 10 | 1249.6890 | 25734 | 780.2 | 106982 | 20.9 |

| # | m/z | Res. | S/N | I | I % |
|---|---|---|---|---|---|
| 1 | 1226.7078 | | | 905095 | 100.0 |
| 2 | 1248.6880 | | | 449052 | 49.6 |
| 3 | 1609.9257 | | | 89076 | 9.8 |

OK



**Figure S15.** HRMS-ESI of **5e**.

# Compound Spectrum List Report

**Analysis Info**

| | | | |
|---|---|---|---|
| Analysis Name | D:\Data\Tevjashova\BEN-28-11-2018_(I)_MeOH_pos_tune_wide.d | Acquisition Date | 29.11.2018 10:24:16 |
| Method | tune_wide.m | Operator | Korolev |
| Sample Name | | Instrument / Ser# | micrOTOF-Q II 10225 |
| Comment | | | |

**Acquisition Parameter**

| | | | | | |
|---|---|---|---|---|---|
| Source Type | ESI | Ion Polarity | Positive | Set Nebulizer | 0.4 Bar |
| Focus | Not active | Set Capillary | 4500 V | Set Dry Heater | 180 °C |
| Scan Begin | 50 m/z | Set End Plate Offset | -500 V | Set Dry Gas | 4.0 l/min |
| Scan End | 3000 m/z | Set Collision Cell RF | 550.0 Vpp | Set Divert Valve | Source |

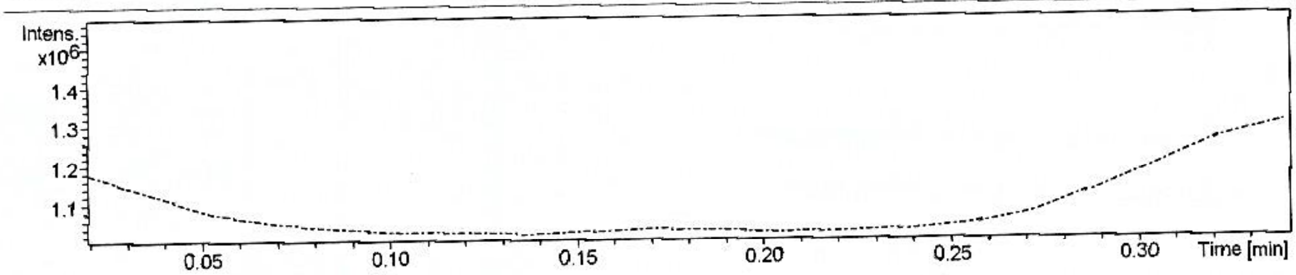


| # | RT [min] | Area | Int. Type | Intens. | S/N | Chromatogram | Max. m/z |
|---|---|---|---|---|---|---|---|
| n.a. | 0.1 | n.a. | Average spectrum | n.a. | n.a. | n.a. | 622.8432 |

**+MS, 0.0-0.1min #(2-6)**

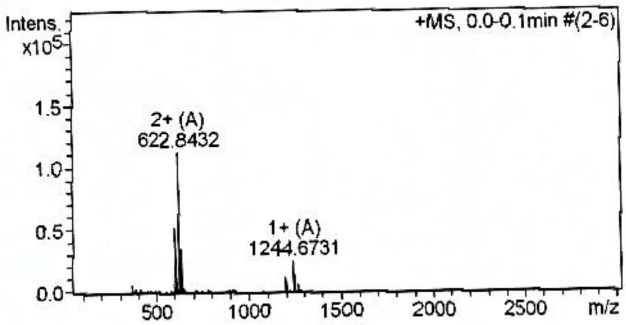


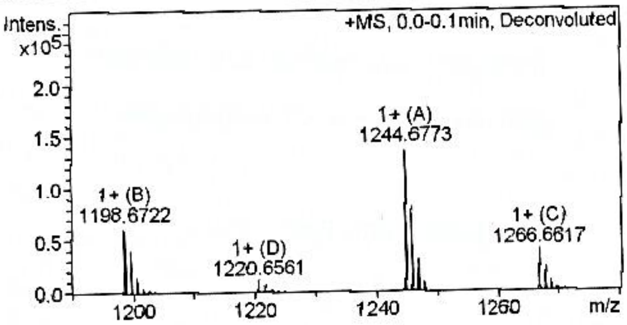


| # | m/z | Res. | S/N | I | I % |
|---|---|---|---|---|---|
| 1 | 599.8403 | 11127 | 1100.0 | 52862 | 46.9 |
| 2 | 600.3432 | 11681 | 754.4 | 36244 | 32.1 |
| 3 | 600.8436 | 12245 | 280.1 | 13472 | 11.9 |
| 4 | 622.8432 | 11023 | 2426.2 | 112745 | 100.0 |
| 5 | 623.3445 | 11133 | 1546.9 | 71848 | 63.7 |
| 6 | 623.8457 | 12046 | 604.4 | 28079 | 24.9 |
| 7 | 633.8354 | 12007 | 769.9 | 35233 | 31.2 |
| 8 | 634.3362 | 11301 | 465.0 | 21283 | 18.9 |
| 9 | 1244.6731 | 15328 | 969.4 | 25686 | 22.8 |
| 10 | 1245.6746 | 14417 | 511.9 | 13574 | 12.0 |

| # | m/z | Res. | S/N | I | I % |
|---|---|---|---|---|---|
| 1 | 1198.6722 | | | 64461 | 46.6 |
| 2 | 1220.6561 | | | 12260 | 8.9 |
| 3 | 1244.6773 | | | 138429 | 100.0 |
| 4 | 1266.6617 | | | 40802 | 29.5 |

Bruker Compass DataAnalysis 4.0 printed: 29.11.2018 10:30:38 Page 1 of 1

**Figure S16.** HRMS-ESI of **5f**.

# Compound Spectrum List Report

**Analysis Info**

| | | | |
|---|---|---|---|
| Analysis Name | D:\Data\Tevjashova\BEN-22-04-2019-1_MeOH_pos_tune_wide.d | Acquisition Date | 22.05.2019 10:34:07 |
| Method | tune_wide.m | Operator | Korolev |
| Sample Name | | Instrument / Ser# | micrOTOF-Q II 10225 |
| Comment | | | |

**Acquisition Parameter**

| | | | | | |
|---|---|---|---|---|---|
| Source Type | ESI | Ion Polarity | Positive | Set Nebulizer | 0.4 Bar |
| Focus | Not active | Set Capillary | 4500 V | Set Dry Heater | 180 °C |
| Scan Begin | 50 m/z | Set End Plate Offset | -500 V | Set Dry Gas | 4.0 l/min |
| Scan End | 3000 m/z | Set Collision Cell RF | 550.0 Vpp | Set Divert Valve | Source |

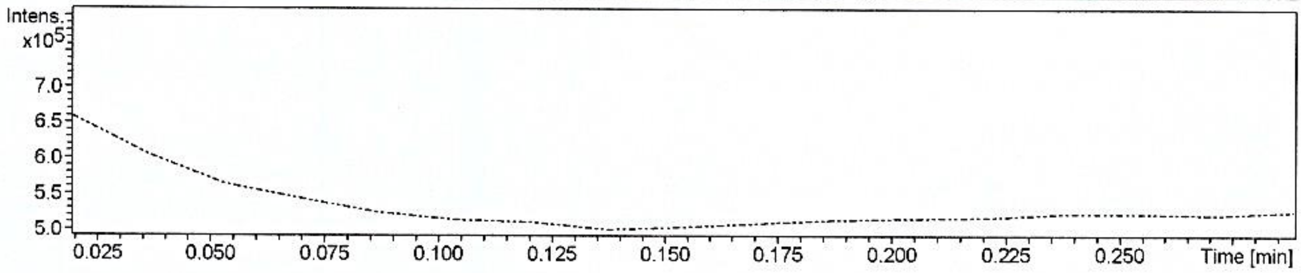


| # | RT [min] | Area | Int. Type | Intens. | S/N | Chromatogram | Max. m/z |
|---|---|---|---|---|---|---|---|
| n.a. | 0.1 | n.a. | Average spectrum | n.a. | n.a. | n.a. | 1200.6495 |

**+MS, 0.0-0.1min #(2-7)**

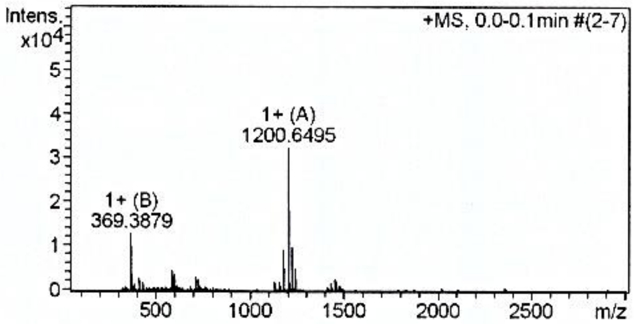


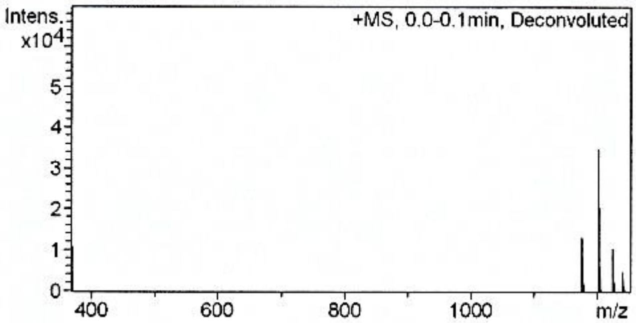


| # | m/z | Res. | S/N | I | I % |
|---|---|---|---|---|---|
| 1 | 369.3879 | 10199 | 854.5 | 12859 | 39.8 |
| 2 | 587.8417 | 12314 | 169.0 | 4561 | 14.1 |
| 3 | 1174.6706 | 15863 | 314.1 | 9096 | 28.2 |
| 4 | 1175.6783 | 14311 | 166.1 | 4816 | 14.9 |
| 5 | 1200.6495 | 14451 | 1147.7 | 32280 | 100.0 |
| 6 | 1201.6567 | 14224 | 647.2 | 18194 | 56.4 |
| 7 | 1202.6581 | 14738 | 191.3 | 5387 | 16.7 |
| 8 | 1222.6297 | 15398 | 357.6 | 9838 | 30.5 |
| 9 | 1223.6387 | 16929 | 259.8 | 7145 | 22.1 |
| 10 | 1238.6093 | 15335 | 186.1 | 5041 | 15.6 |

Ok

| # | m/z | Res. | S/N | I | I % |
|---|---|---|---|---|---|
| 1 | 369.3874 | | | 12858 | 35.7 |
| 2 | 1174.6731 | | | 13657 | 37.9 |
| 3 | 1200.6510 | | | 36001 | 100.0 |
| 4 | 1222.6320 | | | 10970 | 30.5 |
| 5 | 1238.6063 | | | 5040 | 14.0 |

Bruker Compass DataAnalysis 4.0 printed: 22.05.2019 10:54:11 Page 1 of 1

**Figure S17.** HRMS-ESI of **5g**.

# Compound Spectrum List Report

**Analysis Info**

| | | | |
|---|---|---|---|
| Analysis Name | D:\Data\MM\MM-051-2.d | Acquisition Date | 9/2/2023 5:48:02 PM |
| Method | tune_norm.m | Operator | Mitrokhov |
| Sample Name | Tune wide | Instrument / Ser# | micrOTOF-Q II 10225 |
| Comment | | | |

**Acquisition Parameter**

| | | | | | |
|---|---|---|---|---|---|
| Source Type | ESI | Ion Polarity | Positive | Set Nebulizer | 0.4 Bar |
| Focus | Active | Set Capillary | 4500 V | Set Dry Heater | 180 °C |
| Scan Begin | 50 m/z | Set End Plate Offset | -500 V | Set Dry Gas | 4.0 l/min |
| Scan End | 3000 m/z | Set Collision Cell RF | 550.0 Vpp | Set Divert Valve | Source |

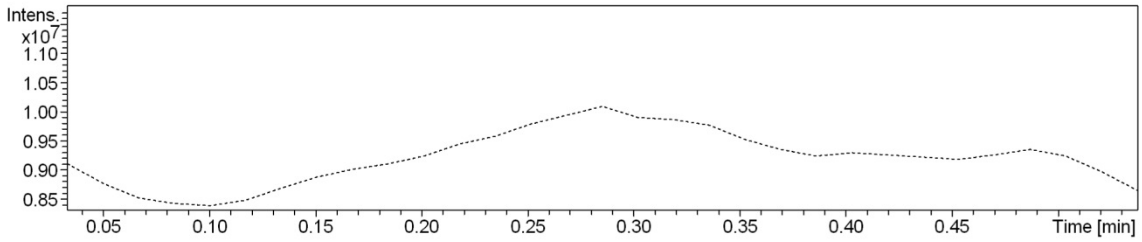


| # | RT [min] | Area | Int. Type | Intens. | S/N | Chromatogram | Max. m/z |
|---|---|---|---|---|---|---|---|
| n.a. | 0.2 | n.a. | Average spectrum | n.a. | n.a. | n.a. | 817.5142 |

**+MS, 0.1-0.4min #(2-20)**

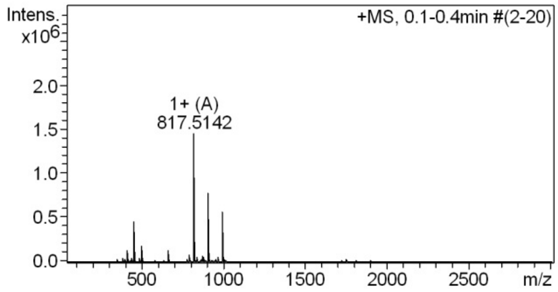


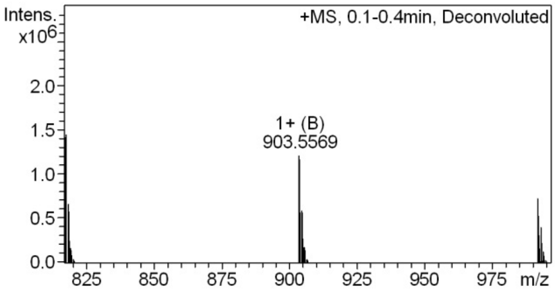


| # | m/z | Res. | S/N | I | I % |
|---|---|---|---|---|---|
| 1 | 452.2837 | 13164 | 3739.0 | 457872 | 31.5 |
| 2 | 452.7837 | 11117 | 1756.0 | 215543 | 14.8 |
| 3 | 496.2971 | 11425 | 1215.2 | 178158 | 12.3 |
| 4 | 817.5142 | 22337 | 5325.5 | 1452560 | 100.0 |
| 5 | 818.5122 | 18573 | 2330.5 | 638574 | 44.0 |
| 6 | 819.5089 | 13201 | 541.6 | 149164 | 10.3 |
| 7 | 903.5585 | 19329 | 2069.9 | 784119 | 54.0 |
| 8 | 904.5576 | 16870 | 1027.2 | 390455 | 26.9 |
| 9 | 991.5870 | 18978 | 1823.4 | 565607 | 38.9 |
| 10 | 992.5866 | 16639 | 1003.5 | 309549 | 21.3 |

| # | m/z | Res. | S/N | I | I % |
|---|---|---|---|---|---|
| 1 | 817.5113 | | | 1584514 | 100.0 |
| 2 | 903.5569 | | | 1241990 | 78.4 |
| 3 | 991.5848 | | | 743764 | 46.9 |

Bruker Compass DataAnalysis 4.0 printed: 9/4/2023 9:20:29 PM Page 1 of 1

**Figure S18.** HRMS-ESI of **6**.

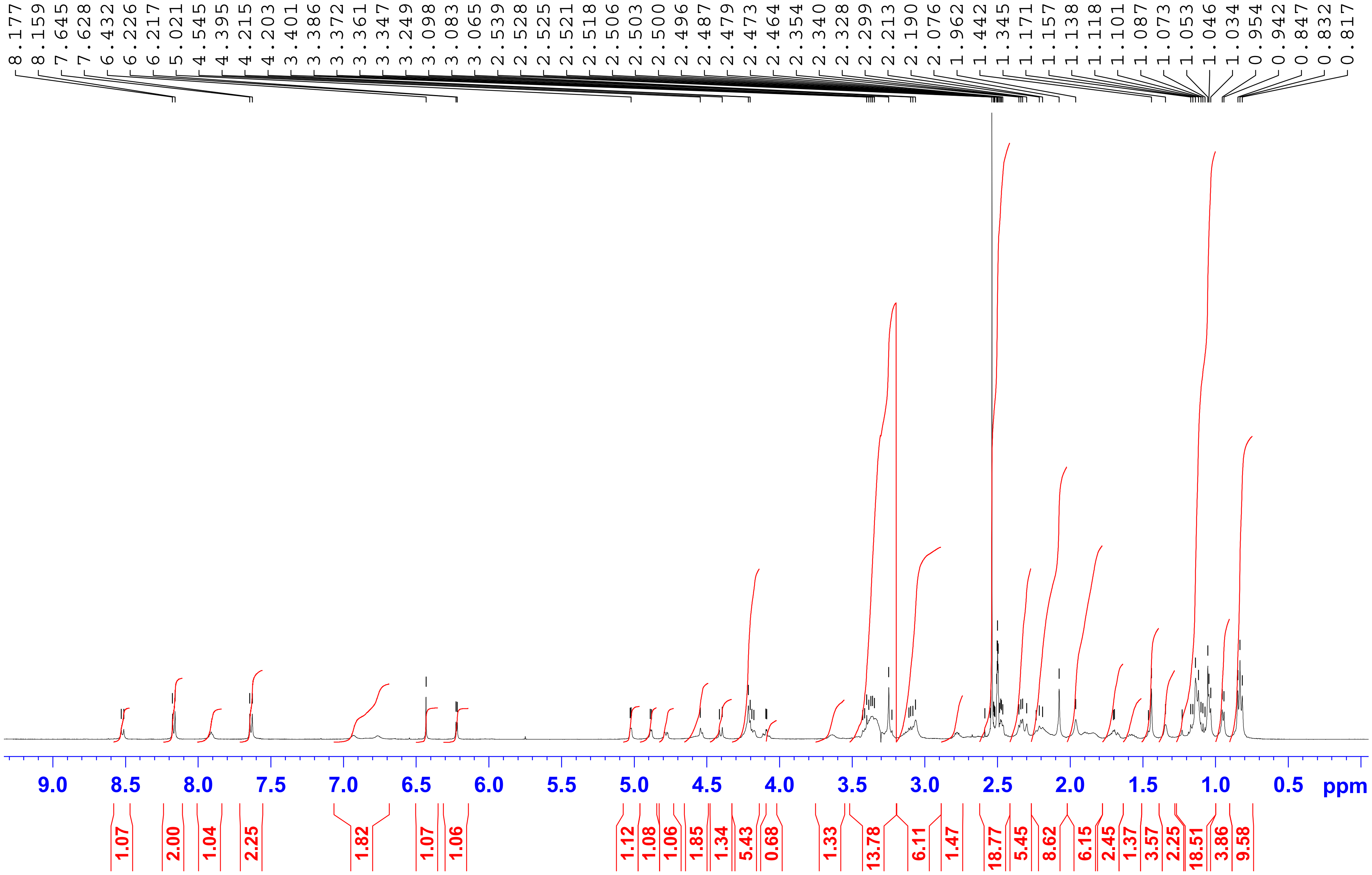

**Figure S19.** $^{1}$H NMR spectra of **4a** (500 MHz, DMSO-$d_6$).

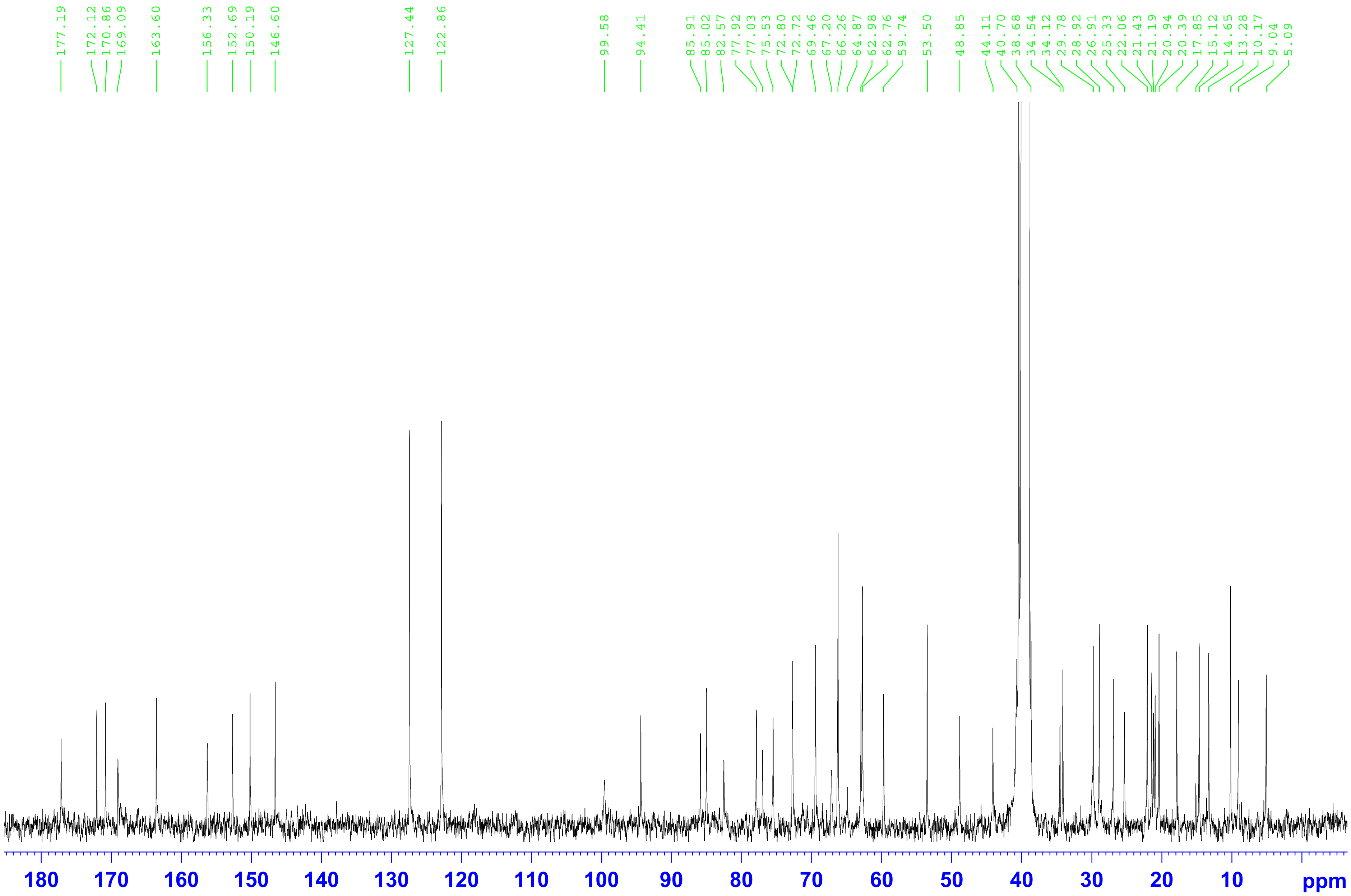


**Figure S20.** $^{13}C$ NMR spectra of **4a** (125 MHz, DMSO-*d*$_6$).

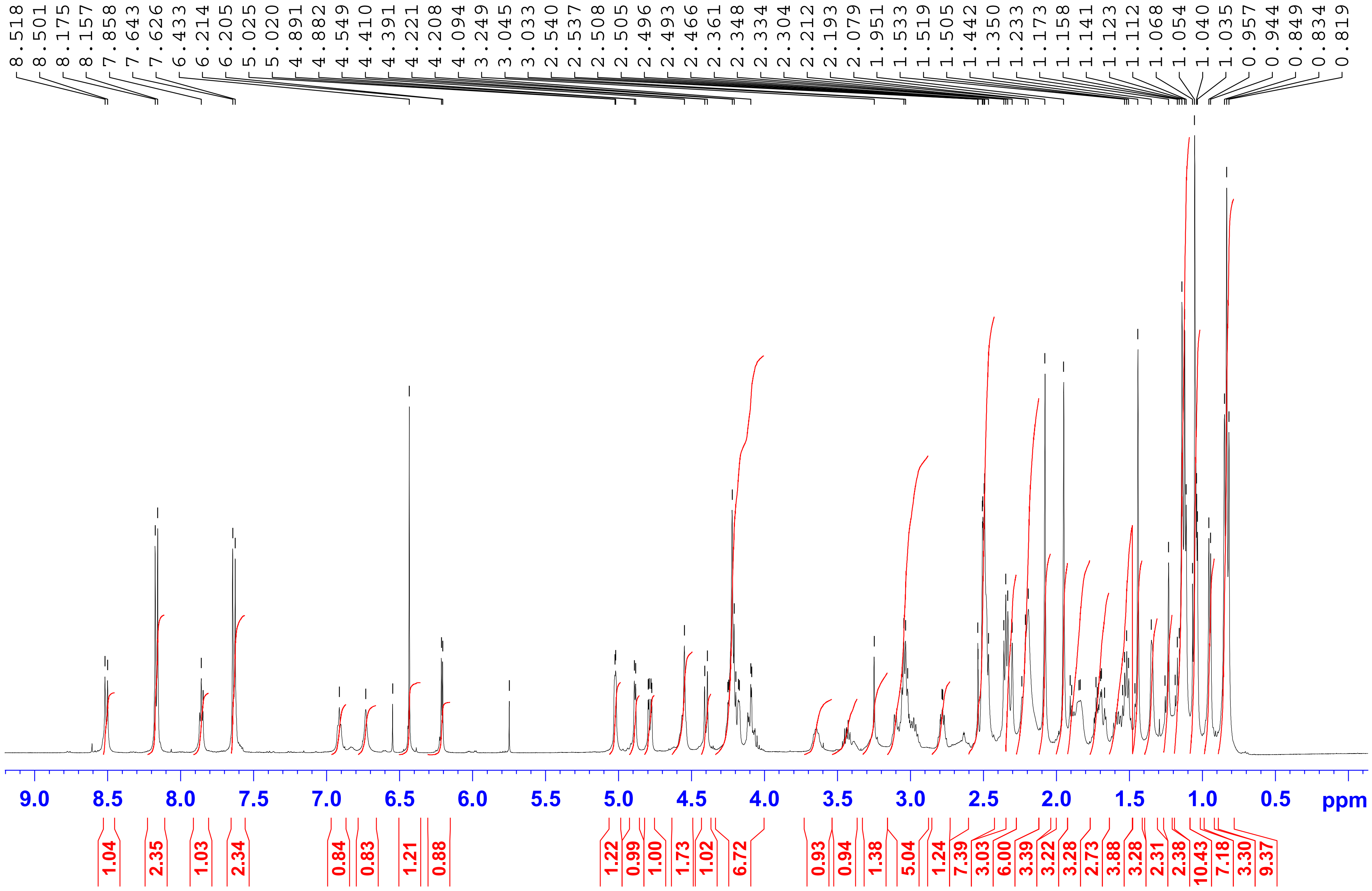

**Figure S21.** $^{1}H$ NMR spectra of **4b** (500 MHz, DMSO-*d*$_6$).

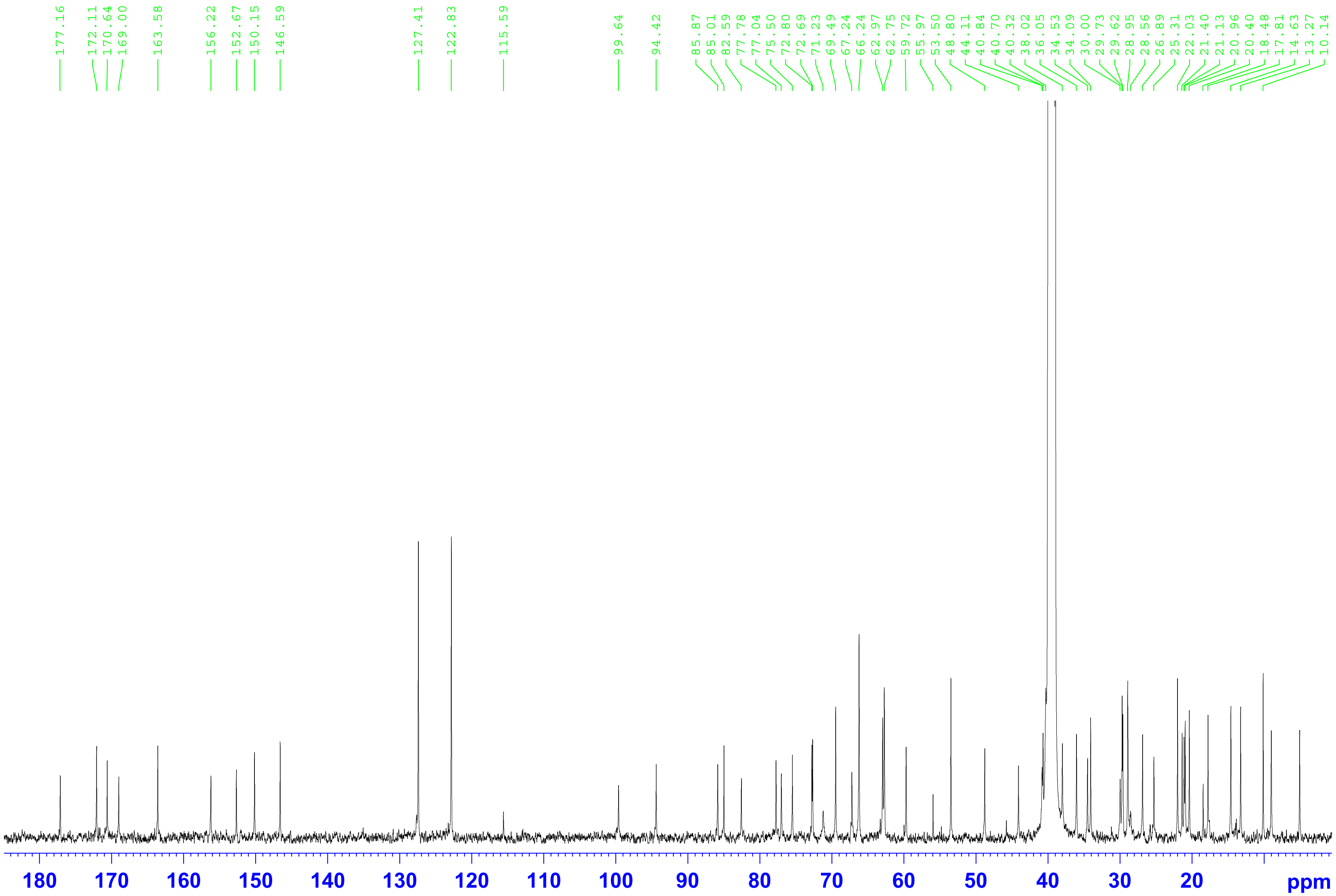


**Figure S22.** $^{13}$C NMR spectra of **4b** (125 MHz, DMSO-$d_6$).

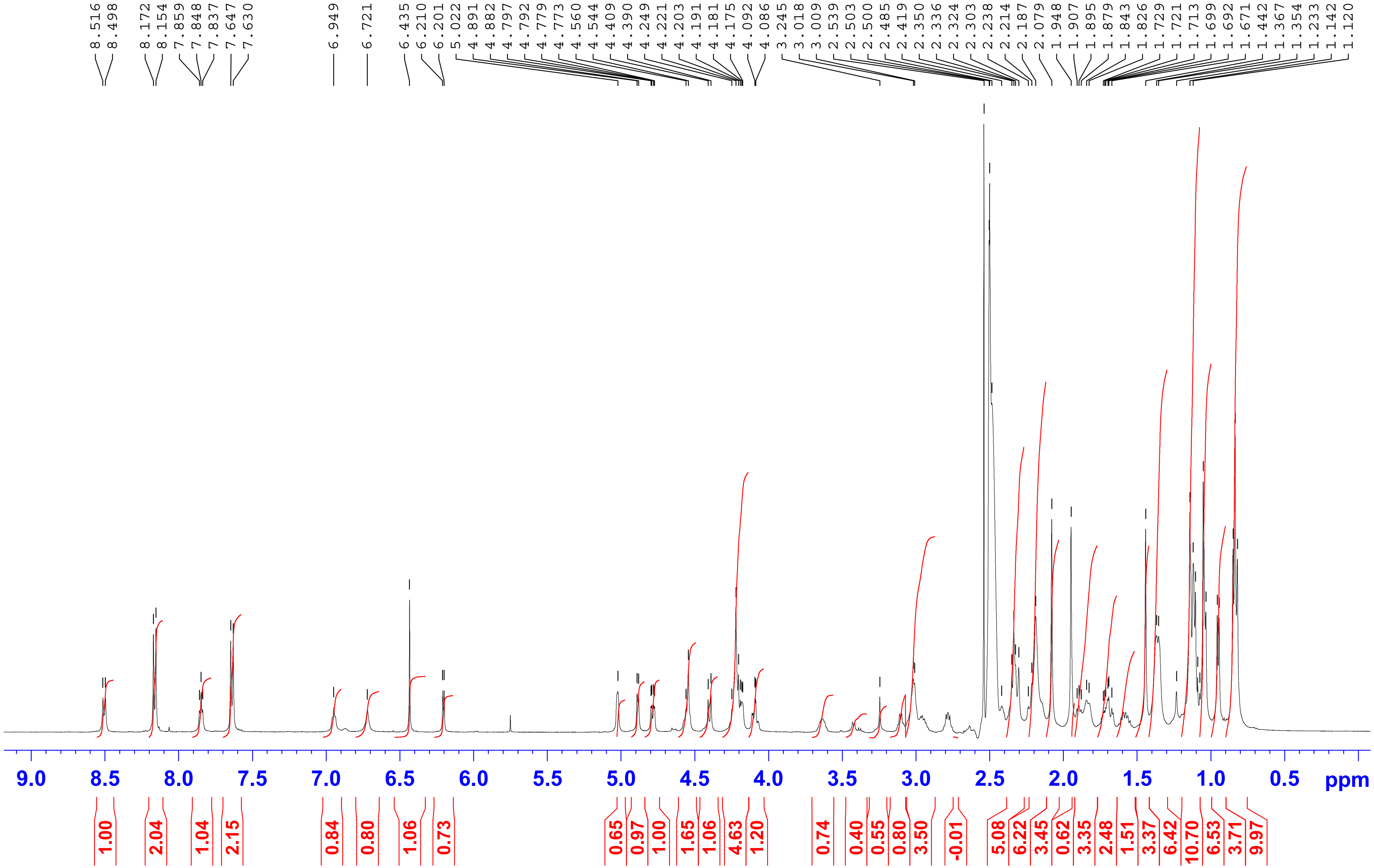


**Figure S23.** $^{1}$H NMR spectra of **4c** (500 MHz, DMSO-$d_6$).

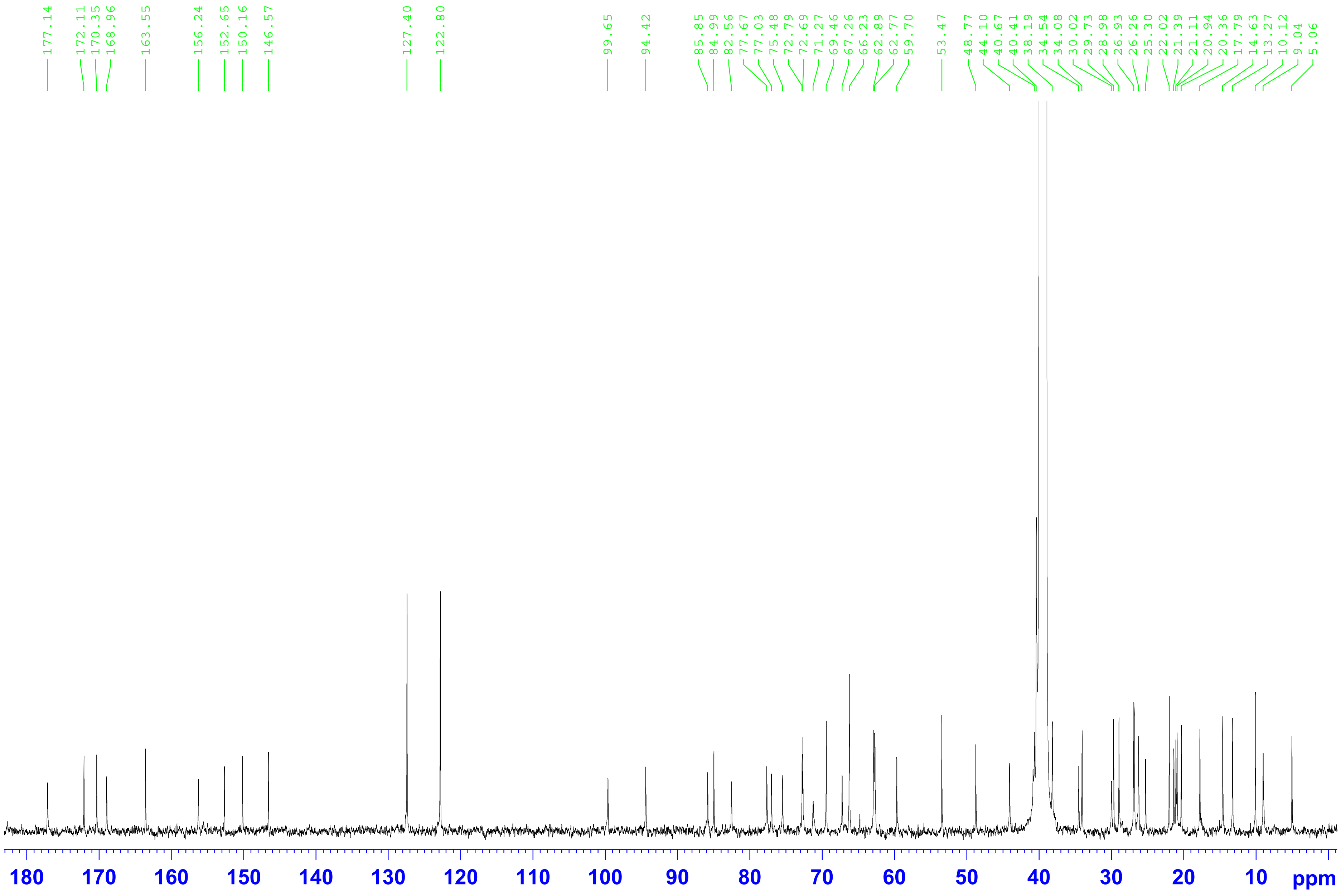


**Figure S24.** $^{13}C$ NMR spectra of **4c** (125 MHz, DMSO-$d_6$).

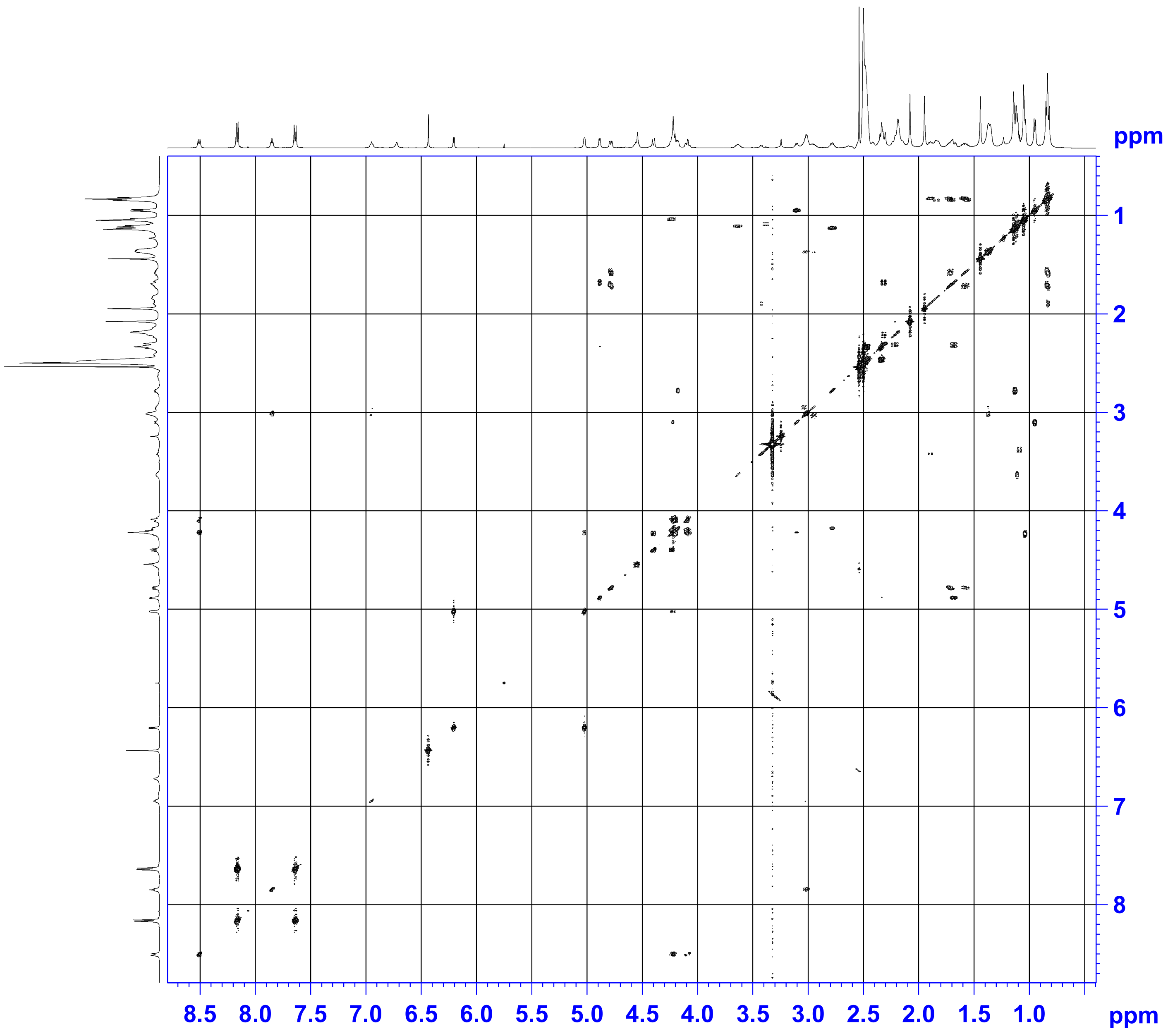


**Figure S25.** $^{1}H$-$^{1}H$ COSY NMR spectrum of **4c** (DMSO-$d_6$).

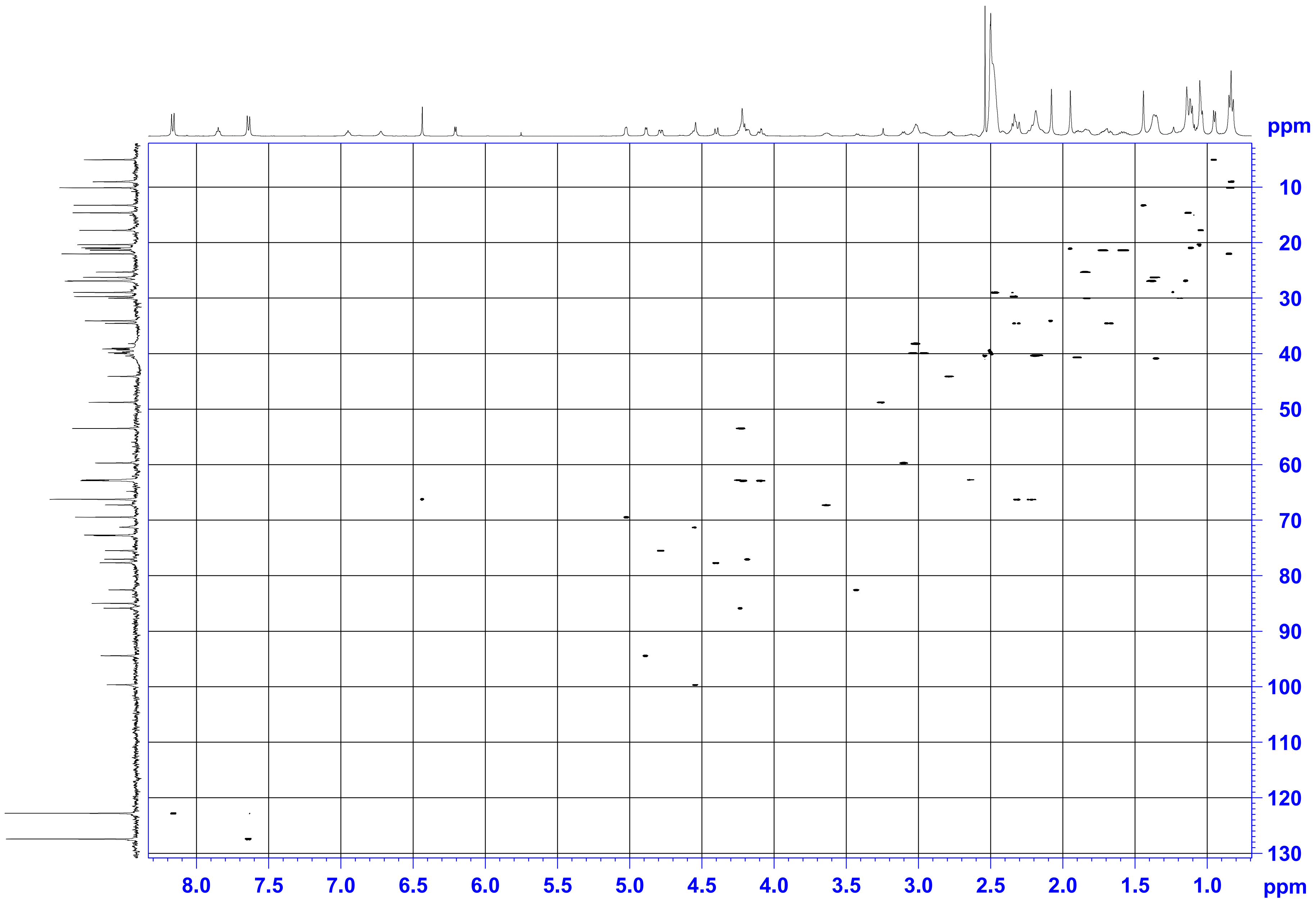


**Figure S26.** $^{1}H$-$^{13}C$ HSQC NMR spectrum of **4c** (DMSO-$d_6$).

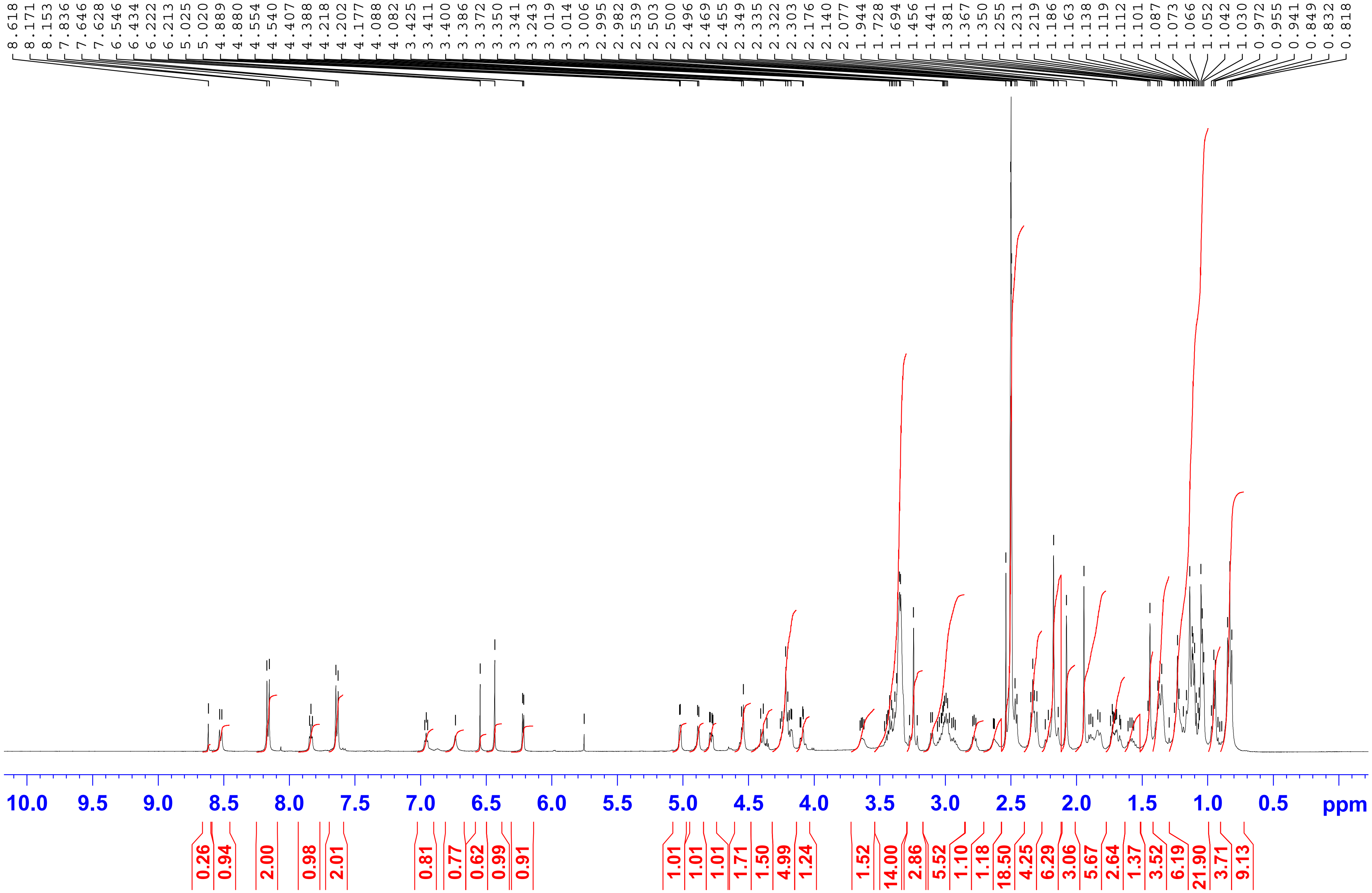

**Figure S27.** $^{1}$H NMR spectra of **4d** (500 MHz, DMSO-$d_6$).

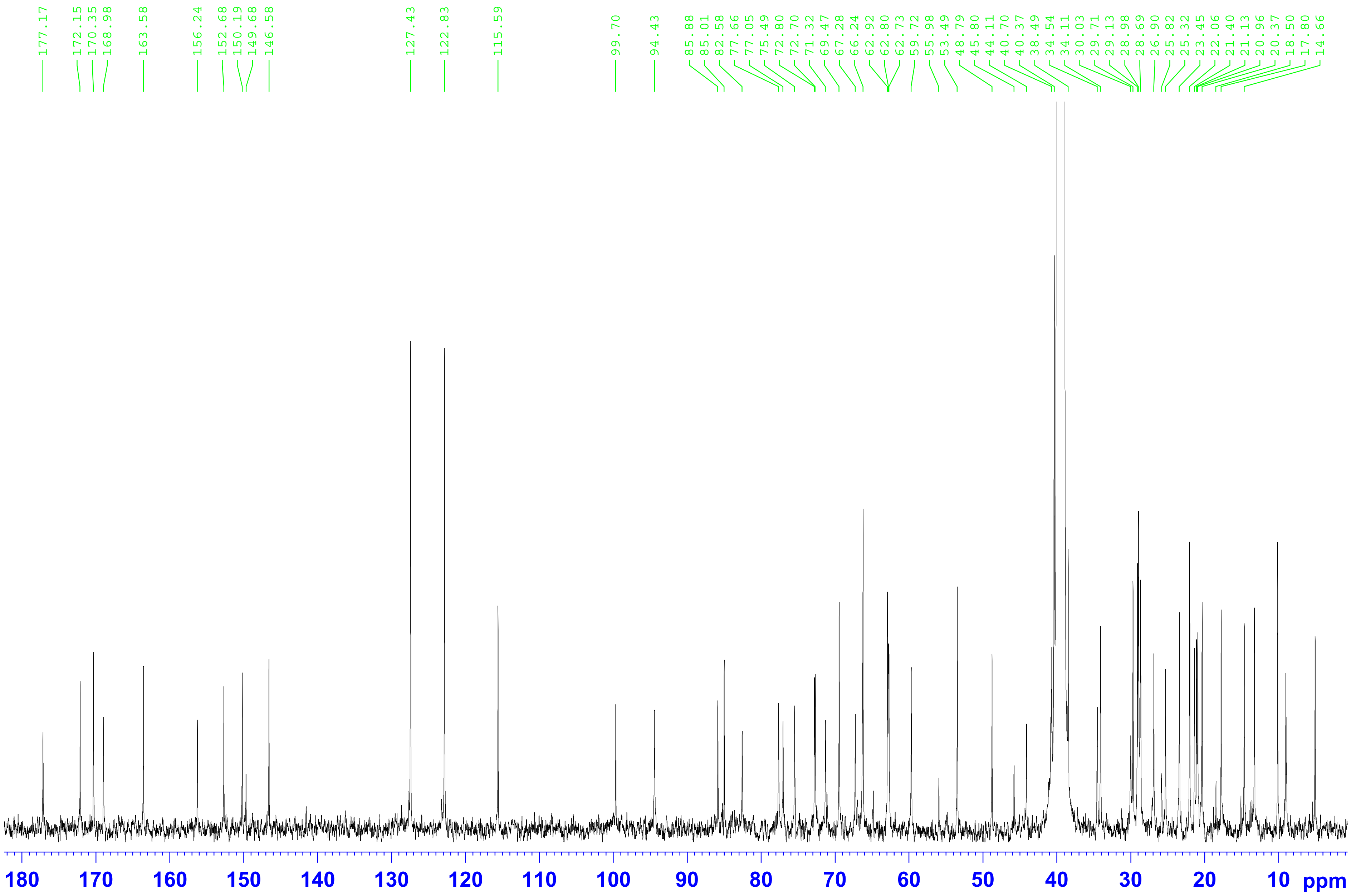


**Figure S28.** $^{13}C$ NMR spectra of **4d** (125 MHz, DMSO-*d*$_6$).

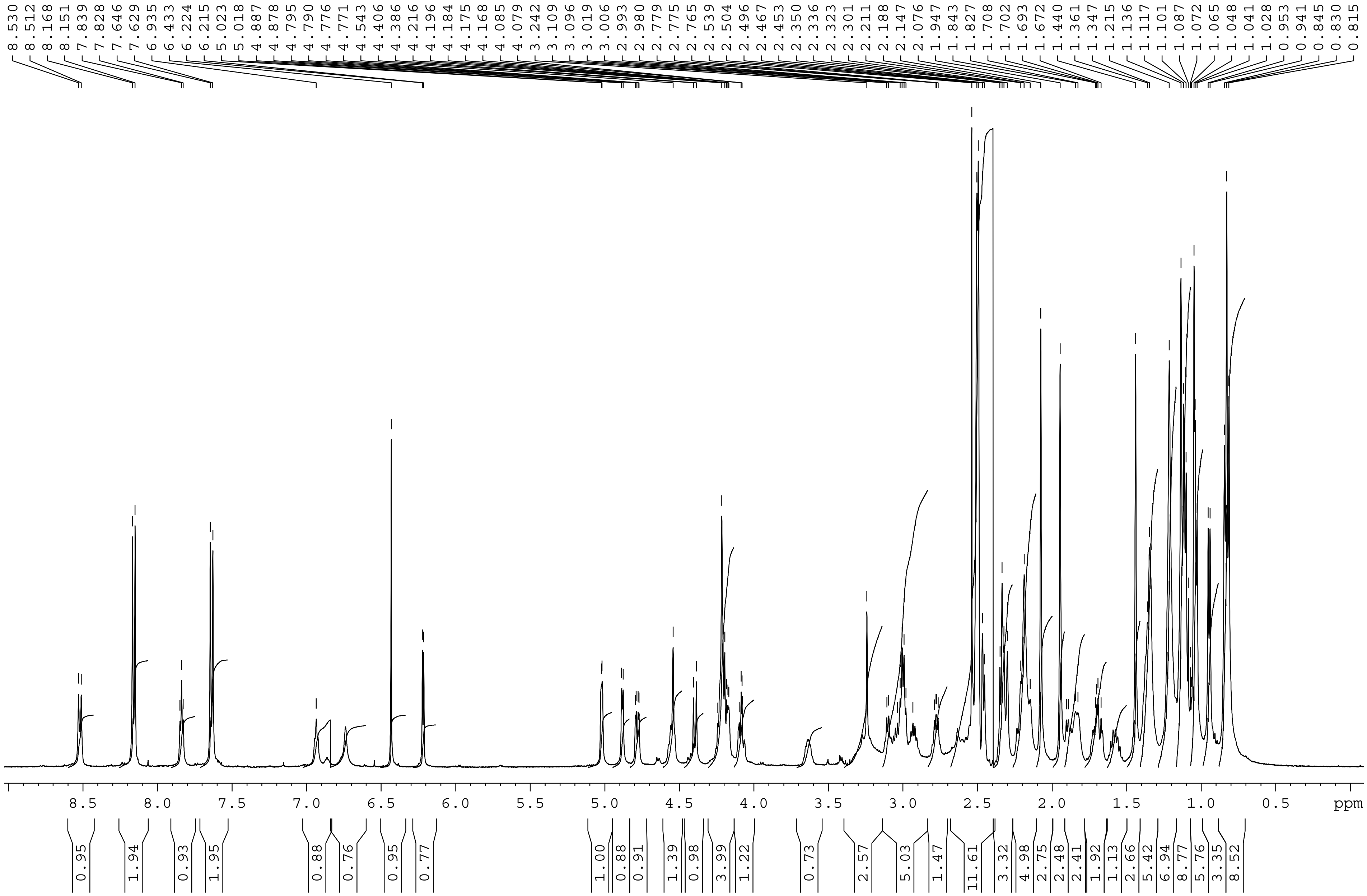

**Figure S29.** $^1$H NMR spectra of **4e** (500 MHz, DMSO-*d*$_6$).

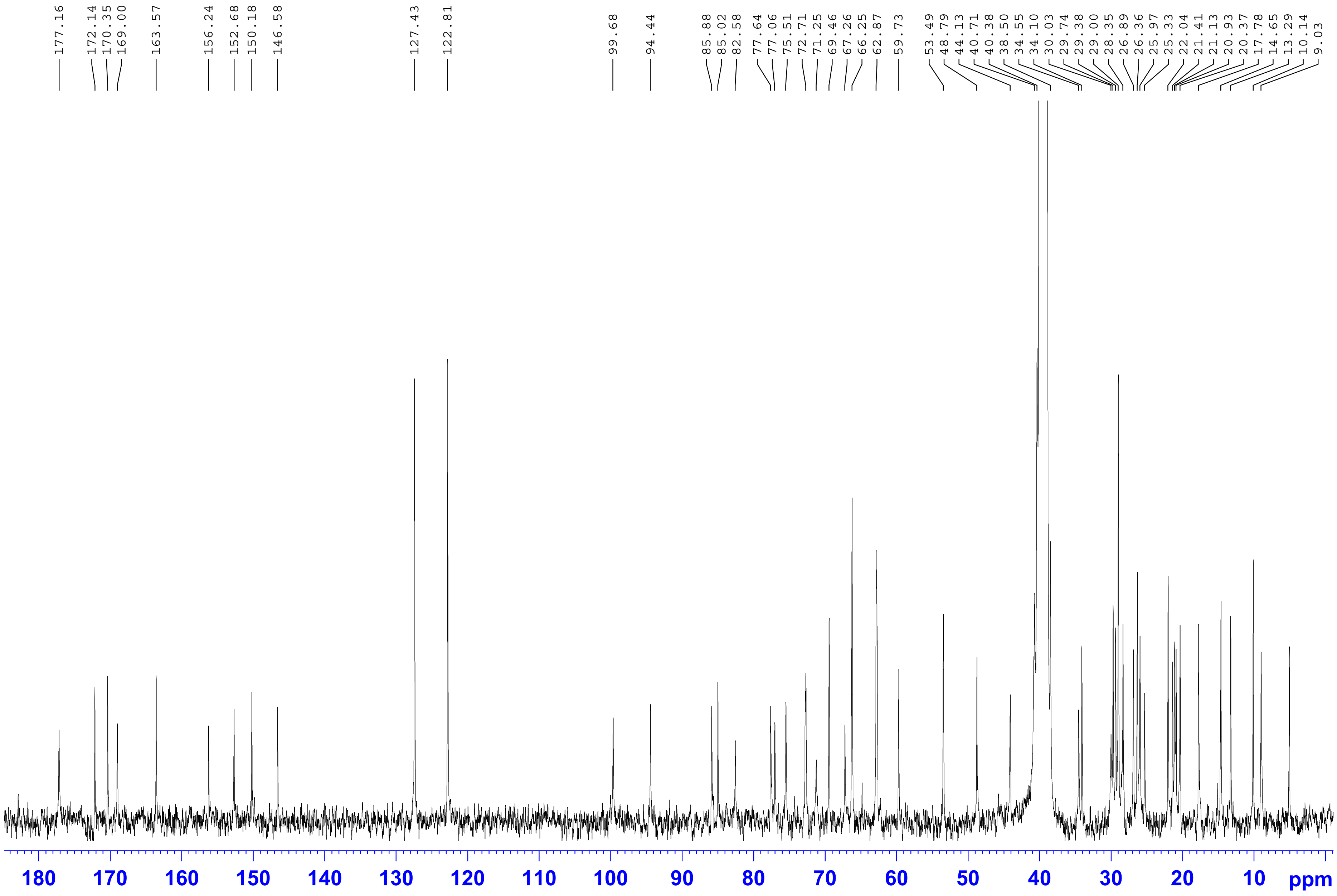


**Figure S30.** $^{13}C$ NMR spectra of **4e** (125 MHz, DMSO-*d*$_6$).

LCTA-3147, COSY.

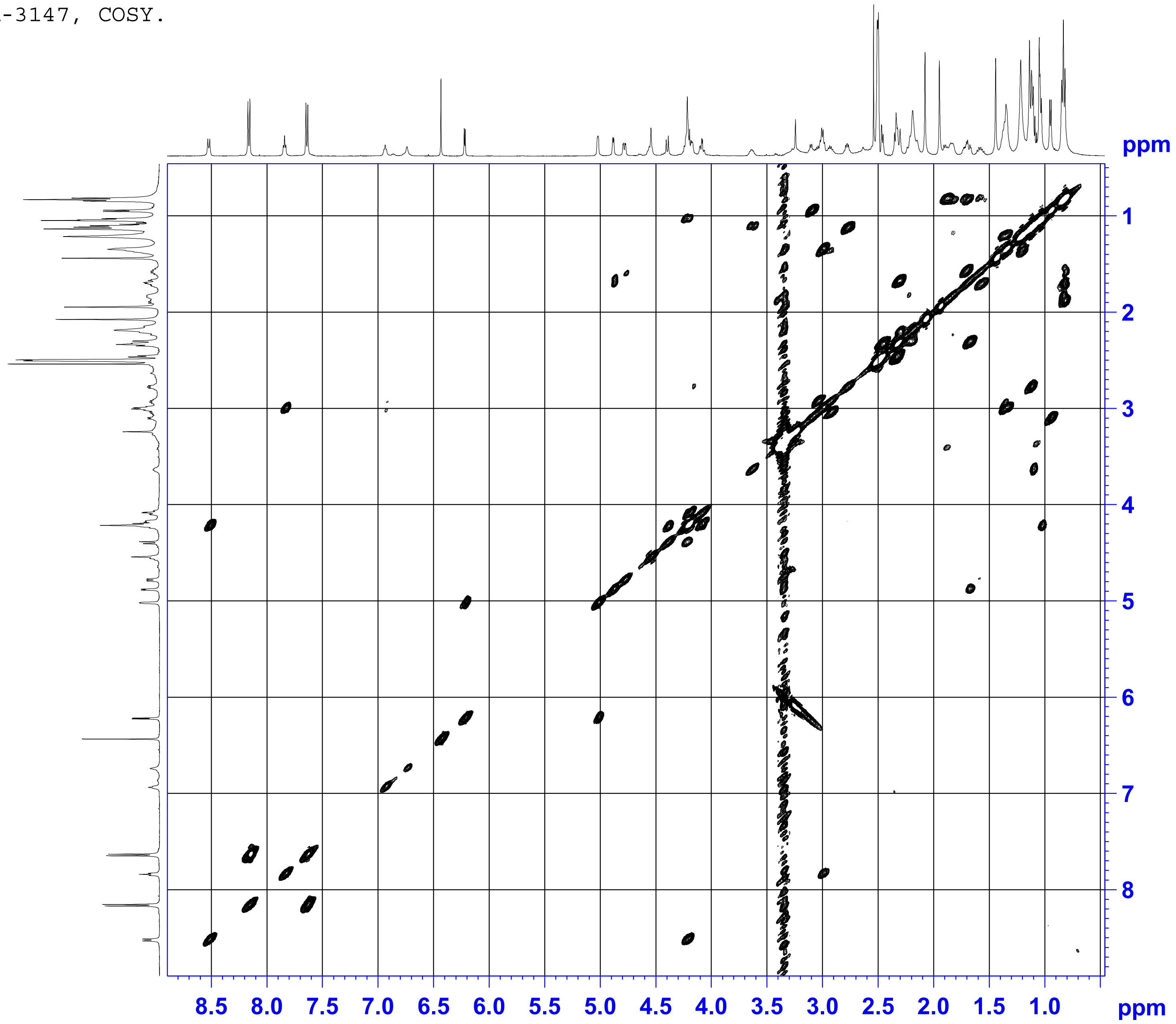


**Figure S31.** $^{1}$H-$^{1}$H COSY NMR spectrum of **4e** (DMSO-*d*₆).

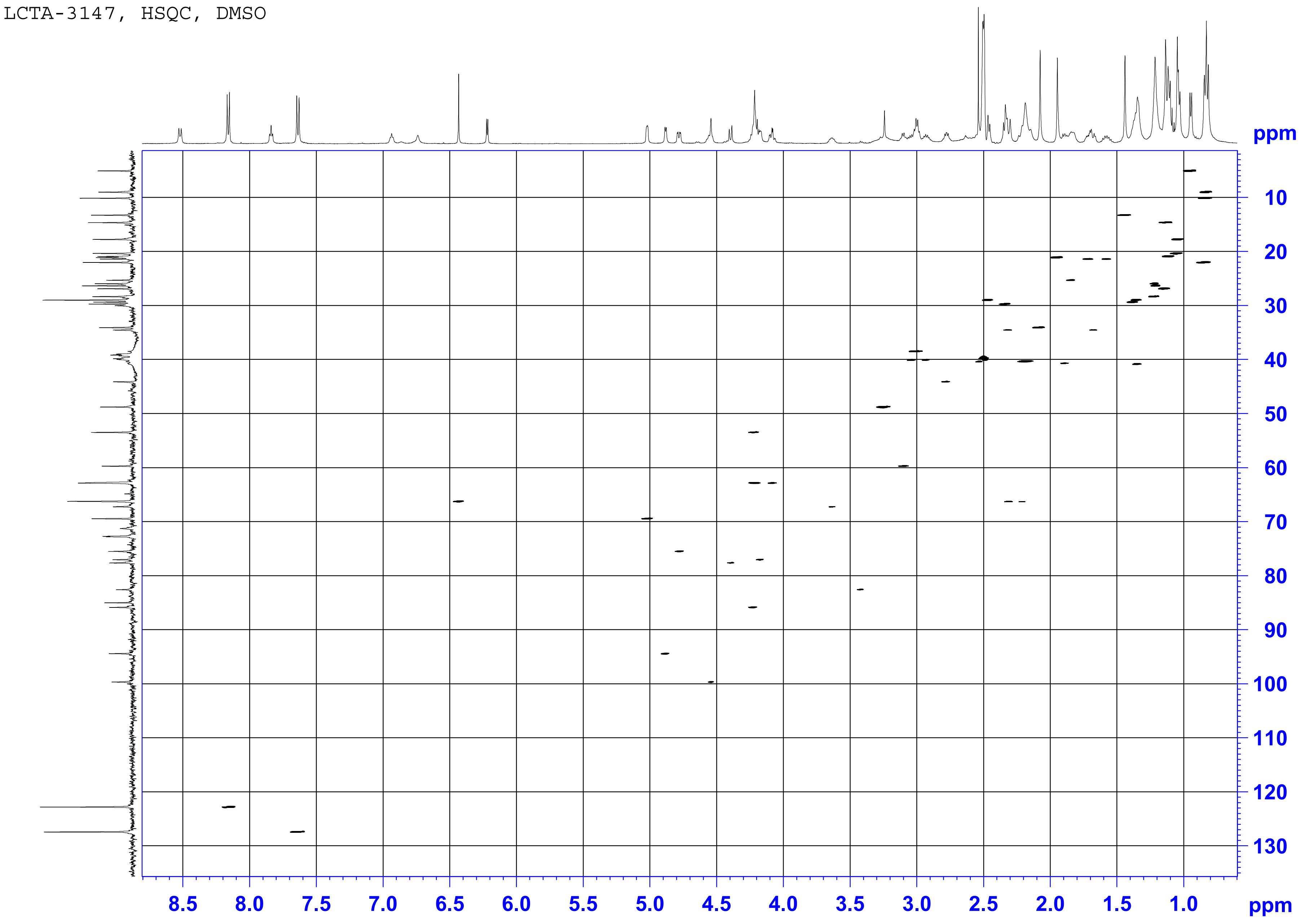


**Figure S32.** $^{1}H$-$^{13}C$ HSQC NMR spectrum of **4e** (DMSO-$d_6$).

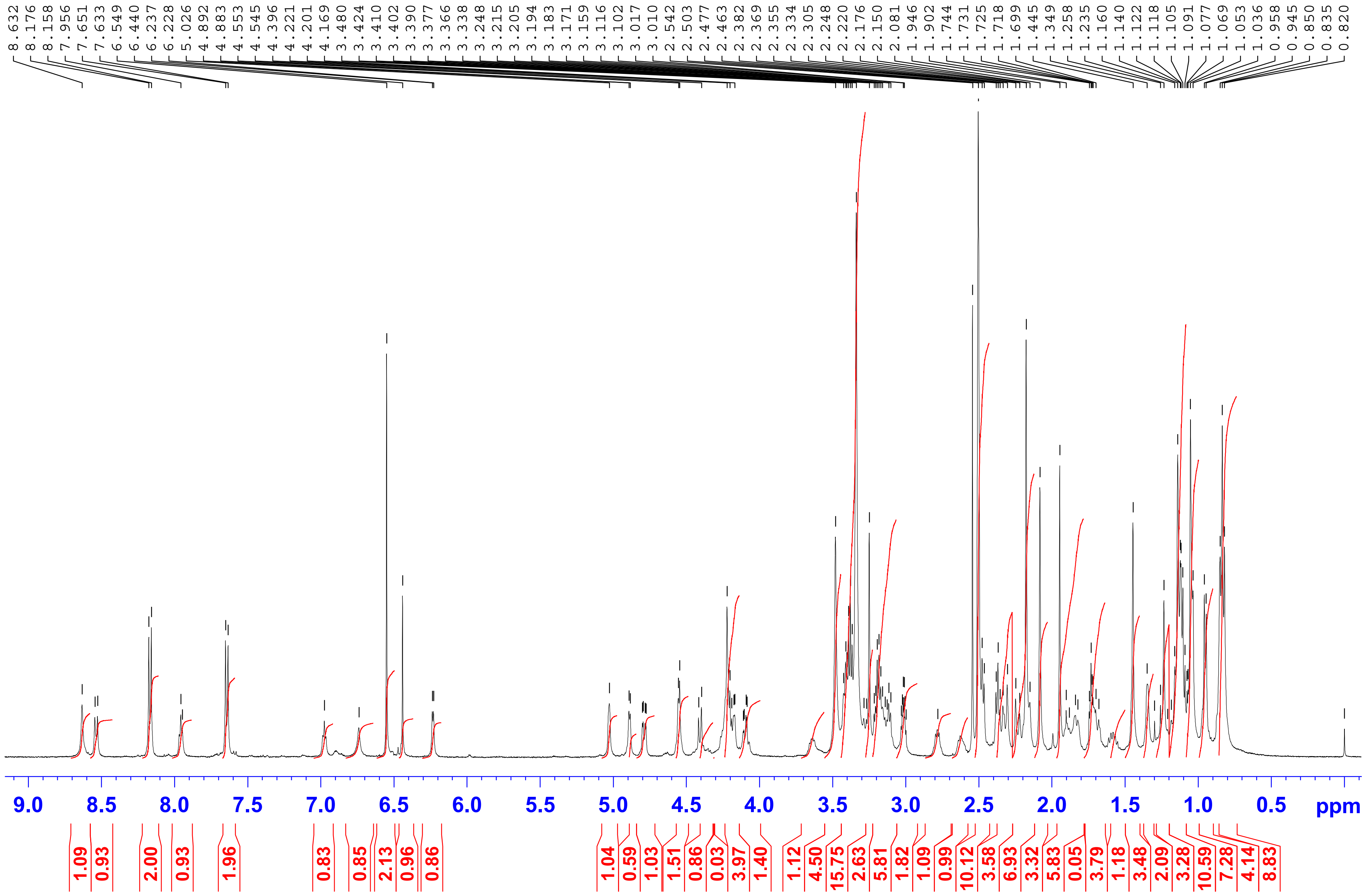

**Figure S33.** $^{1}$H NMR spectra of **4f** (500 MHz, DMSO-*d*6).

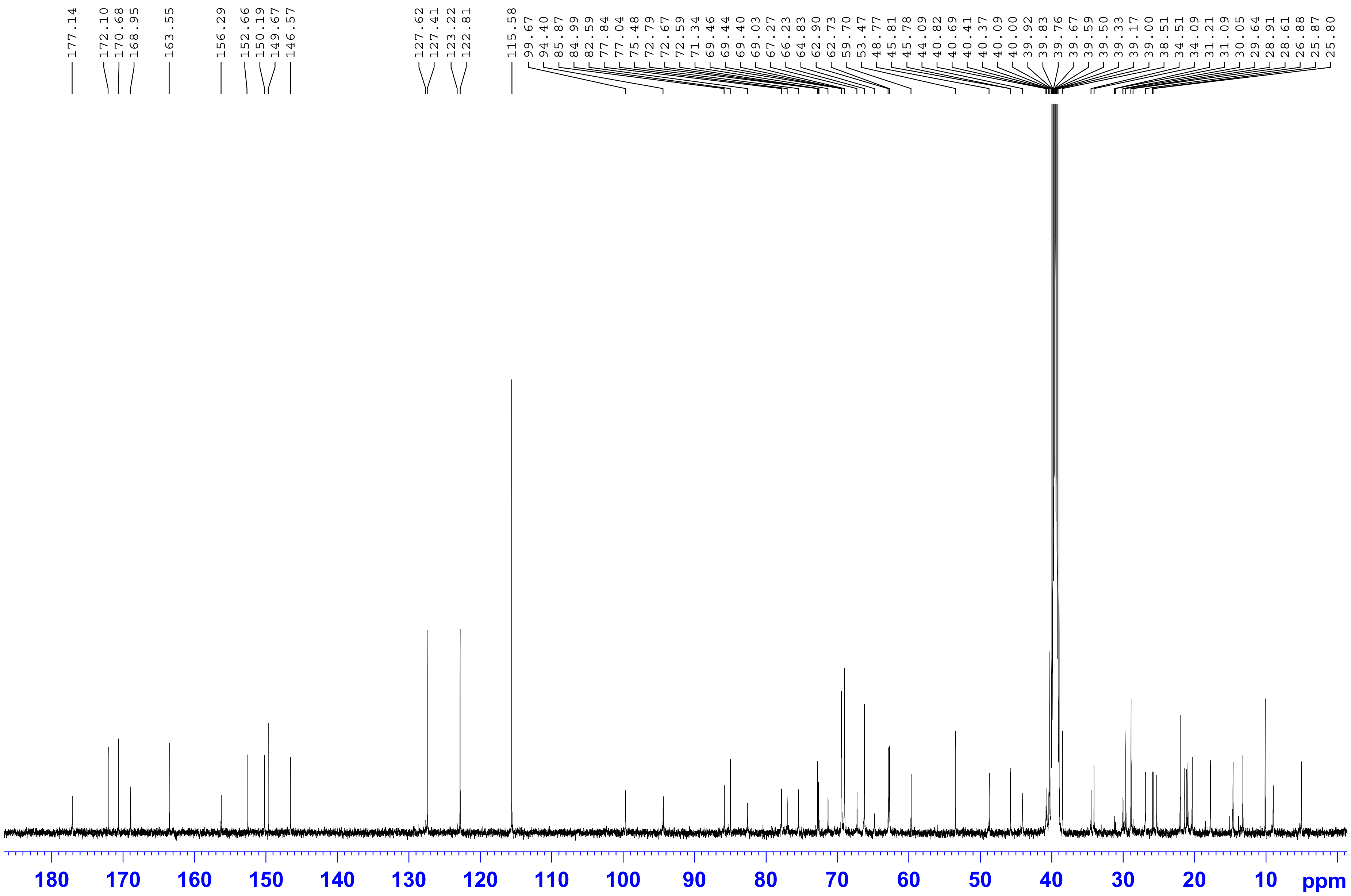


**Figure S34.** $^{13}C$ NMR spectra of **4f** (125 MHz, DMSO-$d_6$).

LCTA-3041 COSY

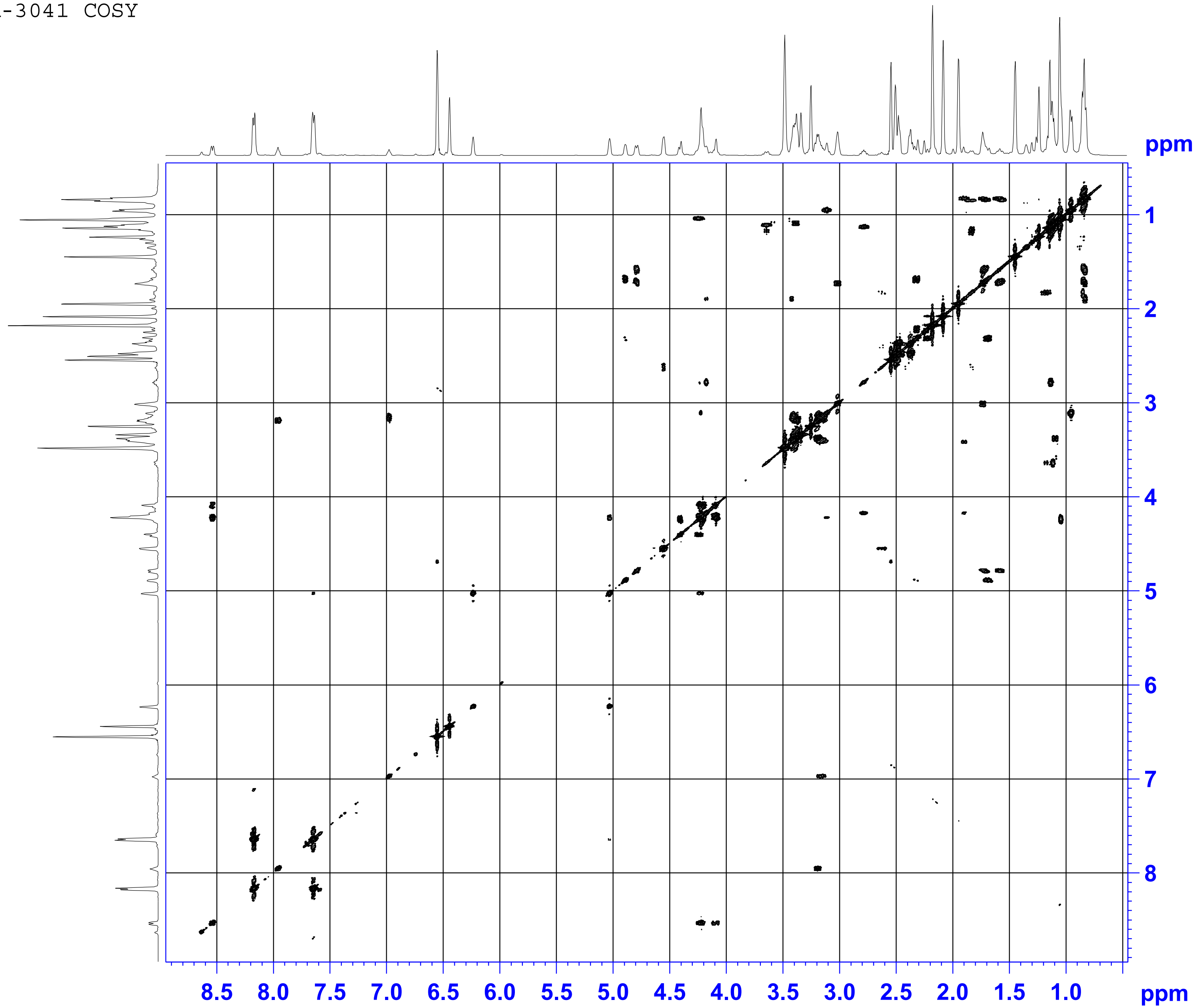


**Figure S35.** $^1$H-$^1$H COSY NMR spectrum of **4f** (DMSO-$d_6$).

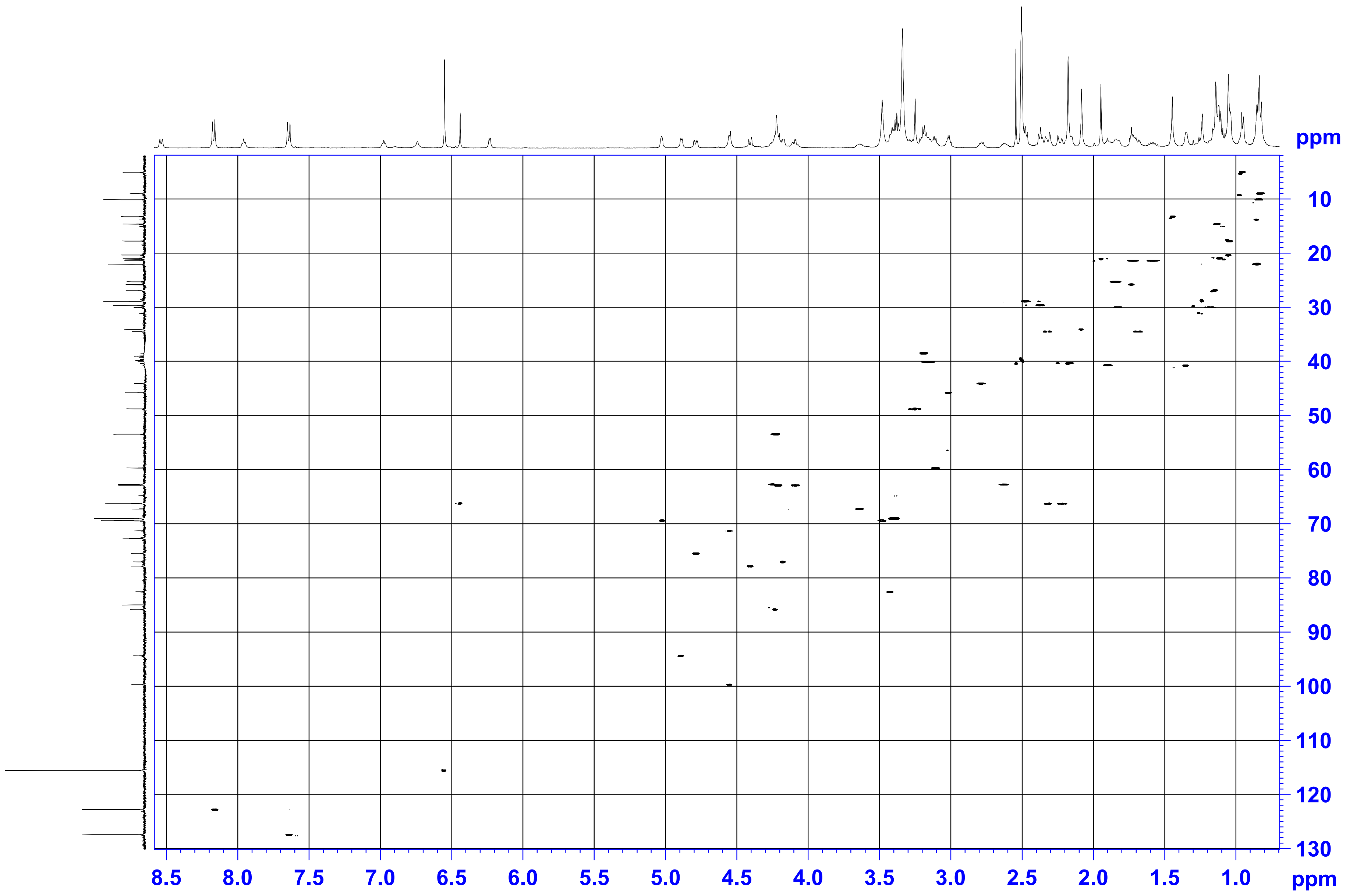


**Figure S36.** $^{1}H$-$^{13}C$ HSQC NMR spectrum of **4f** (DMSO-$d_6$).

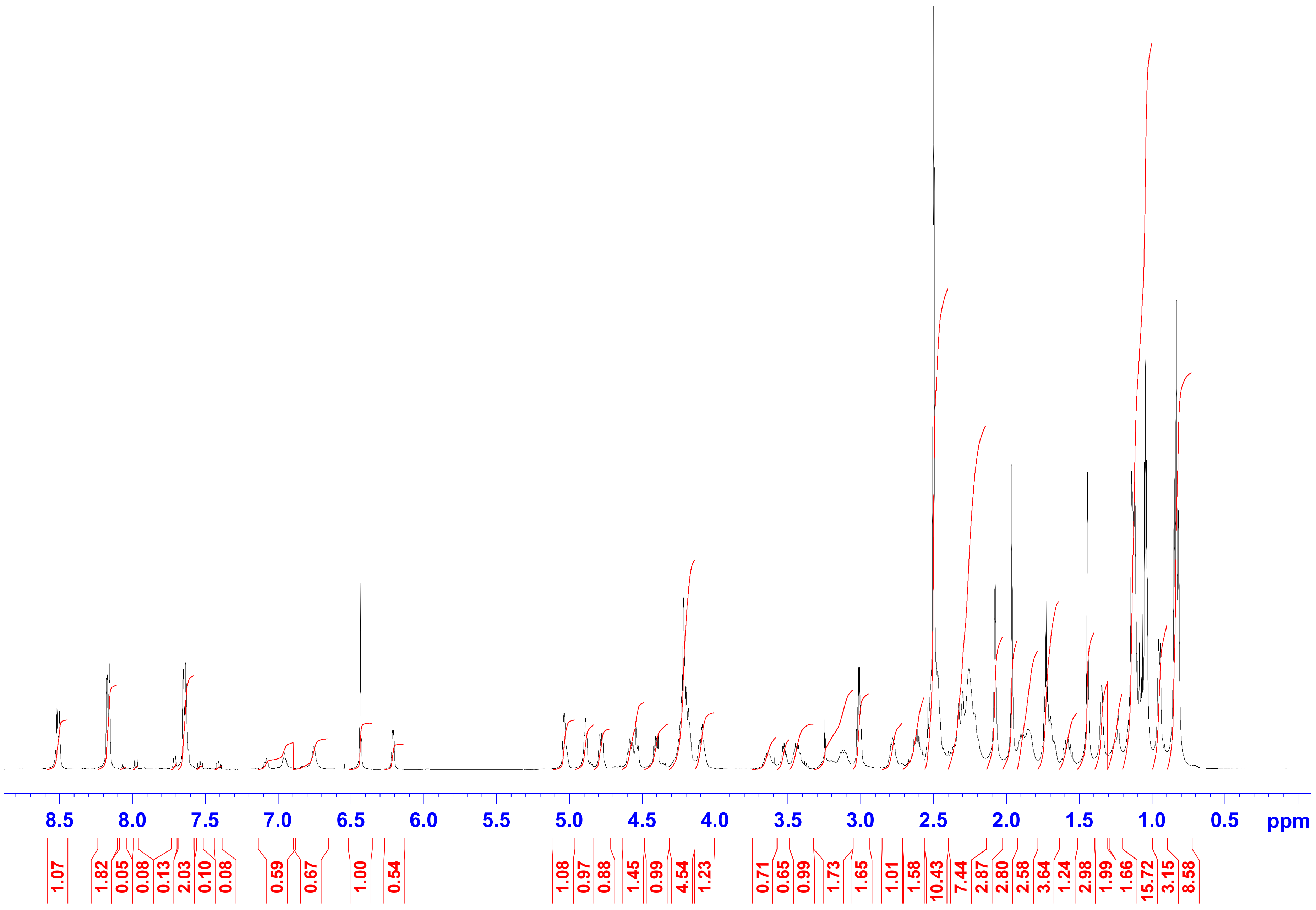


**Figure S37.** $^1$H NMR spectra of **4g** (500 MHz, DMSO-*d*$_6$).

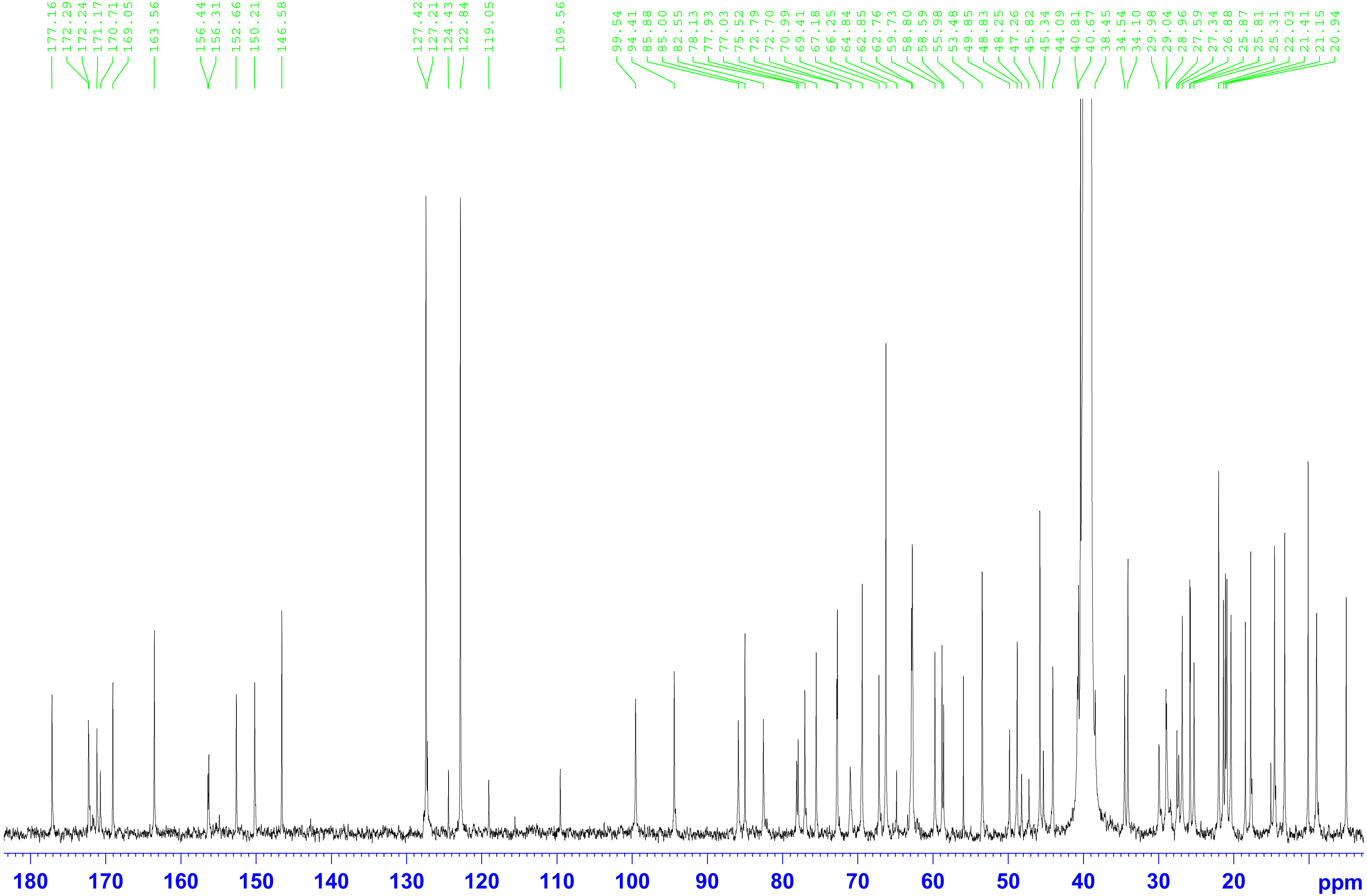


**Figure S38.** $^{13}C$ NMR spectra of **4g** (125 MHz, DMSO-$d_6$).

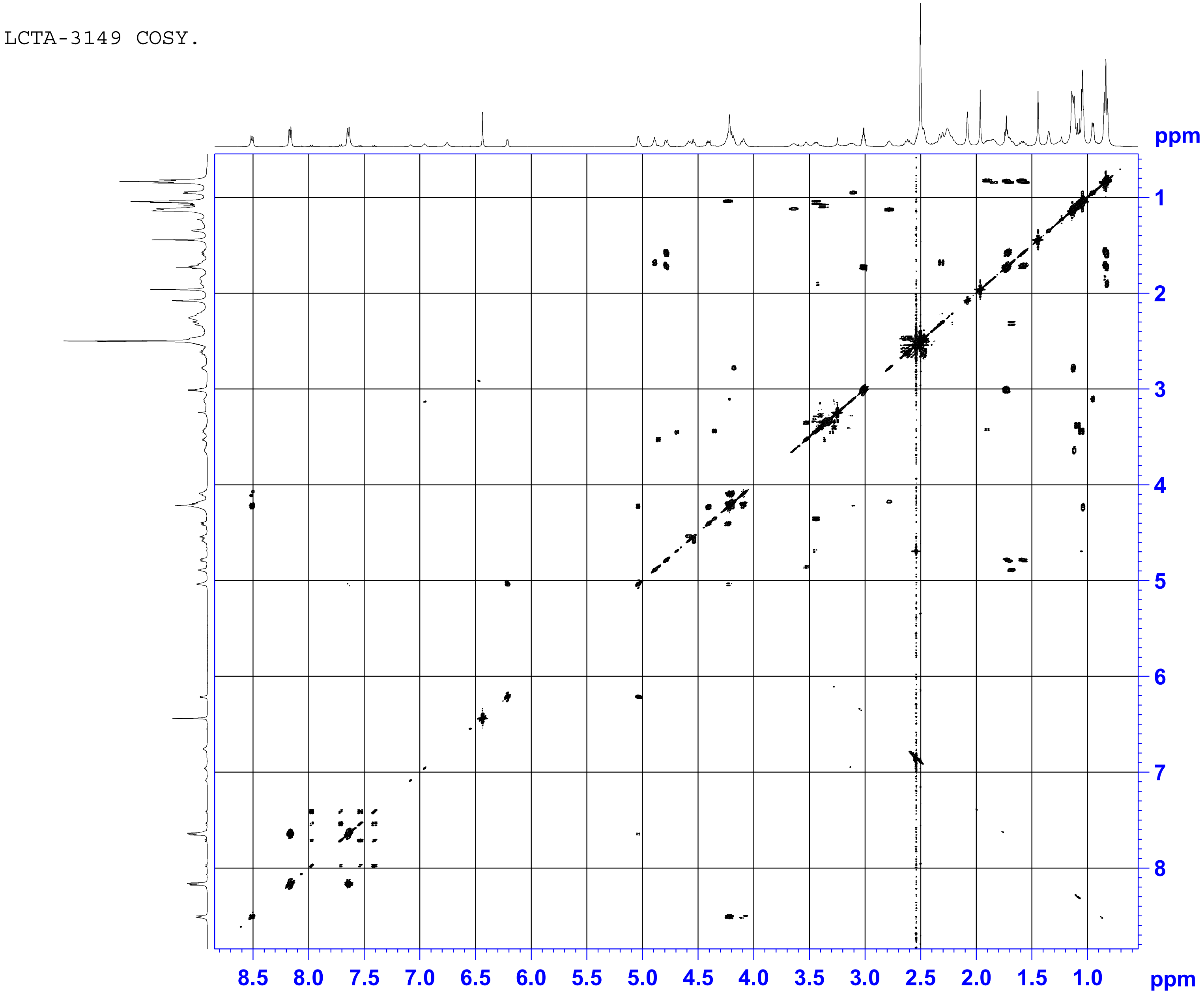


**Figure S39.** $^1H$-$^1H$ COSY NMR spectrum of **4g** (DMSO-*d*$_6$).

LCTA-3149 HSQC.

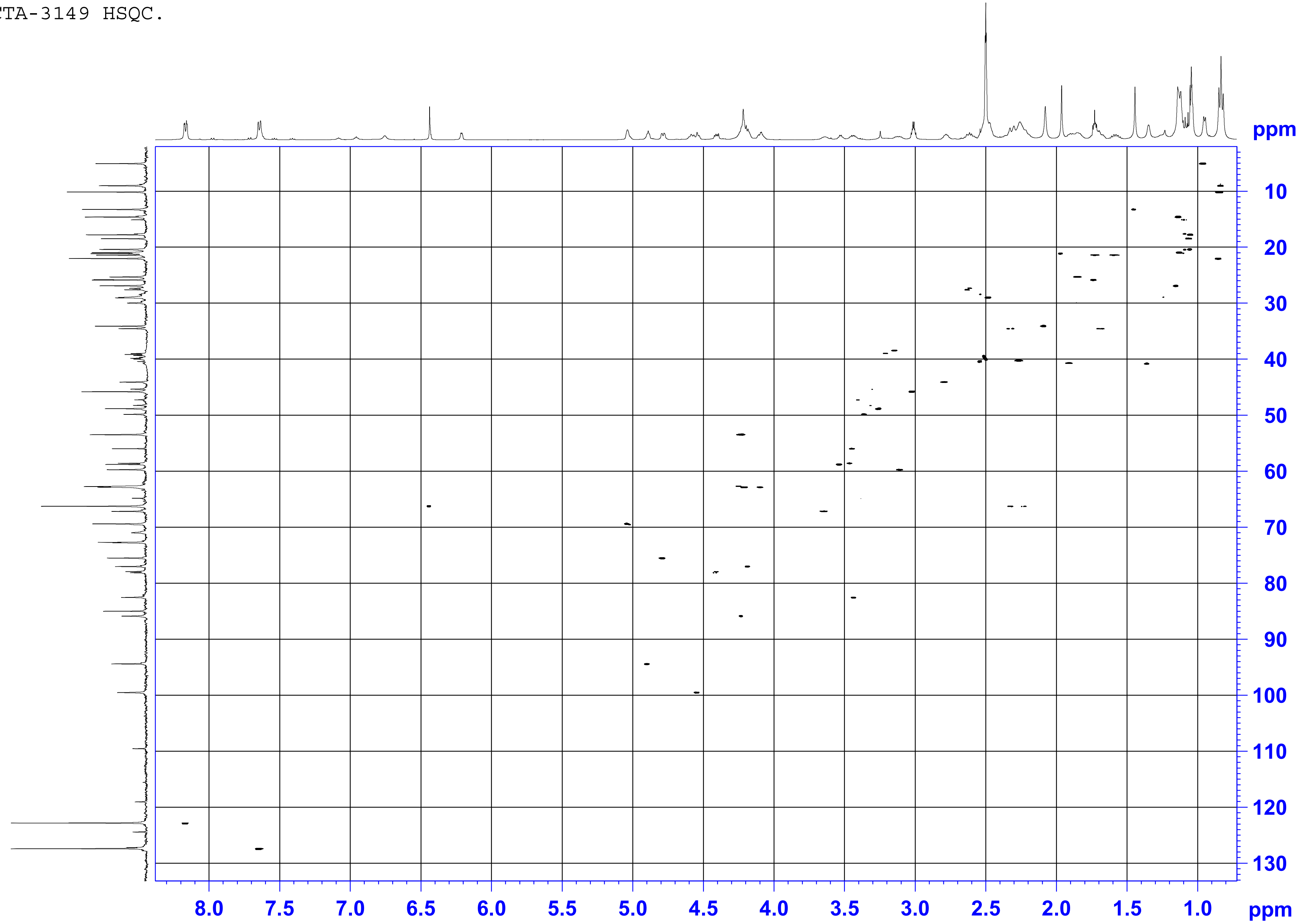


**Figure S40.** $^{1}$H-$^{13}$C HSQC NMR spectrum of **4g** (DMSO-*d*$_6$).

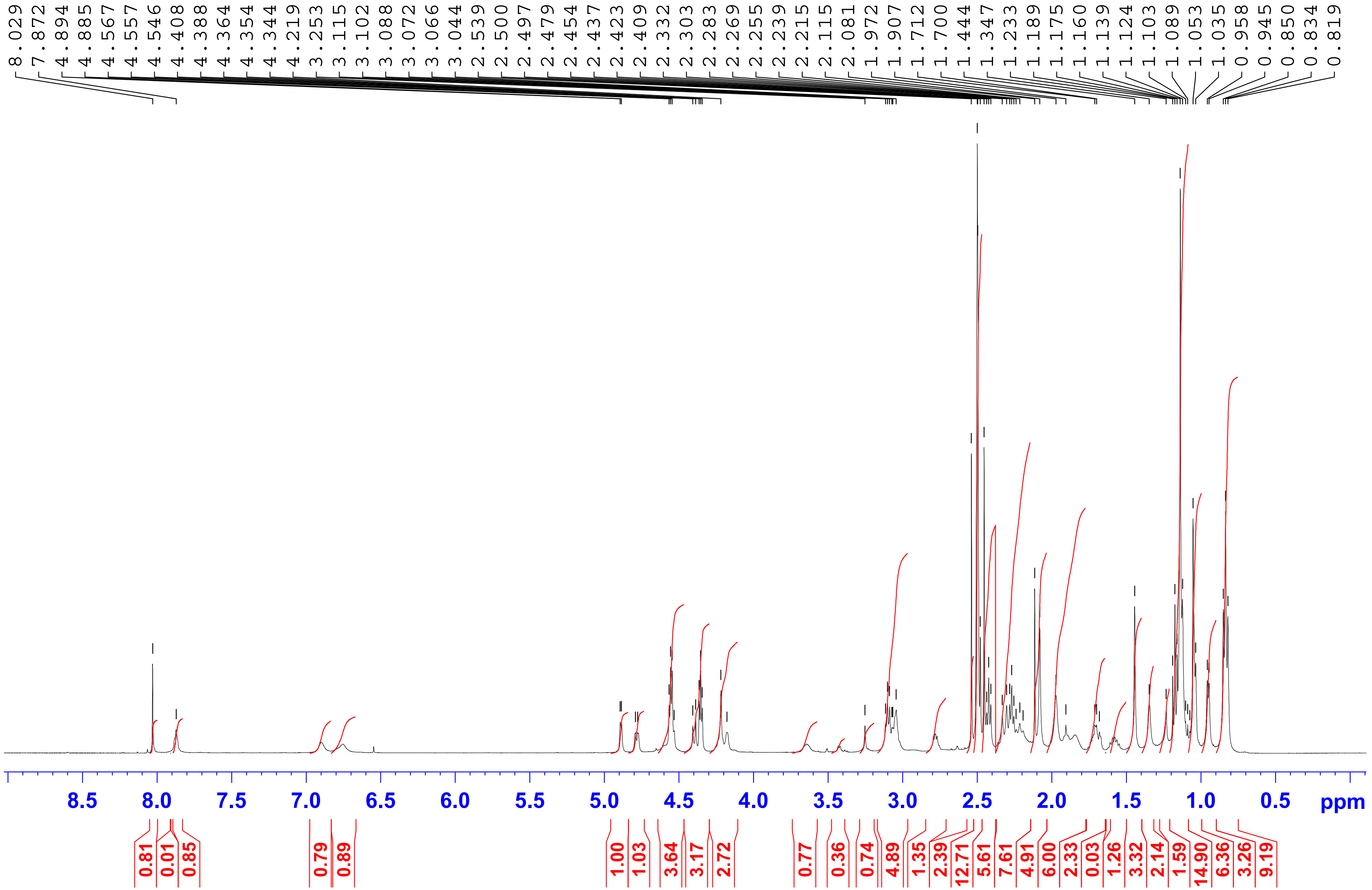

**Figure S41.** $^{1}$H NMR spectra of **5a** (500 MHz, DMSO-$d_6$).

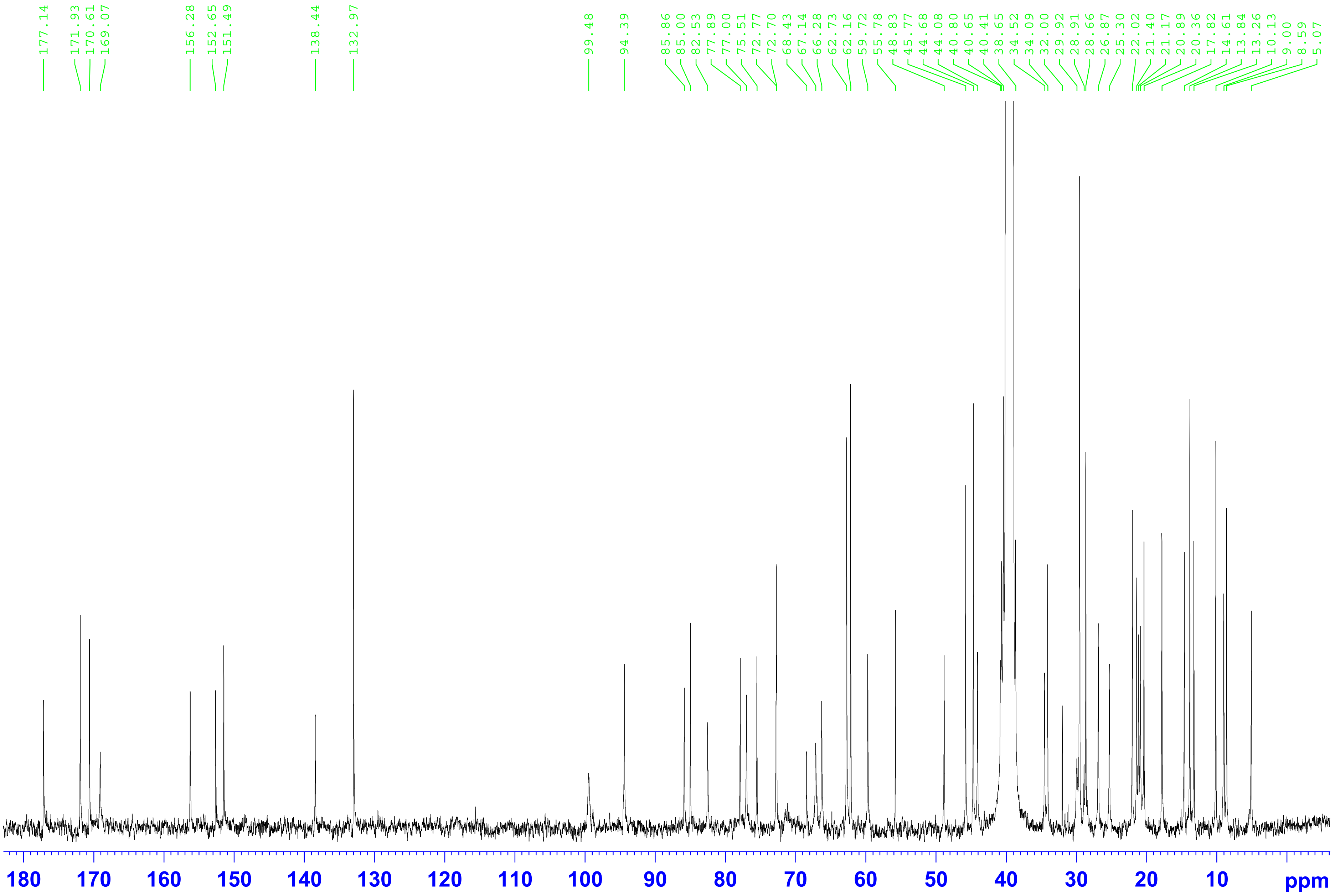


**Figure S42.** $^{13}$C NMR spectra of **5a** (125 MHz, DMSO-*d*$_6$).

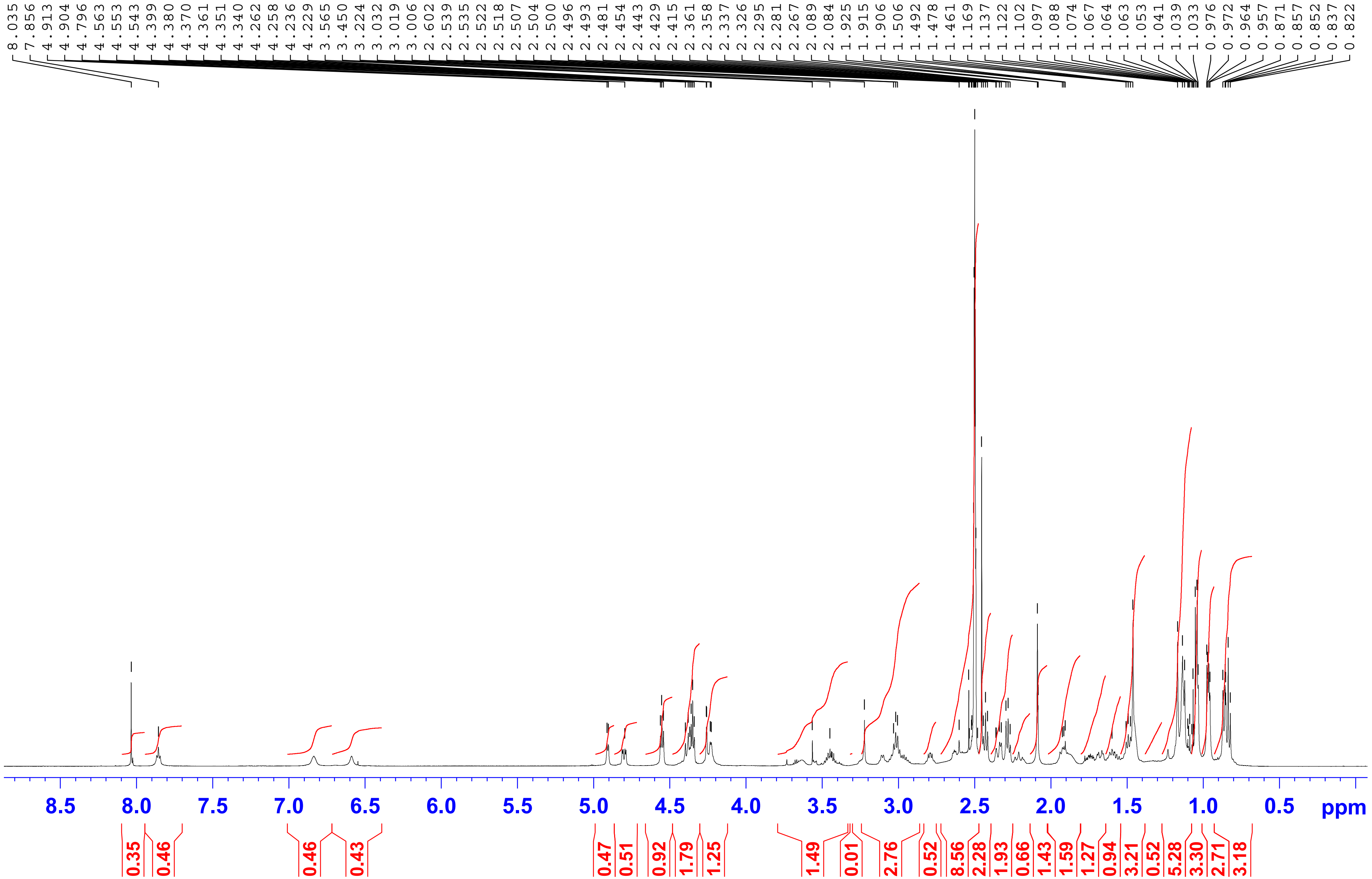


**Figure S43.** ¹H NMR spectra of **5b** (500 MHz, DMSO-$d_6$).

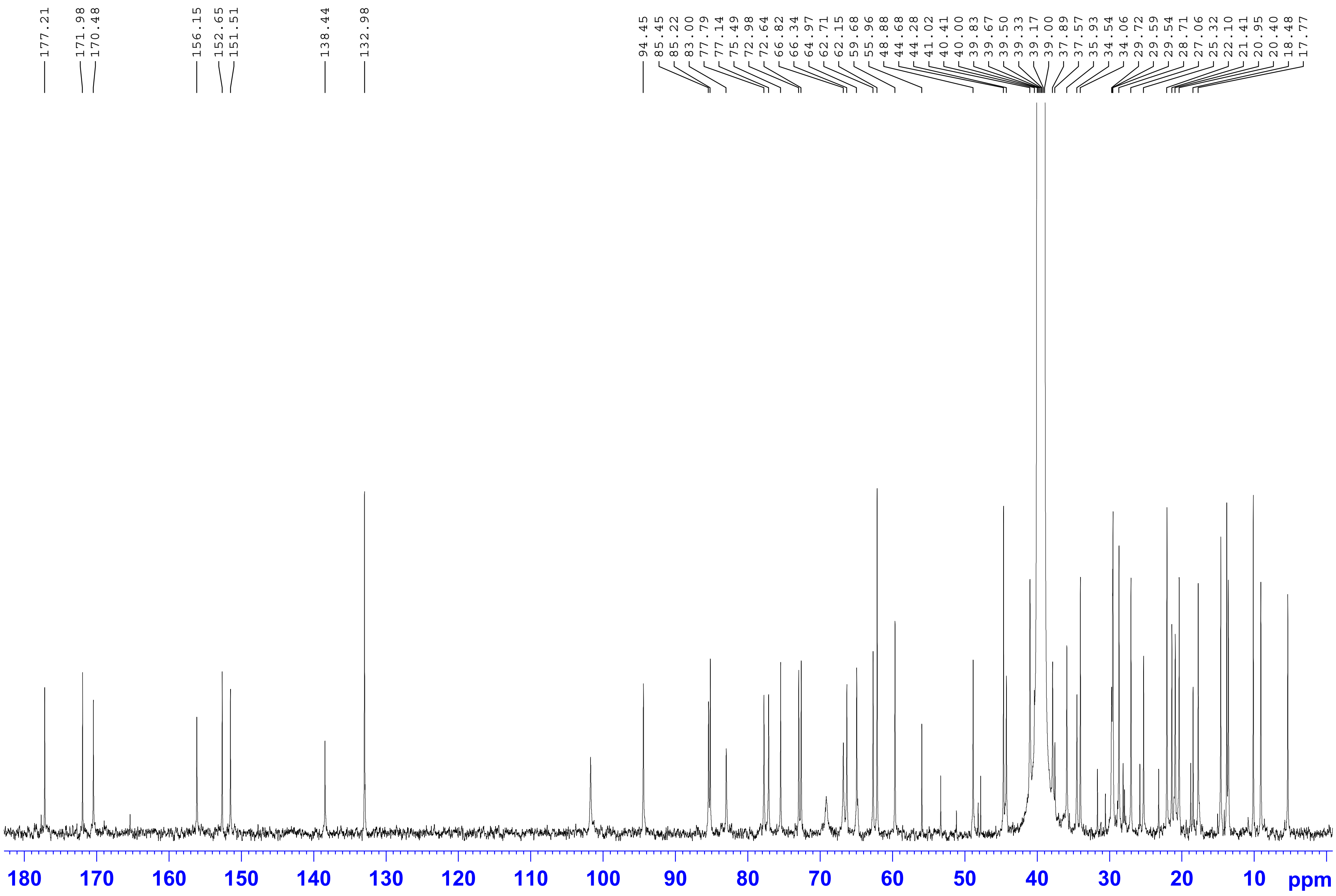


**Figure S44.** $^{13}C$ NMR spectra of **5b** (125 MHz, DMSO-$d_6$).

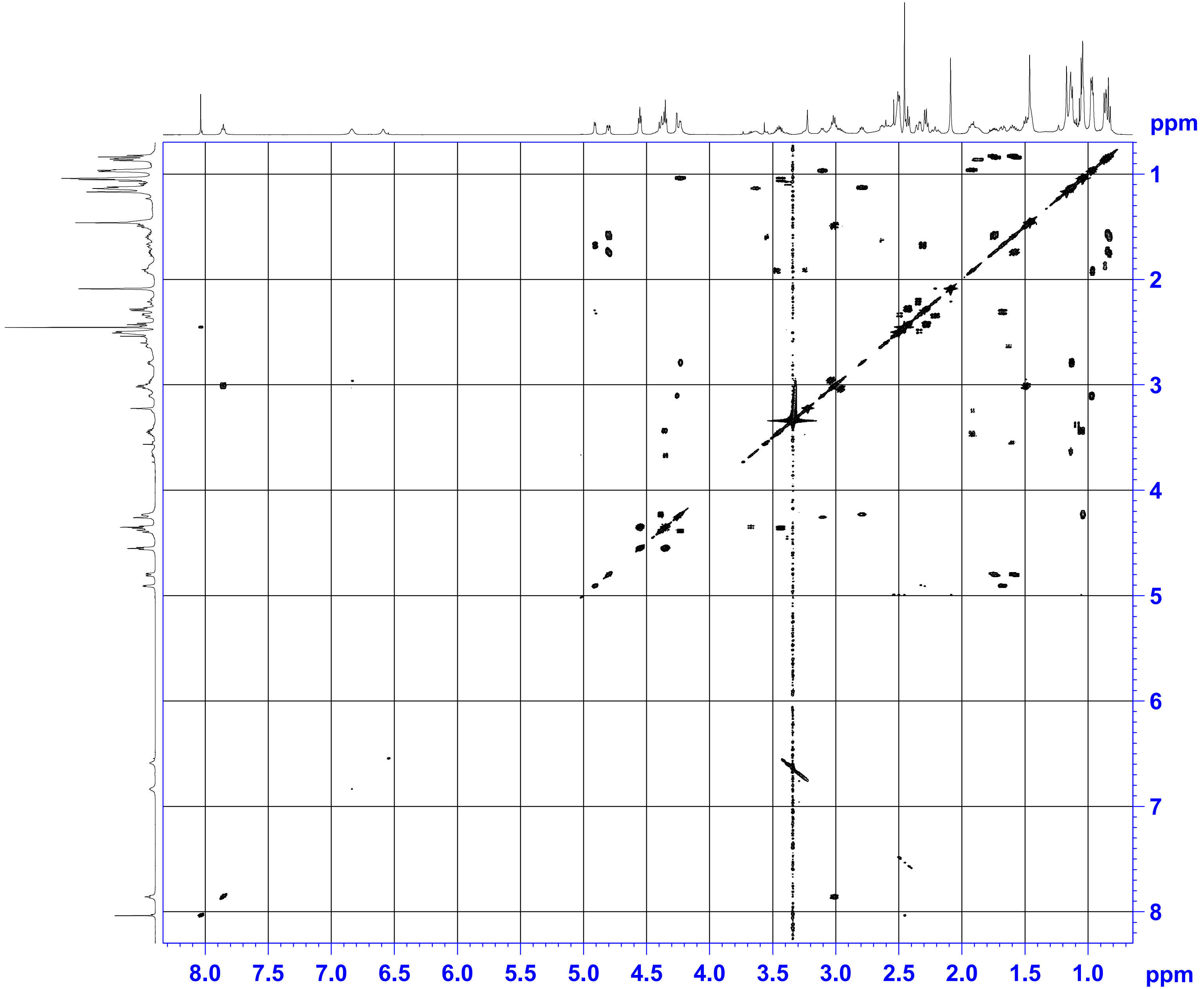


**Figure S45.** ¹H-¹H COSY NMR spectrum of **5b** (DMSO-$d_6$).

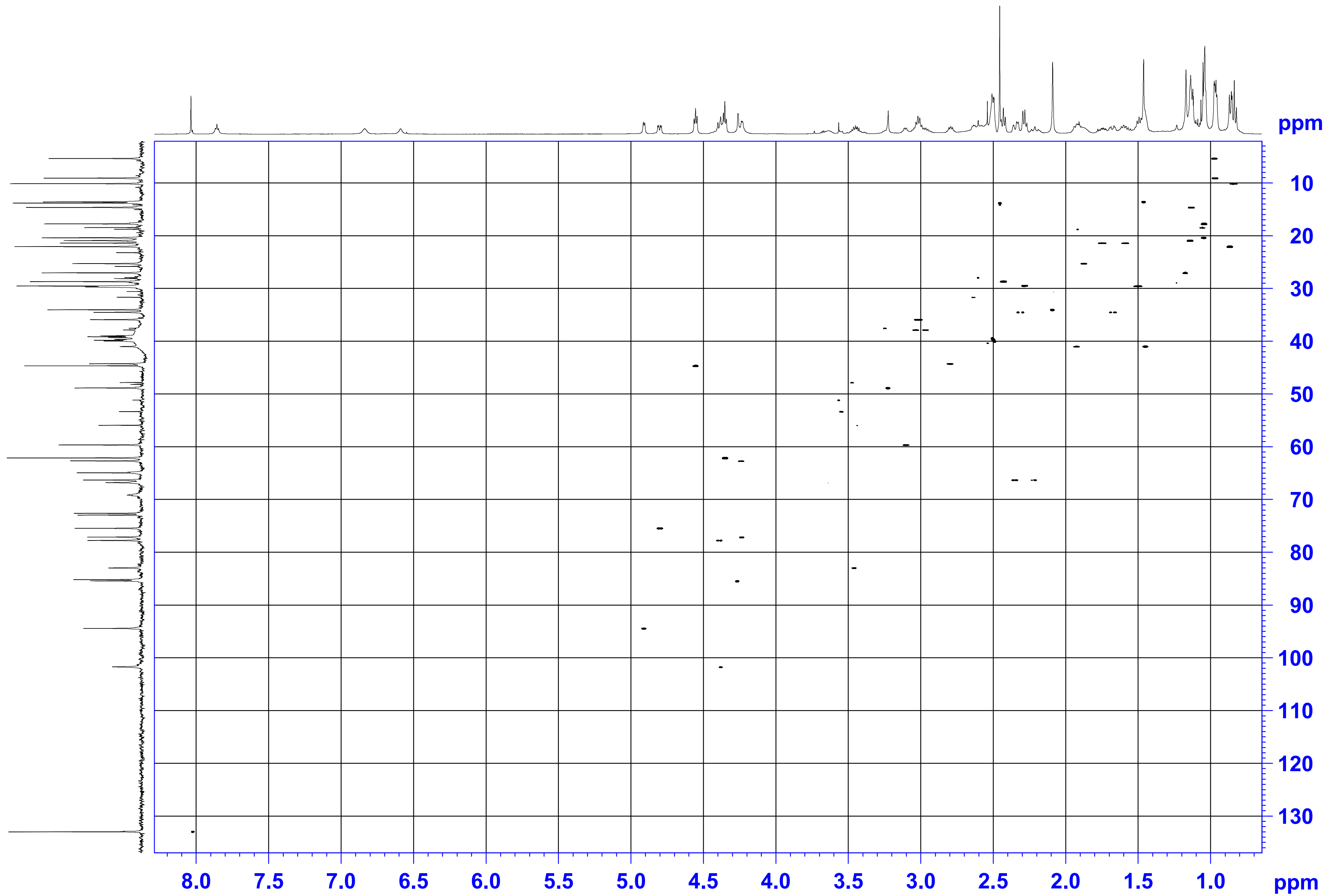


**Figure S46.** $^{1}H$-$^{13}C$ HSQC NMR spectrum of **5b** (DMSO-$d_6$).

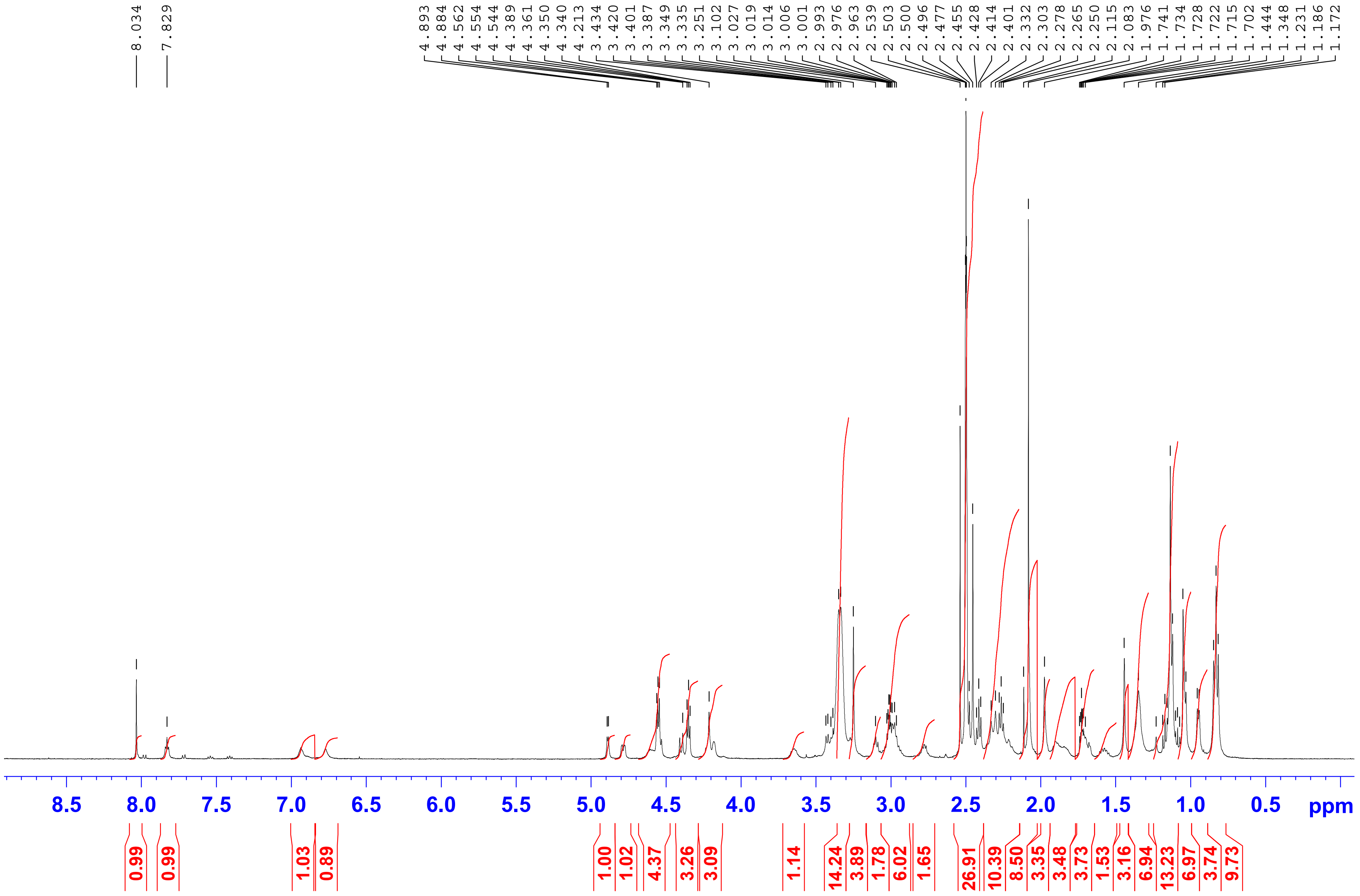

**Figure S47.** $^{1}$H NMR spectra of **5c** (500 MHz, DMSO-$d_6$).

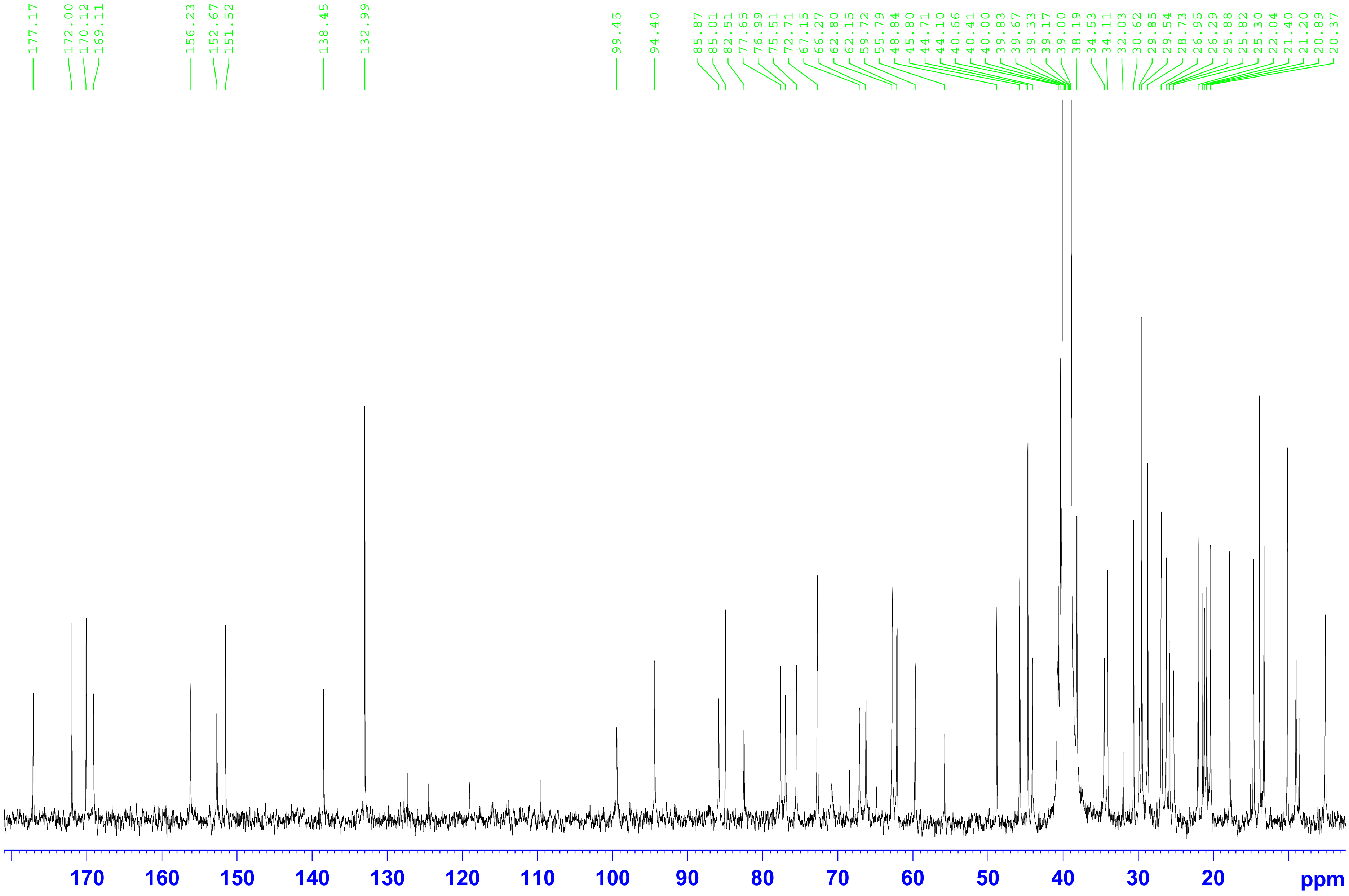


**Figure S48.** $^{13}C$ NMR spectra of **5c** (125 MHz, DMSO-$d_6$).

LCTA-3065 COSY.

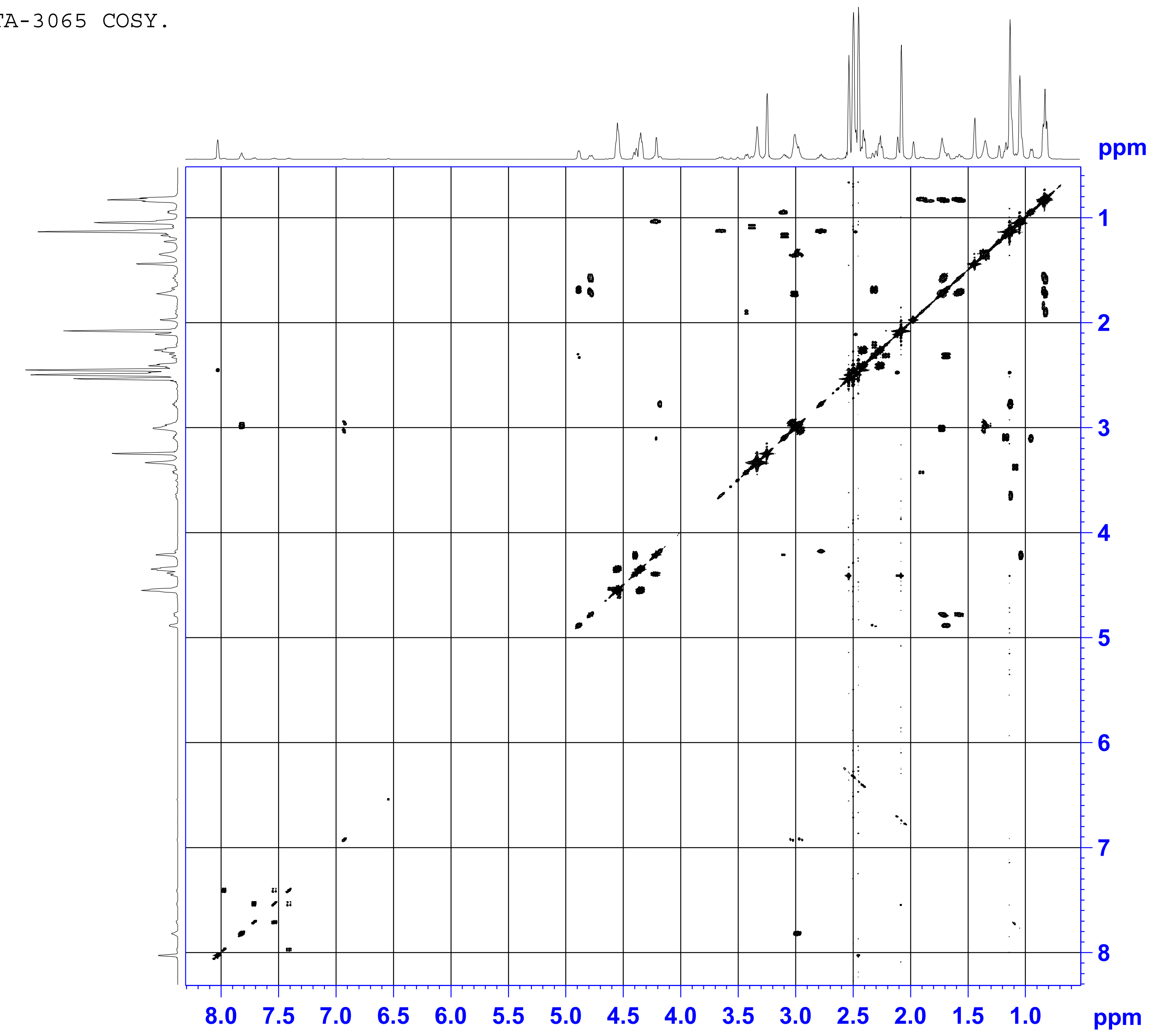


**Figure S49.** $^1H$-$^1H$ COSY NMR spectrum of **5c** (DMSO-$d_6$).

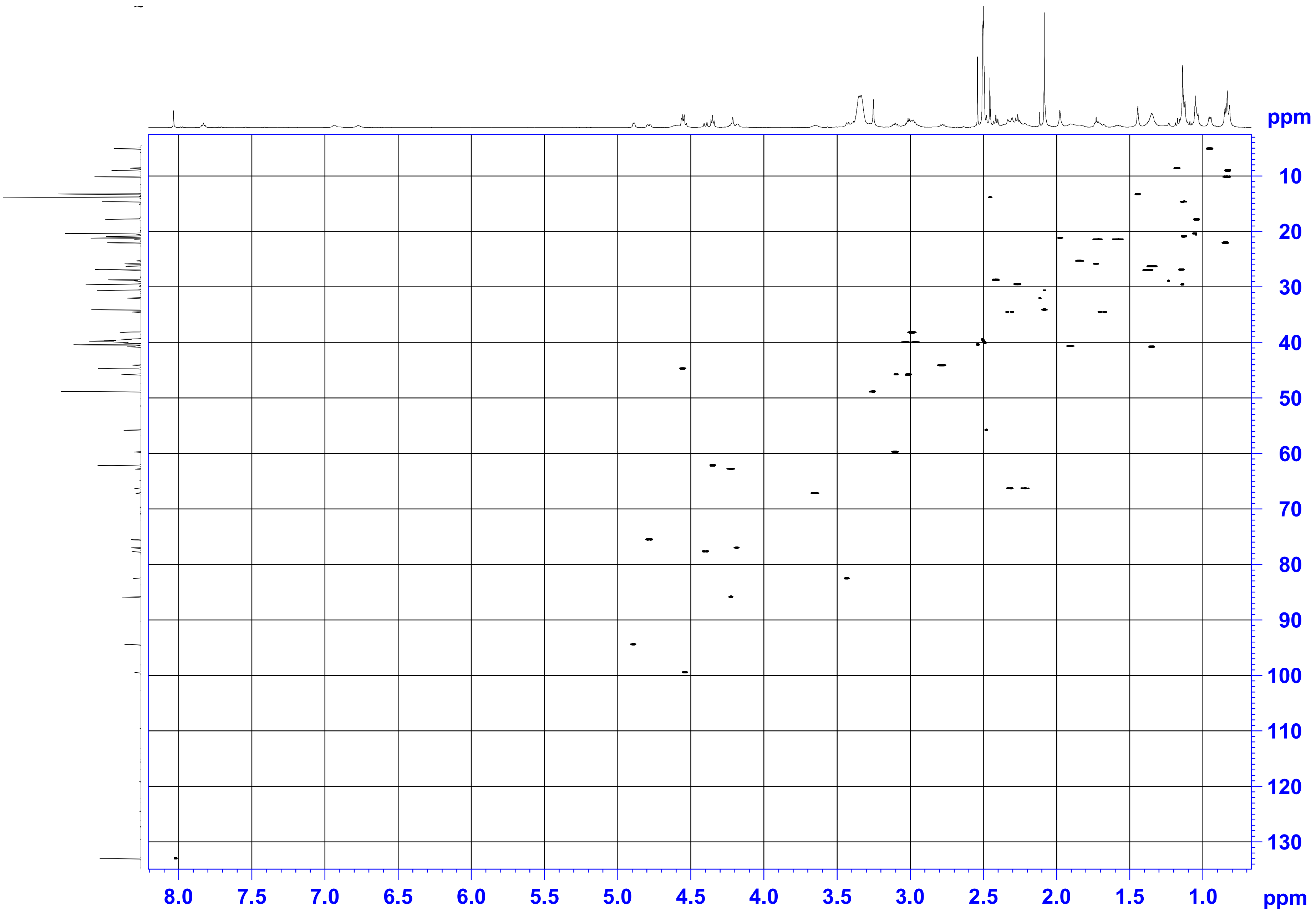


**Figure S50.** $^{1}H$-$^{13}C$ HSQC NMR spectrum of **5c** (DMSO-$d_6$).

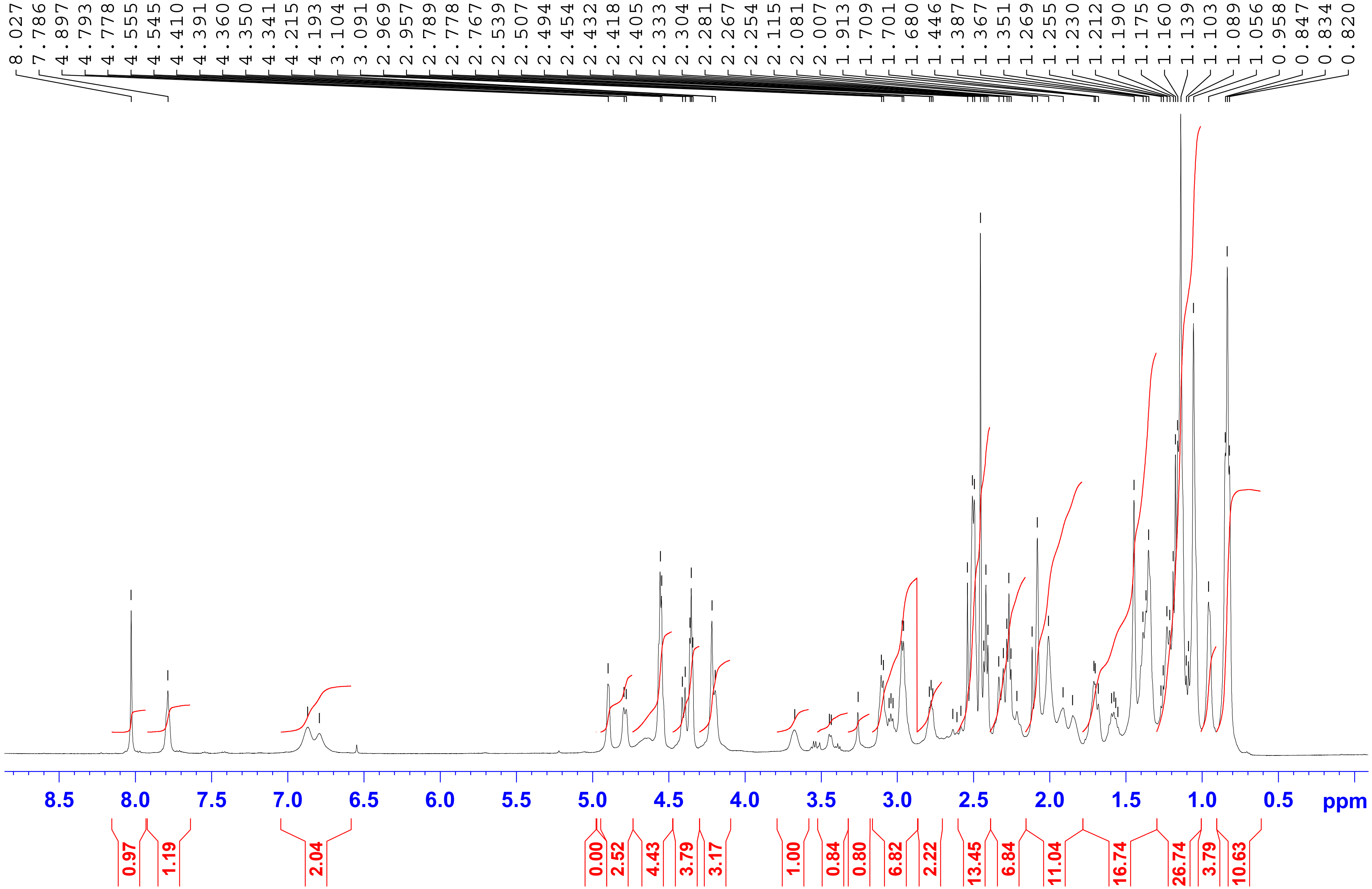


**Figure S51.** $^{1}$H NMR spectra of **5d** (500 MHz, DMSO-*d*6).

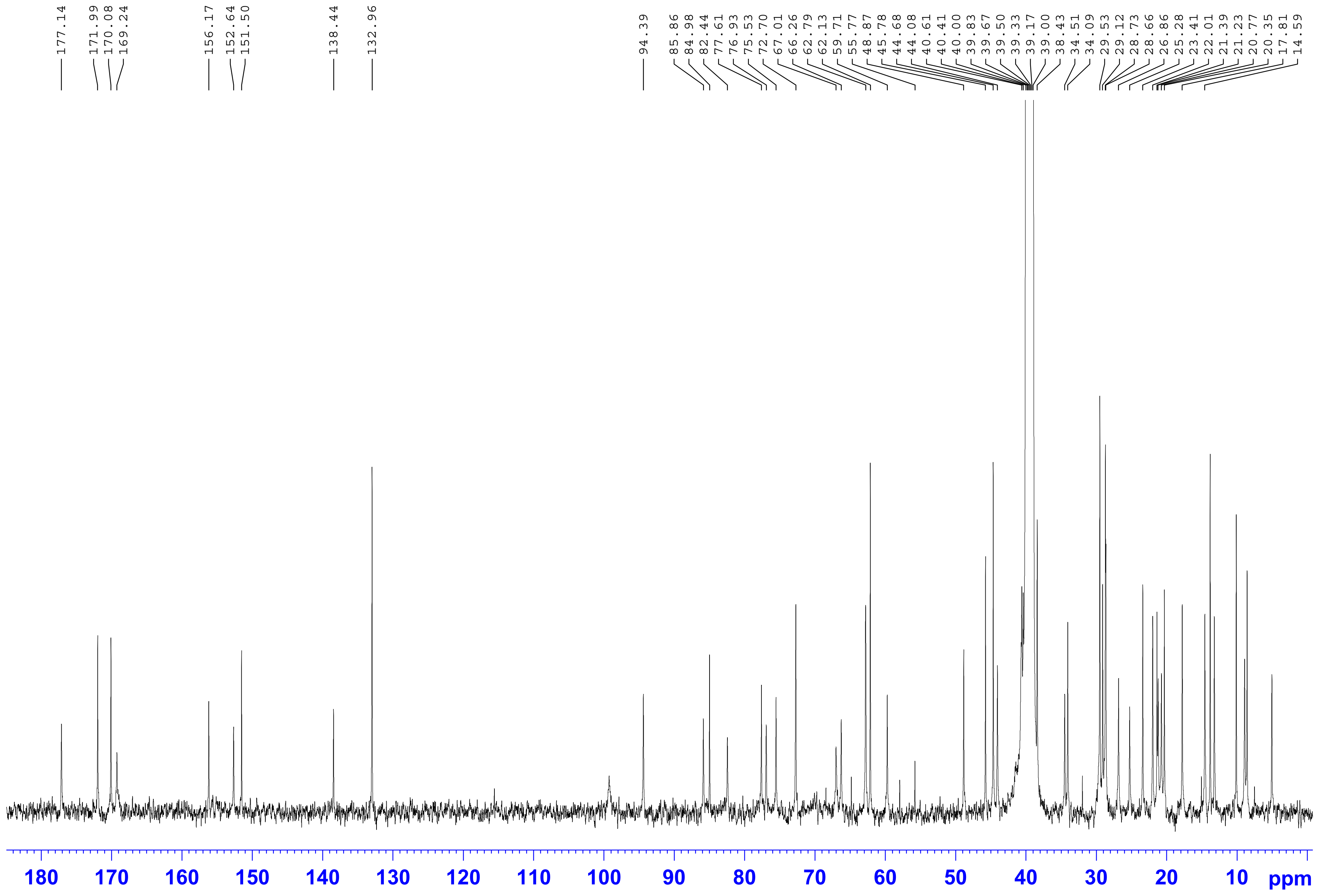


**Figure S52.** $^{13}C$ NMR spectra of **5d** (125 MHz, DMSO-$d_6$).

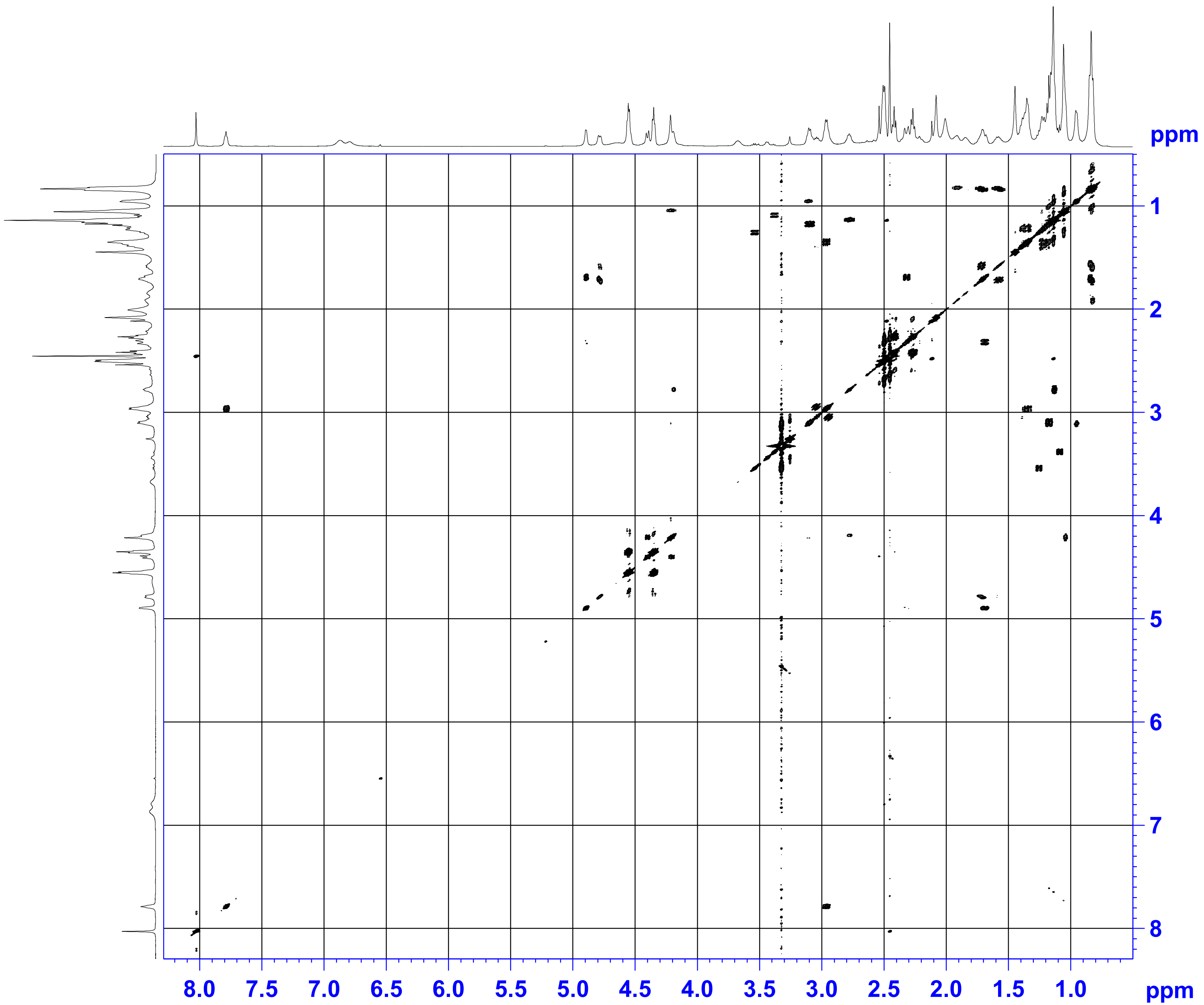


**Figure S53.** $^{1}$H-$^{1}$H COSY NMR spectrum of **5d** (DMSO-$d_6$).

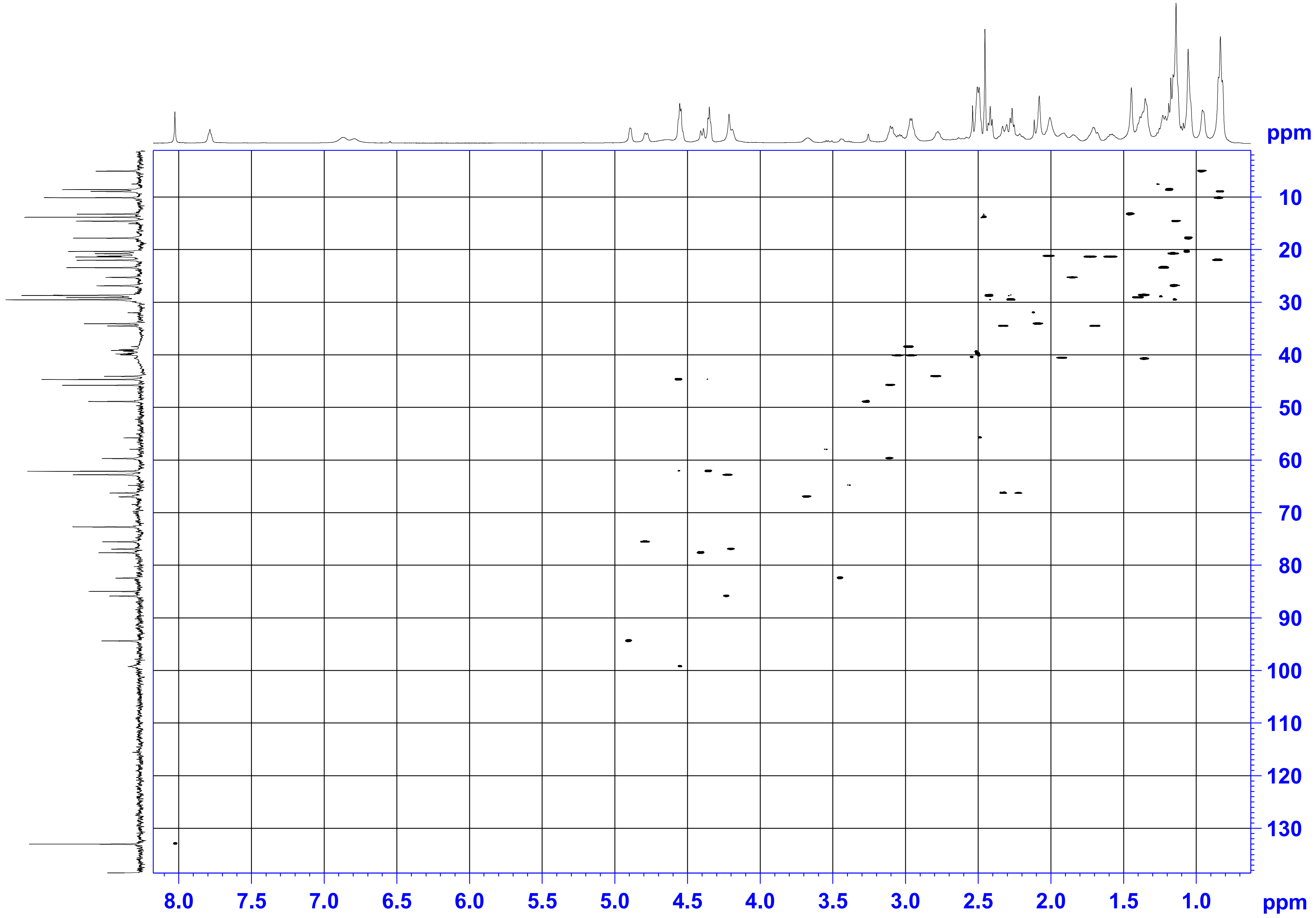


**Figure S54.** $^{1}H$-$^{13}C$ HSQC NMR spectrum of **5d** (DMSO-$d_6$).

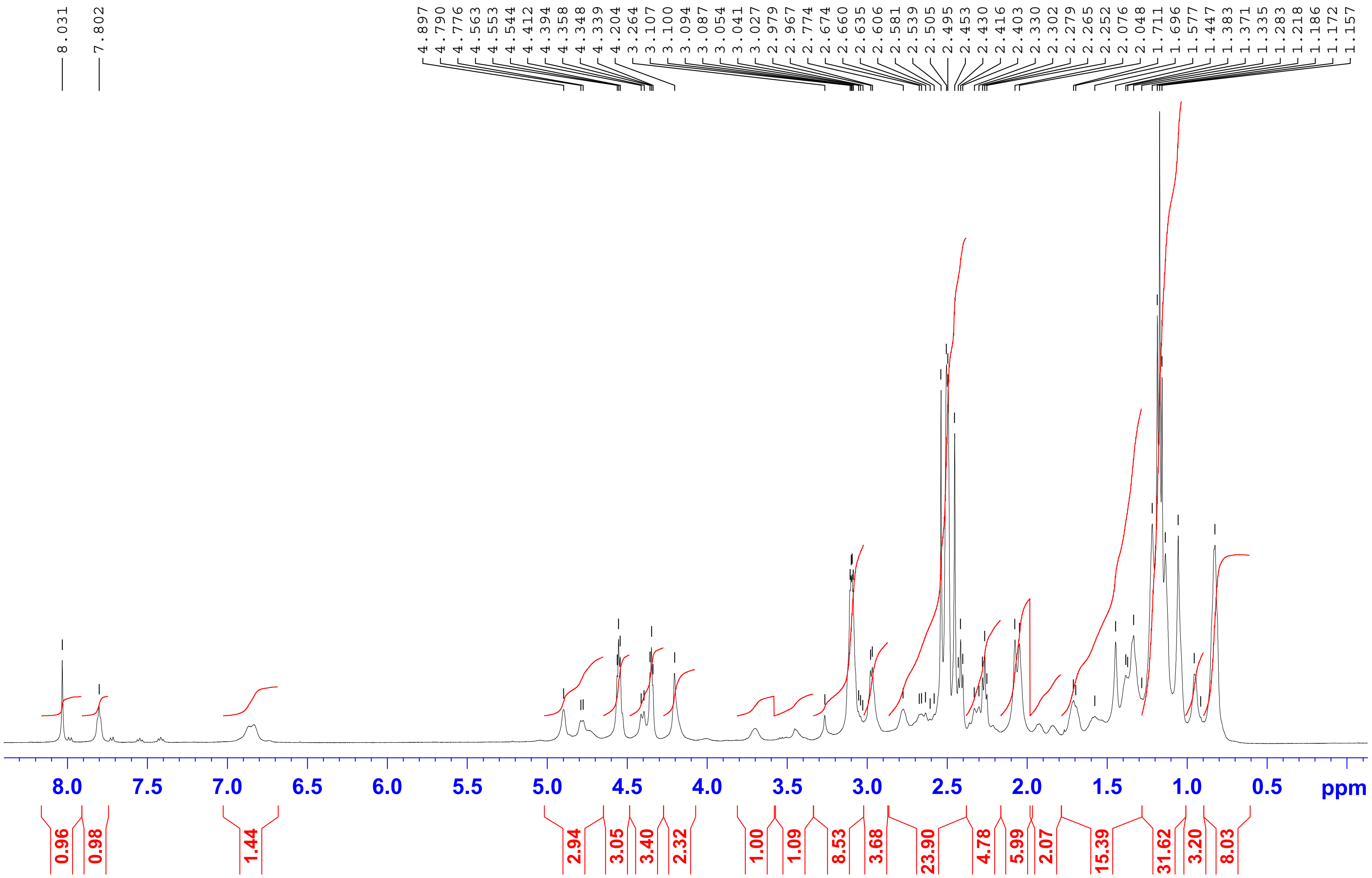

**Figure S55.** $^{1}$H NMR spectra of **5e** (500 MHz, DMSO-*d*6).

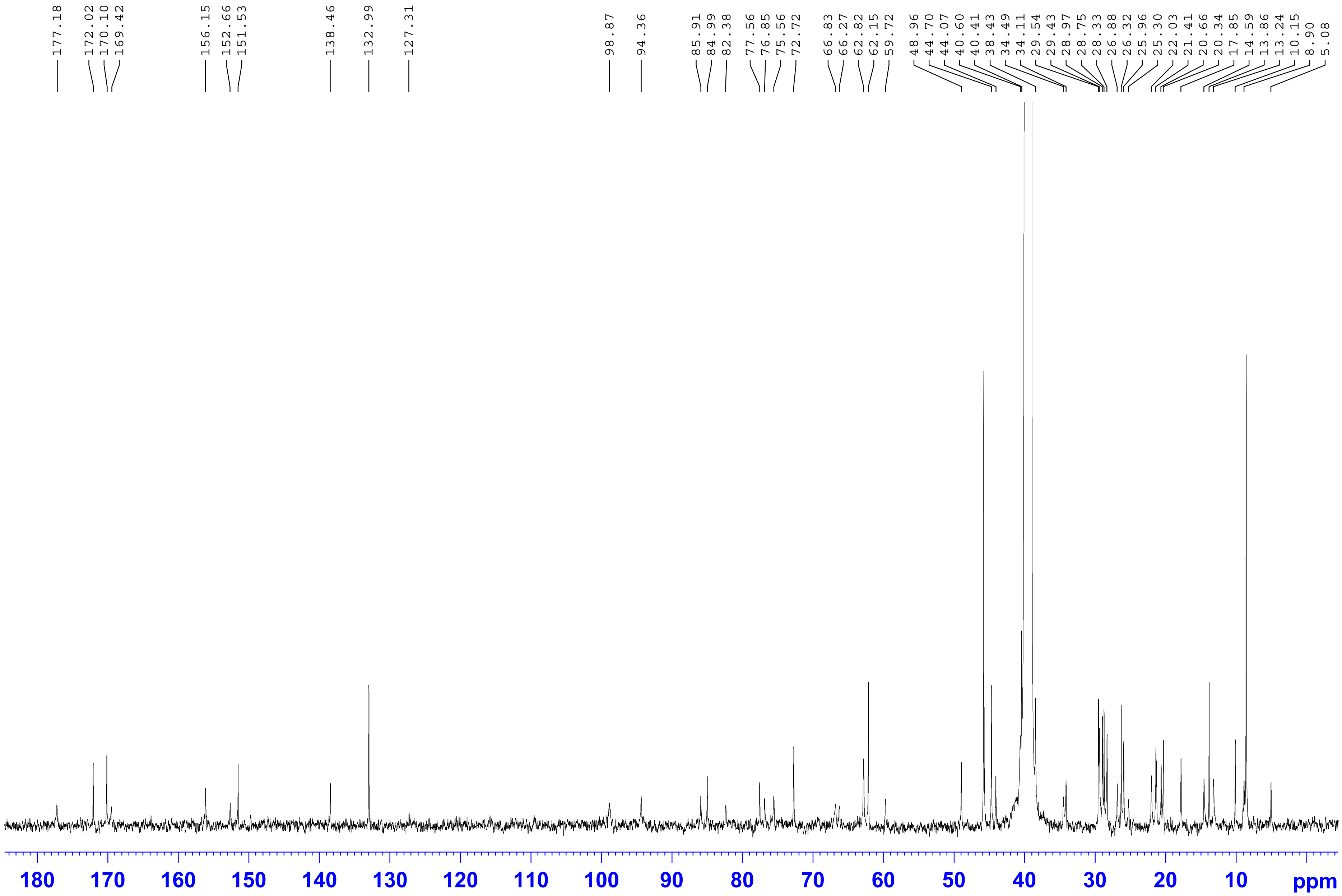


**Figure S56.** $^{13}C$ NMR spectra of **5e** (125 MHz, DMSO-*d*$_6$).

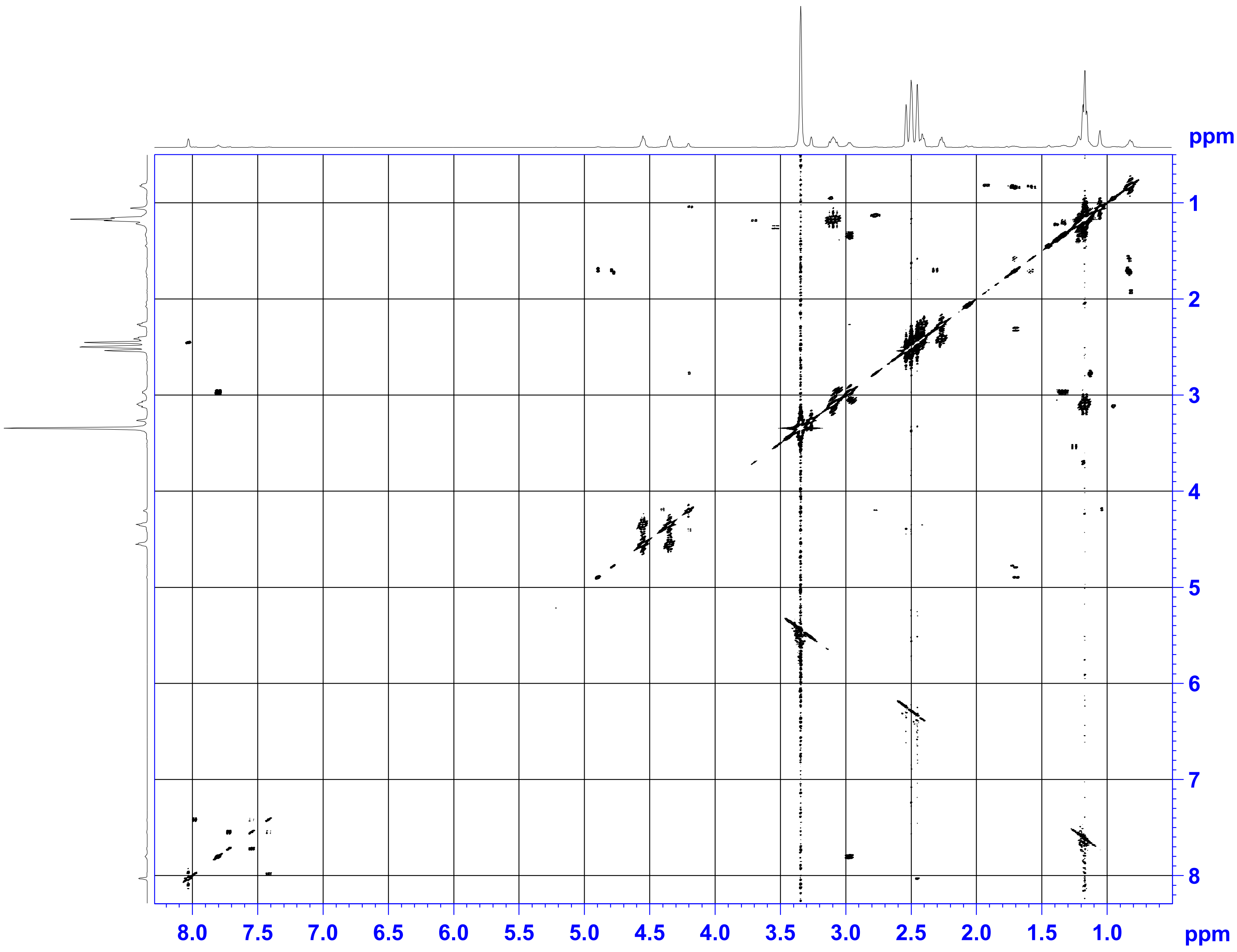


**Figure S57.** $^{1}H$-$^{1}H$ COSY NMR spectrum of **5e** (DMSO-$d_6$).

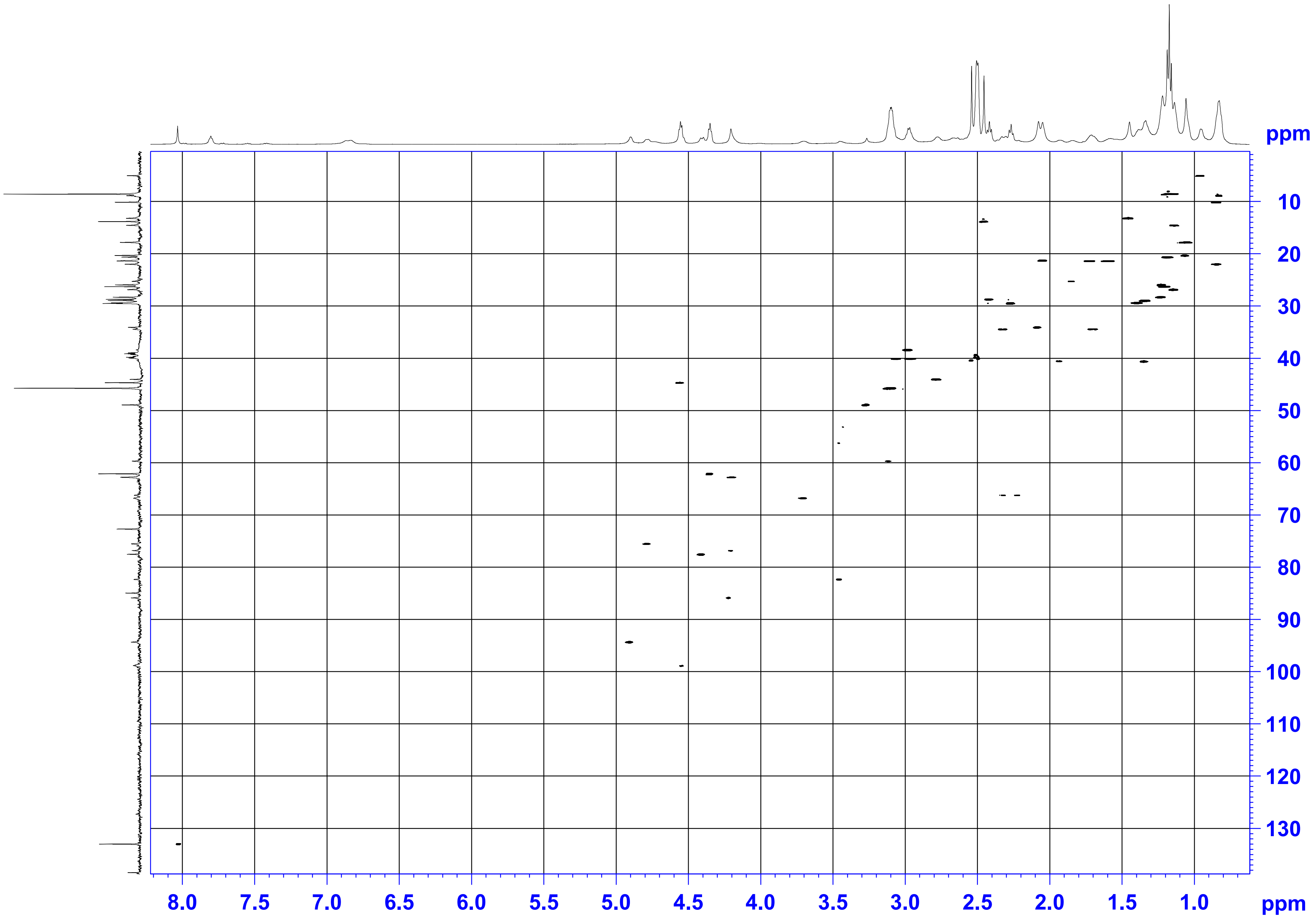


**Figure S58.** $^{1}H$-$^{13}C$ HSQC NMR spectrum of **5e** (DMSO-$d_6$).

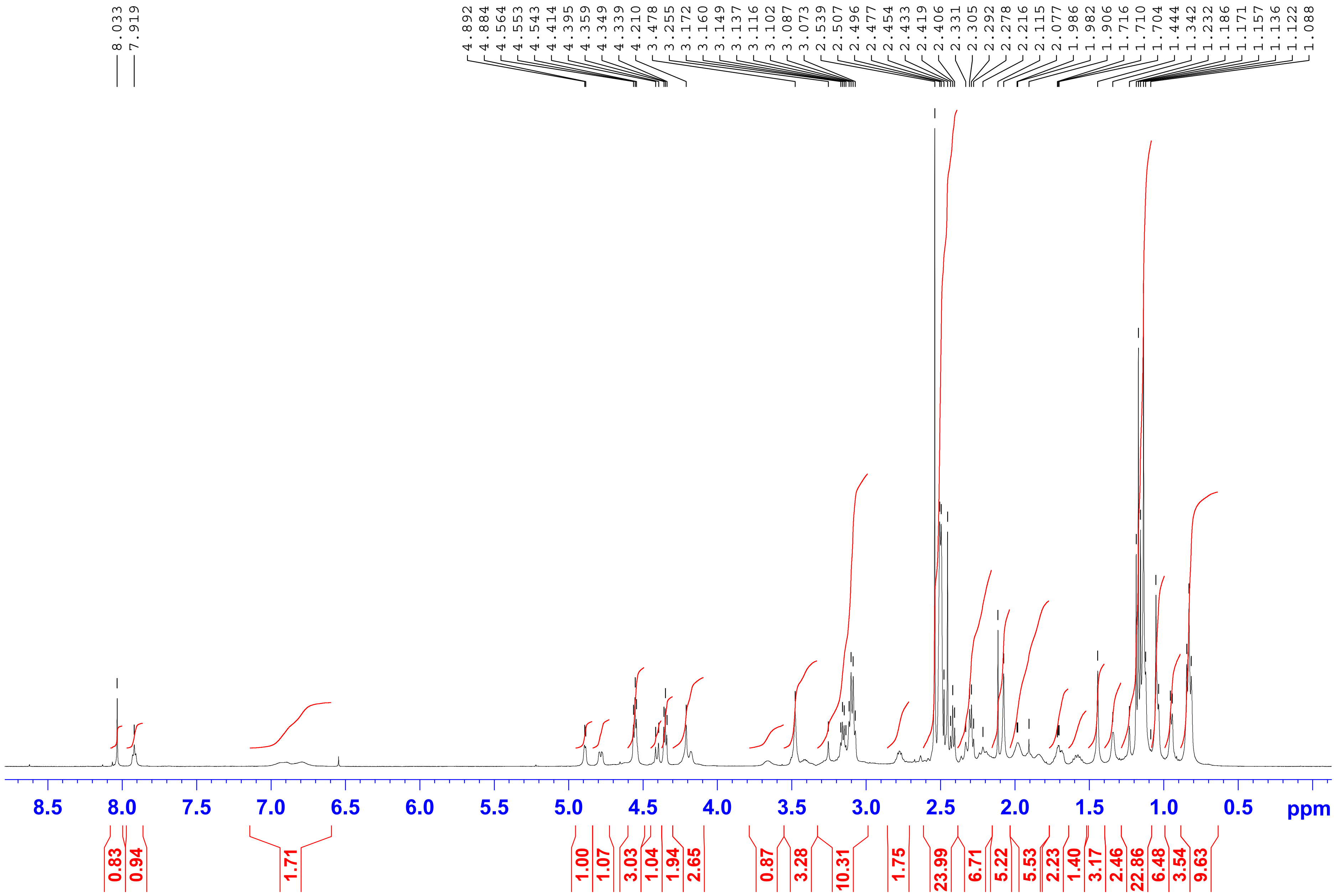


**Figure S59.** ¹H NMR spectra of **5f** (500 MHz, DMSO-*d*₆).

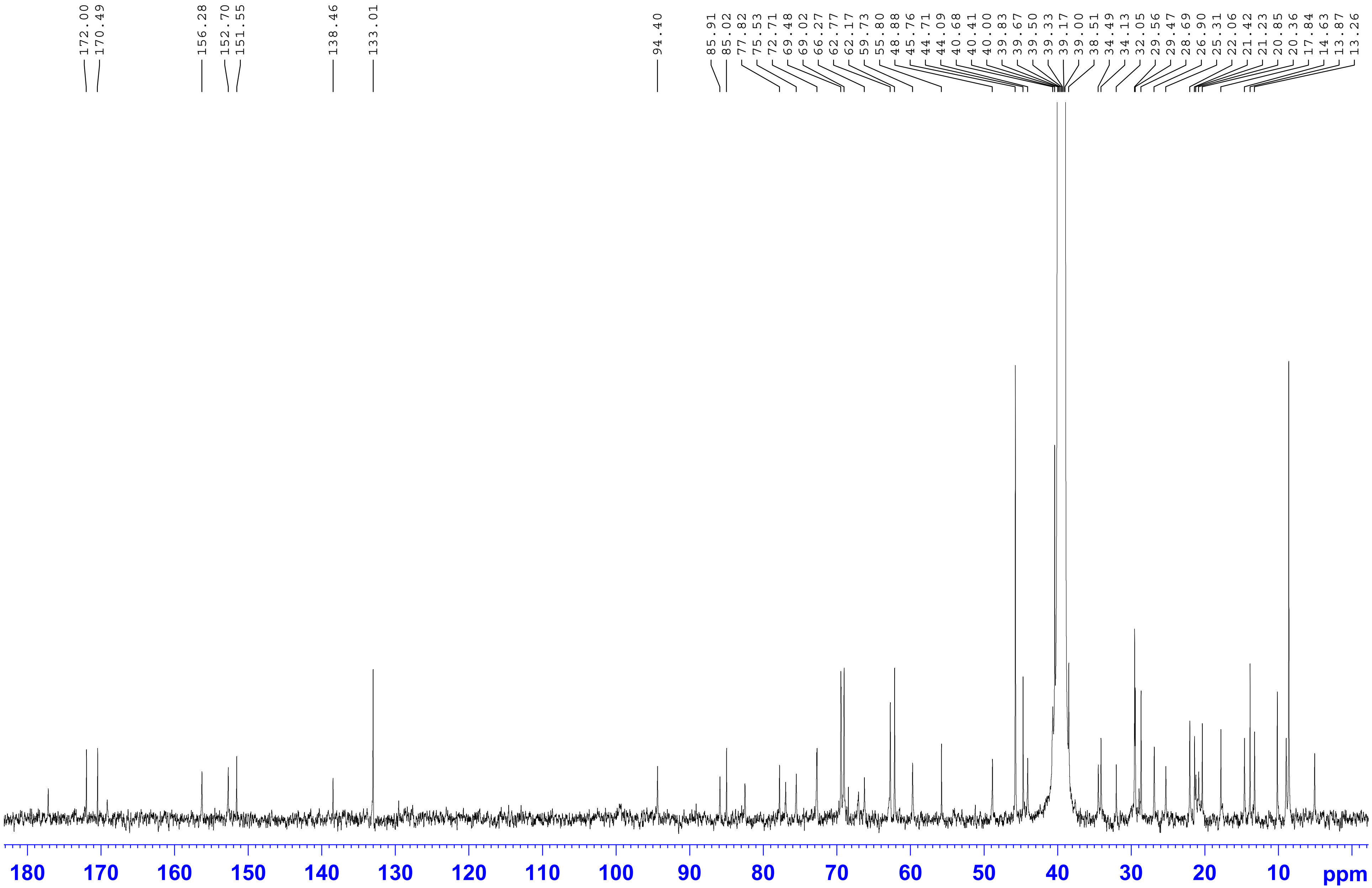


**Figure S60.** $^{13}C$ NMR spectra of **5f** (125 MHz, DMSO-$d_6$).

LCTA-3062 COSY

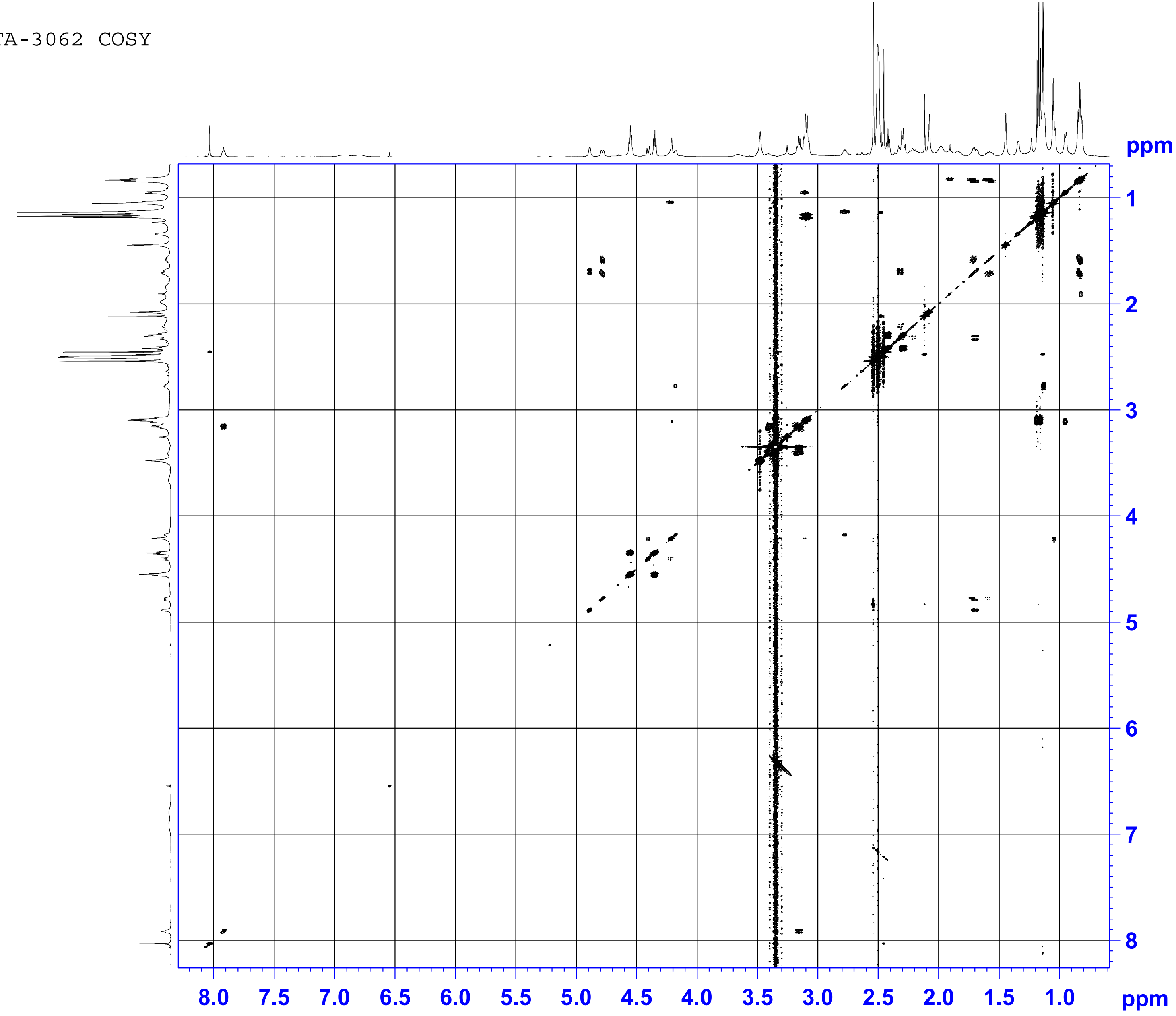


**Figure S61.** $^1H$-$^1H$ COSY NMR spectrum of **5f** (DMSO-*d*$_6$).

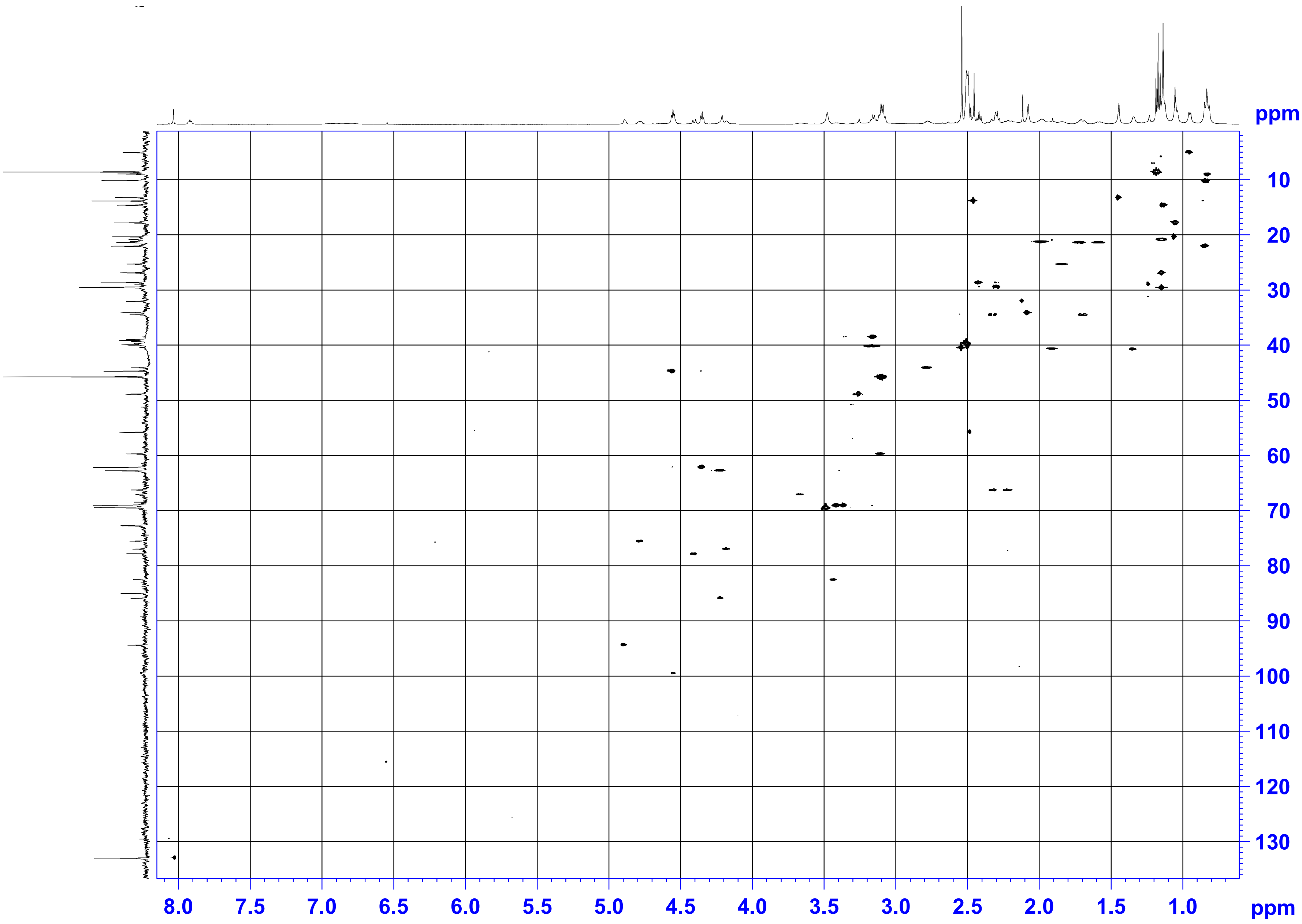


**Figure S62.** $^{1}H$-$^{13}C$ HSQC NMR spectrum of **5f** (DMSO-$d_6$).

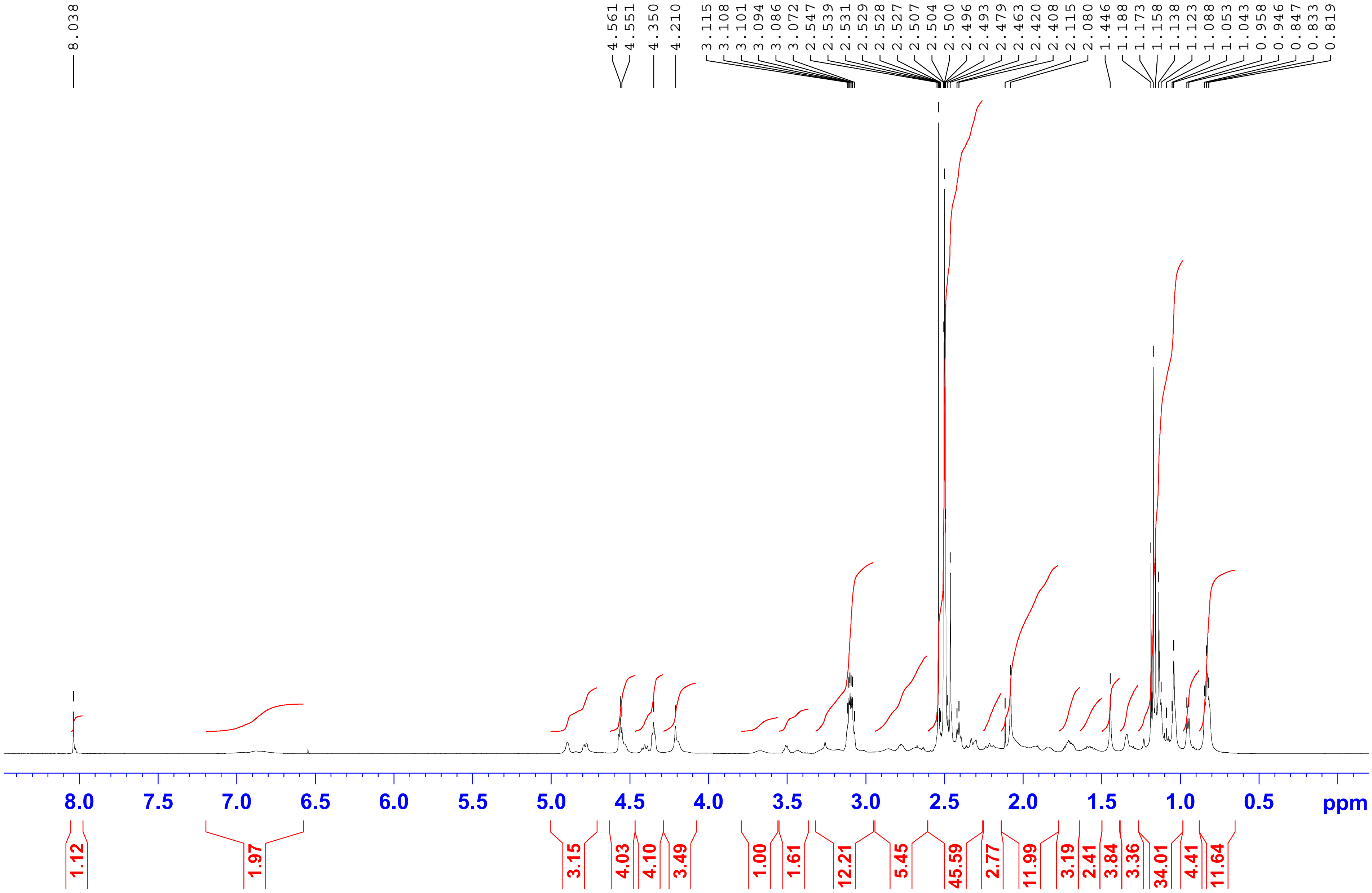

**Figure S63.** $^{1}$H NMR spectra of **5g** (500 MHz, DMSO-$d_6$).

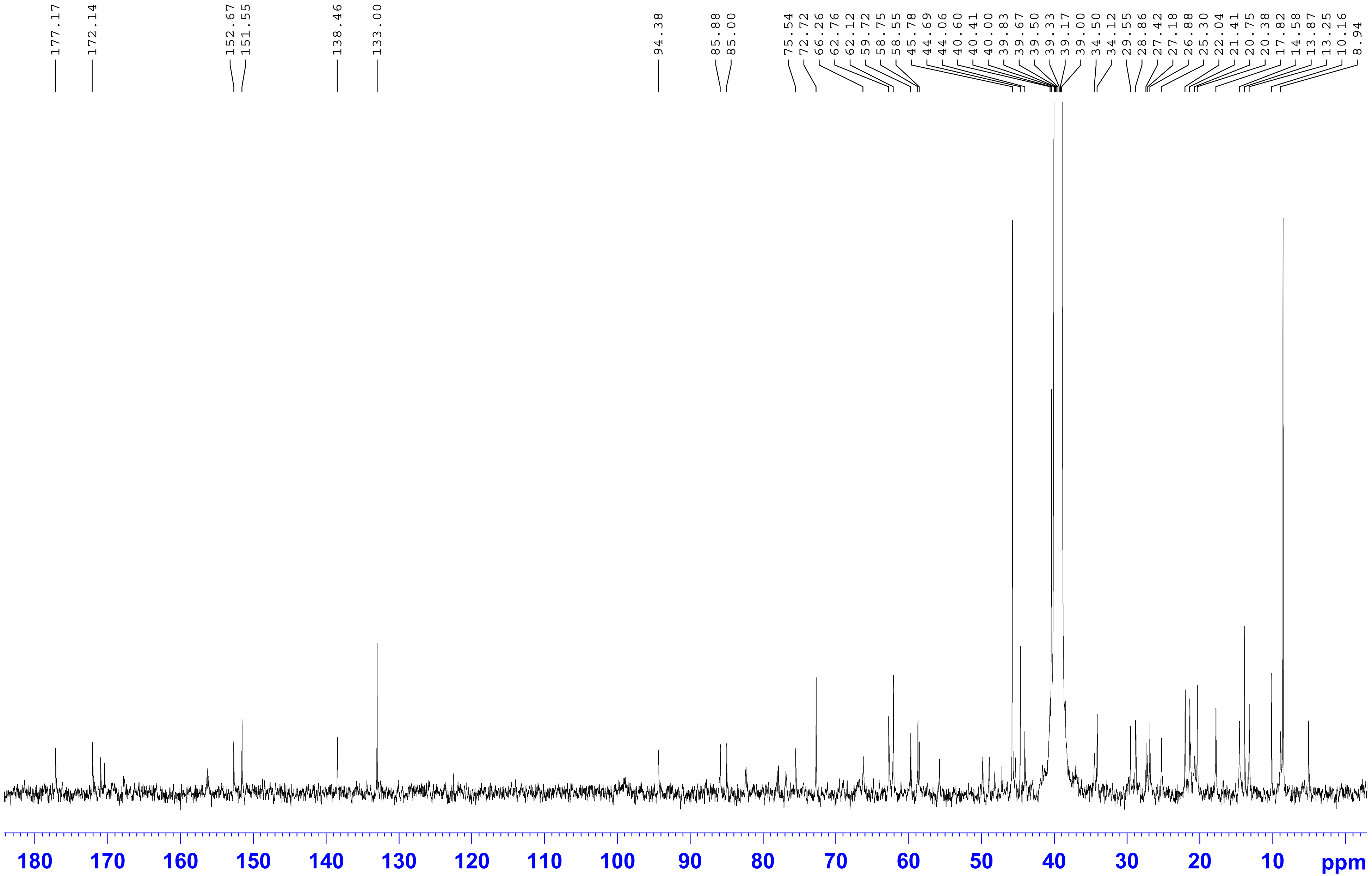


**Figure S64.** $^{13}$C NMR spectra of **5g** (125 MHz, DMSO-*d*$_6$).

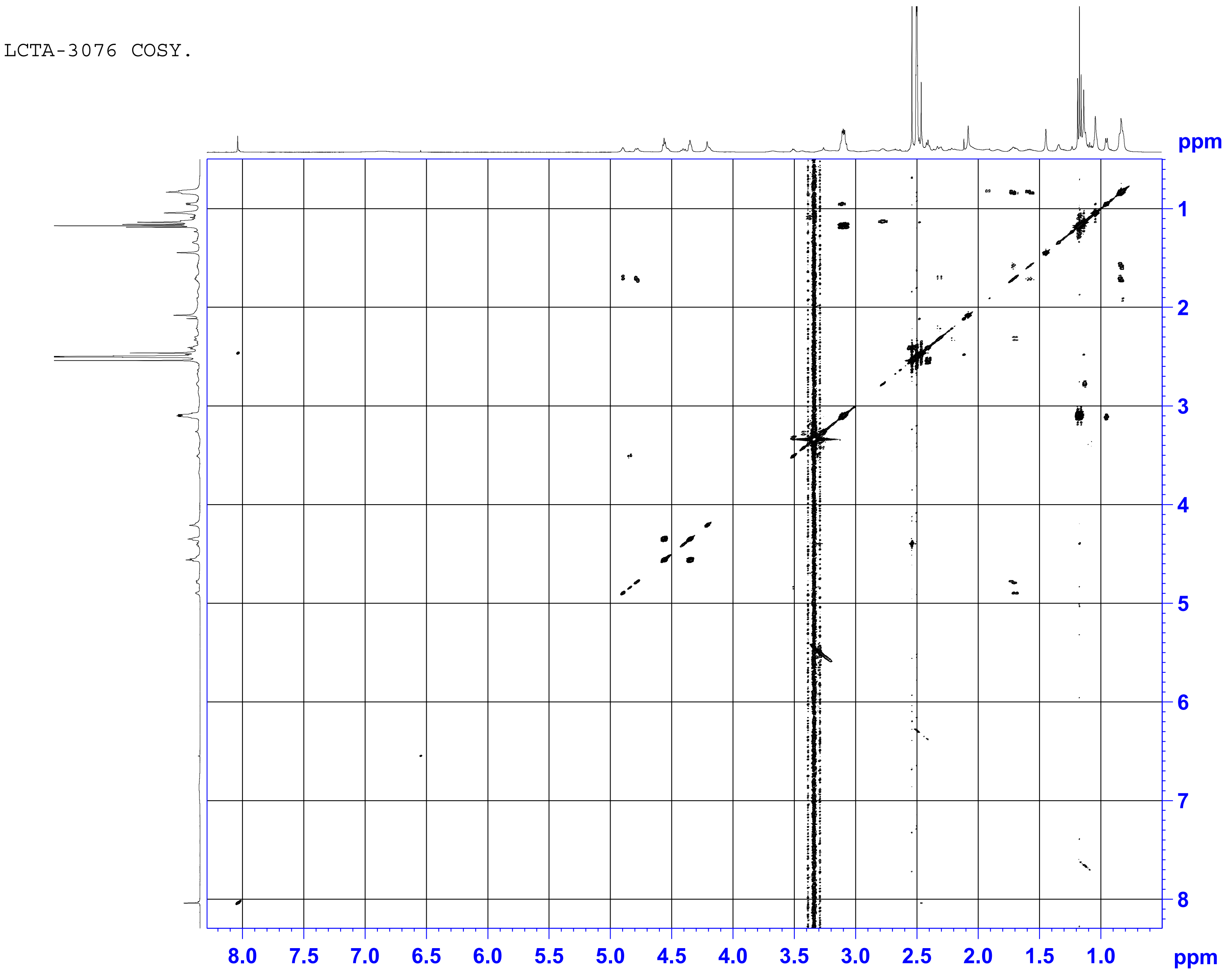


**Figure S65.** $^{1}H$-$^{1}H$ COSY NMR spectrum of **5g** (DMSO-$d_6$).

LCTA-3076 HSQC.

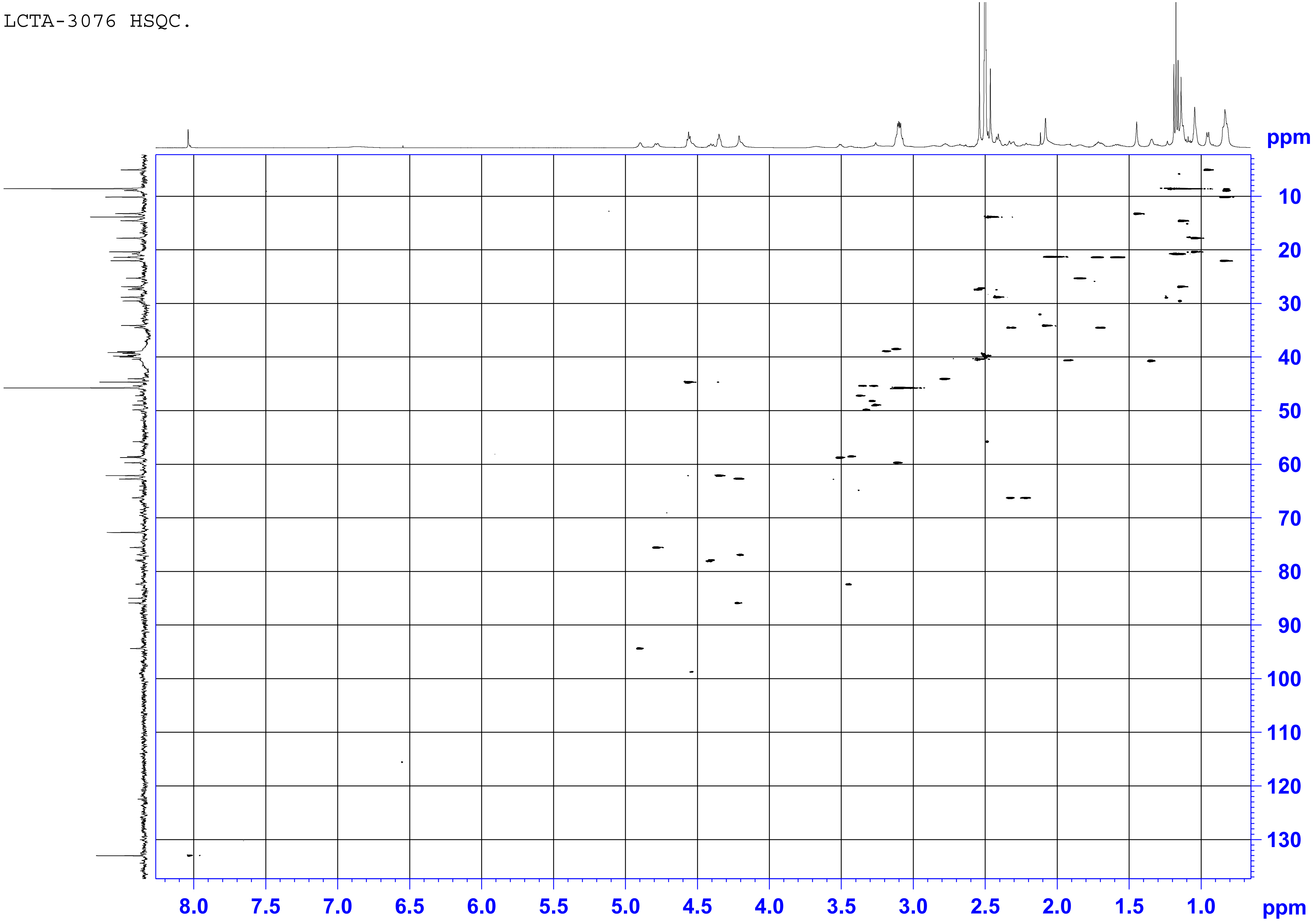


**Figure S66.** $^{1}$H-$^{13}$C HSQC NMR spectrum of **5g** (DMSO-$d_6$).

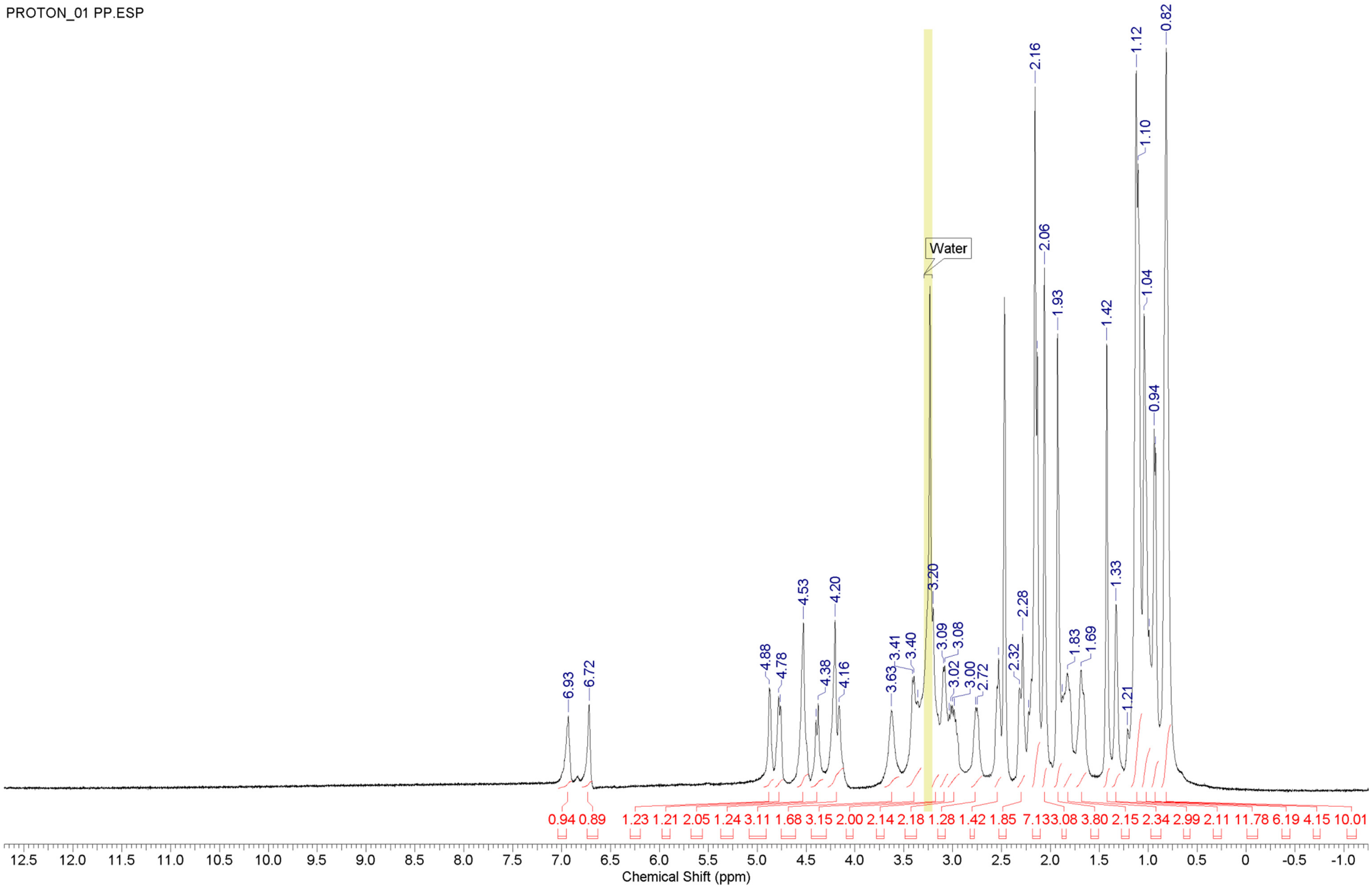


**Figure S67.** $^{1}H$ NMR spectra of **6** (400 MHz, DMSO-$d_6$).

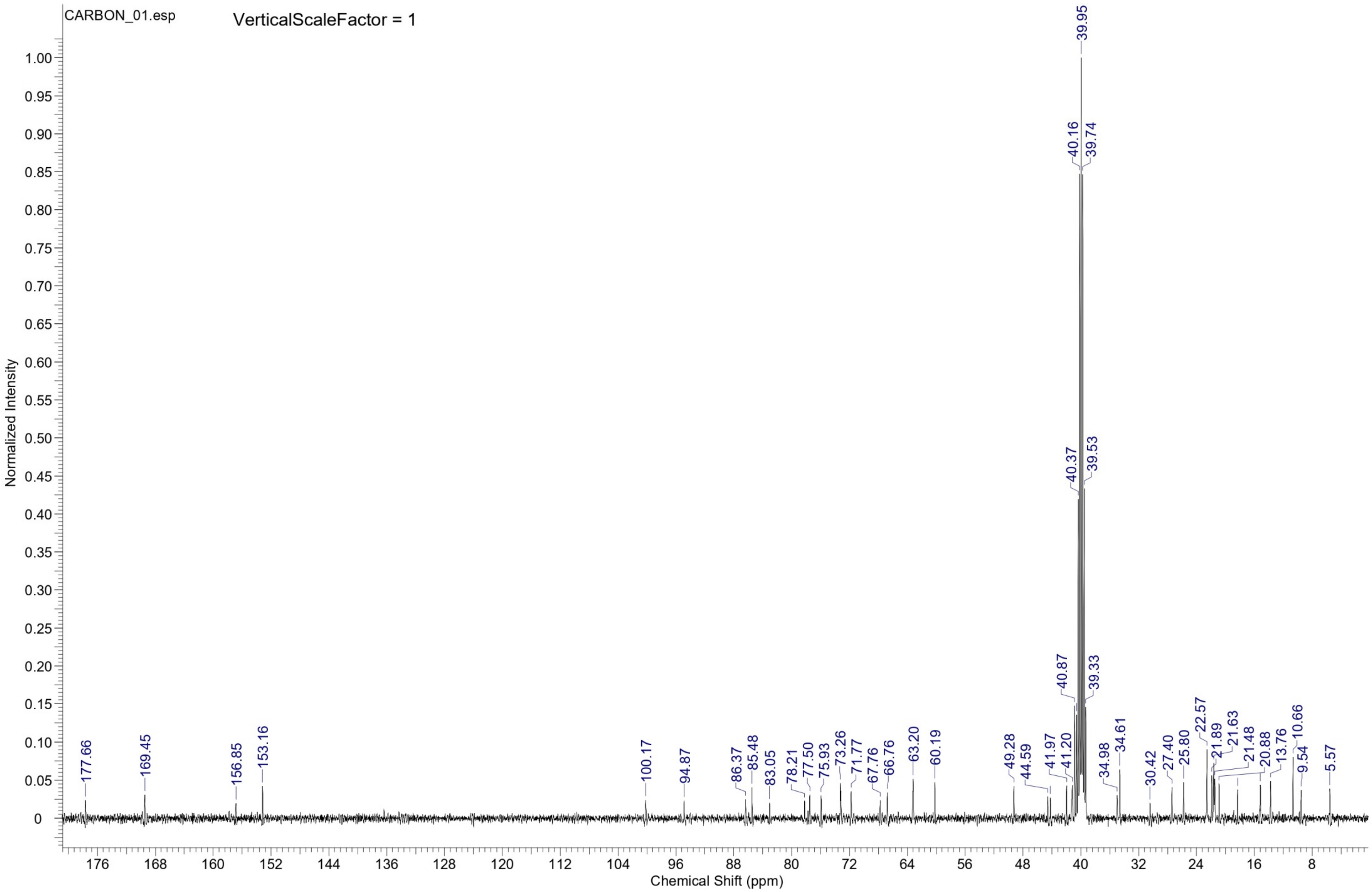


**Figure S68.** $^{13}C$ NMR spectra of **6** (100 MHz, DMSO-$d_6$).

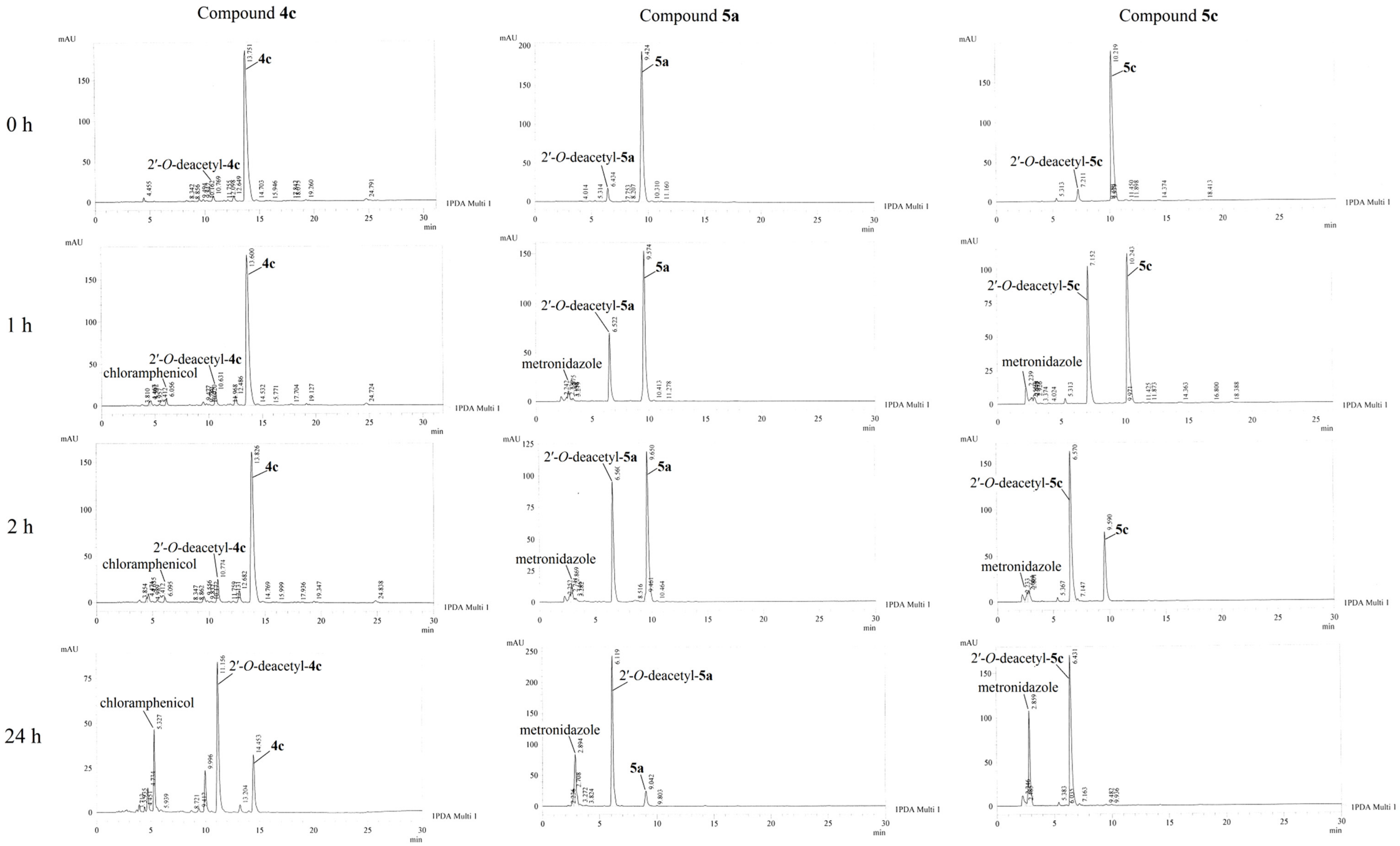


**Figure S69.** HPLC analysis of compounds **4c**, **5a**, **5c** stability in PBS buffer (pH 7.4) during incubation at 37 °C.

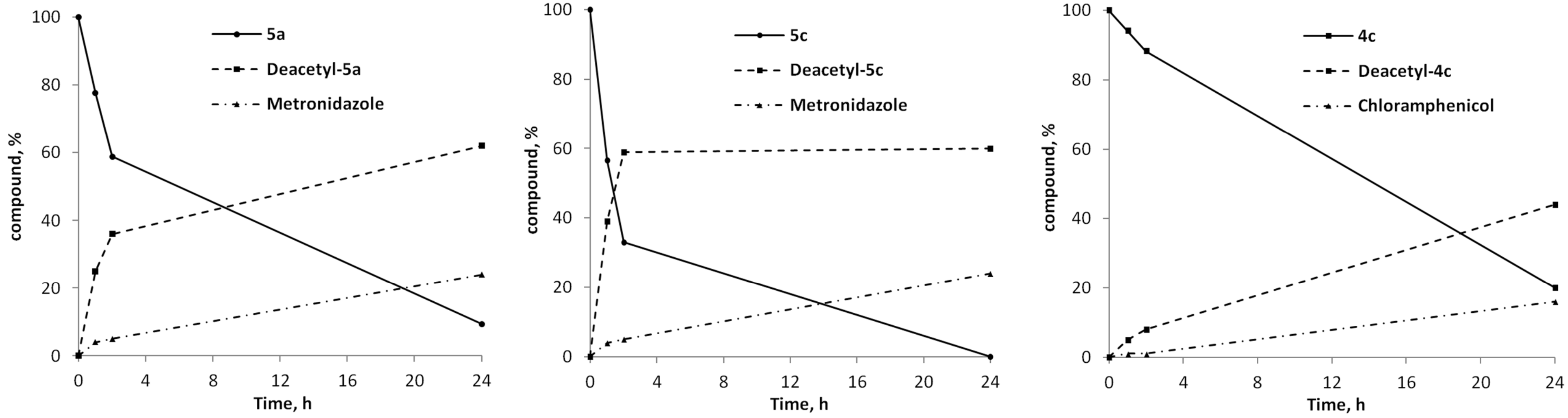


**Figure S70.** Stability of compounds **4c**, **5a** and **5c** in PBS buffer (pH 7.4) upon incubation at 37 °C represented as kinetic curves.

# Compound Spectrum List Report

**Analysis Info**

| | | | |
|---|---|---|---|
| Analysis Name | D:\Data\AST\3146-2.d | Acquisition Date | 1/16/2024 9:08:14 PM |
| Method | tune_norm.m | Operator | Mitrokhov |
| Sample Name | Tune wide | Instrument / Ser# | micrOTOF-Q II 10225 |
| Comment | | | |

**Acquisition Parameter**

| | | | | | |
|---|---|---|---|---|---|
| Source Type | ESI | Ion Polarity | Positive | Set Nebulizer | 0.4 Bar |
| Focus | Not active | Set Capillary | 4500 V | Set Dry Heater | 180 °C |
| Scan Begin | 50 m/z | Set End Plate Offset | -500 V | Set Dry Gas | 4.0 l/min |
| Scan End | 3000 m/z | Set Collision Cell RF | 550.0 Vpp | Set Divert Valve | Source |

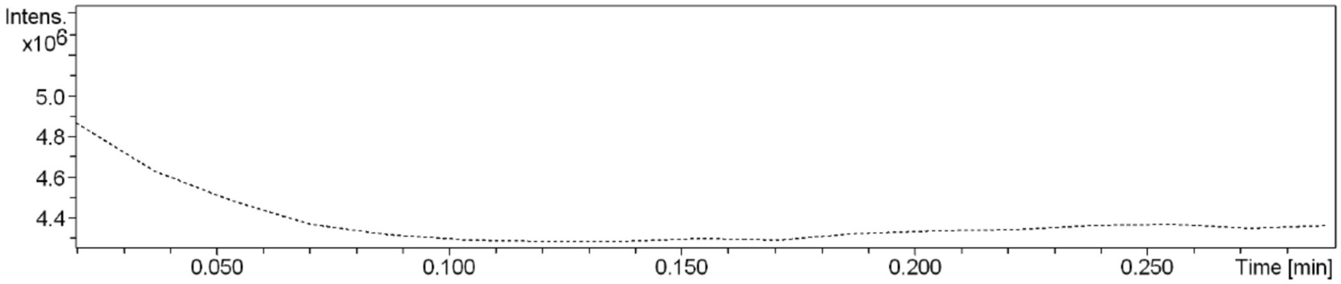


| # | RT [min] | Area | Int. Type | Intens. | S/N | Chromatogram | Max. m/z |
|---|---|---|---|---|---|---|---|
| n.a. | 0.1 | n.a. | Average spectrum | n.a. | n.a. | n.a. | 647.2994 |

**+MS, 0.1-0.1min #(3-7)**

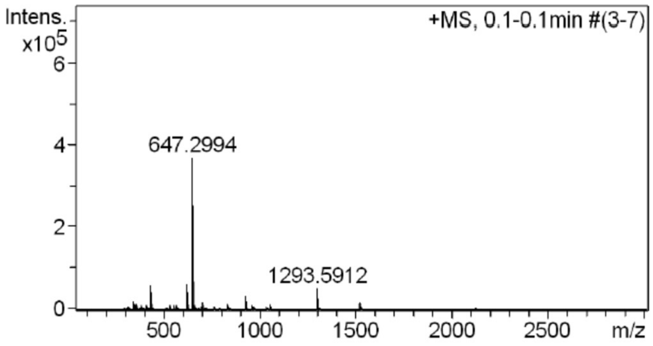


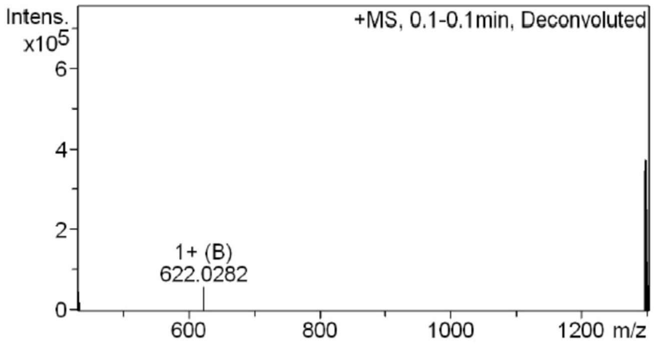


| # | m/z | Res. | S/N | I | I % |
|---|---|---|---|---|---|
| 1 | 431.1610 | 6773 | 494.5 | 56790 | 15.4 |
| 2 | 622.0284 | 7531 | 422.7 | 59946 | 16.3 |
| 3 | 647.2994 | 7116 | 2516.0 | 368502 | 100.0 |
| 4 | 647.8009 | 7160 | 1715.7 | 251531 | 68.3 |
| 5 | 648.2990 | 7140 | 2244.2 | 329160 | 89.3 |
| 6 | 648.7994 | 7295 | 1247.6 | 183219 | 49.7 |
| 7 | 649.2987 | 7400 | 699.7 | 102932 | 27.9 |
| 8 | 649.7990 | 7644 | 298.3 | 44035 | 11.9 |
| 9 | 1293.5912 | 8480 | 517.3 | 52054 | 14.1 |
| 10 | 1295.5901 | 8993 | 484.4 | 48726 | 13.2 |

| # | m/z | Res. | S/N | I | I % |
|---|---|---|---|---|---|
| 1 | 431.1589 | | | 56790 | 19.8 |
| 2 | 622.0282 | | | 59946 | 20.9 |
| 3 | 1294.5874 | | | 286682 | 100.0 |

**Figure S71.** HRMS-ESI of the **4c** metabolite corresponding to 2′-*O*-deacetyl-**4c**.

# Compound Spectrum List Report

**Analysis Info**

| | | | |
|---|---|---|---|
| Analysis Name | D:\Data\AST\3004(1).d | Acquisition Date | 1/16/2024 9:53:18 PM |
| Method | tune_norm.m | Operator | Mitrokhov |
| Sample Name | Tune wide | Instrument / Ser# | micrOTOF-Q II 10225 |
| Comment | | | |

**Acquisition Parameter**

| | | | | | |
|---|---|---|---|---|---|
| Source Type | ESI | Ion Polarity | Positive | Set Nebulizer | 0.4 Bar |
| Focus | Not active | Set Capillary | 4500 V | Set Dry Heater | 180 °C |
| Scan Begin | 50 m/z | Set End Plate Offset | -500 V | Set Dry Gas | 4.0 l/min |
| Scan End | 3000 m/z | Set Collision Cell RF | 550.0 Vpp | Set Divert Valve | Source |

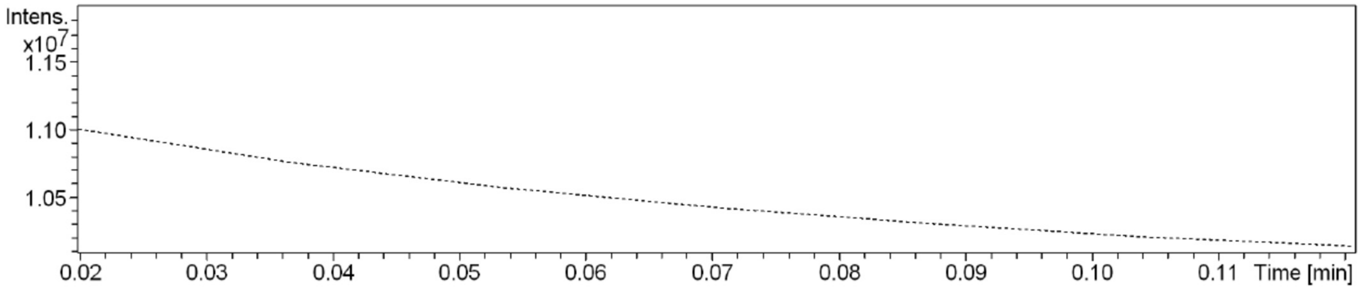


| # | RT [min] | Area | Int. Type | Intens. | S/N | Chromatogram | Max. m/z |
|---|---|---|---|---|---|---|---|
| n.a. | 0.1 | n.a. | Average spectrum | n.a. | n.a. | n.a. | 557.8103 |

**+MS, 0.0-0.1min #(2-7)**

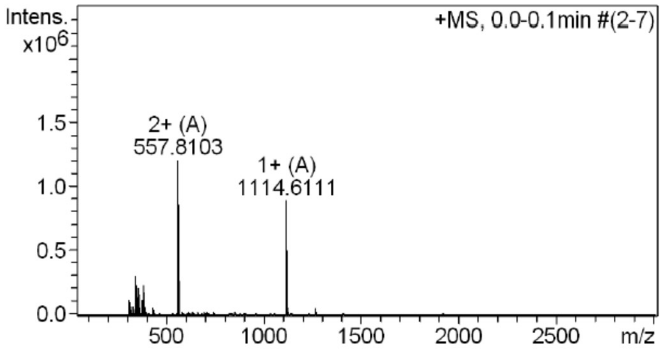


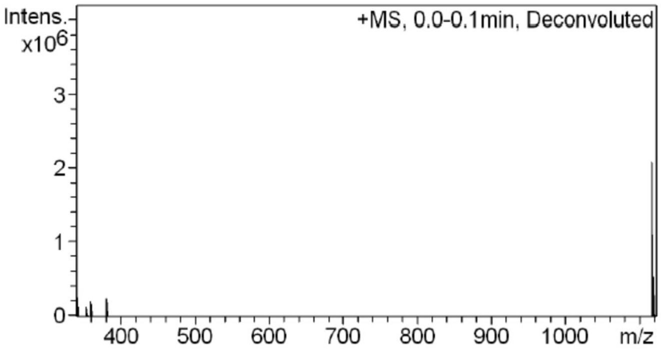


| # | m/z | Res. | S/N | I | I % |
|---|---|---|---|---|---|
| 1 | 341.3048 | 6093 | 1752.1 | 302844 | 25.1 |
| 2 | 353.2653 | 6254 | 772.1 | 137317 | 11.4 |
| 3 | 359.3149 | 6149 | 1127.1 | 203237 | 16.9 |
| 4 | 381.2965 | 6238 | 1225.2 | 232046 | 19.3 |
| 5 | 557.8103 | 5633 | 4834.0 | 1205134 | 100.0 |
| 6 | 558.3107 | 6276 | 3730.8 | 929747 | 77.1 |
| 7 | 558.8114 | 7002 | 1469.4 | 366195 | 30.4 |
| 8 | 1114.6111 | 7574 | 5810.3 | 889891 | 73.8 |
| 9 | 1115.6138 | 7742 | 3610.5 | 552209 | 45.8 |
| 10 | 1116.6170 | 8054 | 1219.7 | 186492 | 15.5 |

| # | m/z | Res. | S/N | I | I % |
|---|---|---|---|---|---|
| 1 | 341.3048 | | | 302844 | 14.5 |
| 2 | 353.2668 | | | 137317 | 6.6 |
| 3 | 359.3146 | | | 203237 | 9.7 |
| 4 | 381.2964 | | | 232046 | 11.1 |
| 5 | 1114.6111 | | | 2095025 | 100.0 |

**Figure S72.** HRMS-ESI of the **5a** metabolite corresponding to 2′-*O*-deacetyl-**5a**.

# Compound Spectrum List Report

**Analysis Info**

| | | | |
|---|---|---|---|
| Analysis Name | D:\Data\AST\AST-1.d | Acquisition Date | 1/12/2024 12:16:22 PM |
| Method | tune_norm.m | Operator | Mitrokhov |
| Sample Name | Tune wide | Instrument / Ser# | micrOTOF-Q II 10225 |
| Comment | | | |

**Acquisition Parameter**

| | | | | | |
|---|---|---|---|---|---|
| Source Type | ESI | Ion Polarity | Positive | Set Nebulizer | 0.4 Bar |
| Focus | Not active | Set Capillary | 4500 V | Set Dry Heater | 180 °C |
| Scan Begin | 50 m/z | Set End Plate Offset | -500 V | Set Dry Gas | 4.0 l/min |
| Scan End | 3000 m/z | Set Collision Cell RF | 550.0 Vpp | Set Divert Valve | Source |

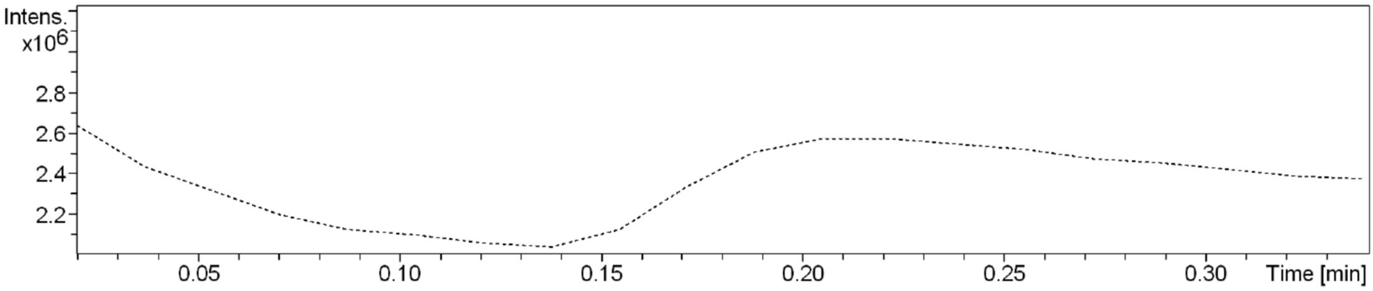


| # | RT [min] | Area | Int. Type | Intens. | S/N | Chromatogram | Max. m/z |
|---|---|---|---|---|---|---|---|
| n.a. | 0.2 | n.a. | Average spectrum | n.a. | n.a. | n.a. | 571.8246 |

**+MS, 0.1-0.3min #(4-17)**

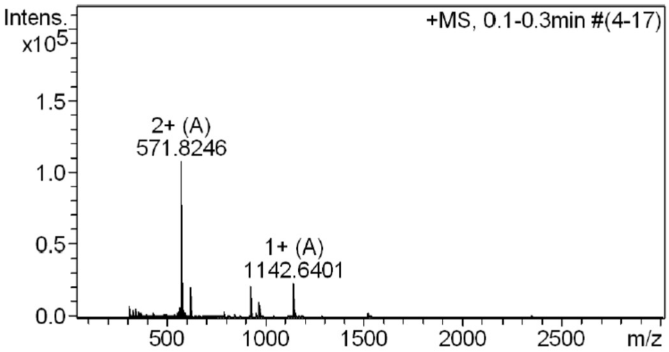


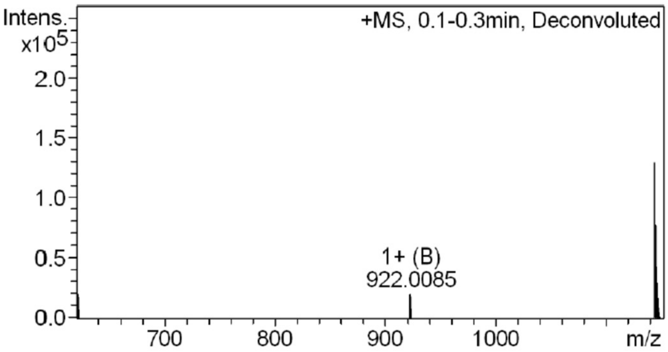


| # | m/z | Res. | S/N | I | I % |
|---|---|---|---|---|---|
| 1 | 313.2730 | 5666 | 114.4 | 7321 | 6.8 |
| 2 | 571.8246 | 6549 | 1082.5 | 107576 | 100.0 |
| 3 | 572.3260 | 6469 | 672.4 | 66871 | 62.2 |
| 4 | 572.8285 | 6552 | 252.5 | 25253 | 23.5 |
| 5 | 573.3280 | 6348 | 75.1 | 7696 | 7.2 |
| 6 | 622.0268 | 6863 | 219.7 | 20292 | 18.9 |
| 7 | 922.0086 | 7642 | 323.5 | 21837 | 20.3 |
| 8 | 967.5352 | 7679 | 157.0 | 10648 | 9.9 |
| 9 | 1142.6401 | 7638 | 359.7 | 23357 | 21.7 |
| 10 | 1143.6425 | 7670 | 231.5 | 15110 | 14.0 |

| # | m/z | Res. | S/N | I | I % |
|---|---|---|---|---|---|
| 1 | 622.0265 | | | 20292 | 15.5 |
| 2 | 922.0085 | | | 21836 | 16.7 |
| 3 | 1142.6413 | | | 130933 | 100.0 |

**Figure S73.** HRMS-ESI of the **5c** metabolite corresponding to 2′-*O*-deacetyl-**5c**.

**Table S3.** Antibacterial activity (MIC, µg/mL) of AZT–MNZ conjugates **5a, 5c** and their 2′-*O*-deacetyl metabolites.

| Strain | AZT | **5a** | 2′-*O*-deacetyl-**5a** | **5c** | 2′-*O*-deacetyl-**5c** |
|---|---|---|---|---|---|
| *S. pneumoniae* ATCC 49619 | 0.03 | 0.125 | 0.125 | 0.25 | 0.125 |
| *S. pneumoniae* ATCC 6305 | 0.03 | 0.125 | 0.125 | 0.125 | 0.125 |
| *S. agalactiae* 1Cp | 0.03 | 0.5 | 1 | 1 | 1 |

# Supplementary Methods

*Analysis of Kinetic Stability*

The analysis of kinetic stability of azithromycin derivatives **4a**, **5a** and **5c** was carried out essentially as previously described [1]. Briefly, hybrid molecules **4a**, **5a**, **5c** (1 mg/mL) were incubated in isotonic phosphate buffer (PBS, pH 7.4) at 37 °C during 0, 1, 2 and 24 h. The ionic strength of the buffer solution was adjusted to 0.5 by adding the calculated amount of KCl. The aliquots at appropriate incubation intervals were analyzed by HPLC, system (A): A – $HCO_2NH_4$ (0.2%) pH = 4.5, B – MeCN, concentration of B varied from 40% to 90% for 30 min. The peaks of metabolites were collected and analyzed using HRMS (ESI).

## Supplementary References

1. Mahfouz, N.M.; Aboul-Fadl, T.; Diab, A.K. Metronidazole twin ester prodrugs: synthesis, physicochemical properties, hydrolysis kinetics and antigiardial activity. *European Journal of Medicinal Chemistry* **1998**, *33*, 675-683, doi: 10.1016/S0223-5234(98)80026-3.
